# Analysis of temporal and spatial changes in the global burden of hypertensive heart disease based on data from the Global Burden of Disease study database and future projections: 1990–2046

**DOI:** 10.3389/fcvm.2025.1540816

**Published:** 2025-12-09

**Authors:** Guoliang Gao, Hui Yang, Guoping Yan, Minqiang Bao, Xuefen Guo, Zhaoyi Chen

**Affiliations:** 1 Department of Electrophysiology, Xuancheng People’s Hospital (Affiliated Xuancheng Hospital of Wannan Medical College), Anhui, China; 2 Department of Critical Care Medicine, Xuancheng People’s Hospital (Affiliated Xuancheng Hospital of Wannan Medical College, Anhui, China; 3 Department of Neurology, Xuancheng People’s Hospital (Affiliated Xuancheng Hospital of Wannan Medical College), Anhui, China; 4 Department of Gastroenterology, Xuancheng People’s Hospital (Affiliated Xuancheng Hospital of Wannan Medical College), Anhui, China

**Keywords:** hypertensive heart disease, Global Burden of Disease (GBD), Bayesian APC modeling, frontier analysis, decomposition analysis

## Abstract

**Background:**

Hypertensive heart disease remains a growing global health challenge, especially in regions with limited socioeconomic development. Understanding how its prevalence, mortality, and disability burden have changed over time is crucial for guiding prevention and control strategies. This study analyzed global trends from 1990 to 2021, examined differences by sex and development level, and projected future patterns to 2046.

**Methods:**

Utilizing data from the Global Burden of Disease (GBD) study, we examined patterns in the occurrence, death rates, and DALYs of hypertensive heart disease from 1990 to 2021; analyzed the time-based patterns of the average annual percentage change (AAPC) through joinpoint regression models; and evaluated the effects of aging, demographic expansion, and epidemiological shifts in hypertensive heart disease burden by integrating frontier and decomposition studies. An assessment of hypertensive heart disease burden and Bayesian age-period-cohort (APC) modeling techniques were employed to forecast future patterns.

**Results:**

From 1990 to 2021, the worldwide incidence of hypertensive heart disease increased from 4,626,598 to 12,505,436 cases, accompanied by a 0.53% AAPC; the number of hypertensive heart disease-related deaths increased from 713,935 to 1,332,099 cases, with a −0.79% AAPC; and the number of DALYs increased from 15,473,830 to 25,462,185 years, with a −0.95% AAPC. The incidence in females was marginally greater than that in males, yet there were comparable decreases in deaths and DALYs for both sexes. There was a notable increase in prevalence in regions with high SDIs, whereas areas with low SDIs experienced greater disease burdens. The increasing worldwide burden of hypertensive heart disease is attributed primarily to aging and population growth. According to the APC model, the worldwide incidence of hypertensive heart disease is expected to increase from 2022 to 2046, with further decreases in deaths and DALYs.

**Conclusions:**

This study systematically reveals the increasing trend in the incidence of hypertensive heart disease globally between 1990 and 2021 and confirms the significant impact of sex and socioeconomic development level on its burden. Forecasts to 2046 indicate that the prevalence will continue to rise, although mortality may decline, achieving the research objective of exploring the global and regional epidemiological characteristics and future trends of hypertensive heart disease.

## Introduction

The prevalence of hypertension, a major health problem worldwide, has increased significantly over the past decades, resulting in a significant disease burden, driven especially by aging and lifestyle changes (1). Hypertensive heart disease, one of the major complications of hypertension, has become the second leading cause of heart failure worldwide and significantly increases the risk of stroke, coronary heart disease, and all-cause mortality (2). The World Health Organization (WHO) has reported a substantial increase in worldwide hypertension rates in recent decades. The number of individuals with hypertension worldwide increased from approximately 594 million in 1975 to approximately 1.3 billion by 2019 (3). Hypertensive heart disease (4) is the primary complication of hypertension. hypertensive heart disease encompasses a variety of conditions in which the structure and functionality of the heart become irregular due to persistently high blood pressure (5, 6). Hypertensive heart disease affects mainly middle-aged and older people older than 65 years, especially men and older women, and the risk is greater among those with lower socioeconomic status. Patients often present with symptoms such as chest pain and breathlessness, which may lead to heart failure in severe cases. Globally, hypertensive heart disease ranks as the second primary cause of heart failure, accounting for 26.2% of all heart failure cases (6). hypertensive heart disease is projected to become the second most common cause of heart failure worldwide by 2020. As of 2020, hypertensive heart disease, along with various other noncommunicable heart diseases, is projected to be among the leading causes of death and disability worldwide (7). hypertensive heart disease represents not only a primary target organ of hypertension but also a significant independent risk factor for stroke, coronary heart disease, and overall mortality (8). Given the long-term characteristics of hypertensive heart disease, many patients experience poverty because of the illness, imposing significant financial strain on their families (9). Moreover, long-term antihypertensive treatment has been consistently shown to reduce the risks of stroke, coronary heart disease, heart failure, and all-cause mortality. Large-scale randomized controlled trials and international guidelines strongly support that the overall benefits of sustained blood pressure reduction far outweigh the potential adverse effects (10). Although some antihypertensive drugs may cause side effects such as electrolyte disturbances, cough, or fatigue, which can influence adherence and, in rare cases, aspects of health-related quality of life, these risks remain minor compared with the substantial cardiovascular protection conferred by effective blood pressure control. Despite these facts and the substantial increase in the worldwide hypertensive heart disease burden (11), along with notable variances among nations, possibly owing to trends in environmental, dietary, and sociodemographic characteristics (12), the epidemiology of hypertensive heart disease remains a critical issue, necessitating more comprehensive research and understanding.

Global studies on the epidemiological characteristics of hypertensive heart disease are still relatively limited. The southwestern region of Cameroon in Africa (13) and the Somali Region (14) presented the highest proportion of echocardiographically confirmed heart disease, accounting for approximately 40% of cases. Nilay et al. analyzed death statistics in the United States from 1999 to 2018 and reported a significant increase in the proportion of all deaths from heart disease, from 4% to 9%, with the fastest rate of increase among white men (15). However, these limited single-center or single-region studies are insufficient to adequately inform global public health policy development. Although previous studies reported a global overview of hypertensive heart disease epidemiology from 1990 to 2019 (1, 11, 16), in this paper, following several previous studies on the global epidemiology of hypertensive heart disease using GBD data, some important shortcomings are still visible. Most of the current analyses depict overall prevalence, mortality and DALY trends instead of providing a clear separation of the underlying causes of these changes. This includes the extent to which population aging, together with demographic growth and epidemiological shifts, can influence or confound existing patterns. Furthermore, few studies have looked in detail at geographic variations or considered how the developmental level of affluence shapes the burden of hypertensive heart disease over time. Additionally, little work has been done on projecting developments in hypertensive heart disease beyond 2019. Such uncertainties mean that sources that could provide better details concerning the factors behind global and regional differences in hypertensive heart disease are lost and that their use to guide focused innovation is limited. Therefore, the present paper sought to conduct a systematic survey of patterns and trends in hypertensive heart disease at the global, regional and sex-specific levels within the period from 1990 to the manuscript publication date (2021), using decomposition and frontiers analyses together with Bayesian age–period–cohort modeling. It is hoped that these methods can be used to make accurate predictions to 2046.

## Methods

Earlier works (17–19) have provided in-depth accounts of the methods employed in GBD studies. In the GBD 2021 study, the effects of 371 diseases and injuries, including their frequency, prevalence, deaths, and disability-adjusted life years (DALYs), in 204 countries and areas from 1990 to 2021 were reported and organized by sex and age. By employing the Global Health Data Exchange query tool (http://ghdx.healthdata.org/gbd-results-tool) (20), we collected information on the frequency, deaths, and DALYs of hypertensive heart disease, including their 95% uncertainty intervals (UIs), and associated rates adjusted for sex and age from 1990 to 2021. Because we used a publicly available dataset, we did not obtain approval from the Ethics Committee of Xuancheng People's Hospital in Anhui Province. We employed DisMod-MR software v2.1 and Bayesian Regularized Trimming (MR-BRT) software v2.1.1 for disease modeling, along with R 4.3.3 for additional analyses and visualization. These specifications provided a robust environment for developing and testing our models. The user interface for each indicator (21) was developed through posterior distributions, fluctuating between the 25th- and 975th-order values over 1,000 posterior extractions. The sociodemographic index (SDI) acts as a collective measure for evaluating the developmental phase of a country or region, considering factors such as birth rates, educational level, and per capita income; SDI scores range from 0 to 1, with higher SDI scores indicating improved socioeconomic status in the country or region. A link exists between the SDI and the incidence of disease illness and death (22). Countries and regions were categorized into five SDI groups—high, medium–high, medium, medium–low, and low—based on their sociodemographic index scores, which combine income per capita, education levels, and fertility rates. This classification enables a detailed analysis of the link between hypertensive heart disease and economic development, ensuring that the study's findings can be replicated by other researchers. Joinpoint regression techniques were employed to examine time-based trends in hypertensive heart disease epidemiological characteristics at the global, regional, and national levels. In this research, crucial trend shift points (specifically, joinpoints) were identified, the overall trend was categorized based on these identified points, and the epidemiological trajectory of each category was assessed by calculating the annual percentage change (APC) and its 95% confidence interval (CI). Additionally, the average annual percentage change (AAPC) functions as the aggregated metric for predetermined time intervals (1990–1999, 2000–2009, 2010–2021, and 1990–2021), which is calculated by averaging the APC across different categories (23). Maintaining asymptotic significance, the 449 randomly shuffled datasets underwent Monte Carlo permutation and Bonferroni modification. When the forecasted figures for the APC or AAPC, coupled with the lowest point of its 95% CI, surpassed 0, an upward trajectory was observed over the period. Conversely, a downward trend was observed during the period when the forecasted figures for the APC or AAPC and the peak 95% CI remained under 0. In addition, the trend was considered consistent (24). Studies of the frontier technique and its breakdown revealed three principal factors affecting changes in prevalence, deaths, and DALYs between 1990 and 2021: the aging process, population growth, and epidemiological changes (19). Epidemiological changes refer to changes in deaths and prevalence rates after adjustments for age and population size. To define hypertensive heart disease, codes associated with the International Classification of Diseases, Ninth (ICD-9) and Tenth (ICD-10) editions were utilized. Diseases classified under the ICD-9 codes 402–402.91 or the ICD-10 codes I11–I11.9 were considered hypertensive heart disease. Previous research (25) has thoroughly recorded the process of data selection utilizing these ICD codes.

### DALYs

The term “DALYs” amalgamates disease burden measurements, merging the total years of life lost (YLLs) due to premature death with the years of healthy life lost due to disease or disability (YLDs). The method to determine DALYs is outlined as follows:

### DALYs = YLLs + YLDs

YLLs due to premature death refer to the total years lost due to early mortality, computed as outlined below:

### YLLs = *N* × L

where N represents the number of deaths and L denotes the variance between the standardized life expectancy and the age at death.

YLDs represent the total years of healthy life lost due to disease or impairment and are computed as follows:

### YLDs = I × DW × L

In this context, “I” represents the episode count, “DW” indicates the disease or disability weight, and “L” is either the disease duration or the extent of disability (26).

### Joinpoint regression model

Joinpoint regression modeling, a statistical technique designed to detect points of trend shifts in time series data, is frequently employed to examine disease burden patterns. Our research employed a joinpoint regression model to pinpoint pivotal trend shifts in hypertensive heart disease incidence, deaths, and DALYs from 1990 to 2021. The specific procedures were as follows: 1. Preparation of the data: Time series data on hypertensive heart disease incidence, mortality, and DALY information were organized by year. 2. Model construction: Using Joinpoint software (version 4.9.0.0), a regression model was formulated (by the U.S. National Cancer Institute) for identifying fluctuations in the sequential data. Every alteration point indicated a notable shift within a trend. 3. For every time frame, the APC along with its 95% CI was determined to evaluate the pattern within each period. If the APC was substantially greater than zero, it signified a marked trend shift during that specific timeframe (27). The differences were deemed statistically significant when P was less than 0.05.

### Decomposition analysis model

Decomposition analysis serves as a statistical technique aimed at pinpointing and quantifying the impact of various elements on total changes. In this research, a decomposition analysis was performed to evaluate how aging, population growth, and epidemiological shifts influence alterations in hypertensive heart disease burden (28). In particular, we segmented the fluctuations in the burden of hypertensive heart disease into three key aspects: shifts in demographic dynamics (encompassing aging and population growth), shifts in epidemiological patterns (covering risk factors and enhanced disease control), and impacts of medical treatment. The procedure for the decomposition analysis was as follows: 1. Preparation of the data: hypertensive heart disease burden information was grouped based on age, sex, and SDI. 2. Development of the decomposition model: The model integrates detailed demographic and epidemiological data and medical intervention information from the Global Burden of Disease Study and other international databases and uses mathematical techniques such as linear regression and Bayesian decomposition to accurately assess the specific contributions of these factors to fluctuations in the burden of hypertensive heart disease, thus providing a solid foundation for understanding and predicting disease trends. 3. Results evaluation: The proportional impact of various factors on the variation in hypertensive heart disease burden was calculated, with the findings being authenticated through statistical analysis to confirm the importance of the study results (29).

### Frontier analysis

Frontier analysis (FA) is a statistical method used to assess the performance of different regions or groups of people in terms of health outcomes and usually involves data envelopment analysis (DEA) or stochastic frontier analysis (SFA) techniques (30). In this study, we used DEA to assess the efficiency and effectiveness of hypertensive heart disease prevention and treatment in different SDI regions. The specific steps were as follows: 1. Data preparation: hypertensive heart disease burden data (prevalence, mortality, and DALYs) and corresponding health care resource data (e.g., number of doctors, number of hospital beds, and health expenditures) for each region were organized into panel data. 2. DEA model construction: An input-oriented DEA model was used, in which the health care resources in each region were used as the input variables, the hypertensive heart disease burden data were used as the output variables, and the efficiency scores of each region were calculated. 3. Analysis of results: The efficiency scores of each region were compared, regions with higher and lower efficiencies were identified, and the factors that led to differences in efficiency were analyzed (31).

### Bayesian APC prediction model

Bayesian APC prediction modeling is a statistical method for analyzing and predicting disease trends. By integrating age effects, period effects, and cohort effects, APC models can analyze disease dynamics comprehensively and predict future trends. The Bayesian APC model estimates and predicts age, period and cohort effects through Bayesian statistical methods. The model can decompose temporal changes in disease incidence or mortality, providing a detailed analysis of disease trends and future projections. These data are typically derived from national or global health databases such as the GBD study database (32). Commonly used methods include residual analysis, prediction testing, and cross-validation. Through these methods, future disease trends can be predicted via Bayesian APC models. The posterior distributions of the model parameters allow future morbidity, mortality, or DALYs to be simulated, and predictions and their uncertainty ranges can be obtained. These predictions provide a scientific basis for the development of public health policies and control strategies (32).

### Statistical analysis

In this study, the data are presented as the means ± standard deviations. For link-point regression analyses, a two-sided test with a significance level of 0.05 was used to identify significant trend change points. R software (version 4.3.3) was used for all the statistical analyses and graphical representations; specifically, the environment was set up via the “configr” package. Data cleaning and calculations were carried out via the “dplyr”, “tidyr” and “purrr” packages for data cleaning and computation, and the “ggplot2” package was used for data visualization. The significance threshold for all analyses was set at *p* < 0.05 to ensure that the results were statistically significant.

## Results

### Global, sex and regional trends

The global hypertensive heart disease incidence increased from 46.3 million cases (125.44 per 100,000 people) in 1990 to 1.25 billion cases (1,482.20 per 100,000 people) in 2021. The annual percentage change was 0.53% (95% CI: 0.49–0.58). During the same period, the number of deaths decreased from 713,935 cases (20.92 per 100,000) to 133,299 cases (16.32 per 100,000). The annual percentage change was −0.79% (95% CI: −0.92‒−0.65). DALYs also decreased, from 154.7 million (406.51 per 100,000) in 1990 to 254.6 million (301.58 per 100,000). The annual percentage change was −0.95% (95% CI: −1.04‒−0.86) (Table 1).

**Table 1:** Sex prevalence, mortality, disability-adjusted life years, and annual percentage changes in hypertensive heart disease by sociodemographic Index quintile (AAPC), 1990–2021.

Category	Prevalence					Mortality					DALYs				
	Num_1990	ASRs_1990	Num_2021	ASRs_2021	AAPC_CI	Num_1990	ASRs_1990	Num_2021	ASRs_2021	AAPC_CI	Num_1990	ASRs_1990	Num_2021	ASRs_2021	AAPC_CI
Global	4626598 (3672198–5826592)	125.44 (98.97–157.96)	12505436 (9866066–15827877)	148.32 (117.32–186.28)	0.53 (0.49–0.58)	713935 (577534–795258)	20.92 (17.14–23.21)	1332099 (1121131–1468852)	16.32 (13.76–18.01)	−0.79 (−0.92–0.65)	15473830 (12310725–17311822)	406.51 (328.94–452.24)	25462185 (21493312–28047521)	301.58 (255.06–332.06)	−0.95 (−1.04–0.86)
Sex															
Female	2491731 (1974900–3138969)	120.81 (95.61–151.86)	6803640 (5402144–8533746)	146.65 (116.97–183.69)	0.62 (0.59–0.65)	415681 (318739–479995)	21.2 (16.38–24.32)	774445 (625933–881245)	16.52 (13.36–18.78)	−0.80 (−0.91–0.68)	8465473 (6330282–9939344)	406.48 (304.77–474.79)	13823618 (10900327–15529129)	299.26 (235.38–336.07)	−0.98 (−1.06–0.90)
Male	2134867 (1696412–2689436)	130.61 (101.97–165.1)	5701796 (4406254–7277011)	148.86 (116.37–189.08)	0.40 (0.38–0.43)	298254 (234225–337630)	20.26 (16.09–22.59)	557655 (436353–643661)	15.85 (12.37–18.26)	−0.80 (−0.1.01–0.59)	7008357 (5503297–7984000)	402.85 (317.39–456.01)	11638567 (9333223–13404664)	301.41 (240.86–345.2)	−0.93 (−1.05–0.81)
SDI region															
High SDI	845598 (645691–1085375)	77.16 (59.92–98.08)	2436473 (1878839–3082797)	113.36 (88.38–139.86)	1.23 (1.19–1.27)	93390 (85036–98125)	8.56 (7.74–9.01)	189010 (157234–209886)	7.7 (6.54–8.53)	−0.30 (−0.60–0.00)	1695962 (1583912–1767114)	155.64 (145.29–162.29)	3098667 (2717718–3397624)	149.35 (134.5–162.92)	−0.09 (−0.39–0.22)
High–middle SDI	992323 (766890–1275711)	107.7 (83.52–137.23)	2740903 (2119692–3504373)	139.85 (108.6–177.78)	0.85 (0.79–0.92)	145582 (128528–161927)	17.83 (15.57–19.73)	275365 (237571–312978)	14.6 (12.53–16.52)	−0.65 (−0.90–0.40)	2891667 (2552953–3233035)	315.23 (278.3–350.96)	4389745 (3890115–4989434)	228.02 (201.54–258.5)	−1.02 (−1.25–0.78)
Low–middle SDI	741968 (598365–921355)	145.51 (115.15–181.68)	1943003 (1538779–2439288)	151.46 (118.98–192.39)	0.13 (0.12–0.14)	132745 (95311–158999)	28.04 (21.09–33.87)	274886 (225956–315309)	23.15 (19.06–26.62)	−0.56 (−0.86–0.25)	3111599 (2200759–3753608)	545.44 (390.96–652.79)	5955402 (4908448–6812461)	438.48 (364.14–503.27)	−0.67 (−0.86–0.47)
Middle SDI	1666506 (1322121–2075894)	189.85 (150.56–235.42)	4456763 (3493912–5664364)	178.07 (140.42–225.7)	−0.23 (−0.30–0.16)	270963 (190727–306789)	35.19 (25.55–39.43)	461730 (349647–546063)	20.26 (15.3–23.96)	−1.79 (−2.00–1.57)	6002937 (4223843–6811307)	645.06 (459.8–729.11)	8930493 (6909201–10413694)	355.67 (273.61–414.56)	−1.91 (−2.02–1.80)
Low SDI	375324 (290235–477935)	202.28 (158.28–263.13)	915835 (705715–1160392)	208.42 (162.49–268.79)	0.10 (0.08–0.13)	70361 (46598–89469)	40.54 (28.07–50.81)	129352 (90728–159533)	33.58 (24.66–40.58)	−0.58 (−0.72–0.44)	1753192 (1128631–2257732)	817.25 (545.8–1035.65)	3055862 (2148436–3826137)	640.71 (451.46–786.79)	−0.76 (−0.86–0.66)

DALYs, disability-adjusted life-years; ASR, age-standardized rate; 95% UI, 95% uncertainty interval; AAPC, average annual percentage change; SDI, sociodemographic Index.

**Figure 1:**
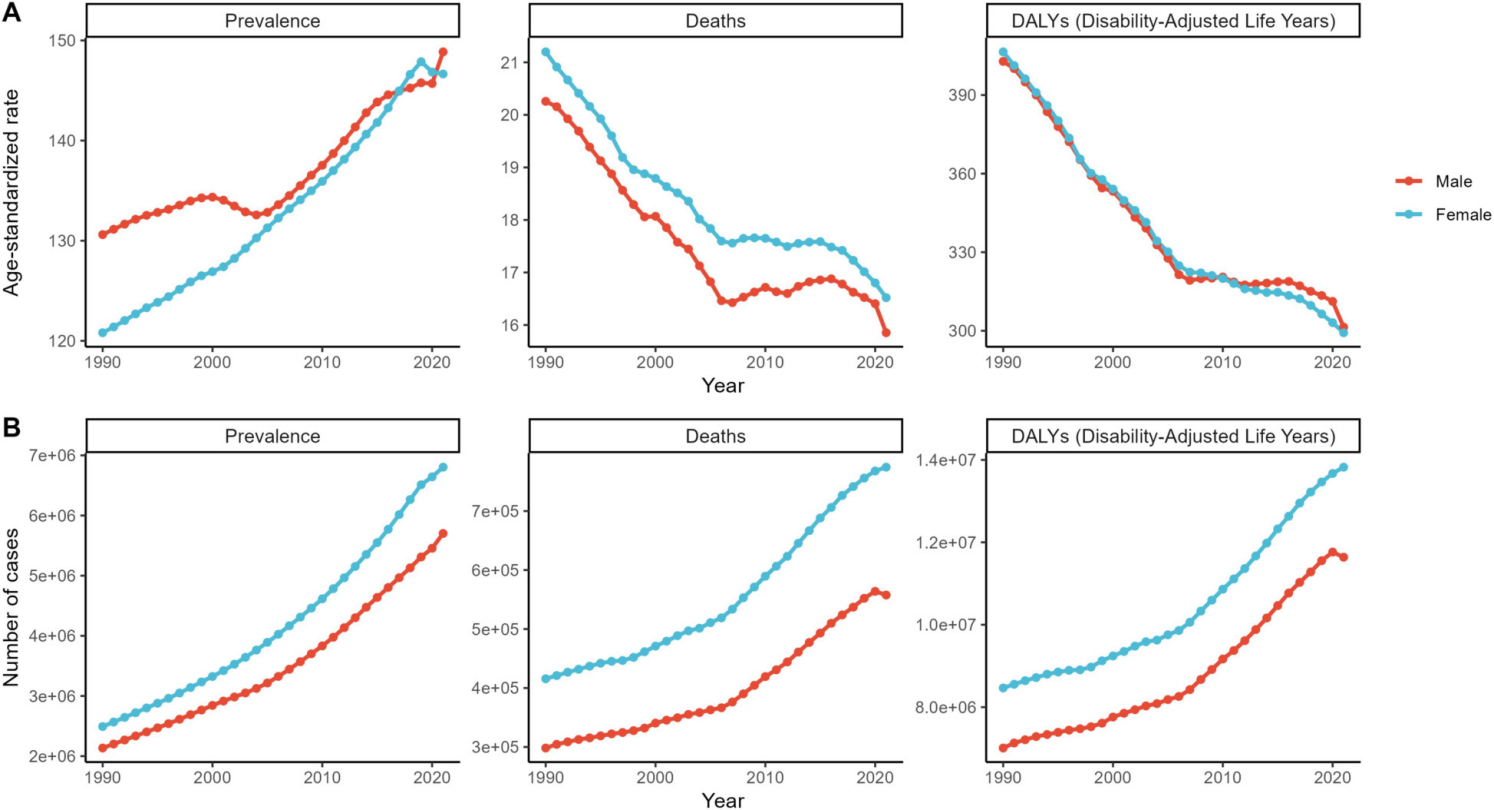
Annual trends in the prevalence of hypertensive heart disease, deaths and disability-adjusted life years by sex, 1990–2021. Worldwide patterns in the ASR **(A)** and shifts in the number **(B)** of individuals with hypertensive heart disease, encompassing the prevalence, deaths, and disability-adjusted life years (DALYs), from 1990 to 2021.

When the data were examined by sex, the rate of increase in the prevalence of hypertensive heart disease was slightly faster among women (AAPC 0.62%) than among men (AAPC 0.40%), yet men remained at the top in terms of absolute numbers. However, there was little difference between the two sexes in terms of reductions in death or disability-adjusted life years (DALYs) (Table 1, Figures 1A,B).

**Figure 2:**
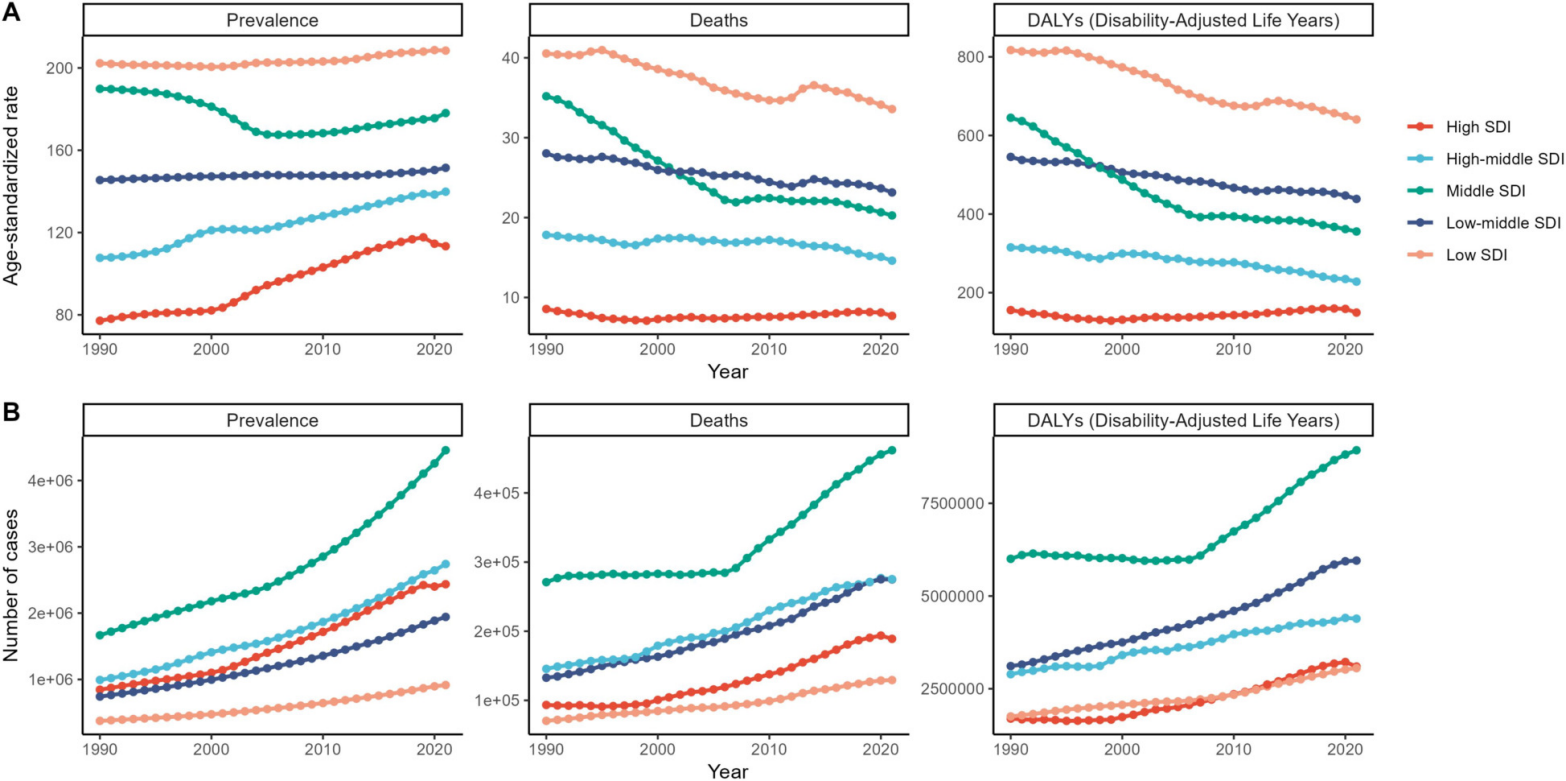
Prevalence of hypertensive heart disease, deaths across various sociodemographic index quintiles from 1990 to 2021, and yearly patterns in disability-adjusted life years. Patterns in the ASR **(A)** and variations in the number **(B)** of individuals with hypertensive heart disease across various sociodemographic index quintiles, encompassing the prevalence, deaths, and disability-adjusted life years (DALYs), from 1990 to 2021.

Prevalence within high-SDI strata increased most rapidly (AAPC 1.23%), a trend likely fueled by better detection and reporting; however, deaths and DALYs decreased only slightly in these areas. Prevalence and deaths increased as DALYs remained persistently high in low-SDI regions, highlighting persistent challenges for health (Table 1, Figures 2A,B).

### National trends

**Figure 3:**
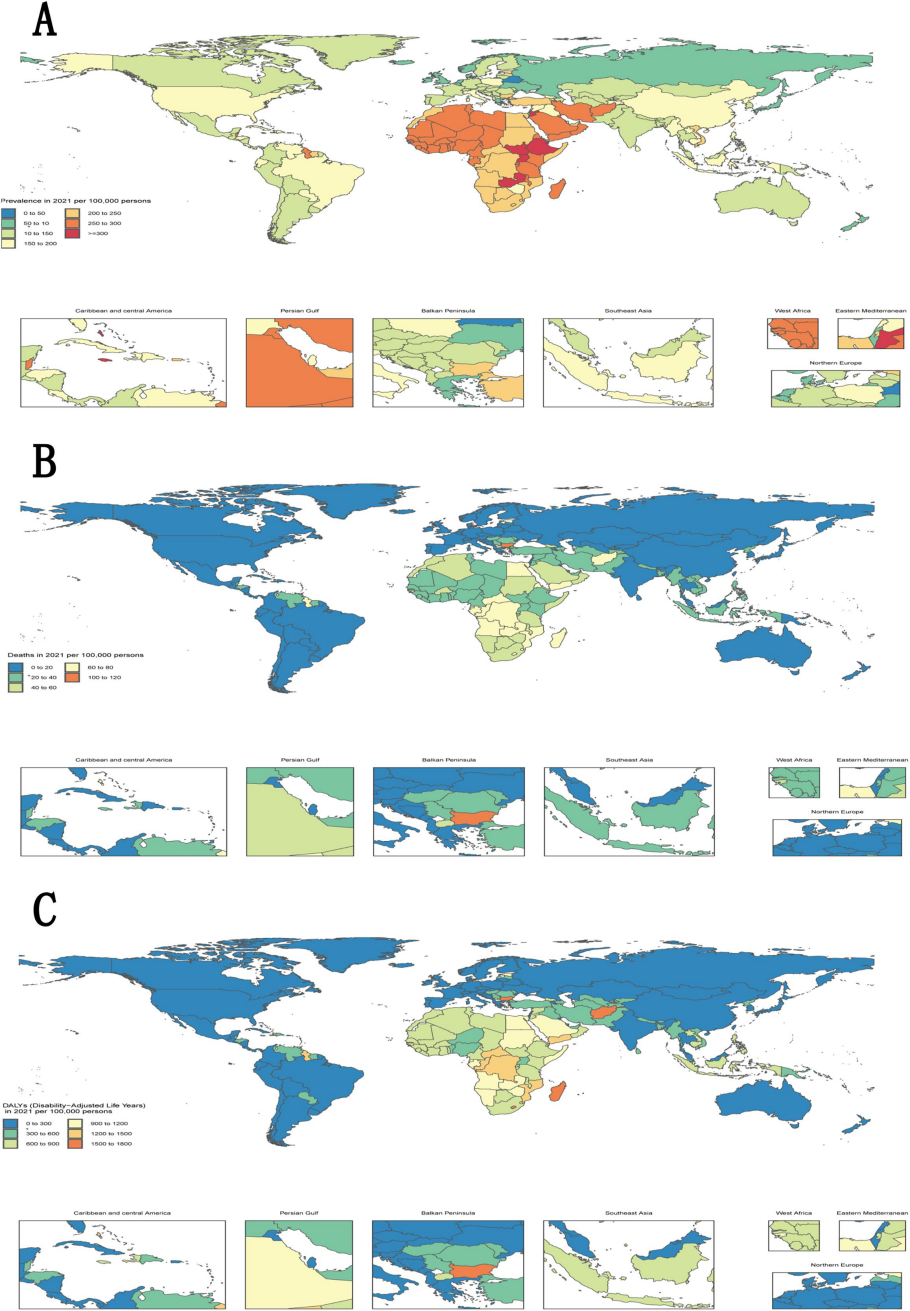
Worldwide yearly patterns in the incidence, deaths, and disability-adjusted life years of hypertensive heart disease, 2021. The worldwide spread of the incidence **(A)**, deaths **(B)**, and disability-adjusted life years (DALYs) **(C)** of hypertensive heart disease in 2021, highlighting notable disparities among nations.

In 2021, the global prevalence of hypertensive heart disease (95% UI: 117.3–186.3) was 148.3 cases per 100,000 individuals, with significant geographic variation in particular regions. The highest hypertensive heart disease prevalence was reported in Andean Latin America, with a regional average of 148.4 cases per 100,000 people; however, much lower values were reported for Australia (56.2) and New Zealand (32.2) (Figure 3A; Supplementary Table S1).

In total, there were 12.5 million fatalities (95% UI: 0.99–15.8 million) worldwide. In terms of probable death, the number of deaths was highest in Andean Latin America (85,962) but relatively low in Australia (27,202) (Figure 3B; Supplementary Table S2).

Worldwide, DALYs reached 25.5 million years (95% UI: 21.5∼28.0 million). Compared with Australia, Andean Latin America was home to many poor individual years lived with disability (ASR 151 vs. 39.4/100,000) (Figure 3C; Supplementary Table S3). The results underscore substantial regional disparities that require attention to protect public health.

### Time trends

**Figure 4:**
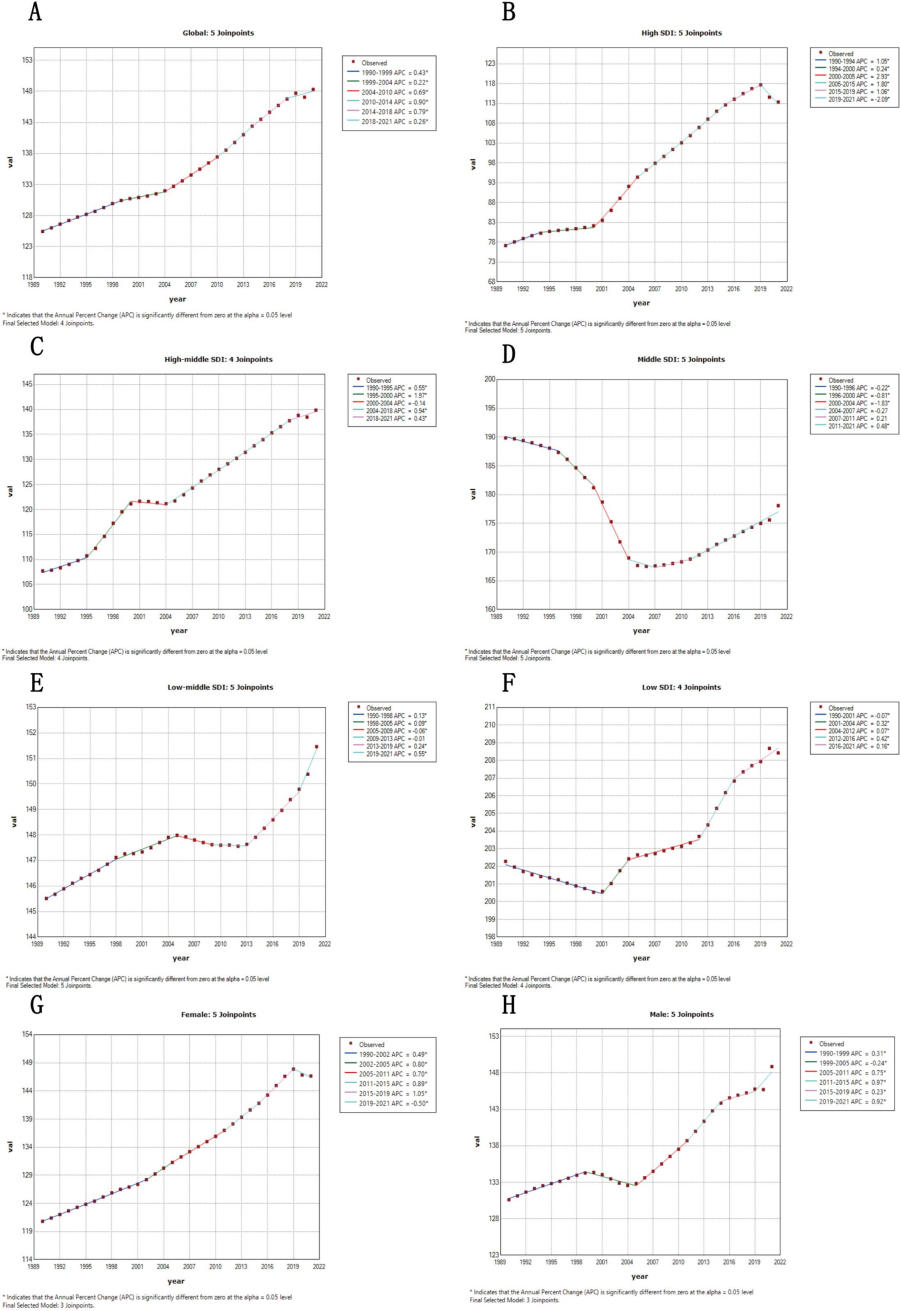
Global prevalence of hypertensive heart disease from 1990 to 2021, analyzed through joinpoint regression, stratified by sociodemographic index quintile and sex. Worldwide occurrence of heart disease related to hypertension in regions with a high sociodemographic index (SDI) **(A)**, regions with a medium–high SDI **(B)**, areas with a medium SDI **(C)**, regions with a low–middle SDI **(D)**, and regions with a low SDI **(E)** for both men **(F)** and women **(H)**, spanning from 1990 to 2021. Combined with point regression analysis.

Advanced regression analysis revealed a series of inflection points in the overall prevalence of hypertensive heart disease cases worldwide between 1990 and 2021. Among males, the prevalence increased sharply for men in 1995 and 2005 but slowed after 2015 (Supplementary Table S5.2), whereas for women, it increased rapidly from 1998 to 2000 and 2010 but decreased after 2018. In countries with high SDIs, the incidence of hypertensive heart disease quickly increased after 2000 but slowed after 2010; in countries with medium-to-high SDI, sharp increases occurred in approximately 2000 and 2010. In low-SDI areas, despite some weakness in prevention and limited health care resource availability (Figure 4), peripheral data updates were needed for several reasons.

Globally, the number of hypertensive heart disease-related deaths has decreased but is still significantly greater in men and in low-SDI areas. The greatest reductions in both deaths and DALYs occurred in high-SDI regions, whereas the decreases slowed or stagnated for low- or middle-SDI regions (Supplementary Figures S1, S2), highlighting the need for region-specific sex-specific health interventions.

### Trends in frontier analysis

**Figure 5:**
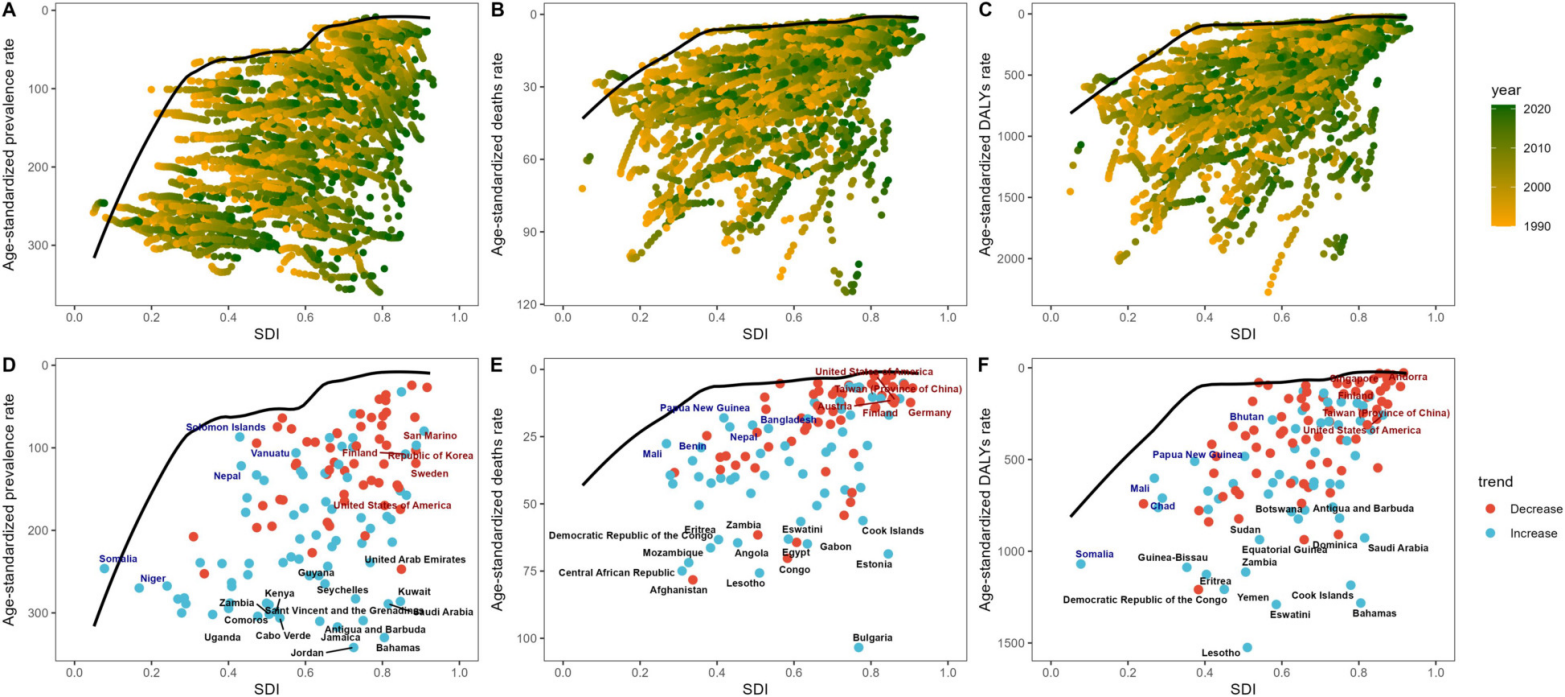
Pioneering examination of worldwide age-adjusted incidence rates, age-adjusted deaths, and the impact of hypertensive heart disease on DALYs from 1990 to 2021. Upper section: Preliminary examination of worldwide hypertensive heart disease incidence from 1990 to 2021, illustrating global patterns in hypertensive heart disease **(A)** deaths **(B)** and DALYs **(C)** Lower section: Preliminary examination of hypertensive heart disease incidence in nations with a high sociodemographic index (SDI) from 1990 to 2021, illustrating the patterns in prevalence among countries with a high SDI **(D)**, those with a medium‒high SDI **(E)**, and those with a medium‒low SDI **(F)**.

In the period from 1990 to 2021, the number of hypertensive heart disease standards declined globally in terms of age-adjusted prevalence, mortality and DALYs. These trends reflect widespread improvements in medical care, but great differences still exist across regions. For example, the prevalence rate ranged from high levels in places such as Jamaica (1990) and Jordan (2021) to very low levels in Norway and Iceland. In the Cook Islands and Lesotho, both mortality and DALYs consistently had the greatest burdens, whereas Sweden, Belgium, and Denmark had much lower burdens (Figure 5).

With respect to the SDI, high-SDI regions experienced only modest increases in prevalence and either steady or decreasing numbers of deaths and DALYs—indicating effective health systems. However, medium- and low-SDI countries experienced sharp increases in both the number of hypertensive heart disease cases and the number of deaths from hypertensive heart disease, highlighting real challenges to disease prevention and control. These results emphasize the impending need for health interventions to be considerably improved in low- and middle-income countries.

### Decomposing and analyzing trends

**Figure 6:**
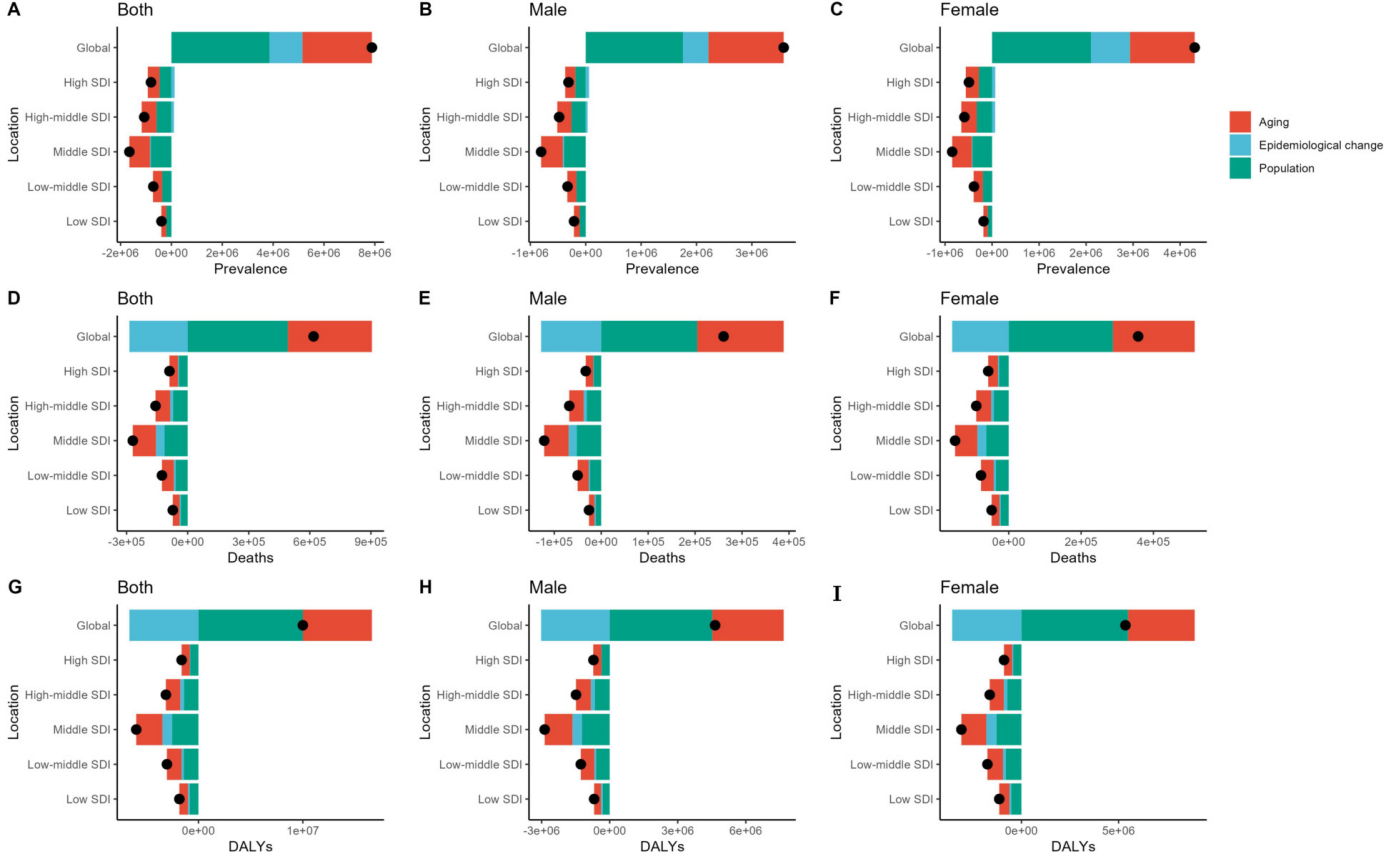
Analysis of the worldwide impact of hypertensive heart disease stratified by SDI and sex, 2021. Pioneering study of worldwide studies examining hypertensive heart disease incidence, with varying SDIs **(A)**, deaths **(D)**, and DALYs **(G)** reported in 2021. Pioneering examination of the worldwide occurrence of hypertension in men, featuring various SDIs **(B)**, deaths **(E)**, and DALYs **(H)** in 2021. Early studies examining the worldwide incidence of hypertensive heart disease across diverse SDIs **(C)** and deaths **(F)** among women in 2021. Pioneering evaluation of DALYs **(I)**.

As shown in an analysis of hypertensive heart disease burden in 2021 by SDI region, substantial variations were detected across different areas. In low- and low–middle-SDI regions, although the death rate was well under 50 years per 1000 people in 2021 (less than 10% of that in high-SDI areas), the prevalence increased dramatically in some cases and even remained at the same level as in previous years—a turnabout marked by population aging as well as shifting demographics. This region-specific burden showed sexual disparities: it was in low-SDI areas where women had a higher prevalence, whereas men suffered more from death rates in low- to middle-SDI areas, as noted in the report (Figure 6).

Overall, aging and demographic changes were the main causes of increased hypertensive heart disease burden in less developed areas. These findings clearly underscore the need for targeted interventions and resource allocation to reduce inequality and improve cardiovascular health outcomes.

### 2022–2046 forecast trends

**Figure 7:**
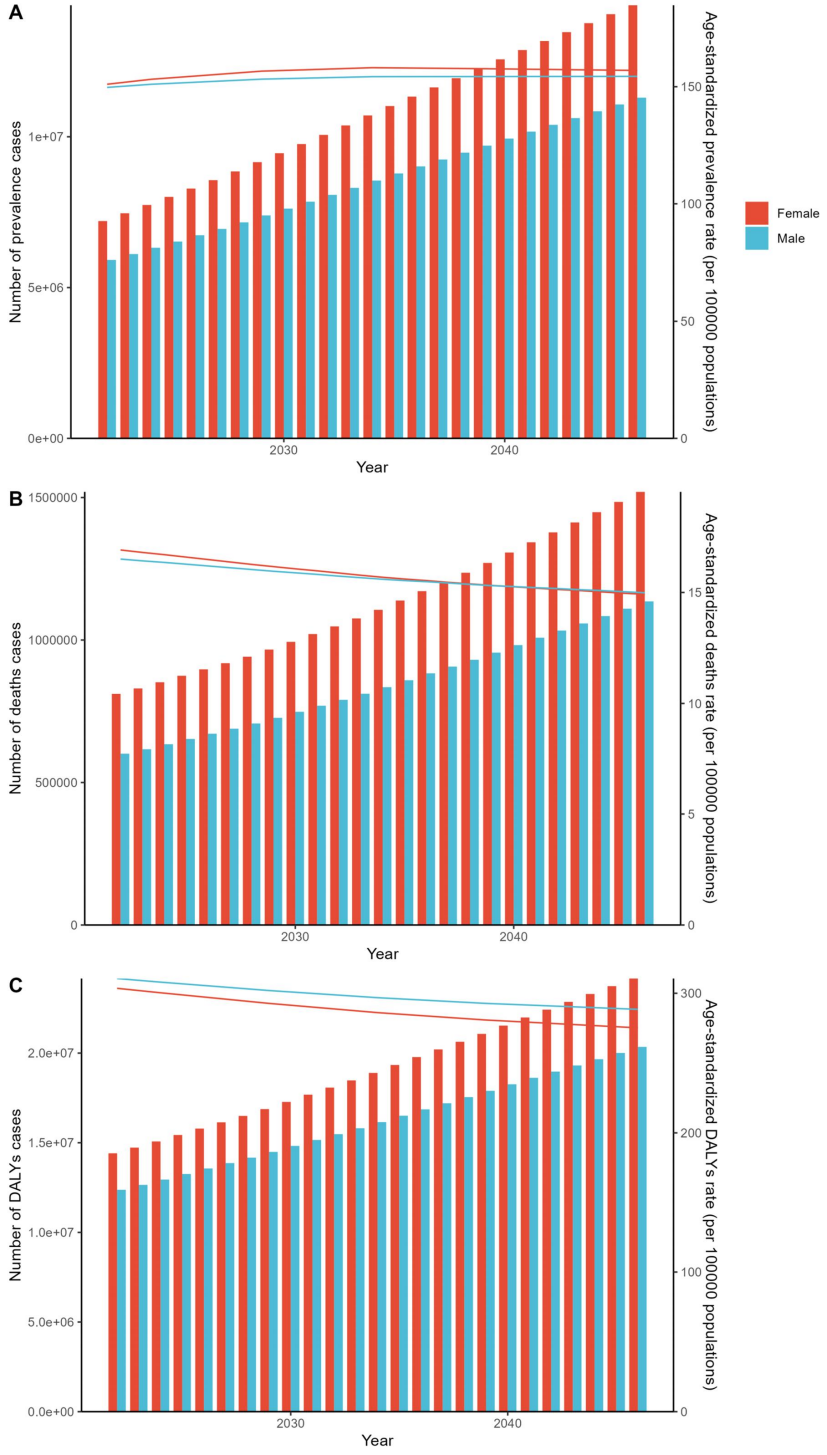
APC framework designed to forecast worldwide patterns in age-adjusted prevalence rates, deaths, and disability-adjusted life years (DALYs) for hypertensive heart disease, stratified by sex, spanning 2022–2046. Forecasted global patterns in age-adjusted (age-standardized prevalence rate) incidence rates **(A)**, deaths **(B)** and DALYs **(C)** for hypertensive heart disease in both sexes from 2022 to 2046.

The APC prediction model suggests that age's all-standard illness hypertensive heart disease (ICH) cases will increase slowly beginning in 2022; however, by 2048, the incidences of both death and YLL are expected to decrease. Although more people are likely to suffer throughout life from hypertensive heart disease, there is at least hope that continuous ongoing changes in treatment management will take some of its lethal load and disability burden away from those who suffer from the disease (Figures 7, Supplementary Table S4).

## Discussion

### Main findings

From 1990 to 2021, hypertension affected increasing numbers of people globally. However, that does not necessarily mean that it is becoming a significant threat to life; at high-speed economic levels, heart disease shows the same rate of development (19). With better health care present in high-SDI regions, there were more significant improvements in the situation. Conversely, low-SDI countries developed more slowly (11). Finally, these latest findings clearly indicate a shift in which more people live longer with hypertensive heart disease. This will create new requirements for the health care system.

### Case definitions

In the GBD league table, the definition of hypertensive heart disease depends upon whether its cause-of-death section records eleven illnesses (ICD-9 402.x) or twelve (I11.x), as in the ICD-10 (33). Although garbage codes are redistributed and DisMod-MR is used to enhance comparability (34), various diagnostic practices differ across different parts of the world. With few echoes and uncertain coding standards, misclassification risks are higher, especially in low-SDI areas (35). Such uncertainties may introduce bias into prevalence or mortality estimates, requiring care in their use and interpretation.

### Analytical assumptions

Methods that identify trends such as joinpoint and decomposition generally depend on the model's main assumptions (26). Forecasting is extended to 2046 to capture today's typical middle-aged cohorts as they continue to live into high-risk years. Future trends depend on current risk factors such as obesity, diabetes and lifestyle trends (36). Uncertainty intervals are required for any meaningful range in policy-based projections based on scenarios (37).

### Strengths and limitations

In this study, a standardized GBD approach across 204 locations was used, and various indicators were employed to corroborate the findings (19). The strengths include consistent definitions and comparable methods. Limitations emerge from variability in diagnostics, modeled estimates where there is little or no primary data and forecast uncertainty—particularly for middle- and low-SDI countries.

### Repercussions

The continuation of an increase in prevalence accompanied by a decrease in mortality indicates that more patients will now survive with chronic disease (38). Efforts should be directed toward early detection of hypertension, proper treatment and improvements in lifestyle. To reduce the disparity and stop the upward trend in the chronic burden of hypertensive heart disease, which is associated with low- and middle-SDI countries, well-targeted prevention and robust health systems are essential (34).

## Conclusions

Globally, hypertensive heart disease portrays a paradoxical picture: an expanding prevalence coupled with waning mortality and DALYs. This represents both improved survival and a growing chronic burden. Projections to 2046 suggest that the prevalence will continue to increase but that mortality associated with the disease will gradually decline. This means that more people will live longer despite having the disease. With respect to this score, regional and sex differences persist, and the burden is crowded by low- and middle-SDI countries. Strengthening hypertension control, preventive strategies and health system capacity—especially in low-resource areas—will be crucial for reducing inequalities in the future and the associated burden.

## Data Availability

The original contributions presented in the study are included in the article/Supplementary Material, further inquiries can be directed to the corresponding author.
