# Supplementary material for: Analysis of temporal and spatial changes in the global burden of hypertensive heart disease based on data from the Global Burden of Disease study database and future projections: 1990–2046
Source: Front Cardiovasc Med. 2025 Dec 9;12:1540816. doi: 10.3389/fcvm.2025.1540816 (PMC12722517; doi:10.3389/fcvm.2025.1540816)
Supplement: Supplementary file 1 [file Datasheet1.doc]

Figure S1: Joinpoint regression analysis of different socio-demographic index quintiles and different sex mortality rates for hypertensive heart disease globally from 1990 to 2021

Figure S2: Joinpoint regression analysis of disability-adjusted life years (DALYs) due to hypertensive heart disease globally by different socio-demographic index quintiles and sex, 1990-2021

Table S1: Prevalent cases of hypertensive heart disease in 1990 and 2021 and the percentage change in the age-standardized rates (ASRs) per 100,000 individuals by location

Table S2: Deaths due to hypertensive heart disease in 1990 and 2021 and the percentage change in the age-standardized rates (ASRs) per 100,000 individuals by location

Table S3: DALYs due to hypertensive heart disease in 1990 and 2021 and the percentage change in the age-standardized rates (ASRs) per 100,000 individuals by location

Table S4: APC model data for the predicted trends in the age-standardized prevalence, age-standardized mortality rate and DALYs for hypertensive heart disease (HHD) globally by sex 2022-2046

Table S5: Age-standardized prevalence and burden of hypertensive heart disease (HHD) worldwide (1990–2021) and frontier analysis results

Table S6: Global age-standardized DALY burden and frontier analysis of hypertensive heart disease (HHD), 1990-2021

Table S7: Age-standardized mortality burden and frontier analysis of hypertensive heart disease (HHD) worldwide, 1990–2021

Table S8: 2021 global and breakdown analysis of the burden of hypertensive heart disease (HHD) by SDI and sex

**
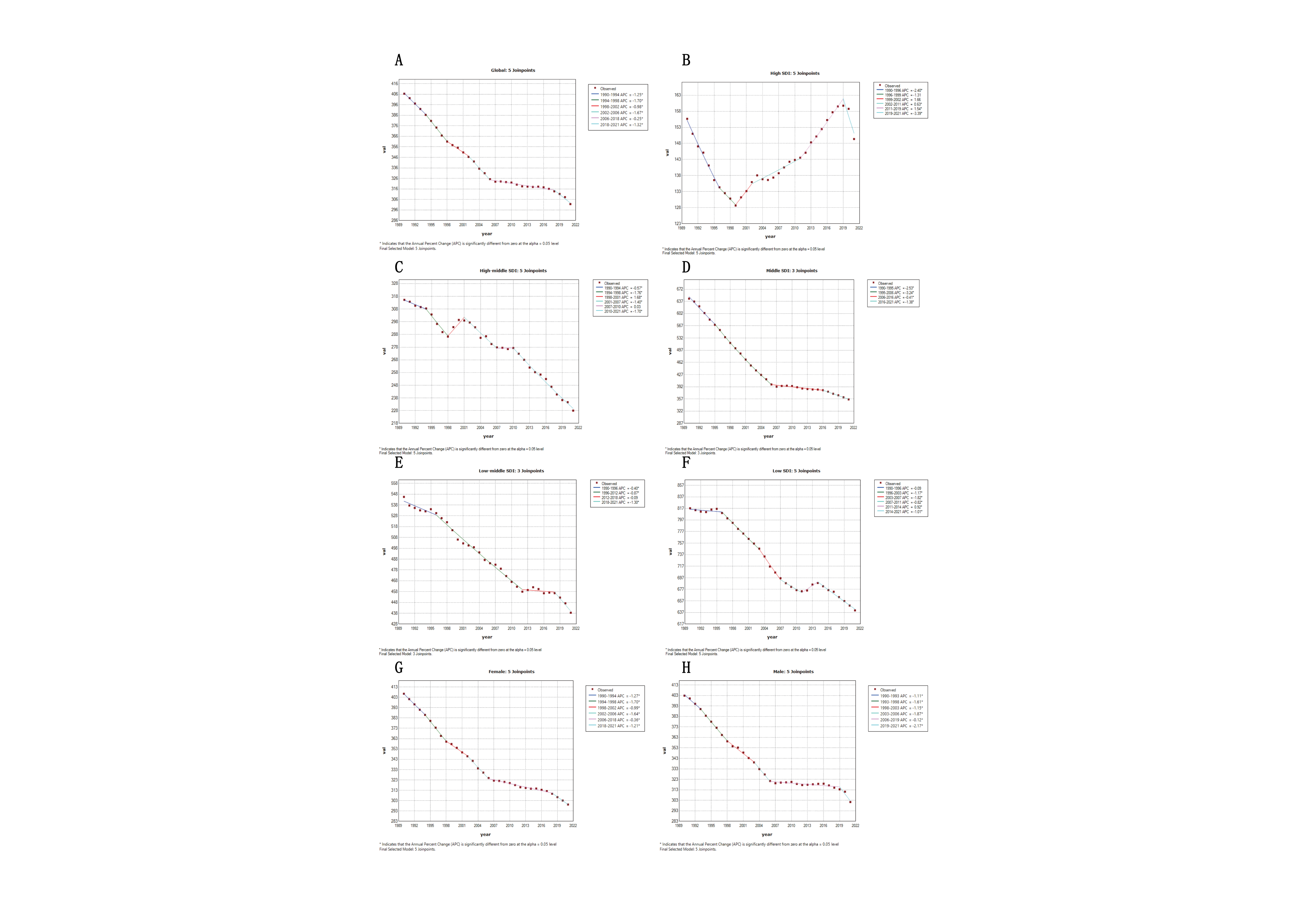
**

Figure S1: Joinpoint regression analysis of different socio-demographic index quintiles and different sex mortality rates for hypertensive heart disease globally, 1990-2021

**
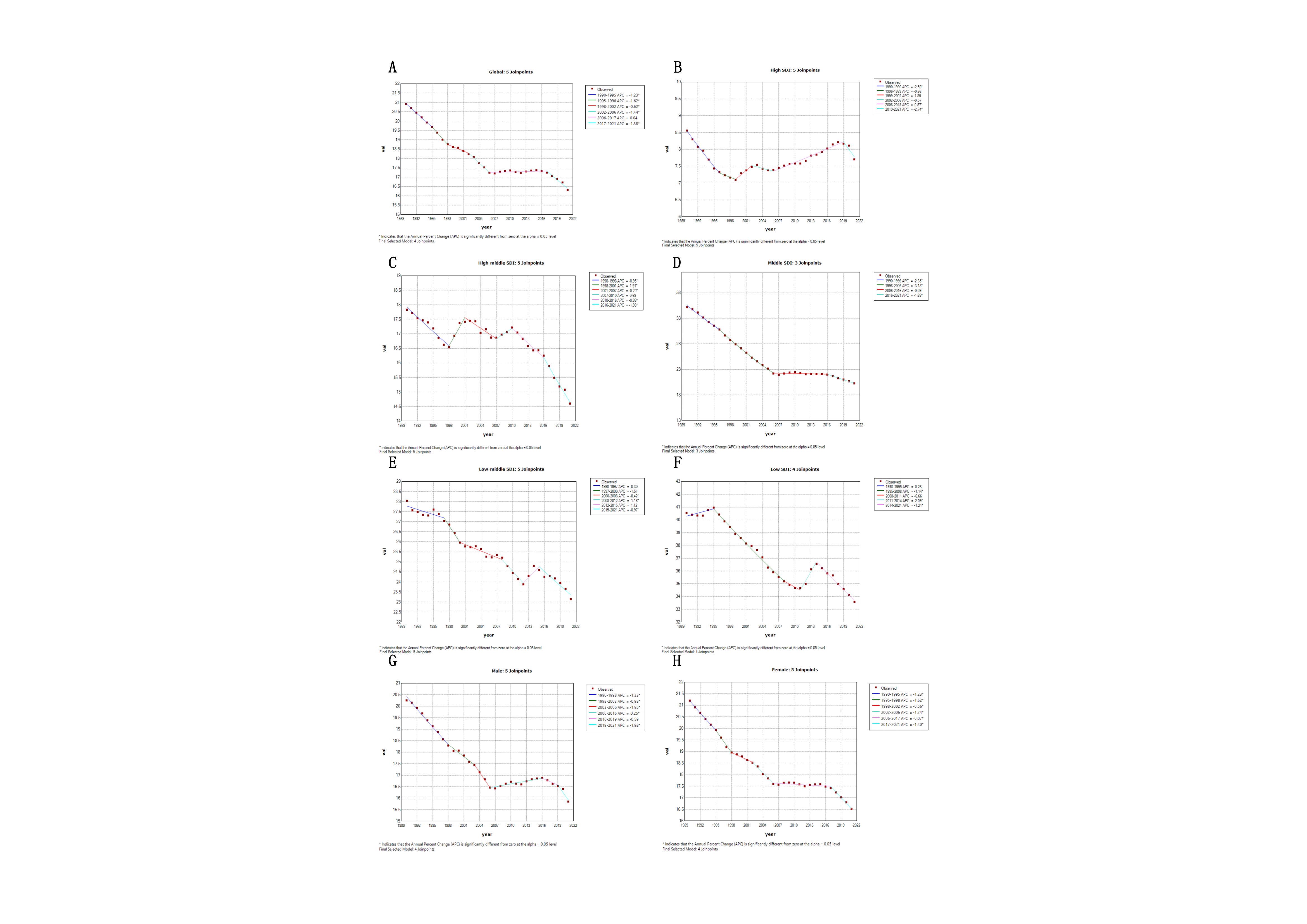
**

**Figure S2 Joinpoint regression analysis of disability-adjusted life years (DALYs) due to hypertensive heart disease globally by different socio-demographic index quintiles and sex, 1990-2021**

**Table S1: Prevalent cases of hypertensive heart disease in 1990 and 2021 and the percentage change in the age-standardized rates (ASRs) per 100,000 individuals by location**

|  | **Table S1: Prevalent cases of hypertensive heart disease in 1990 and 2021and the percentage change in the age-standardised rates (ASRs) per 100,000, by location** | | | | |
| --- | --- | --- | --- | --- | --- |
| Location | 1990_No_.95._UI. | 1990_ASRs_per_100000_.95._UI. | 2021_No_.95._UI. | 2021_ASRs_per_100000_.95._UI. | Percentage_change_in_the_ASRs_per_100000 |
| Global | 4626598 (3672198,5826592) | 125.4 (99,158) | 12505436 (9866066,15827877) | 148.3 (117.3,186.3) | 18.2 (10.6,26) |
| Andean Latin America | 29499 (23627,36701) | 150.2 (118.6,191.6) | 85962 (65683,110961) | 148.4 (112.9,192.7) | -1.2 (-13.4,10.3) |
| Australasia | 7290 (5725,9401) | 31.4 (24.6,40.1) | 30058 (24138,37342) | 52.4 (42,64.4) | 13.5 (-1.3,28.7) |
| Australia | 5975 (4642,7805) | 30.9 (24.1,40.2) | 27202 (21729,33484) | 56.2 (45.2,68.4) | -9.5 (-21.5,3.2) |
| New Zealand | 1315 (932,1803) | 33.7 (24.3,45.5) | 2856 (2065,3922) | 32.2 (23.3,43.7) | -0.4 (-13.9,15.4) |
| Caribbean | 36018 (28469,45489) | 141.5 (112.5,179.6) | 105243 (81142,136189) | 195.5 (151.1,253.3) | 67 (43,93.5) |
| Antigua and Barbuda | 159 (125,197) | 275.5 (216.9,339.8) | 306 (236,394) | 309.3 (241.2,398.4) | 81.5 (50.9,115.5) |
| Barbados | 569 (431,755) | 178.6 (140.1,232) | 1118 (865,1449) | 215.6 (168.9,276) | -4.2 (-16.5,10.8) |
| Belize | 174 (139,218) | 184.4 (146.2,234.6) | 699 (543,882) | 254.9 (195.1,330.8) | 38.1 (24.3,52.5) |
| Bermuda | 50 (38,64) | 82.4 (63.4,106.9) | 227 (171,302) | 161.1 (123.7,210.7) | 12.3 (-4.9,29.1) |
| Dominican Republic | 6675 (5359,8387) | 191.8 (150.6,243) | 18468 (14187,24517) | 188.5 (143.8,251.2) | 6.7 (-5.1,18.8) |
| Grenada | 137 (105,177) | 175.2 (136.3,222.6) | 226 (177,294) | 219.7 (172.4,284.5) | 20.7 (8.1,34.9) |
| Jamaica | 6207 (4916,7921) | 330.5 (263,421.7) | 10038 (7913,12821) | 317 (248.5,406.6) | 38.2 (19.5,60.2) |
| Puerto Rico | 5300 (4065,7001) | 144.6 (112.1,189.4) | 16851 (12755,22248) | 214.3 (164,278) | 95.6 (69.7,123.1) |
| Saint Kitts and Nevis | 57 (43,76) | 144 (110.8,191.7) | 121 (95,156) | 206.6 (160.1,263.9) | 154.4 (117.5,191.3) |
| Saint Lucia | 195 (151,253) | 224.7 (174.4,289.1) | 595 (461,766) | 255.1 (197.4,327.8) | 3.5 (-8.3,16.8) |
| Saint Vincent and the Grenadines | 180 (141,229) | 248.6 (197.5,317.3) | 425 (328,546) | 310.2 (241,395.5) | -1.7 (-17.7,14) |
| United States Virgin Islands | 129 (102,166) | 169.2 (132.2,219.3) | 352 (259,470) | 186.8 (141.8,241.7) | 25.4 (12,40.5) |
| Central Asia | 35124 (25708,46386) | 80.1 (57.8,107.6) | 69365 (48393,95011) | 95.6 (64.6,132.6) | 6.9 (-6.7,20.1) |
| Georgia | 4637 (3275,6460) | 78.3 (55.3,109) | 9299 (4653,13839) | 152.2 (79,223.7) | 0.5 (-12.5,16) |
| Mongolia | 522 (354,728) | 53.6 (35.8,75.3) | 962 (662,1362) | 51.9 (34.1,75.7) | -4.1 (-14.7,7.5) |
| Turkmenistan | 1058 (747,1446) | 62.6 (44.2,86.9) | 2841 (1978,3894) | 82 (55,116) | 48.2 (30.4,66.4) |
| Central Europe | 142712 (104117,188158) | 100.3 (73.7,131.3) | 339147 (247045,439437) | 146 (107.1,186.7) | 43.4 (25,66.6) |
| Bosnia and Herzegovina | 2670 (1972,3470) | 75.6 (54.6,99.4) | 5584 (3737,7682) | 87.9 (59.8,119.8) | 13.6 (-0.1,27.6) |
| Hungary | 13914 (9273,19952) | 96 (65.8,136.1) | 29679 (19624,41792) | 142.4 (97.5,198.4) | 24.8 (10.1,42) |
| Montenegro | 347 (255,468) | 59.4 (43.8,80.5) | 586 (417,834) | 60.3 (43.2,84) | 11.9 (-4,28.4) |
| North Macedonia | 2203 (1602,2929) | 134.7 (97.2,180.9) | 4481 (3149,6298) | 145.1 (104.8,200.2) | -2.7 (-15.2,11.9) |
| Romania | 30048 (19529,42165) | 114.7 (76.2,161) | 54656 (33411,78224) | 138.8 (87.9,194.6) | 10.4 (-5.1,26.7) |
| Central Latin America | 103128 (82807,129101) | 136.8 (108,171.6) | 284539 (222180,367456) | 119 (92.2,154) | 19.3 (3.8,33.9) |
| Central Sub-Saharan Africa | 38770 (28042,51326) | 221.6 (161.5,300.5) | 106346 (78305,139741) | 239.1 (176.9,320.4) | 33.4 (12.6,58.6) |
| Central African Republic | 1689 (1222,2260) | 198.6 (144.3,271.1) | 3440 (2496,4560) | 207.7 (152.6,279) | -1 (-16.6,16.8) |
| Democratic Republic of the Congo | 27118 (19459,35744) | 223.6 (159.3,300.7) | 71715 (52951,94800) | 240.2 (178.2,326.6) | 94.4 (33.7,138.4) |
| East Asia | 1550300 (1198536,1971217) | 216.7 (168.4,272) | 4066468 (3120125,5242374) | 193.2 (147.4,245.9) | -11.4 (-28,7.6) |
| Democratic People's Republic of Korea | 20489 (15857,26581) | 160.3 (121.5,211) | 54979 (42119,71899) | 181.4 (140,237.9) | 49.1 (27.5,77.3) |
| Taiwan (Province of China) | 26792 (20270,35311) | 196.6 (147.5,260.3) | 99331 (76001,129684) | 230.6 (178.4,301) | -3.1 (-20.8,18.3) |
| Eastern Europe | 77819 (57322,105778) | 28.7 (21.3,38.8) | 154963 (107122,212913) | 42.8 (29.8,58.3) | -4.1 (-18.4,14) |
| Republic of Moldova | 1314 (982,1751) | 30.5 (22.8,40.3) | 5861 (4077,8234) | 97.8 (68.4,137.9) | 31 (11.2,54.6) |
| Russian Federation | 48079 (34855,66026) | 27.6 (20.1,37.8) | 104820 (72024,145518) | 42.7 (29.5,59) | 19.3 (-0.8,40.5) |
| Ukraine | 22659 (16375,31114) | 32.1 (23.5,43.8) | 29953 (20897,41634) | 37.3 (25.9,51.2) | 45.6 (30.6,60.2) |
| Eastern Sub-Saharan Africa | 175066 (131637,224238) | 282.2 (216.1,371.2) | 425630 (324248,532350) | 291.8 (224,375.4) | 3.1 (-14.1,22.6) |
| United Republic of Tanzania | 27778 (20595,36322) | 297.9 (220.9,394.7) | 64360 (47219,83565) | 282.4 (209.2,370.7) | 16.3 (-1,37.3) |
| High-income Asia Pacific | 105137 (75815,138635) | 57.4 (41.7,75) | 297204 (219641,391369) | 57.6 (43.8,72.4) | 70.7 (49,93.5) |
| Brunei Darussalam | 63 (46,83) | 58 (35.8,82.7) | 176 (126,242) | 57 (36.2,80.5) | -20.6 (-37.2,-4.9) |
| Japan | 75062 (51652,100265) | 48.7 (34.4,64.7) | 195613 (140989,264657) | 44.9 (33.6,58.3) | 227.7 (174.1,285.5) |
| Republic of Korea | 28327 (21349,36057) | 107.7 (78.2,142.2) | 91856 (71867,116578) | 103 (81.1,130) | 48.3 (25.5,72.7) |
| High-income North America | 316482 (237496,411000) | 91.8 (70.3,118.4) | 949322 (738427,1167271) | 150.7 (119.5,181.9) | 1.5 (-13.7,20.6) |
| Canada | 12490 (9811,15645) | 38.7 (30.7,48.4) | 63810 (50259,80384) | 94.9 (75.7,117.7) | 7.8 (-6.9,26.4) |
| Greenland | 24 (19,31) | 63.6 (47.2,80.7) | 52 (39,67) | 84.3 (63.6,108.3) | 53.8 (33.3,78.7) |
| United States of America | 303960 (226202,396179) | 97.3 (74.3,126.1) | 885445 (687332,1097741) | 157.5 (124.1,191.3) | 21 (0.7,43.1) |
| North Africa and Middle East | 365066 (297761,449144) | 239.5 (192.8,299.2) | 1027631 (814739,1265656) | 243.3 (192.1,303.6) | 10.9 (-5.6,29.2) |
| Palestine | 1948 (1579,2382) | 233 (185.2,292.7) | 5551 (4410,6865) | 234.1 (184,299.1) | 57.6 (35.4,85.3) |
| Syrian Arab Republic | 9096 (7232,11237) | 188.8 (145,240.9) | 20771 (15724,27068) | 178.9 (135.2,235.9) | 77.2 (50.1,114.2) |
| United Arab Emirates | 966 (771,1189) | 193.6 (150.9,245.9) | 12043 (9193,16090) | 247.1 (189.3,324.3) | -13 (-21.1,-5) |
| Oceania | 2767 (2167,3496) | 115.7 (90.2,148) | 6738 (5279,8527) | 106.9 (83.7,136.8) | -29.7 (-39.3,-17.4) |
| American Samoa | 21 (16,27) | 109.9 (85.3,141.3) | 39 (29,52) | 91.6 (67.1,123.8) | 12.3 (-1,27) |
| Cook Islands | 38 (30,47) | 332.9 (259.4,426.9) | 80 (63,103) | 317.5 (252.2,402.5) | -9.4 (-22.1,6.7) |
| Guam | 139 (109,172) | 212.1 (163,272) | 251 (187,333) | 119.7 (89.3,160.4) | -5.8 (-20.2,11.5) |
| Northern Mariana Islands | 10 (8,13) | 72.1 (55.1,93.9) | 27 (21,37) | 63.2 (48.2,84.9) | -0.2 (-12.4,13.5) |
| Solomon Islands | 98 (77,125) | 90 (69.7,117.4) | 255 (198,327) | 86.8 (66.6,112.9) | -9.4 (-19,-1) |
| Tokelau | 2 (1,2) | 138.2 (108.4,179.2) | 2 (1,2) | 120.2 (91.8,160.2) | -10.8 (-24.1,2.1) |
| Tuvalu | 7 (6,9) | 133.4 (103,169.8) | 11 (9,15) | 118.8 (91.1,152.1) | 50.9 (31.8,73.9) |
| South Asia | 470748 (374964,589000) | 104.1 (82.7,129.6) | 1432788 (1119541,1828057) | 111.1 (86.6,142.7) | -7.7 (-20.7,6.5) |
| Southeast Asia | 370474 (301792,452815) | 168.1 (135.9,207.4) | 960247 (767865,1203976) | 163.3 (131.2,205.2) | 7.9 (-0.9,19) |
| Lao People's Democratic Republic | 2827 (2176,3630) | 164.3 (129.2,211.5) | 6753 (5273,8611) | 170.1 (130,221.1) | 10.2 (-0.7,22.6) |
| Malaysia | 5459 (4289,6897) | 64 (49.2,83.6) | 15482 (11993,20002) | 58.9 (45.2,76.7) | 4.6 (-5.8,16.3) |
| Southern Latin America | 39029 (28709,52164) | 88.1 (64.9,117.8) | 98819 (70049,130976) | 110.1 (78.3,145.8) | 8.6 (-1.4,21.4) |
| Southern Sub-Saharan Africa | 48002 (35809,61790) | 196.8 (147.1,256.1) | 108006 (80828,139595) | 210.6 (157.4,272.2) | 7.5 (-3.5,21.1) |
| Tropical Latin America | 122755 (95999,153967) | 149.9 (118.5,187.4) | 411264 (317930,527720) | 166 (129.1,213.6) | 13.7 (2.4,27.2) |
| Western Europe | 394304 (293760,522422) | 65.8 (49.5,86.4) | 1094895 (830592,1423768) | 100.8 (78,128.1) | 5.1 (-4.6,15.3) |
| Ireland | 584 (411,813) | 14.7 (10.5,20.1) | 2426 (1684,3302) | 29.5 (21,39.6) | -10.9 (-19.2,-3.3) |
| Western Sub-Saharan Africa | 196108 (151200,249117) | 257.4 (201.8,331.6) | 450801 (347733,569919) | 266.3 (206.7,342.2) | -11.8 (-20.3,-4.3) |
| Burkina Faso | 9728 (7143,12831) | 266.9 (199,350.2) | 23000 (17386,30261) | 281.9 (213.1,377.8) | 13.2 (-1.8,29) |
| Democratic Socialist Republic of Sri Lanka | 16412 (12864,20932) | 172.6 (133.1,221.9) | 42928 (33236,55632) | 164.8 (128.8,213) | 17.3 (4.8,32.1) |
| Republic of Armenia | 2250 (1634,3008) | 95.7 (68.2,129.7) | 5546 (4222,7105) | 127.8 (97.5,163.3) | 49.3 (32.6,67.2) |
| Republic of the Philippines | 50220 (40648,61454) | 191.7 (154.2,236.4) | 144189 (115437,178488) | 200 (157.7,249.9) | -61.2 (-68,-53.2) |
| Republic of the Union of Myanmar | 31942 (25145,40959) | 162.8 (127.2,207.4) | 71192 (55944,91130) | 162.7 (126.6,208.3) | 222.1 (166.7,282.3) |
| Republic of Maldives | 81 (64,103) | 114.4 (88.1,147) | 403 (319,507) | 130.8 (99.7,170.1) | 312.8 (227.3,417.6) |
| Republic of Vanuatu | 49 (38,63) | 94.9 (73.2,121.9) | 139 (108,178) | 94.4 (72.4,123.1) | 169.3 (117.2,240.6) |
| Kingdom of Tonga | 39 (31,50) | 78.6 (61.3,99.9) | 58 (44,76) | 74.6 (56.8,98) | 221.2 (162.9,297.5) |
| Republic of Indonesia | 153918 (124479,188583) | 189.5 (151.4,232.2) | 380544 (298134,479534) | 190.4 (148.5,240.8) | 54.3 (34.8,73.1) |
| Kingdom of Cambodia | 7236 (5683,9247) | 193.7 (147.6,254.4) | 20955 (16200,26879) | 196.7 (153.5,254.2) | 16.2 (-0.9,35.9) |
| Federated States of Micronesia | 58 (44,74) | 132.2 (101.8,169.8) | 76 (59,97) | 128.2 (98.2,166.3) | 3.4 (-0.9,8.6) |
| Independent State of Samoa | 113 (88,144) | 149.6 (116.2,193) | 173 (136,226) | 132.6 (102.8,175.2) | 3.9 (-6,16.2) |
| Independent State of Papua New Guinea | 1607 (1254,2043) | 110.2 (86,141.6) | 4474 (3509,5595) | 105.1 (82.6,134.7) | 2.2 (-7.4,12.4) |
| Republic of the Marshall Islands | 18 (14,24) | 130.1 (101.4,168.8) | 34 (26,44) | 121.3 (93.9,156.5) | 0.5 (-8.9,10.1) |
| Republic of Fiji | 356 (280,449) | 121.3 (93.4,157.4) | 756 (580,979) | 117.4 (91.3,154.7) | 5.1 (-5,16) |
| Republic of Kiribati | 24 (18,30) | 77.3 (59.2,99.9) | 43 (33,56) | 74.6 (56.2,97.3) | 11.5 (5.6,18.4) |
| People's Republic of China | 1503019 (1161178,1916382) | 218.2 (169.3,274.8) | 3912158 (2989417,5056002) | 192.5 (146.7,245) | 3 (-1,7.6) |
| Republic of Tajikistan | 4380 (3244,5778) | 170.3 (124,229.1) | 8278 (6037,10852) | 163.2 (112,221) | -1.6 (-11.2,8.8) |
| Kyrgyz Republic | 1729 (1267,2331) | 62.2 (44.4,84.2) | 3881 (2703,5169) | 92.8 (62.8,127.4) | -1 (-10.5,10.3) |
| Republic of Kazakhstan | 7900 (5778,10651) | 66.2 (47.6,90) | 9401 (6295,13336) | 58.7 (39,84) | 0 (-9.5,10.4) |
| Socialist Republic of Viet Nam | 80435 (62618,103863) | 214.8 (167.6,277.8) | 182456 (142953,233597) | 205.7 (159.8,267.4) | 5.6 (-4.8,17.4) |
| Czech Republic | 4326 (3138,5982) | 32 (23.4,43.5) | 23607 (16047,33674) | 104.7 (72.8,143.7) | -4.7 (-13.5,5.4) |
| Republic of Bulgaria | 16441 (11773,22258) | 140.1 (103.3,184.4) | 35325 (25085,48102) | 239 (173.4,315.9) | -1.3 (-12.3,9.8) |
| Republic of Uzbekistan | 8266 (6159,10768) | 73.6 (53.2,98.1) | 20669 (14514,28162) | 87.8 (59.8,125.3) | 6.1 (-3.3,17.6) |
| Democratic Republic of Timor-Leste | 423 (338,526) | 191.6 (148.3,247.2) | 1433 (1124,1876) | 178.2 (139.9,230.1) | -5.2 (-14.6,4.2) |
| Republic of Croatia | 5105 (3601,6941) | 91.9 (63.7,125.1) | 6749 (4344,9160) | 73 (49.3,96.3) | 3 (-6.7,14.2) |
| Republic of Azerbaijan | 4381 (3176,5864) | 99.3 (71.1,133.9) | 8488 (5942,11489) | 98.3 (66.7,134.8) | 0.2 (-10.5,14) |
| Kingdom of Thailand | 19727 (15690,25064) | 63.5 (49.5,81.2) | 88634 (68950,116757) | 82.7 (64.3,108.5) | -1.8 (-14.7,17.1) |
| Republic of Albania | 1532 (1124,2032) | 82 (59,110.8) | 3718 (2632,5124) | 84.6 (61.1,114) | -7.8 (-19.1,4.7) |
| Republic of Lithuania | 987 (719,1346) | 22.2 (16.3,30) | 3627 (2409,5263) | 59.8 (39.5,85.9) | -4.4 (-19.6,14.8) |
| Republic of Latvia | 612 (438,847) | 17.3 (12.4,23.6) | 3151 (2073,4603) | 71.2 (46.9,103.9) | 46.1 (27.3,74.7) |
| Republic of Estonia | 1312 (968,1747) | 65.4 (48.9,86.3) | 6158 (4003,8647) | 210.6 (142.8,290.9) | 64.2 (42.9,87.3) |
| Republic of Cyprus | 405 (275,568) | 56.9 (39.2,77.8) | 1381 (978,1934) | 67.4 (48.9,92.3) | 145.5 (112.8,183.2) |
| Republic of Austria | 10735 (8064,13167) | 86.3 (66.2,105.1) | 21767 (16092,28138) | 103.8 (78.7,132.5) | 32.5 (16.4,51.1) |
| Kingdom of Belgium | 3059 (2182,4227) | 19.4 (14.1,26.3) | 5979 (4434,8252) | 23 (17.6,30.5) | 61.9 (39.8,86.6) |
| Republic of Belarus | 2856 (2044,3852) | 22.4 (16.1,30.3) | 1393 (954,1954) | 8.7 (6,12) | 1.6 (-6.4,10.8) |
| Kingdom of Denmark | 1147 (796,1578) | 13.5 (9.7,18.2) | 3479 (2424,4915) | 27.5 (19.6,37.3) | -3.2 (-14.1,9.5) |
| Principality of Andorra | 43 (32,57) | 82.9 (62.4,110) | 139 (105,183) | 85.3 (64.9,112) | 6 (-5,19.3) |
| Republic of Slovenia | 2026 (1352,2820) | 83.7 (56.1,115.5) | 7186 (4739,10174) | 148.3 (100.8,206.7) | 29.1 (14.3,46.3) |
| Slovak Republic | 3613 (2728,4757) | 61.5 (46.7,80.7) | 9341 (6639,13164) | 96.9 (70.2,133.5) | -6.1 (-18.6,8.1) |
| Republic of Serbia | 7857 (5308,10891) | 80.6 (53.3,112.2) | 15331 (9429,21626) | 89.4 (56.6,123.2) | 10.7 (2.9,17.9) |
| Federal Republic of Germany | 103336 (76205,141423) | 79.2 (59,106.5) | 252729 (177135,345631) | 111.5 (79.9,150.4) | -5.7 (-17.1,6.7) |
| Republic of Finland | 2615 (1749,3576) | 36.6 (25.4,49.4) | 15435 (10231,22314) | 107.6 (73.8,150.4) | 11.8 (-2,25.8) |
| Republic of Singapore | 1686 (1272,2157) | 78.4 (54.4,104.3) | 9560 (7224,12371) | 114.5 (87.3,147.9) | -6.6 (-17.8,5.3) |
| French Republic | 106021 (79900,141753) | 118.9 (90.6,158) | 247024 (179959,334930) | 145.1 (109,193.9) | -4.6 (-16,7.5) |
| Argentine Republic | 26995 (19723,36504) | 87.3 (64.5,117.4) | 57338 (38246,77055) | 99.3 (67.4,132.6) | -1.7 (-14,11) |
| Republic of Poland | 50348 (38535,66385) | 119.1 (91.9,155.4) | 137968 (105620,175866) | 183.2 (141.1,230.1) | 0.7 (-11.4,15.3) |
| United Kingdom of Great Britain and Northern Ireland | 18302 (12529,24992) | 20.1 (14.5,27) | 46948 (33456,63289) | 35.3 (25.6,46.5) | 31.6 (15.1,51.6) |
| Swiss Confederation | 5080 (3299,7198) | 45.8 (31,63.7) | 17438 (12462,23781) | 86.2 (63.3,115.2) | 0.5 (-10.4,12.1) |
| Kingdom of Sweden | 7222 (4887,10667) | 43.9 (30.5,63.5) | 30386 (21432,42602) | 118.7 (84.2,161.8) | 21.4 (6.2,38.2) |
| Republic of Guyana | 864 (679,1101) | 247.7 (190.1,323.5) | 1492 (1160,1938) | 264.8 (206.6,341.5) | 3.3 (-10.4,15.6) |
| Kingdom of Spain | 24331 (17680,33504) | 44.5 (32.2,60.8) | 98813 (77720,119433) | 86.7 (70.2,103.9) | 1.9 (-11.3,15.7) |
| Commonwealth of Dominica | 152 (118,200) | 251.5 (197.4,328.1) | 206 (160,270) | 260.3 (202.9,339.9) | -5.2 (-18.9,7.8) |
| Kingdom of Norway | 1185 (651,1749) | 16.2 (9.4,23.3) | 3018 (1998,4329) | 26.8 (18.1,37.5) | 7.2 (-4.4,19.4) |
| Portuguese Republic | 7159 (5393,9602) | 54.3 (41.2,71.5) | 21202 (15195,28667) | 74.3 (55.1,98.6) | -0.6 (-11.9,12.2) |
| Kingdom of the Netherlands | 3824 (2919,4991) | 18.4 (14.3,23.8) | 14549 (10624,19619) | 38.3 (27.9,51) | 27.6 (13.7,42.1) |
| Republic of Cuba | 7138 (5544,9100) | 68.5 (53.4,87.9) | 34615 (26158,44563) | 174.4 (134.5,224.5) | 0.9 (-9.4,14.2) |
| Commonwealth of the Bahamas | 455 (361,573) | 309.3 (241.7,391.1) | 1208 (943,1550) | 329.9 (257.4,428.2) | -7.6 (-17.2,2.6) |
| Republic of Malta | 130 (97,174) | 32.9 (24.4,43.5) | 650 (470,891) | 61.5 (45.4,82.2) | -16.6 (-30.4,-1.4) |
| Grand Duchy of Luxembourg | 198 (128,284) | 37 (24.5,51.7) | 710 (507,966) | 63 (45.5,84.4) | -4.6 (-14.8,6.7) |
| Republic of Guatemala | 1995 (1569,2615) | 67.9 (52.1,90.4) | 6590 (4962,8819) | 64 (48,85.5) | -3.2 (-15.5,12.3) |
| Republic of Honduras | 3734 (2958,4648) | 195.4 (153,247.5) | 11389 (8660,14805) | 195 (147.2,254.7) | -43.6 (-53,-33.5) |
| Republic of El Salvador | 2430 (1871,3136) | 85 (64.5,110.3) | 5025 (3803,6684) | 77 (58.1,102.9) | -3.4 (-15.6,10.9) |
| Republic of Costa Rica | 2360 (1874,3029) | 139.3 (108.9,179.8) | 8556 (6503,11105) | 156.5 (117.8,204.2) | -6.7 (-18,6.3) |
| Republic of Italy | 91410 (59107,124524) | 100.9 (67.1,136.5) | 293224 (219071,374596) | 169.9 (129.6,213.9) | -3 (-14.6,9.3) |
| Republic of Colombia | 29780 (23639,37936) | 187.9 (145.2,243.1) | 73340 (55690,97055) | 132.1 (100.3,175.3) | -12.8 (-26.6,3.7) |
| Hellenic Republic | 5928 (4238,7837) | 40.2 (29.3,52.9) | 11594 (8060,15784) | 41.1 (29.8,53.5) | -7.7 (-19.7,6) |
| State of Kuwait | 1904 (1559,2321) | 306.4 (243.7,387.8) | 8228 (6490,10337) | 286.2 (222.3,367.5) | -12.3 (-25.6,1.1) |
| State of Israel | 1163 (793,1594) | 24.6 (17,33.3) | 4719 (3370,6384) | 35.9 (26.3,48.5) | -8.6 (-19.9,6) |
| Hashemite Kingdom of Jordan | 3883 (3174,4778) | 305.7 (244.4,390.3) | 23947 (19043,29713) | 341.9 (268.8,427.8) | -4.6 (-15.8,7.8) |
| Republic of Iraq | 16235 (13093,20154) | 206.6 (162.6,262.4) | 42680 (33586,53788) | 194.9 (151.4,253.3) | -11.4 (-22.5,0.6) |
| Islamic Republic of Iran | 52354 (42370,64334) | 226.1 (183.3,279) | 185438 (147645,229529) | 250.2 (196.9,312.9) | -3.5 (-17.5,14.1) |
| Republic of Iceland | 36 (24,50) | 12.3 (8.1,16.9) | 148 (103,204) | 24.2 (17.4,33.2) | -13 (-25.6,0.8) |
| Kingdom of Bahrain | 237 (189,298) | 139.9 (108.7,180.5) | 1536 (1197,1963) | 180.7 (139.3,231.8) | -5.1 (-17,8.6) |
| Arab Republic of Egypt | 57917 (46297,71094) | 241.4 (190.9,306.9) | 125969 (97722,158183) | 226.7 (176.4,290.9) | -10.9 (-22.4,3.4) |
| People's Democratic Republic of Algeria | 31522 (24802,39505) | 272.2 (216.5,339) | 97354 (76502,122619) | 288.6 (224.5,366.6) | -0.5 (-12.4,14.8) |
| Republic of Paraguay | 3650 (2911,4552) | 170.7 (134.2,217.7) | 10733 (8380,13754) | 193.6 (149,249.8) | 6.7 (-1.1,13.8) |
| Federative Republic of Brazil | 119105 (93006,149374) | 149.3 (117.9,187.4) | 400532 (309339,513910) | 165.3 (128.3,212.7) | 5.7 (-7.3,19.4) |
| Eastern Republic of Uruguay | 2342 (1672,3249) | 60.1 (43.7,81.8) | 5329 (3366,7310) | 87.9 (57.7,119.6) | 0.9 (-10.4,13.9) |
| Bolivarian Republic of Venezuela | 16888 (13437,21551) | 187.7 (144.4,243.4) | 48741 (37347,64086) | 173.2 (132.1,230.2) | 8.5 (-0.3,16.5) |
| Republic of Panama | 1283 (992,1678) | 90.1 (68.8,118.2) | 6060 (4579,7843) | 135.9 (102.7,176.9) | 20.4 (4.9,36.7) |
| People's Republic of Bangladesh | 53125 (42591,67255) | 131.9 (103.1,168) | 171255 (133385,219308) | 139.5 (108.7,178.4) | 4.5 (-5.6,13.8) |
| Islamic Republic of Afghanistan | 15224 (11956,19153) | 260.8 (204.7,333.9) | 23769 (18964,29386) | 252.6 (194.6,323.8) | -2.8 (-9.5,3.6) |
| Kingdom of Saudi Arabia | 15638 (12867,19303) | 279.9 (222.2,355.7) | 50759 (40865,63580) | 289.1 (222.8,372.7) | 1.5 (-8.2,13.8) |
| Kingdom of Morocco | 31466 (25134,39184) | 230.6 (181,292.5) | 74291 (58209,94416) | 232.2 (180,299.9) | 0.5 (-7.7,7.3) |
| Republic of Yemen | 10886 (8842,13456) | 251.4 (197.6,319.4) | 33875 (27386,41746) | 253.8 (200.1,325.2) | 3.5 (-8.8,17.7) |
| State of Qatar | 198 (157,252) | 143.8 (113.2,184.3) | 2081 (1580,2739) | 174.5 (133.7,227.2) | -7.9 (-20.4,6.3) |
| Republic of Tunisia | 12314 (9935,15535) | 264.2 (210.6,335.1) | 35846 (27616,45233) | 283.4 (220,357.5) | 14.3 (-1.3,31.4) |
| Republic of Turkey | 72979 (58126,91111) | 235.9 (185,297.1) | 208006 (160011,267069) | 234.4 (181.5,301.2) | 23.8 (10,39.2) |
| Republic of Djibouti | 329 (245,426) | 299.1 (221.6,392.1) | 1596 (1167,2060) | 300.7 (218.9,395.2) | -0.1 (-11.3,12.9) |
| Republic of Chile | 9690 (7274,12757) | 102.3 (74.9,135.1) | 36147 (26855,47810) | 139.5 (104.3,184) | 4.3 (-2.3,10.3) |
| Union of the Comoros | 496 (364,640) | 297.6 (219.3,390.8) | 1332 (988,1718) | 304.1 (225.6,398.9) | 0.3 (-10,12.7) |
| Republic of Burundi | 5659 (4137,7437) | 278.1 (203.5,373.1) | 11909 (8826,15355) | 288.9 (211.4,378.8) | -4.6 (-15.7,8.1) |
| Republic of Equatorial Guinea | 330 (238,445) | 214.2 (154.5,293.2) | 1057 (785,1383) | 243.6 (177.4,325.9) | 30.2 (15.2,48.6) |
| Republic of Haiti | 4247 (3289,5438) | 159.7 (122.2,206) | 9778 (7626,12262) | 160.6 (122.3,209) | -7 (-17,5.8) |
| Republic of Peru | 14667 (11722,18441) | 127.4 (99.9,162.9) | 42384 (31863,55132) | 126.8 (94.7,166.6) | -4.2 (-15.2,10.1) |
| Republic of the Congo | 1847 (1333,2428) | 216.3 (159.1,291.3) | 5136 (3795,6826) | 234.8 (174.2,315) | 25 (10.3,41.6) |
| Republic of Angola | 6556 (4835,8659) | 217.9 (157.6,293.3) | 22744 (16487,29818) | 240.1 (175.5,316.4) | 46.3 (24.7,70.1) |
| Republic of Suriname | 449 (358,567) | 189.5 (149.4,241.3) | 1259 (961,1606) | 212.1 (162.9,271.4) | 13.7 (-2.9,31.1) |
| Islamic Republic of Pakistan | 58297 (45927,73617) | 119.7 (94,149.7) | 125006 (97730,160203) | 125.1 (94.9,159.1) | 36.4 (18.7,56.5) |
| Federal Democratic Republic of Nepal | 7343 (5793,9136) | 101.2 (78.2,129.4) | 24573 (19181,31598) | 121.8 (95,156.7) | 7 (2.6,12.6) |
| Plurinational State of Bolivia | 3830 (3028,4840) | 132 (102.9,170.9) | 12584 (9667,16457) | 149.9 (114,196.3) | 3.3 (-6.6,13.9) |
| Republic of Ecuador | 11002 (8835,13730) | 213.2 (168,270.9) | 30995 (23949,40975) | 193 (149.3,254.8) | 2.4 (-7.5,14.2) |
| Kingdom of Lesotho | 1512 (1086,2081) | 200 (143.7,278.5) | 1867 (1297,2508) | 204.8 (145.6,273.5) | 2.4 (-8.7,13.6) |
| Republic of Trinidad and Tobago | 1663 (1322,2136) | 203.1 (160.1,259.1) | 3698 (2839,4796) | 197.7 (152.8,253.6) | 10.6 (0.7,23.8) |
| Republic of Uganda | 16760 (12519,21315) | 298.1 (221.2,390.4) | 41182 (31316,51826) | 316.3 (241.4,414.8) | 8.6 (3.7,14.6) |
| Republic of Madagascar | 12973 (9515,16598) | 299.4 (221.6,394.7) | 26909 (19685,35263) | 294.6 (218.6,387.3) | -2.7 (-13.4,9.2) |
| Republic of Mozambique | 12024 (8653,16261) | 239.3 (176.3,324.2) | 22833 (16501,29847) | 239.2 (168.1,317.9) | 10.7 (1.8,18.5) |
| Republic of Botswana | 933 (647,1273) | 203.1 (140.8,277.9) | 2631 (1891,3487) | 209.8 (149.3,283.7) | 10.7 (1.6,18.8) |
| Republic of Zambia | 6923 (5101,9025) | 292.7 (215.7,391.8) | 17823 (13059,23217) | 301.6 (226.2,391.6) | 13.4 (-1,28.4) |
| Republic of Mauritius | 1097 (857,1431) | 169.8 (131.7,225.7) | 3643 (2816,4632) | 210.2 (162.2,267.8) | 53.3 (34.7,72.3) |
| Republic of Seychelles | 161 (126,205) | 282 (221.3,359.9) | 296 (235,376) | 282.9 (221.6,364) | 2.9 (-12.3,19.9) |
| Republic of Liberia | 2772 (2056,3647) | 269.7 (201.8,356.5) | 5056 (3854,6511) | 275.2 (206.3,360.4) | 20.3 (-1.2,44.7) |
| Republic of Guinea-Bissau | 785 (581,1043) | 244.6 (181.8,326) | 1471 (1115,1930) | 250.6 (184.3,332.5) | 18.3 (-8.9,54.5) |
| Sultanate of Oman | 1376 (1160,1660) | 194.5 (160,242.7) | 5509 (4319,7028) | 256 (199.5,332.8) | 18.5 (1.2,43.8) |
| Republic of Guinea | 8005 (5976,10655) | 269.4 (202.8,361.9) | 13776 (10349,17796) | 273.2 (202.6,357.9) | 103.1 (64.7,147.3) |
| State of Libya | 4717 (3810,5737) | 257.4 (205,323.4) | 12464 (9808,15544) | 252.9 (195.3,319.8) | 193.9 (121.5,279.9) |
| Republic of Nicaragua | 2315 (1847,2968) | 164.4 (128.6,213.3) | 6686 (5157,8659) | 146.7 (112.8,192.7) | 22.1 (2,46.2) |
| Republic of the Gambia | 777 (567,995) | 256.7 (188.6,338.4) | 2325 (1730,3037) | 263.5 (194,349.7) | 40.7 (20.9,64.2) |
| Republic of Ghana | 14049 (10994,17964) | 273.9 (209.5,364.8) | 39122 (29121,50718) | 278 (208.8,367.6) | 2.2 (-18.4,26) |
| Lebanese Republic | 3765 (3008,4802) | 193.7 (151.5,249.5) | 11626 (8948,14947) | 184.8 (142.4,236.4) | 97.3 (65.2,140.6) |
| United Mexican States | 42341 (33212,53402) | 110.5 (85.6,139.9) | 118152 (87685,154937) | 100 (74.1,131.1) | 101.3 (62.5,147.6) |
| Republic of Cameroon | 10478 (7964,13357) | 281.8 (208.6,369.1) | 29984 (22631,39372) | 277.7 (208.9,368.4) | 46.1 (21.7,76.2) |
| Republic of C么te d'Ivoire | 8227 (6224,10706) | 260.2 (195.2,348.7) | 25205 (18928,32311) | 268.1 (202.2,354.8) | 68.4 (44.5,98.8) |
| Republic of Chad | 6466 (4758,8521) | 251.3 (186.1,333.4) | 13319 (10071,17001) | 267.4 (196.4,350.1) | 70 (39.1,108.8) |
| Republic of Benin | 4950 (3714,6545) | 268.8 (201.6,355.7) | 12735 (9652,16403) | 276.7 (203.8,366.9) | 87.1 (56.2,126.6) |
| Gabonese Republic | 1229 (889,1651) | 242.4 (178.8,328.9) | 2254 (1656,3009) | 254.8 (185.9,340.4) | 59.5 (34.9,83.6) |
| Republic of Zimbabwe | 6987 (5093,9324) | 201.5 (147.8,275.6) | 11038 (7703,14486) | 196.1 (138.7,265.6) | 107.7 (62.3,152.7) |
| Togolese Republic | 2682 (2033,3513) | 257.1 (192.8,340.3) | 8481 (6405,10940) | 268 (203,353.8) | 65.5 (39.3,104) |
| Republic of Sierra Leone | 4632 (3464,6141) | 247.2 (186.3,328.3) | 8889 (6577,11664) | 264.9 (194.6,351.9) | 36.9 (15.9,59.2) |
| Kingdom of Eswatini | 492 (356,656) | 205.5 (147.3,281.4) | 973 (691,1295) | 210.4 (149.5,284.7) | 32.8 (15.3,52.3) |
| Democratic Republic of Sao Tome and Principe | 166 (124,214) | 277.2 (207.9,357.4) | 285 (211,363) | 290.1 (214.4,376.9) | 95.1 (52.1,154.4) |
| Federal Republic of Nigeria | 97321 (76006,122714) | 250.1 (195.4,313.6) | 201897 (159272,249677) | 257.2 (202,324) | 170.4 (115.7,234.9) |
| Republic of Palau | 5 (4,7) | 57.4 (44.1,73.7) | 10 (8,13) | 52.5 (40,69.9) | 88.2 (58.3,133.6) |
| Republic of Niue | 3 (2,4) | 129.2 (100.6,165.3) | 2 (2,3) | 119.2 (92,156.2) | 75.4 (54.9,95.2) |
| Republic of Malawi | 8564 (6247,11210) | 268.6 (199.9,356.7) | 17162 (12596,22120) | 266 (197.5,351.9) | 3.4 (-0.8,7.9) |
| Kingdom of Bhutan | 244 (197,303) | 131.7 (103.7,166.8) | 755 (598,950) | 132.9 (104.2,168.2) | 2.9 (-6.1,14.6) |
| Republic of India | 351739 (276997,440739) | 97.9 (77,122.4) | 1111198 (849201,1438465) | 106.2 (82,137.4) | 5.6 (-4.3,16.3) |
| Federal Republic of Somalia | 4824 (3670,6249) | 258.3 (192,341.7) | 12368 (9261,16240) | 246.3 (181,329.9) | 3.1 (-6.9,13.3) |
| Republic of Nauru | 4 (3,5) | 106.9 (80.8,138.9) | 5 (3,6) | 93.2 (71.7,125) | -1.5 (-10.5,9.7) |
| Republic of Rwanda | 6499 (4794,8479) | 280.7 (207.9,373.7) | 16054 (11677,20918) | 296.5 (218.9,389.7) | 6.4 (-2.8,18.5) |
| Federal Democratic Republic of Ethiopia | 42281 (32269,53799) | 270.8 (211.6,346.4) | 117032 (92515,145979) | 301.9 (241.2,382.3) | 3 (-6,14.5) |
| State of Eritrea | 2252 (1628,2953) | 274.2 (204,366.8) | 6491 (4804,8400) | 288.1 (211.8,380.9) | 2.6 (-7.5,14.4) |
| Republic of Kenya | 20620 (16034,25924) | 290.3 (228.1,368.4) | 58551 (46258,73320) | 299 (234.6,374.6) | 1.5 (-9.1,13.8) |
| Republic of South Sudan | 6959 (5160,9138) | 304.1 (223.1,406.1) | 9655 (7232,12431) | 300 (220.7,394.2) | 1.4 (-8.4,11.9) |
| Republic of Cabo Verde | 701 (523,918) | 297 (222.1,385.6) | 1292 (974,1679) | 306.1 (228.2,401.3) | 2.4 (-7,13.1) |
| Republic of South Africa | 36968 (27586,47246) | 195.6 (145.8,253.3) | 88802 (66718,113987) | 212.4 (159.1,273.6) | 2.1 (-6.7,11.9) |
| Republic of Senegal | 7263 (5373,9409) | 253.2 (187.2,336.2) | 18406 (13867,24074) | 263.4 (198.6,348) | 5.9 (-3.7,16.9) |
| Republic of the Niger | 6021 (4531,7795) | 264.5 (198.1,351.3) | 18675 (13793,24197) | 269.9 (203.1,357.2) | 8.4 (-2.2,19.6) |
| Republic of Namibia | 1110 (783,1504) | 205.3 (147.6,279.8) | 2695 (1962,3578) | 227.1 (165.5,303.9) | 2 (-7.2,12.2) |
| Islamic Republic of Mauritania | 2398 (1778,3129) | 266 (197.1,349.9) | 5646 (4265,7309) | 288.3 (217.2,379.9) | 2.9 (-0.9,6.8) |
| Principality of Monaco | 40 (29,53) | 50 (37.7,66.2) | 89 (65,122) | 79.8 (59.5,106.4) | 4.6 (-4.2,15.8) |
| Republic of Mali | 8679 (6465,11318) | 267.4 (197.2,353.6) | 21232 (15937,27193) | 283.2 (211.2,371.4) | 4 (-6.1,14.8) |
| Republic of Sudan | 20242 (15914,25545) | 234.5 (182.1,299.9) | 44932 (35370,56827) | 239 (185.7,306.2) | 7.2 (-2.8,18.3) |
| Republic of San Marino | 27 (20,37) | 73 (55.3,98.6) | 85 (62,117) | 96.9 (71.8,130.4) | 4.2 (-6.8,14.6) |

**Table S2:** Deaths due to hypertensive heart disease in 1990 and 2021 and the percentage change in the age-standardized rates (ASRs) per 100,000 individuals by location

|  | | | | | |
| --- | --- | --- | --- | --- | --- |
| Location | X1990_No_.95._UI. | X1990_ASRs_per_100000_.95._UI. | X2021_No_.95._UI. | X2021_ASRs_per_100000_.95._UI. | Percentage_change_in_the_ASRs_per_100000 |
| Global | 4626598 (3672198,5826592) | 125.4 (99,158) | 12505436 (9866066,15827877) | 148.3 (117.3,186.3) | 18.2 (10.6,26) |
| Andean Latin America | 29499 (23627,36701) | 150.2 (118.6,191.6) | 85962 (65683,110961) | 148.4 (112.9,192.7) | -1.2 (-13.4,10.3) |
| Australasia | 7290 (5725,9401) | 31.4 (24.6,40.1) | 30058 (24138,37342) | 52.4 (42,64.4) | 13.5 (-1.3,28.7) |
| Australia | 5975 (4642,7805) | 30.9 (24.1,40.2) | 27202 (21729,33484) | 56.2 (45.2,68.4) | -9.5 (-21.5,3.2) |
| New Zealand | 1315 (932,1803) | 33.7 (24.3,45.5) | 2856 (2065,3922) | 32.2 (23.3,43.7) | -0.4 (-13.9,15.4) |
| Caribbean | 36018 (28469,45489) | 141.5 (112.5,179.6) | 105243 (81142,136189) | 195.5 (151.1,253.3) | 67 (43,93.5) |
| Antigua and Barbuda | 159 (125,197) | 275.5 (216.9,339.8) | 306 (236,394) | 309.3 (241.2,398.4) | 81.5 (50.9,115.5) |
| Barbados | 569 (431,755) | 178.6 (140.1,232) | 1118 (865,1449) | 215.6 (168.9,276) | -4.2 (-16.5,10.8) |
| Belize | 174 (139,218) | 184.4 (146.2,234.6) | 699 (543,882) | 254.9 (195.1,330.8) | 38.1 (24.3,52.5) |
| Bermuda | 50 (38,64) | 82.4 (63.4,106.9) | 227 (171,302) | 161.1 (123.7,210.7) | 12.3 (-4.9,29.1) |
| Dominican Republic | 6675 (5359,8387) | 191.8 (150.6,243) | 18468 (14187,24517) | 188.5 (143.8,251.2) | 6.7 (-5.1,18.8) |
| Grenada | 137 (105,177) | 175.2 (136.3,222.6) | 226 (177,294) | 219.7 (172.4,284.5) | 20.7 (8.1,34.9) |
| Jamaica | 6207 (4916,7921) | 330.5 (263,421.7) | 10038 (7913,12821) | 317 (248.5,406.6) | 38.2 (19.5,60.2) |
| Puerto Rico | 5300 (4065,7001) | 144.6 (112.1,189.4) | 16851 (12755,22248) | 214.3 (164,278) | 95.6 (69.7,123.1) |
| Saint Kitts and Nevis | 57 (43,76) | 144 (110.8,191.7) | 121 (95,156) | 206.6 (160.1,263.9) | 154.4 (117.5,191.3) |
| Saint Lucia | 195 (151,253) | 224.7 (174.4,289.1) | 595 (461,766) | 255.1 (197.4,327.8) | 3.5 (-8.3,16.8) |
| Saint Vincent and the Grenadines | 180 (141,229) | 248.6 (197.5,317.3) | 425 (328,546) | 310.2 (241,395.5) | -1.7 (-17.7,14) |
| United States Virgin Islands | 129 (102,166) | 169.2 (132.2,219.3) | 352 (259,470) | 186.8 (141.8,241.7) | 25.4 (12,40.5) |
| Central Asia | 35124 (25708,46386) | 80.1 (57.8,107.6) | 69365 (48393,95011) | 95.6 (64.6,132.6) | 6.9 (-6.7,20.1) |
| Georgia | 4637 (3275,6460) | 78.3 (55.3,109) | 9299 (4653,13839) | 152.2 (79,223.7) | 0.5 (-12.5,16) |
| Mongolia | 522 (354,728) | 53.6 (35.8,75.3) | 962 (662,1362) | 51.9 (34.1,75.7) | -4.1 (-14.7,7.5) |
| Turkmenistan | 1058 (747,1446) | 62.6 (44.2,86.9) | 2841 (1978,3894) | 82 (55,116) | 48.2 (30.4,66.4) |
| Central Europe | 142712 (104117,188158) | 100.3 (73.7,131.3) | 339147 (247045,439437) | 146 (107.1,186.7) | 43.4 (25,66.6) |
| Bosnia and Herzegovina | 2670 (1972,3470) | 75.6 (54.6,99.4) | 5584 (3737,7682) | 87.9 (59.8,119.8) | 13.6 (-0.1,27.6) |
| Hungary | 13914 (9273,19952) | 96 (65.8,136.1) | 29679 (19624,41792) | 142.4 (97.5,198.4) | 24.8 (10.1,42) |
| Montenegro | 347 (255,468) | 59.4 (43.8,80.5) | 586 (417,834) | 60.3 (43.2,84) | 11.9 (-4,28.4) |
| North Macedonia | 2203 (1602,2929) | 134.7 (97.2,180.9) | 4481 (3149,6298) | 145.1 (104.8,200.2) | -2.7 (-15.2,11.9) |
| Romania | 30048 (19529,42165) | 114.7 (76.2,161) | 54656 (33411,78224) | 138.8 (87.9,194.6) | 10.4 (-5.1,26.7) |
| Central Latin America | 103128 (82807,129101) | 136.8 (108,171.6) | 284539 (222180,367456) | 119 (92.2,154) | 19.3 (3.8,33.9) |
| Central Sub-Saharan Africa | 38770 (28042,51326) | 221.6 (161.5,300.5) | 106346 (78305,139741) | 239.1 (176.9,320.4) | 33.4 (12.6,58.6) |
| Central African Republic | 1689 (1222,2260) | 198.6 (144.3,271.1) | 3440 (2496,4560) | 207.7 (152.6,279) | -1 (-16.6,16.8) |
| Democratic Republic of the Congo | 27118 (19459,35744) | 223.6 (159.3,300.7) | 71715 (52951,94800) | 240.2 (178.2,326.6) | 94.4 (33.7,138.4) |
| East Asia | 1550300 (1198536,1971217) | 216.7 (168.4,272) | 4066468 (3120125,5242374) | 193.2 (147.4,245.9) | -11.4 (-28,7.6) |
| Democratic People's Republic of Korea | 20489 (15857,26581) | 160.3 (121.5,211) | 54979 (42119,71899) | 181.4 (140,237.9) | 49.1 (27.5,77.3) |
| Taiwan (Province of China) | 26792 (20270,35311) | 196.6 (147.5,260.3) | 99331 (76001,129684) | 230.6 (178.4,301) | -3.1 (-20.8,18.3) |
| Eastern Europe | 77819 (57322,105778) | 28.7 (21.3,38.8) | 154963 (107122,212913) | 42.8 (29.8,58.3) | -4.1 (-18.4,14) |
| Republic of Moldova | 1314 (982,1751) | 30.5 (22.8,40.3) | 5861 (4077,8234) | 97.8 (68.4,137.9) | 31 (11.2,54.6) |
| Russian Federation | 48079 (34855,66026) | 27.6 (20.1,37.8) | 104820 (72024,145518) | 42.7 (29.5,59) | 19.3 (-0.8,40.5) |
| Ukraine | 22659 (16375,31114) | 32.1 (23.5,43.8) | 29953 (20897,41634) | 37.3 (25.9,51.2) | 45.6 (30.6,60.2) |
| Eastern Sub-Saharan Africa | 175066 (131637,224238) | 282.2 (216.1,371.2) | 425630 (324248,532350) | 291.8 (224,375.4) | 3.1 (-14.1,22.6) |
| United Republic of Tanzania | 27778 (20595,36322) | 297.9 (220.9,394.7) | 64360 (47219,83565) | 282.4 (209.2,370.7) | 16.3 (-1,37.3) |
| High-income Asia Pacific | 105137 (75815,138635) | 57.4 (41.7,75) | 297204 (219641,391369) | 57.6 (43.8,72.4) | 70.7 (49,93.5) |
| Brunei Darussalam | 63 (46,83) | 58 (35.8,82.7) | 176 (126,242) | 57 (36.2,80.5) | -20.6 (-37.2,-4.9) |
| Japan | 75062 (51652,100265) | 48.7 (34.4,64.7) | 195613 (140989,264657) | 44.9 (33.6,58.3) | 227.7 (174.1,285.5) |
| Republic of Korea | 28327 (21349,36057) | 107.7 (78.2,142.2) | 91856 (71867,116578) | 103 (81.1,130) | 48.3 (25.5,72.7) |
| High-income North America | 316482 (237496,411000) | 91.8 (70.3,118.4) | 949322 (738427,1167271) | 150.7 (119.5,181.9) | 1.5 (-13.7,20.6) |
| Canada | 12490 (9811,15645) | 38.7 (30.7,48.4) | 63810 (50259,80384) | 94.9 (75.7,117.7) | 7.8 (-6.9,26.4) |
| Greenland | 24 (19,31) | 63.6 (47.2,80.7) | 52 (39,67) | 84.3 (63.6,108.3) | 53.8 (33.3,78.7) |
| United States of America | 303960 (226202,396179) | 97.3 (74.3,126.1) | 885445 (687332,1097741) | 157.5 (124.1,191.3) | 21 (0.7,43.1) |
| North Africa and Middle East | 365066 (297761,449144) | 239.5 (192.8,299.2) | 1027631 (814739,1265656) | 243.3 (192.1,303.6) | 10.9 (-5.6,29.2) |
| Palestine | 1948 (1579,2382) | 233 (185.2,292.7) | 5551 (4410,6865) | 234.1 (184,299.1) | 57.6 (35.4,85.3) |
| Syrian Arab Republic | 9096 (7232,11237) | 188.8 (145,240.9) | 20771 (15724,27068) | 178.9 (135.2,235.9) | 77.2 (50.1,114.2) |
| United Arab Emirates | 966 (771,1189) | 193.6 (150.9,245.9) | 12043 (9193,16090) | 247.1 (189.3,324.3) | -13 (-21.1,-5) |
| Oceania | 2767 (2167,3496) | 115.7 (90.2,148) | 6738 (5279,8527) | 106.9 (83.7,136.8) | -29.7 (-39.3,-17.4) |
| American Samoa | 21 (16,27) | 109.9 (85.3,141.3) | 39 (29,52) | 91.6 (67.1,123.8) | 12.3 (-1,27) |
| Cook Islands | 38 (30,47) | 332.9 (259.4,426.9) | 80 (63,103) | 317.5 (252.2,402.5) | -9.4 (-22.1,6.7) |
| Guam | 139 (109,172) | 212.1 (163,272) | 251 (187,333) | 119.7 (89.3,160.4) | -5.8 (-20.2,11.5) |
| Northern Mariana Islands | 10 (8,13) | 72.1 (55.1,93.9) | 27 (21,37) | 63.2 (48.2,84.9) | -0.2 (-12.4,13.5) |
| Solomon Islands | 98 (77,125) | 90 (69.7,117.4) | 255 (198,327) | 86.8 (66.6,112.9) | -9.4 (-19,-1) |
| Tokelau | 2 (1,2) | 138.2 (108.4,179.2) | 2 (1,2) | 120.2 (91.8,160.2) | -10.8 (-24.1,2.1) |
| Tuvalu | 7 (6,9) | 133.4 (103,169.8) | 11 (9,15) | 118.8 (91.1,152.1) | 50.9 (31.8,73.9) |
| South Asia | 470748 (374964,589000) | 104.1 (82.7,129.6) | 1432788 (1119541,1828057) | 111.1 (86.6,142.7) | -7.7 (-20.7,6.5) |
| Southeast Asia | 370474 (301792,452815) | 168.1 (135.9,207.4) | 960247 (767865,1203976) | 163.3 (131.2,205.2) | 7.9 (-0.9,19) |
| Lao People's Democratic Republic | 2827 (2176,3630) | 164.3 (129.2,211.5) | 6753 (5273,8611) | 170.1 (130,221.1) | 10.2 (-0.7,22.6) |
| Malaysia | 5459 (4289,6897) | 64 (49.2,83.6) | 15482 (11993,20002) | 58.9 (45.2,76.7) | 4.6 (-5.8,16.3) |
| Southern Latin America | 39029 (28709,52164) | 88.1 (64.9,117.8) | 98819 (70049,130976) | 110.1 (78.3,145.8) | 8.6 (-1.4,21.4) |
| Southern Sub-Saharan Africa | 48002 (35809,61790) | 196.8 (147.1,256.1) | 108006 (80828,139595) | 210.6 (157.4,272.2) | 7.5 (-3.5,21.1) |
| Tropical Latin America | 122755 (95999,153967) | 149.9 (118.5,187.4) | 411264 (317930,527720) | 166 (129.1,213.6) | 13.7 (2.4,27.2) |
| Western Europe | 394304 (293760,522422) | 65.8 (49.5,86.4) | 1094895 (830592,1423768) | 100.8 (78,128.1) | 5.1 (-4.6,15.3) |
| Ireland | 584 (411,813) | 14.7 (10.5,20.1) | 2426 (1684,3302) | 29.5 (21,39.6) | -10.9 (-19.2,-3.3) |
| Western Sub-Saharan Africa | 196108 (151200,249117) | 257.4 (201.8,331.6) | 450801 (347733,569919) | 266.3 (206.7,342.2) | -11.8 (-20.3,-4.3) |
| Burkina Faso | 9728 (7143,12831) | 266.9 (199,350.2) | 23000 (17386,30261) | 281.9 (213.1,377.8) | 13.2 (-1.8,29) |
| Democratic Socialist Republic of Sri Lanka | 16412 (12864,20932) | 172.6 (133.1,221.9) | 42928 (33236,55632) | 164.8 (128.8,213) | 17.3 (4.8,32.1) |
| Republic of Armenia | 2250 (1634,3008) | 95.7 (68.2,129.7) | 5546 (4222,7105) | 127.8 (97.5,163.3) | 49.3 (32.6,67.2) |
| Republic of the Philippines | 50220 (40648,61454) | 191.7 (154.2,236.4) | 144189 (115437,178488) | 200 (157.7,249.9) | -61.2 (-68,-53.2) |
| Republic of the Union of Myanmar | 31942 (25145,40959) | 162.8 (127.2,207.4) | 71192 (55944,91130) | 162.7 (126.6,208.3) | 222.1 (166.7,282.3) |
| Republic of Maldives | 81 (64,103) | 114.4 (88.1,147) | 403 (319,507) | 130.8 (99.7,170.1) | 312.8 (227.3,417.6) |
| Republic of Vanuatu | 49 (38,63) | 94.9 (73.2,121.9) | 139 (108,178) | 94.4 (72.4,123.1) | 169.3 (117.2,240.6) |
| Kingdom of Tonga | 39 (31,50) | 78.6 (61.3,99.9) | 58 (44,76) | 74.6 (56.8,98) | 221.2 (162.9,297.5) |
| Republic of Indonesia | 153918 (124479,188583) | 189.5 (151.4,232.2) | 380544 (298134,479534) | 190.4 (148.5,240.8) | 54.3 (34.8,73.1) |
| Kingdom of Cambodia | 7236 (5683,9247) | 193.7 (147.6,254.4) | 20955 (16200,26879) | 196.7 (153.5,254.2) | 16.2 (-0.9,35.9) |
| Federated States of Micronesia | 58 (44,74) | 132.2 (101.8,169.8) | 76 (59,97) | 128.2 (98.2,166.3) | 3.4 (-0.9,8.6) |
| Independent State of Samoa | 113 (88,144) | 149.6 (116.2,193) | 173 (136,226) | 132.6 (102.8,175.2) | 3.9 (-6,16.2) |
| Independent State of Papua New Guinea | 1607 (1254,2043) | 110.2 (86,141.6) | 4474 (3509,5595) | 105.1 (82.6,134.7) | 2.2 (-7.4,12.4) |
| Republic of the Marshall Islands | 18 (14,24) | 130.1 (101.4,168.8) | 34 (26,44) | 121.3 (93.9,156.5) | 0.5 (-8.9,10.1) |
| Republic of Fiji | 356 (280,449) | 121.3 (93.4,157.4) | 756 (580,979) | 117.4 (91.3,154.7) | 5.1 (-5,16) |
| Republic of Kiribati | 24 (18,30) | 77.3 (59.2,99.9) | 43 (33,56) | 74.6 (56.2,97.3) | 11.5 (5.6,18.4) |
| People's Republic of China | 1503019 (1161178,1916382) | 218.2 (169.3,274.8) | 3912158 (2989417,5056002) | 192.5 (146.7,245) | 3 (-1,7.6) |
| Republic of Tajikistan | 4380 (3244,5778) | 170.3 (124,229.1) | 8278 (6037,10852) | 163.2 (112,221) | -1.6 (-11.2,8.8) |
| Kyrgyz Republic | 1729 (1267,2331) | 62.2 (44.4,84.2) | 3881 (2703,5169) | 92.8 (62.8,127.4) | -1 (-10.5,10.3) |
| Republic of Kazakhstan | 7900 (5778,10651) | 66.2 (47.6,90) | 9401 (6295,13336) | 58.7 (39,84) | 0 (-9.5,10.4) |
| Socialist Republic of Viet Nam | 80435 (62618,103863) | 214.8 (167.6,277.8) | 182456 (142953,233597) | 205.7 (159.8,267.4) | 5.6 (-4.8,17.4) |
| Czech Republic | 4326 (3138,5982) | 32 (23.4,43.5) | 23607 (16047,33674) | 104.7 (72.8,143.7) | -4.7 (-13.5,5.4) |
| Republic of Bulgaria | 16441 (11773,22258) | 140.1 (103.3,184.4) | 35325 (25085,48102) | 239 (173.4,315.9) | -1.3 (-12.3,9.8) |
| Republic of Uzbekistan | 8266 (6159,10768) | 73.6 (53.2,98.1) | 20669 (14514,28162) | 87.8 (59.8,125.3) | 6.1 (-3.3,17.6) |
| Democratic Republic of Timor-Leste | 423 (338,526) | 191.6 (148.3,247.2) | 1433 (1124,1876) | 178.2 (139.9,230.1) | -5.2 (-14.6,4.2) |
| Republic of Croatia | 5105 (3601,6941) | 91.9 (63.7,125.1) | 6749 (4344,9160) | 73 (49.3,96.3) | 3 (-6.7,14.2) |
| Republic of Azerbaijan | 4381 (3176,5864) | 99.3 (71.1,133.9) | 8488 (5942,11489) | 98.3 (66.7,134.8) | 0.2 (-10.5,14) |
| Kingdom of Thailand | 19727 (15690,25064) | 63.5 (49.5,81.2) | 88634 (68950,116757) | 82.7 (64.3,108.5) | -1.8 (-14.7,17.1) |
| Republic of Albania | 1532 (1124,2032) | 82 (59,110.8) | 3718 (2632,5124) | 84.6 (61.1,114) | -7.8 (-19.1,4.7) |
| Republic of Lithuania | 987 (719,1346) | 22.2 (16.3,30) | 3627 (2409,5263) | 59.8 (39.5,85.9) | -4.4 (-19.6,14.8) |
| Republic of Latvia | 612 (438,847) | 17.3 (12.4,23.6) | 3151 (2073,4603) | 71.2 (46.9,103.9) | 46.1 (27.3,74.7) |
| Republic of Estonia | 1312 (968,1747) | 65.4 (48.9,86.3) | 6158 (4003,8647) | 210.6 (142.8,290.9) | 64.2 (42.9,87.3) |
| Republic of Cyprus | 405 (275,568) | 56.9 (39.2,77.8) | 1381 (978,1934) | 67.4 (48.9,92.3) | 145.5 (112.8,183.2) |
| Republic of Austria | 10735 (8064,13167) | 86.3 (66.2,105.1) | 21767 (16092,28138) | 103.8 (78.7,132.5) | 32.5 (16.4,51.1) |
| Kingdom of Belgium | 3059 (2182,4227) | 19.4 (14.1,26.3) | 5979 (4434,8252) | 23 (17.6,30.5) | 61.9 (39.8,86.6) |
| Republic of Belarus | 2856 (2044,3852) | 22.4 (16.1,30.3) | 1393 (954,1954) | 8.7 (6,12) | 1.6 (-6.4,10.8) |
| Kingdom of Denmark | 1147 (796,1578) | 13.5 (9.7,18.2) | 3479 (2424,4915) | 27.5 (19.6,37.3) | -3.2 (-14.1,9.5) |
| Principality of Andorra | 43 (32,57) | 82.9 (62.4,110) | 139 (105,183) | 85.3 (64.9,112) | 6 (-5,19.3) |
| Republic of Slovenia | 2026 (1352,2820) | 83.7 (56.1,115.5) | 7186 (4739,10174) | 148.3 (100.8,206.7) | 29.1 (14.3,46.3) |
| Slovak Republic | 3613 (2728,4757) | 61.5 (46.7,80.7) | 9341 (6639,13164) | 96.9 (70.2,133.5) | -6.1 (-18.6,8.1) |
| Republic of Serbia | 7857 (5308,10891) | 80.6 (53.3,112.2) | 15331 (9429,21626) | 89.4 (56.6,123.2) | 10.7 (2.9,17.9) |
| Federal Republic of Germany | 103336 (76205,141423) | 79.2 (59,106.5) | 252729 (177135,345631) | 111.5 (79.9,150.4) | -5.7 (-17.1,6.7) |
| Republic of Finland | 2615 (1749,3576) | 36.6 (25.4,49.4) | 15435 (10231,22314) | 107.6 (73.8,150.4) | 11.8 (-2,25.8) |
| Republic of Singapore | 1686 (1272,2157) | 78.4 (54.4,104.3) | 9560 (7224,12371) | 114.5 (87.3,147.9) | -6.6 (-17.8,5.3) |
| French Republic | 106021 (79900,141753) | 118.9 (90.6,158) | 247024 (179959,334930) | 145.1 (109,193.9) | -4.6 (-16,7.5) |
| Argentine Republic | 26995 (19723,36504) | 87.3 (64.5,117.4) | 57338 (38246,77055) | 99.3 (67.4,132.6) | -1.7 (-14,11) |
| Republic of Poland | 50348 (38535,66385) | 119.1 (91.9,155.4) | 137968 (105620,175866) | 183.2 (141.1,230.1) | 0.7 (-11.4,15.3) |
| United Kingdom of Great Britain and Northern Ireland | 18302 (12529,24992) | 20.1 (14.5,27) | 46948 (33456,63289) | 35.3 (25.6,46.5) | 31.6 (15.1,51.6) |
| Swiss Confederation | 5080 (3299,7198) | 45.8 (31,63.7) | 17438 (12462,23781) | 86.2 (63.3,115.2) | 0.5 (-10.4,12.1) |
| Kingdom of Sweden | 7222 (4887,10667) | 43.9 (30.5,63.5) | 30386 (21432,42602) | 118.7 (84.2,161.8) | 21.4 (6.2,38.2) |
| Republic of Guyana | 864 (679,1101) | 247.7 (190.1,323.5) | 1492 (1160,1938) | 264.8 (206.6,341.5) | 3.3 (-10.4,15.6) |
| Kingdom of Spain | 24331 (17680,33504) | 44.5 (32.2,60.8) | 98813 (77720,119433) | 86.7 (70.2,103.9) | 1.9 (-11.3,15.7) |
| Commonwealth of Dominica | 152 (118,200) | 251.5 (197.4,328.1) | 206 (160,270) | 260.3 (202.9,339.9) | -5.2 (-18.9,7.8) |
| Kingdom of Norway | 1185 (651,1749) | 16.2 (9.4,23.3) | 3018 (1998,4329) | 26.8 (18.1,37.5) | 7.2 (-4.4,19.4) |
| Portuguese Republic | 7159 (5393,9602) | 54.3 (41.2,71.5) | 21202 (15195,28667) | 74.3 (55.1,98.6) | -0.6 (-11.9,12.2) |
| Kingdom of the Netherlands | 3824 (2919,4991) | 18.4 (14.3,23.8) | 14549 (10624,19619) | 38.3 (27.9,51) | 27.6 (13.7,42.1) |
| Republic of Cuba | 7138 (5544,9100) | 68.5 (53.4,87.9) | 34615 (26158,44563) | 174.4 (134.5,224.5) | 0.9 (-9.4,14.2) |
| Commonwealth of the Bahamas | 455 (361,573) | 309.3 (241.7,391.1) | 1208 (943,1550) | 329.9 (257.4,428.2) | -7.6 (-17.2,2.6) |
| Republic of Malta | 130 (97,174) | 32.9 (24.4,43.5) | 650 (470,891) | 61.5 (45.4,82.2) | -16.6 (-30.4,-1.4) |
| Grand Duchy of Luxembourg | 198 (128,284) | 37 (24.5,51.7) | 710 (507,966) | 63 (45.5,84.4) | -4.6 (-14.8,6.7) |
| Republic of Guatemala | 1995 (1569,2615) | 67.9 (52.1,90.4) | 6590 (4962,8819) | 64 (48,85.5) | -3.2 (-15.5,12.3) |
| Republic of Honduras | 3734 (2958,4648) | 195.4 (153,247.5) | 11389 (8660,14805) | 195 (147.2,254.7) | -43.6 (-53,-33.5) |
| Republic of El Salvador | 2430 (1871,3136) | 85 (64.5,110.3) | 5025 (3803,6684) | 77 (58.1,102.9) | -3.4 (-15.6,10.9) |
| Republic of Costa Rica | 2360 (1874,3029) | 139.3 (108.9,179.8) | 8556 (6503,11105) | 156.5 (117.8,204.2) | -6.7 (-18,6.3) |
| Republic of Italy | 91410 (59107,124524) | 100.9 (67.1,136.5) | 293224 (219071,374596) | 169.9 (129.6,213.9) | -3 (-14.6,9.3) |
| Republic of Colombia | 29780 (23639,37936) | 187.9 (145.2,243.1) | 73340 (55690,97055) | 132.1 (100.3,175.3) | -12.8 (-26.6,3.7) |
| Hellenic Republic | 5928 (4238,7837) | 40.2 (29.3,52.9) | 11594 (8060,15784) | 41.1 (29.8,53.5) | -7.7 (-19.7,6) |
| State of Kuwait | 1904 (1559,2321) | 306.4 (243.7,387.8) | 8228 (6490,10337) | 286.2 (222.3,367.5) | -12.3 (-25.6,1.1) |
| State of Israel | 1163 (793,1594) | 24.6 (17,33.3) | 4719 (3370,6384) | 35.9 (26.3,48.5) | -8.6 (-19.9,6) |
| Hashemite Kingdom of Jordan | 3883 (3174,4778) | 305.7 (244.4,390.3) | 23947 (19043,29713) | 341.9 (268.8,427.8) | -4.6 (-15.8,7.8) |
| Republic of Iraq | 16235 (13093,20154) | 206.6 (162.6,262.4) | 42680 (33586,53788) | 194.9 (151.4,253.3) | -11.4 (-22.5,0.6) |
| Islamic Republic of Iran | 52354 (42370,64334) | 226.1 (183.3,279) | 185438 (147645,229529) | 250.2 (196.9,312.9) | -3.5 (-17.5,14.1) |
| Republic of Iceland | 36 (24,50) | 12.3 (8.1,16.9) | 148 (103,204) | 24.2 (17.4,33.2) | -13 (-25.6,0.8) |
| Kingdom of Bahrain | 237 (189,298) | 139.9 (108.7,180.5) | 1536 (1197,1963) | 180.7 (139.3,231.8) | -5.1 (-17,8.6) |
| Arab Republic of Egypt | 57917 (46297,71094) | 241.4 (190.9,306.9) | 125969 (97722,158183) | 226.7 (176.4,290.9) | -10.9 (-22.4,3.4) |
| People's Democratic Republic of Algeria | 31522 (24802,39505) | 272.2 (216.5,339) | 97354 (76502,122619) | 288.6 (224.5,366.6) | -0.5 (-12.4,14.8) |
| Republic of Paraguay | 3650 (2911,4552) | 170.7 (134.2,217.7) | 10733 (8380,13754) | 193.6 (149,249.8) | 6.7 (-1.1,13.8) |
| Federative Republic of Brazil | 119105 (93006,149374) | 149.3 (117.9,187.4) | 400532 (309339,513910) | 165.3 (128.3,212.7) | 5.7 (-7.3,19.4) |
| Eastern Republic of Uruguay | 2342 (1672,3249) | 60.1 (43.7,81.8) | 5329 (3366,7310) | 87.9 (57.7,119.6) | 0.9 (-10.4,13.9) |
| Bolivarian Republic of Venezuela | 16888 (13437,21551) | 187.7 (144.4,243.4) | 48741 (37347,64086) | 173.2 (132.1,230.2) | 8.5 (-0.3,16.5) |
| Republic of Panama | 1283 (992,1678) | 90.1 (68.8,118.2) | 6060 (4579,7843) | 135.9 (102.7,176.9) | 20.4 (4.9,36.7) |
| People's Republic of Bangladesh | 53125 (42591,67255) | 131.9 (103.1,168) | 171255 (133385,219308) | 139.5 (108.7,178.4) | 4.5 (-5.6,13.8) |
| Islamic Republic of Afghanistan | 15224 (11956,19153) | 260.8 (204.7,333.9) | 23769 (18964,29386) | 252.6 (194.6,323.8) | -2.8 (-9.5,3.6) |
| Kingdom of Saudi Arabia | 15638 (12867,19303) | 279.9 (222.2,355.7) | 50759 (40865,63580) | 289.1 (222.8,372.7) | 1.5 (-8.2,13.8) |
| Kingdom of Morocco | 31466 (25134,39184) | 230.6 (181,292.5) | 74291 (58209,94416) | 232.2 (180,299.9) | 0.5 (-7.7,7.3) |
| Republic of Yemen | 10886 (8842,13456) | 251.4 (197.6,319.4) | 33875 (27386,41746) | 253.8 (200.1,325.2) | 3.5 (-8.8,17.7) |
| State of Qatar | 198 (157,252) | 143.8 (113.2,184.3) | 2081 (1580,2739) | 174.5 (133.7,227.2) | -7.9 (-20.4,6.3) |
| Republic of Tunisia | 12314 (9935,15535) | 264.2 (210.6,335.1) | 35846 (27616,45233) | 283.4 (220,357.5) | 14.3 (-1.3,31.4) |
| Republic of Turkey | 72979 (58126,91111) | 235.9 (185,297.1) | 208006 (160011,267069) | 234.4 (181.5,301.2) | 23.8 (10,39.2) |
| Republic of Djibouti | 329 (245,426) | 299.1 (221.6,392.1) | 1596 (1167,2060) | 300.7 (218.9,395.2) | -0.1 (-11.3,12.9) |
| Republic of Chile | 9690 (7274,12757) | 102.3 (74.9,135.1) | 36147 (26855,47810) | 139.5 (104.3,184) | 4.3 (-2.3,10.3) |
| Union of the Comoros | 496 (364,640) | 297.6 (219.3,390.8) | 1332 (988,1718) | 304.1 (225.6,398.9) | 0.3 (-10,12.7) |
| Republic of Burundi | 5659 (4137,7437) | 278.1 (203.5,373.1) | 11909 (8826,15355) | 288.9 (211.4,378.8) | -4.6 (-15.7,8.1) |
| Republic of Equatorial Guinea | 330 (238,445) | 214.2 (154.5,293.2) | 1057 (785,1383) | 243.6 (177.4,325.9) | 30.2 (15.2,48.6) |
| Republic of Haiti | 4247 (3289,5438) | 159.7 (122.2,206) | 9778 (7626,12262) | 160.6 (122.3,209) | -7 (-17,5.8) |
| Republic of Peru | 14667 (11722,18441) | 127.4 (99.9,162.9) | 42384 (31863,55132) | 126.8 (94.7,166.6) | -4.2 (-15.2,10.1) |
| Republic of the Congo | 1847 (1333,2428) | 216.3 (159.1,291.3) | 5136 (3795,6826) | 234.8 (174.2,315) | 25 (10.3,41.6) |
| Republic of Angola | 6556 (4835,8659) | 217.9 (157.6,293.3) | 22744 (16487,29818) | 240.1 (175.5,316.4) | 46.3 (24.7,70.1) |
| Republic of Suriname | 449 (358,567) | 189.5 (149.4,241.3) | 1259 (961,1606) | 212.1 (162.9,271.4) | 13.7 (-2.9,31.1) |
| Islamic Republic of Pakistan | 58297 (45927,73617) | 119.7 (94,149.7) | 125006 (97730,160203) | 125.1 (94.9,159.1) | 36.4 (18.7,56.5) |
| Federal Democratic Republic of Nepal | 7343 (5793,9136) | 101.2 (78.2,129.4) | 24573 (19181,31598) | 121.8 (95,156.7) | 7 (2.6,12.6) |
| Plurinational State of Bolivia | 3830 (3028,4840) | 132 (102.9,170.9) | 12584 (9667,16457) | 149.9 (114,196.3) | 3.3 (-6.6,13.9) |
| Republic of Ecuador | 11002 (8835,13730) | 213.2 (168,270.9) | 30995 (23949,40975) | 193 (149.3,254.8) | 2.4 (-7.5,14.2) |
| Kingdom of Lesotho | 1512 (1086,2081) | 200 (143.7,278.5) | 1867 (1297,2508) | 204.8 (145.6,273.5) | 2.4 (-8.7,13.6) |
| Republic of Trinidad and Tobago | 1663 (1322,2136) | 203.1 (160.1,259.1) | 3698 (2839,4796) | 197.7 (152.8,253.6) | 10.6 (0.7,23.8) |
| Republic of Uganda | 16760 (12519,21315) | 298.1 (221.2,390.4) | 41182 (31316,51826) | 316.3 (241.4,414.8) | 8.6 (3.7,14.6) |
| Republic of Madagascar | 12973 (9515,16598) | 299.4 (221.6,394.7) | 26909 (19685,35263) | 294.6 (218.6,387.3) | -2.7 (-13.4,9.2) |
| Republic of Mozambique | 12024 (8653,16261) | 239.3 (176.3,324.2) | 22833 (16501,29847) | 239.2 (168.1,317.9) | 10.7 (1.8,18.5) |
| Republic of Botswana | 933 (647,1273) | 203.1 (140.8,277.9) | 2631 (1891,3487) | 209.8 (149.3,283.7) | 10.7 (1.6,18.8) |
| Republic of Zambia | 6923 (5101,9025) | 292.7 (215.7,391.8) | 17823 (13059,23217) | 301.6 (226.2,391.6) | 13.4 (-1,28.4) |
| Republic of Mauritius | 1097 (857,1431) | 169.8 (131.7,225.7) | 3643 (2816,4632) | 210.2 (162.2,267.8) | 53.3 (34.7,72.3) |
| Republic of Seychelles | 161 (126,205) | 282 (221.3,359.9) | 296 (235,376) | 282.9 (221.6,364) | 2.9 (-12.3,19.9) |
| Republic of Liberia | 2772 (2056,3647) | 269.7 (201.8,356.5) | 5056 (3854,6511) | 275.2 (206.3,360.4) | 20.3 (-1.2,44.7) |
| Republic of Guinea-Bissau | 785 (581,1043) | 244.6 (181.8,326) | 1471 (1115,1930) | 250.6 (184.3,332.5) | 18.3 (-8.9,54.5) |
| Sultanate of Oman | 1376 (1160,1660) | 194.5 (160,242.7) | 5509 (4319,7028) | 256 (199.5,332.8) | 18.5 (1.2,43.8) |
| Republic of Guinea | 8005 (5976,10655) | 269.4 (202.8,361.9) | 13776 (10349,17796) | 273.2 (202.6,357.9) | 103.1 (64.7,147.3) |
| State of Libya | 4717 (3810,5737) | 257.4 (205,323.4) | 12464 (9808,15544) | 252.9 (195.3,319.8) | 193.9 (121.5,279.9) |
| Republic of Nicaragua | 2315 (1847,2968) | 164.4 (128.6,213.3) | 6686 (5157,8659) | 146.7 (112.8,192.7) | 22.1 (2,46.2) |
| Republic of the Gambia | 777 (567,995) | 256.7 (188.6,338.4) | 2325 (1730,3037) | 263.5 (194,349.7) | 40.7 (20.9,64.2) |
| Republic of Ghana | 14049 (10994,17964) | 273.9 (209.5,364.8) | 39122 (29121,50718) | 278 (208.8,367.6) | 2.2 (-18.4,26) |
| Lebanese Republic | 3765 (3008,4802) | 193.7 (151.5,249.5) | 11626 (8948,14947) | 184.8 (142.4,236.4) | 97.3 (65.2,140.6) |
| United Mexican States | 42341 (33212,53402) | 110.5 (85.6,139.9) | 118152 (87685,154937) | 100 (74.1,131.1) | 101.3 (62.5,147.6) |
| Republic of Cameroon | 10478 (7964,13357) | 281.8 (208.6,369.1) | 29984 (22631,39372) | 277.7 (208.9,368.4) | 46.1 (21.7,76.2) |
| Republic of C么te d'Ivoire | 8227 (6224,10706) | 260.2 (195.2,348.7) | 25205 (18928,32311) | 268.1 (202.2,354.8) | 68.4 (44.5,98.8) |
| Republic of Chad | 6466 (4758,8521) | 251.3 (186.1,333.4) | 13319 (10071,17001) | 267.4 (196.4,350.1) | 70 (39.1,108.8) |
| Republic of Benin | 4950 (3714,6545) | 268.8 (201.6,355.7) | 12735 (9652,16403) | 276.7 (203.8,366.9) | 87.1 (56.2,126.6) |
| Gabonese Republic | 1229 (889,1651) | 242.4 (178.8,328.9) | 2254 (1656,3009) | 254.8 (185.9,340.4) | 59.5 (34.9,83.6) |
| Republic of Zimbabwe | 6987 (5093,9324) | 201.5 (147.8,275.6) | 11038 (7703,14486) | 196.1 (138.7,265.6) | 107.7 (62.3,152.7) |
| Togolese Republic | 2682 (2033,3513) | 257.1 (192.8,340.3) | 8481 (6405,10940) | 268 (203,353.8) | 65.5 (39.3,104) |
| Republic of Sierra Leone | 4632 (3464,6141) | 247.2 (186.3,328.3) | 8889 (6577,11664) | 264.9 (194.6,351.9) | 36.9 (15.9,59.2) |
| Kingdom of Eswatini | 492 (356,656) | 205.5 (147.3,281.4) | 973 (691,1295) | 210.4 (149.5,284.7) | 32.8 (15.3,52.3) |
| Democratic Republic of Sao Tome and Principe | 166 (124,214) | 277.2 (207.9,357.4) | 285 (211,363) | 290.1 (214.4,376.9) | 95.1 (52.1,154.4) |
| Federal Republic of Nigeria | 97321 (76006,122714) | 250.1 (195.4,313.6) | 201897 (159272,249677) | 257.2 (202,324) | 170.4 (115.7,234.9) |
| Republic of Palau | 5 (4,7) | 57.4 (44.1,73.7) | 10 (8,13) | 52.5 (40,69.9) | 88.2 (58.3,133.6) |
| Republic of Niue | 3 (2,4) | 129.2 (100.6,165.3) | 2 (2,3) | 119.2 (92,156.2) | 75.4 (54.9,95.2) |
| Republic of Malawi | 8564 (6247,11210) | 268.6 (199.9,356.7) | 17162 (12596,22120) | 266 (197.5,351.9) | 3.4 (-0.8,7.9) |
| Kingdom of Bhutan | 244 (197,303) | 131.7 (103.7,166.8) | 755 (598,950) | 132.9 (104.2,168.2) | 2.9 (-6.1,14.6) |
| Republic of India | 351739 (276997,440739) | 97.9 (77,122.4) | 1111198 (849201,1438465) | 106.2 (82,137.4) | 5.6 (-4.3,16.3) |
| Federal Republic of Somalia | 4824 (3670,6249) | 258.3 (192,341.7) | 12368 (9261,16240) | 246.3 (181,329.9) | 3.1 (-6.9,13.3) |
| Republic of Nauru | 4 (3,5) | 106.9 (80.8,138.9) | 5 (3,6) | 93.2 (71.7,125) | -1.5 (-10.5,9.7) |
| Republic of Rwanda | 6499 (4794,8479) | 280.7 (207.9,373.7) | 16054 (11677,20918) | 296.5 (218.9,389.7) | 6.4 (-2.8,18.5) |
| Federal Democratic Republic of Ethiopia | 42281 (32269,53799) | 270.8 (211.6,346.4) | 117032 (92515,145979) | 301.9 (241.2,382.3) | 3 (-6,14.5) |
| State of Eritrea | 2252 (1628,2953) | 274.2 (204,366.8) | 6491 (4804,8400) | 288.1 (211.8,380.9) | 2.6 (-7.5,14.4) |
| Republic of Kenya | 20620 (16034,25924) | 290.3 (228.1,368.4) | 58551 (46258,73320) | 299 (234.6,374.6) | 1.5 (-9.1,13.8) |
| Republic of South Sudan | 6959 (5160,9138) | 304.1 (223.1,406.1) | 9655 (7232,12431) | 300 (220.7,394.2) | 1.4 (-8.4,11.9) |
| Republic of Cabo Verde | 701 (523,918) | 297 (222.1,385.6) | 1292 (974,1679) | 306.1 (228.2,401.3) | 2.4 (-7,13.1) |
| Republic of South Africa | 36968 (27586,47246) | 195.6 (145.8,253.3) | 88802 (66718,113987) | 212.4 (159.1,273.6) | 2.1 (-6.7,11.9) |
| Republic of Senegal | 7263 (5373,9409) | 253.2 (187.2,336.2) | 18406 (13867,24074) | 263.4 (198.6,348) | 5.9 (-3.7,16.9) |
| Republic of the Niger | 6021 (4531,7795) | 264.5 (198.1,351.3) | 18675 (13793,24197) | 269.9 (203.1,357.2) | 8.4 (-2.2,19.6) |
| Republic of Namibia | 1110 (783,1504) | 205.3 (147.6,279.8) | 2695 (1962,3578) | 227.1 (165.5,303.9) | 2 (-7.2,12.2) |
| Islamic Republic of Mauritania | 2398 (1778,3129) | 266 (197.1,349.9) | 5646 (4265,7309) | 288.3 (217.2,379.9) | 2.9 (-0.9,6.8) |
| Principality of Monaco | 40 (29,53) | 50 (37.7,66.2) | 89 (65,122) | 79.8 (59.5,106.4) | 4.6 (-4.2,15.8) |
| Republic of Mali | 8679 (6465,11318) | 267.4 (197.2,353.6) | 21232 (15937,27193) | 283.2 (211.2,371.4) | 4 (-6.1,14.8) |
| Republic of Sudan | 20242 (15914,25545) | 234.5 (182.1,299.9) | 44932 (35370,56827) | 239 (185.7,306.2) | 7.2 (-2.8,18.3) |
| Republic of San Marino | 27 (20,37) | 73 (55.3,98.6) | 85 (62,117) | 96.9 (71.8,130.4) | 4.2 (-6.8,14.6) |

**Table S3:** DALYs due to hypertensive heart disease in 1990 and 2021 and the percentage change in the age-standardized rates (ASRs) per 100,000 individuals by location

| **Table S3:**DALYs due to hypertensive heart disease in 1990 and 2021 and the percentage change in the age-standardised rates (ASRs) per 100,000, by location | | | | | |
| --- | --- | --- | --- | --- | --- |
| Location | X1990_No_.95._UI. | X1990_ASRs_per_100000_.95._UI. | X2021_No_.95._UI. | X2021_ASRs_per_100000_.95._UI. | Percentage_change_in_the_ASRs_per_100000 |
| Global | 15473830 (12310725,17311822) | 406.5 (328.9,452.2) | 25462185 (21493312,28047521) | 301.6 (255.1,332.1) | -25.8 (-34.5,-10.1) |
| Andean Latin America | 53334 (45900,60206) | 267.1 (230.8,301.8) | 87400 (70638,106994) | 151 (122.1,184.7) | -43.5 (-52.8,-32.1) |
| Australasia | 12664 (11732,13304) | 56.3 (51.8,59.3) | 22285 (19484,24367) | 38.8 (34.5,42) | -28.1 (-47.1,3.4) |
| Australia | 9900 (9123,10456) | 53 (48.5,56.1) | 19217 (16782,21092) | 39.4 (35,42.9) | -50.5 (-60.9,-37.6) |
| New Zealand | 2764 (2577,2918) | 72.7 (67.5,76.8) | 3068 (2698,3325) | 35.3 (31.4,38.1) | -43.6 (-58.4,-23.9) |
| Caribbean | 105057 (86186,124660) | 411.5 (340.5,485.4) | 220547 (183991,262110) | 408.8 (340.5,486.4) | -31.1 (-34.6,-27.4) |
| Antigua and Barbuda | 385 (357,411) | 706 (653.6,755.6) | 826 (768,889) | 819.5 (763.1,877.4) | -25.7 (-29.6,-21.4) |
| Barbados | 1015 (945,1080) | 346.7 (323.5,367.2) | 1582 (1290,1878) | 310.8 (252.5,369.3) | -51.5 (-54.7,-48) |
| Belize | 360 (336,389) | 381.2 (356.1,411.4) | 1571 (1391,1767) | 526.5 (466.1,591.5) | -0.7 (-13.7,12.8) |
| Bermuda | 114 (107,122) | 190.1 (178.3,202.4) | 227 (196,275) | 160.7 (138.3,195.1) | 16.1 (6.6,26.7) |
| Dominican Republic | 15303 (12955,18895) | 429 (362.8,524.3) | 36487 (27498,49295) | 365.3 (275.4,494.9) | 6.6 (-14.3,32) |
| Grenada | 415 (381,449) | 578 (530.8,628.3) | 708 (615,796) | 651.8 (571.6,729.8) | -10.3 (-28.1,8.2) |
| Jamaica | 14126 (13263,14957) | 775.3 (728.9,820.2) | 19792 (15779,25222) | 622.4 (495.2,795.6) | 38.1 (20,57.6) |
| Puerto Rico | 9924 (9444,10402) | 280 (266.4,293.1) | 15898 (13395,18270) | 218.6 (185.7,251.6) | -15.4 (-27.9,2.7) |
| Saint Kitts and Nevis | 214 (200,227) | 590.2 (551.5,625.4) | 353 (292,414) | 560.8 (472.4,643.7) | 126.1 (96.6,154.7) |
| Saint Lucia | 662 (623,705) | 826.9 (781.2,876.3) | 1348 (1128,1588) | 573.6 (480.5,673.9) | -11.8 (-29.2,10.7) |
| Saint Vincent and the Grenadines | 593 (553,632) | 856.6 (796.2,911.7) | 1415 (1249,1595) | 1043.8 (927.6,1173.1) | -14.8 (-36.6,10.2) |
| United States Virgin Islands | 453 (364,582) | 570 (465.2,720) | 517 (386,685) | 310.4 (232.8,413.7) | 12.8 (-3.1,29.3) |
| Central Asia | 147720 (130145,166387) | 323.9 (284.1,368.1) | 283348 (241620,334738) | 376.1 (320.9,441.9) | -22.9 (-41.5,-0.2) |
| Georgia | 16253 (13542,19290) | 267.6 (223.1,317.1) | 47086 (41242,53133) | 759.2 (664.7,856.7) | -22.1 (-42.4,18) |
| Mongolia | 2744 (1715,4033) | 273.9 (170.8,401.7) | 3505 (2406,4943) | 167.9 (114.3,236.2) | -19.7 (-37.2,1.8) |
| Turkmenistan | 6462 (4694,8324) | 350.3 (254.3,452.5) | 16284 (11701,23606) | 421.9 (305.9,605.6) | -21.9 (-34,-9) |
| Central Europe | 591357 (566986,618798) | 413.1 (394.8,432.6) | 956691 (873567,1033375) | 418.1 (380.5,451.9) | -5 (-20,10.4) |
| Bosnia and Herzegovina | 9550 (7581,12239) | 257.6 (207.3,328.8) | 14545 (10097,19093) | 228.9 (157.8,299.7) | -30.6 (-41.7,-17.4) |
| Hungary | 74338 (69148,79637) | 523.6 (487.2,560.2) | 96907 (86183,106011) | 479 (429.4,525.3) | 21.8 (6.8,39) |
| Montenegro | 946 (749,1259) | 157.9 (125.3,209.8) | 1735 (1309,2282) | 192.2 (145.6,251.4) | -21.5 (-40.9,2.8) |
| North Macedonia | 10466 (8448,13285) | 636.8 (512.8,795.6) | 16668 (12388,21945) | 635.9 (471.8,823.3) | -48.2 (-59.8,-35.5) |
| Romania | 186463 (176034,196147) | 720.6 (681,756.9) | 227932 (203667,254481) | 577.3 (516.8,646.6) | -45.5 (-60.9,-23.6) |
| Central Latin America | 246746 (237068,254869) | 323.9 (308.8,335.8) | 400713 (340191,468740) | 165.7 (141,193.2) | 16.1 (-5.1,40.5) |
| Central Sub-Saharan Africa | 271363 (146935,388906) | 1326.9 (764.1,1844.6) | 576693 (363693,812010) | 1213.1 (774,1694.9) | -24.7 (-46.4,6.6) |
| Central African Republic | 17025 (6063,26668) | 1655.3 (635,2491.4) | 27868 (11814,44223) | 1433.9 (628.5,2179.1) | -27.1 (-54.1,17.1) |
| Democratic Republic of the Congo | 166707 (76872,246496) | 1173.1 (572.2,1702.3) | 381139 (226561,557194) | 1208.8 (725.4,1754.2) | 183.7 (127.7,261) |
| East Asia | 5094621 (3390117,6059526) | 704.8 (483.2,825.2) | 5816085 (4174234,7400720) | 292.6 (210.4,371.8) | -21.9 (-39.3,1.1) |
| Democratic People's Republic of Korea | 68064 (40911,97438) | 504.5 (315.8,705.2) | 142229 (101840,187748) | 465.3 (336.7,611.3) | 43.9 (15.7,79.3) |
| Taiwan (Province of China) | 55225 (52722,57861) | 404.9 (381.2,424.2) | 84569 (75170,91762) | 198 (177.8,214.3) | -38.7 (-64,5.3) |
| Eastern Europe | 278145 (263673,291845) | 101.6 (96.2,106.6) | 486144 (446186,530864) | 139.7 (128.2,152.8) | -28.1 (-54.3,16.4) |
| Republic of Moldova | 4959 (4540,5376) | 114.1 (104.5,123.2) | 33402 (29662,37575) | 552.9 (491.9,622.3) | 20.4 (-16.1,72.7) |
| Russian Federation | 161055 (156323,165959) | 90.8 (88.1,93.6) | 283980 (262212,304773) | 120.2 (111.1,129) | 32.5 (-9.6,90.6) |
| Ukraine | 86567 (77959,95805) | 123.4 (111,136) | 107636 (79815,141420) | 141.8 (104.1,186.1) | 1.2 (-8.1,9) |
| Eastern Sub-Saharan Africa | 813363 (498148,1044829) | 1151.6 (741.1,1457.2) | 1226566 (838034,1554382) | 786.2 (549.7,992.2) | -34 (-58.5,11.2) |
| United Republic of Tanzania | 95671 (49319,129926) | 974.1 (510.2,1296.2) | 161375 (77636,229768) | 702.4 (325.8,998.4) | -11.2 (-40.8,29.3) |
| High-income Asia Pacific | 285967 (256365,304070) | 159.1 (141.7,169.8) | 271542 (221083,316519) | 47.1 (40.2,56.3) | 140.9 (103.4,188.5) |
| Brunei Darussalam | 359 (267,438) | 369 (281.4,446.7) | 623 (508,808) | 223.3 (182.3,284.7) | -56.4 (-63,-49.5) |
| Japan | 210802 (190712,222341) | 137.7 (123.1,145.9) | 193598 (156853,218427) | 40.9 (35.5,44.6) | 98.3 (73.3,124) |
| Republic of Korea | 68498 (42624,79448) | 284.2 (192.2,332.7) | 66592 (51734,107310) | 75.4 (58.5,119.5) | -8.5 (-18.5,2.5) |
| High-income North America | 515673 (485611,535500) | 152.1 (144,157.8) | 1404762 (1266197,1545161) | 236.5 (214.9,259.5) | 21.7 (-14.9,74.6) |
| Canada | 12292 (11465,13047) | 38.7 (36.2,41) | 41475 (37569,45051) | 61.4 (56.8,66.2) | -0.1 (-27.7,41.4) |
| Greenland | 91 (75,115) | 237.4 (195,302.8) | 99 (75,124) | 146 (113.1,180.7) | -5.6 (-14,3.6) |
| United States of America | 503279 (473963,522726) | 164 (155.3,170.1) | 1363166 (1227012,1502150) | 257.6 (233.7,283) | -19.9 (-28.8,-9.8) |
| North Africa and Middle East | 1560227 (1166916,1870658) | 1025.9 (781.2,1223.5) | 2854430 (2223168,3367199) | 692.1 (549,810.5) | -32 (-51.4,-6.5) |
| Palestine | 7863 (5435,11165) | 1011.4 (701.1,1420.2) | 12557 (10160,15312) | 615.1 (500.9,736) | -5.5 (-44.7,46.9) |
| Syrian Arab Republic | 41496 (31062,53560) | 859.1 (648.5,1082.7) | 69650 (50603,96766) | 629.7 (467.3,857) | -20.5 (-30.9,-10.5) |
| United Arab Emirates | 2710 (1944,3861) | 694.3 (493.7,932) | 14049 (10555,18484) | 545.4 (424.6,681) | -48.8 (-56.5,-40.8) |
| Oceania | 17829 (10921,24839) | 583.6 (371.2,789.7) | 35086 (23181,51353) | 434.6 (296.5,627.6) | -75.5 (-79.6,-70.8) |
| American Samoa | 94 (69,114) | 407.8 (297.3,483.9) | 142 (115,179) | 293.5 (236.7,367.1) | -29.2 (-37.1,-21.7) |
| Cook Islands | 282 (225,352) | 2274.6 (1843.7,2825.1) | 297 (236,371) | 1184.9 (935.3,1495.6) | -33.3 (-48.2,-15) |
| Guam | 658 (416,799) | 899.7 (557.6,1056.8) | 596 (489,853) | 289.5 (238.7,415.3) | -47.8 (-54.4,-40.4) |
| Northern Mariana Islands | 36 (25,48) | 183.5 (134.8,234.6) | 75 (60,87) | 145.3 (116.5,168.7) | 12.5 (-9.8,41.7) |
| Solomon Islands | 781 (389,1266) | 563.9 (300,875.3) | 1780 (1092,2644) | 483 (305.3,700.2) | -38.9 (-52.3,-27.7) |
| Tokelau | 10 (7,13) | 774.8 (527.7,1047.2) | 7 (4,9) | 460.6 (300.8,624.5) | -27.1 (-41,-11.6) |
| Tuvalu | 65 (37,90) | 996.4 (572.4,1341.3) | 64 (43,85) | 626.3 (423.1,819.5) | 21.8 (-1.8,43.5) |
| South Asia | 1817086 (1179625,2449771) | 345.1 (226.3,463.8) | 4123050 (3221046,5404918) | 301.7 (238.1,392.3) | -39.8 (-53.4,-22.9) |
| Southeast Asia | 1514602 (1050905,1853653) | 611.5 (426.6,743.7) | 2960770 (2139789,3454908) | 464.8 (339.7,536.9) | -8.6 (-31,33.4) |
| Lao People's Democratic Republic | 24561 (9884,37506) | 1208.3 (507.1,1800.1) | 30719 (19174,41815) | 690.3 (427.3,933.1) | -27.8 (-49.8,16.8) |
| Malaysia | 17428 (12081,20968) | 192.4 (132.4,231.6) | 32784 (27717,38925) | 118.9 (101.2,140.7) | -13.4 (-33.1,15) |
| Southern Latin America | 129092 (122998,134161) | 289.4 (274.8,301.4) | 191779 (173311,203910) | 213.2 (193.3,226.4) | -29.1 (-47.3,7.7) |
| Southern Sub-Saharan Africa | 202984 (179665,249010) | 758.9 (665,944.8) | 483470 (427107,568062) | 889.1 (785.1,1034.2) | 3 (-27.2,60.9) |
| Tropical Latin America | 401304 (385288,413173) | 455 (431.3,470.2) | 593759 (541161,645012) | 234.8 (213.3,255.2) | -47.1 (-66.8,-1.2) |
| Western Europe | 800560 (740059,838840) | 134.9 (124.6,141.2) | 1301048 (1089766,1426440) | 111.1 (95.1,120.7) | -22.3 (-47.2,16.1) |
| Ireland | 2135 (1982,2267) | 54.6 (50.5,58) | 2669 (2270,2951) | 32 (27.5,35.4) | -58.5 (-68,-37.8) |
| Western Sub-Saharan Africa | 614136 (448612,771928) | 712.7 (522.3,889.5) | 1166017 (722290,1453815) | 590.8 (378.8,726.1) | -59.1 (-68.9,-38.3) |
| Burkina Faso | 32266 (21091,44505) | 761.7 (502.6,1037) | 77315 (42901,114268) | 854.9 (485.7,1240.3) | -7.8 (-36.7,64.9) |
| Republic of Chile | 22505 (21465,23619) | 239.9 (228,252.5) | 1406681 (886661,1835952) | 632.3 (405.4,817.6) | -51.1 (-55.3,-47.3) |
| State of Kuwait | 4416 (4080,4779) | 771.7 (693.9,836.5) | 72758 (56726,91612) | 68.3 (53.3,86) | 37.5 (25.3,50.4) |
| Republic of Yemen | 68038 (37406,97753) | 1535.4 (892.7,2190.9) | 259 (169,350) | 340 (228.6,449.4) | -77.1 (-82.4,-69.4) |
| Principality of Andorra | 81 (52,118) | 169.9 (110.1,250.7) | 57121 (49790,64213) | 287.4 (250.7,323.4) | 274.6 (212.7,351.1) |
| Republic of Ecuador | 21980 (20928,23074) | 431.3 (409.8,453.4) | 34919 (30705,39155) | 207.3 (149,270.1) | 391.7 (323.1,471.5) |
| Republic of Italy | 184295 (167527,194486) | 206.1 (186.6,217.8) | 19682 (14135,25741) | 292.5 (208.2,374.2) | 147 (101.9,218.8) |
| Commonwealth of Dominica | 601 (514,693) | 1030.5 (887.5,1187.9) | 5589287 (3973271,7160215) | 761.6 (484.9,1033.3) | 384.6 (319.2,462.4) |
| Togolese Republic | 8790 (5891,12156) | 998.9 (402.1,1478.5) | 50629 (24116,77835) | 31.9 (28,34.8) | 32.4 (22.6,42.6) |
| Republic of the Union of Myanmar | 228041 (85900,340404) | 760 (509.6,963.8) | 578 (365,804) | 27.4 (23.4,30) | 14.9 (-15.7,51.8) |
| Independent State of Samoa | 637 (425,815) | 164.6 (155.1,173.1) | 4182 (3648,4572) | 786.8 (473.8,1208.6) | -31.7 (-43.7,-10.2) |
| Eastern Republic of Uruguay | 6429 (6060,6769) | 696.6 (480.2,960.5) | 3287 (2736,3615) | 547.2 (214.2,825.8) | -32 (-54.3,202.2) |
| Republic of Tajikistan | 21396 (14833,29865) | 822.7 (562.9,1168.7) | 3560 (2114,5433) | 784.2 (424,1133.6) | -34.6 (-54.7,0.2) |
| Republic of Madagascar | 85731 (58534,112010) | 1710.8 (1185.8,2203.5) | 44542 (17660,68324) | 344.1 (289.6,409.5) | -32.2 (-51.5,-2.8) |
| Republic of the Congo | 18169 (9636,26811) | 1856.2 (1060.1,2670.4) | 49 (26,72) | 80.3 (69.1,91.9) | -33.7 (-51.8,-1) |
| Lebanese Republic | 10775 (4089,16256) | 552.1 (217.5,832) | 15591 (13021,18651) | 391.7 (294.9,502.3) | -56.1 (-69.2,-26.6) |
| Kingdom of Lesotho | 8514 (6065,11425) | 1070.7 (766.9,1431.9) | 7911 (6766,9105) | 927.3 (526.7,1308.3) | 3.5 (-18.3,27.3) |
| Republic of Malawi | 29248 (13297,40444) | 818.8 (408.1,1117) | 2354 (1733,3145) | 761.6 (362.1,1123.1) | -11.8 (-36.5,20.4) |
| Republic of the Philippines | 208671 (184716,242040) | 730.4 (649.3,855.9) | 171574 (94256,255973) | 548.4 (377.2,725.4) | -4.9 (-28.5,31.6) |
| Republic of Poland | 110534 (106210,113713) | 260.6 (249.4,268.3) | 27500 (12837,41650) | 369.5 (270.4,507.1) | 3.3 (-26.9,36.5) |
| State of Libya | 10755 (6432,15702) | 585.2 (357.8,839.1) | 1005 (691,1346) | 61.9 (52.7,68.8) | -55.1 (-70.5,-17.1) |
| Republic of Mozambique | 66374 (42546,86945) | 1240.3 (832,1583.7) | 77033 (56095,105728) | 86.8 (71.5,96) | -28.4 (-47.8,2.1) |
| Republic of Peru | 20276 (16663,24350) | 171.9 (140.4,205) | 118111 (98054,132719) | 1113.3 (681.3,1527.6) | -25.4 (-44.8,6.4) |
| Republic of the Gambia | 2338 (1638,3224) | 670.4 (477.5,921.8) | 113714 (91511,126696) | 167.3 (134.1,205.9) | -20.7 (-45.7,24.3) |
| Portuguese Republic | 15622 (14540,16607) | 124.8 (114.7,132.7) | 69552 (37233,99623) | 936.5 (567.8,1356.7) | -27.9 (-46.2,2.7) |
| Federative Republic of Brazil | 393990 (378605,405804) | 457.8 (433.7,473.4) | 538 (425,673) | 738.9 (618.4,869.2) | -20.4 (-44.8,10.1) |
| Republic of Slovenia | 7120 (6673,7561) | 290 (272.6,306.9) | 173056 (102963,255186) | 266.8 (153.4,380) | -70.4 (-72.8,-62) |
| Republic of Serbia | 49770 (38839,65165) | 570.7 (449.7,738) | 597858 (494705,708972) | 360.3 (282,459.4) | -39.5 (-54.7,-14.9) |
| Republic of Finland | 7660 (7065,8189) | 107 (98.4,113.9) | 22182 (12523,31886) | 279.9 (223.9,352.8) | -70.3 (-71.7,-69) |
| People's Democratic Republic of Algeria | 107726 (73661,152284) | 1080 (730.5,1444.9) | 103765 (80607,132906) | 1509 (999.7,2082.2) | -73.5 (-80,-41.3) |
| Republic of El Salvador | 4167 (3511,4818) | 142.6 (120.5,167.2) | 7211 (5802,9110) | 433.5 (334.1,554.4) | -57.7 (-61.5,-54.4) |
| Socialist Republic of Viet Nam | 237132 (155824,331695) | 616.7 (413.7,850.4) | 164136 (106340,227693) | 370.4 (287.9,487.4) | 55.4 (42.1,69.4) |
| State of Qatar | 444 (362,543) | 564.5 (458.3,688.2) | 1923 (1501,2464) | 1207.5 (705.7,2037.5) | 58.6 (49.9,67.9) |
| People's Republic of China | 4971332 (3273139,5916486) | 716 (488.7,841.4) | 417 (318,560) | 34.5 (30.1,37.7) | -38.5 (-57.2,-17.3) |
| Republic of Burundi | 23012 (2429,37421) | 1044 (115.1,1664.8) | 154624 (87035,250892) | 229.7 (209.4,244.6) | 57.1 (43.2,71.6) |
| Republic of Honduras | 9927 (8182,13360) | 501.3 (407.3,725.6) | 4722 (4031,5176) | 96.9 (76,124.5) | -32.5 (-44.6,-14.8) |
| Grand Duchy of Luxembourg | 471 (435,501) | 89.6 (83.2,95.2) | 132243 (120140,140836) | 854.3 (731.3,1004.4) | -24.8 (-43.6,6.3) |
| Republic of Armenia | 7900 (6710,9181) | 325.9 (277,377.9) | 32692 (25670,41778) | 232.7 (211.2,252.7) | -28.2 (-44.4,-5) |
| Principality of Monaco | 73 (53,105) | 94.2 (69.1,136) | 373973 (319740,442813) | 681.6 (411.9,1052.7) | -38.9 (-54.8,-13) |
| Kingdom of Cambodia | 39770 (21163,56010) | 906.7 (493.5,1269.5) | 575098 (523244,624524) | 516.2 (472.7,543.3) | -32.5 (-48.7,-13.3) |
| Republic of Mauritius | 6386 (6005,6786) | 908.2 (853.8,964.3) | 33590 (20078,53557) | 717.8 (443.5,983.5) | -33.6 (-46.8,-16.6) |
| Arab Republic of Egypt | 390734 (317265,523699) | 1697.3 (1389.8,2342.5) | 8989 (8210,9478) | 126.1 (113.4,136.6) | -19.3 (-43.8,31.7) |
| Republic of Guyana | 6412 (5824,7034) | 1724.8 (1567.9,1884.3) | 41188 (24553,57293) | 245.3 (196.3,312.9) | -39.6 (-57.6,-11.7) |
| Republic of Djibouti | 1498 (988,2091) | 1213.2 (812.7,1675.2) | 10729 (9654,11605) | 1739.3 (1494,1984.7) | -63.7 (-70.5,-55.4) |
| Republic of Croatia | 24439 (22229,26890) | 456.9 (415.4,503.5) | 10624 (8514,13530) | 721.2 (359.3,1098.5) | -63.5 (-77,-13.6) |
| Republic of Lithuania | 3537 (3045,3999) | 79.5 (68.5,90.1) | 245017 (209985,279334) | 183.9 (163,198.1) | 16.5 (-18.2,69.6) |
| Commonwealth of the Bahamas | 1902 (1757,2048) | 1202.9 (1115.2,1295.8) | 47905 (42353,51649) | 417.2 (233.6,680) | -14.7 (-36.5,12.7) |
| Republic of Uganda | 42753 (14777,69085) | 724.8 (249.4,1122) | 23390 (12756,38912) | 300.3 (193.4,462.6) | -19 (-51.4,26.5) |
| Republic of Mali | 28515 (12979,40030) | 714.5 (332.9,981.5) | 78690 (49989,121123) | 25 (20.7,31) | -39.2 (-55.4,-4.7) |
| Republic of Fiji | 2585 (2033,3235) | 689.1 (543,856) | 3817 (3170,4702) | 601.8 (314.8,846.2) | -59.9 (-70.5,-45.5) |
| Kingdom of the Netherlands | 7570 (6866,8026) | 37.4 (34,39.6) | 55312 (28601,79286) | 186.1 (143.3,244.8) | -19.1 (-46.5,18.4) |
| Republic of Azerbaijan | 24068 (18127,32799) | 515.6 (386.3,694.8) | 41 (31,55) | 159.1 (134.9,174.6) | -25.9 (-49,6.2) |
| Republic of Maldives | 351 (138,528) | 418.6 (178.5,623) | 385452 (319589,426754) | 83.3 (70.5,93.9) | -26.7 (-49.8,8.4) |
| Republic of Sudan | 111642 (62107,158466) | 1264.2 (733.9,1767.2) | 22824 (19048,25780) | 155.3 (136.5,173.4) | -17 (-49.1,52.6) |
| Republic of Chad | 19013 (10580,25381) | 682.6 (378.5,924.6) | 8667 (7597,9676) | 761.6 (566.7,962.2) | -41.1 (-58.5,-11) |
| Republic of Palau | 24 (18,32) | 241.6 (185.5,322.5) | 12916 (9266,16856) | 318.8 (215.2,413.2) | -21.5 (-48.3,7.9) |
| Republic of Estonia | 5479 (4725,6193) | 277 (238.2,314.1) | 1823 (1218,2375) | 1004.1 (870.5,1205.8) | -21.4 (-47.1,29.8) |
| Republic of Austria | 19358 (18013,20530) | 159.3 (148.3,168.8) | 1112 (965,1337) | 164.3 (125.3,215.8) | -25.5 (-42.7,1.9) |
| Independent State of Papua New Guinea | 10179 (5110,15560) | 525.6 (279.6,792.6) | 133 (101,175) | 389.9 (286.2,517.1) | -28 (-44.2,-2.7) |
| Republic of Senegal | 21051 (15515,27822) | 649.4 (485.1,854) | 96903 (70462,130773) | 154.9 (136.2,174.5) | -47.9 (-62.4,-26.6) |
| Republic of Malta | 421 (387,455) | 105.9 (96.8,114.6) | 7449 (5771,9425) | 397.5 (308.5,501.8) | -23.5 (-44.3,5.7) |
| Kingdom of Belgium | 6185 (5535,6613) | 40 (35.9,42.7) | 120729 (90411,155436) | 585.6 (444.4,740.3) | -67.8 (-75.6,-37) |
| Republic of Cyprus | 2112 (1077,2973) | 398.7 (190.3,572.2) | 51915 (34324,70273) | 671.6 (451.3,896) | -14.8 (-36.6,19.6) |
| Republic of Suriname | 1411 (1203,1603) | 560.7 (479.6,637.9) | 5356 (3413,7372) | 648.1 (421.2,884.4) | -16.2 (-36.3,8.5) |
| Republic of Guatemala | 4609 (4402,4832) | 153.9 (146.5,161.6) | 14189 (12340,16036) | 338.5 (295.5,381.7) | -23.4 (-44.4,9.8) |
| Swiss Confederation | 13199 (11525,14777) | 118.6 (103.8,132.5) | 8583 (6891,9545) | 29.8 (24.7,32.7) | -18.2 (-37.5,10) |
| Kingdom of Bahrain | 826 (691,996) | 640.6 (538.2,777.7) | 298 (248,341) | 44.4 (37.7,50.5) | -22.7 (-44.2,4.3) |
| Federal Democratic Republic of Nepal | 33615 (19692,47512) | 402.2 (245.8,561.4) | 78137 (70996,83060) | 60.4 (55.8,64) | -20.8 (-39.1,4.4) |
| Republic of Indonesia | 652144 (376783,858215) | 682.4 (398.6,898.9) | 8294 (6728,10457) | 182.9 (148.5,230.2) | -23 (-45.9,4.6) |
| Kingdom of Saudi Arabia | 62091 (38778,87991) | 1146.6 (739.8,1590.6) | 328382 (261451,398248) | 387.5 (306.1,470.2) | -20.6 (-45.9,23.2) |
| Union of the Comoros | 2226 (1115,3377) | 1203.6 (626.1,1779.7) | 247369 (177566,330242) | 600.4 (437.2,803.7) | -23.4 (-41.1,2.4) |
| Federal Republic of Germany | 321511 (297735,340428) | 244.9 (227.1,258.4) | 12936 (10640,15659) | 201.6 (165.8,244.3) | -14.3 (-38.3,26.3) |
| Republic of Uzbekistan | 33618 (24584,44477) | 294.3 (214.9,390.3) | 53023 (29514,73413) | 778.3 (438.6,1075.7) | -40.6 (-55.9,-16.9) |
| Republic of Zambia | 37911 (24951,49297) | 1398.8 (952,1771) | 43862 (23424,65915) | 741.3 (406.8,1074) | -12.7 (-38.5,20.7) |
| Hellenic Republic | 17016 (15585,17961) | 118.6 (107.6,125.1) | 29504 (16275,44117) | 80.8 (69.5,89.3) | -37.1 (-53,-7.2) |
| Republic of Iceland | 151 (135,163) | 50.3 (45.3,54) | 868 (729,963) | 863.6 (540.2,1198.2) | -10.2 (-33.3,19.4) |
| Republic of Cuba | 12868 (12217,13486) | 127.1 (120.6,133.1) | 274341 (169776,385208) | 284 (213.8,364.7) | -12.6 (-33.8,31.4) |
| Bolivarian Republic of Venezuela | 55673 (53019,58228) | 599 (564.2,627.5) | 3159586 (2355443,4063497) | 1501.8 (790.5,2264) | -28.1 (-55.5,25.5) |
| Republic of Belarus | 14152 (11340,17112) | 109.2 (87.6,131.6) | 139756 (69846,216041) | 1524.5 (835.4,2159.8) | -29.5 (-49.4,7.9) |
| Republic of Latvia | 2396 (2128,2650) | 68.8 (61.1,76.1) | 15560 (8326,22375) | 332.1 (252.4,430.9) | -10.8 (-33.6,39.8) |
| Republic of Guinea | 22845 (13813,32386) | 694.1 (422.1,985) | 18661 (14137,24329) | 936.8 (537.5,1499) | -8.1 (-36.7,49.5) |
| Republic of Bulgaria | 80445 (72497,88519) | 721.9 (653.4,790.7) | 4304 (2413,6912) | 772 (438.4,1125.2) | 4.3 (-16,34.7) |
| Kingdom of Tonga | 104 (79,137) | 188.2 (143,247.2) | 8402 (5156,11943) | 839.7 (514.5,1178.2) | -24 (-36.4,-1) |
| Kingdom of Morocco | 137558 (79023,196932) | 1011.9 (592.8,1432.7) | 6783 (4594,9942) | 160.4 (109.5,232.7) | -30.4 (-51.5,5) |
| Islamic Republic of Afghanistan | 130958 (54852,214070) | 1996.9 (899.2,3200.3) | 32058 (19233,46695) | 1315.7 (814.9,1854.3) | -7.4 (-27.7,26.2) |
| Republic of South Africa | 151616 (130825,191597) | 724.7 (620.4,933.6) | 13010 (8607,18168) | 1040.9 (713.2,1435.2) | -42.9 (-61.7,22.5) |
| Republic of Vanuatu | 392 (234,591) | 78.1 (73.6,82.4) | 115841 (70734,159409) | 687.9 (430.5,937.2) | -38.2 (-51.3,-11.5) |
| Republic of Niue | 14 (10,18) | 610.5 (373.8,880.9) | 29032 (23222,35564) | 173.9 (140.6,211.2) | -60 (-74.2,1.8) |
| Republic of San Marino | 45 (36,62) | 641.8 (438.9,825.9) | 12504 (10390,14217) | 230.6 (193.7,262) | -43.2 (-48,-38.6) |
| Democratic Socialist Republic of Sri Lanka | 63057 (49500,73411) | 121.8 (96.7,167.8) | 737 (597,920) | 908.9 (738.2,1122.2) | -42.4 (-58.8,-4.6) |
| Republic of Ghana | 54916 (37499,71491) | 623.4 (482,725.2) | 383838 (314586,423770) | 198.1 (166.6,216.7) | 1.2 (-15.9,21) |
| Republic of Colombia | 84386 (80302,87982) | 861.5 (607.4,1120.8) | 32525 (25545,41376) | 213.4 (168.7,269.1) | -41.1 (-50.6,-28.2) |
| Republic of Guinea-Bissau | 4562 (2217,6789) | 521.5 (493.2,545.3) | 72 (50,98) | 73 (49.9,101.9) | -51.8 (-70.2,-12.8) |
| Federal Republic of Nigeria | 301600 (214949,395417) | 1114 (570.9,1623.1) | 282 (173,409) | 751.1 (467.9,1055) | -28 (-47.1,7.4) |
| Republic of Paraguay | 7314 (6245,8844) | 705.6 (510,918.9) | 1559 (1142,2238) | 226.6 (169.4,301.9) | -18.4 (-44.8,39.3) |
| Slovak Republic | 12902 (9545,18737) | 338.4 (289,407) | 126710 (77388,187035) | 1281.4 (819.4,1836) | -33.5 (-53.9,-2) |
| Republic of Haiti | 28710 (11623,45810) | 219.2 (162.2,319) | 11498 (9998,13138) | 196.5 (170.7,224.5) | -26.3 (-31.1,-22.2) |
| People's Republic of Bangladesh | 208172 (138543,293340) | 474.1 (320.7,669) | 180 (129,242) | 103.1 (73.3,139.7) | 13.9 (6,21.4) |
| Kingdom of Thailand | 31811 (22326,40702) | 94.9 (67.5,121.2) | 462146 (330161,621472) | 421 (304.7,575.2) | -28.5 (-33.3,-23.8) |
| Republic of Seychelles | 963 (822,1073) | 1706.2 (1455.3,1901.3) | 31731 (16813,43876) | 710.1 (366.4,975.3) | -23.3 (-30.2,-17.5) |
| Republic of Namibia | 6902 (5276,9111) | 1159.4 (879.2,1522) | 6990 (3632,10859) | 1290.2 (685.4,1912.5) | 17.2 (3.8,31.9) |
| Kingdom of Norway | 3139 (2842,3318) | 42.5 (38.7,44.8) | 8388 (4452,12360) | 1087.6 (584.8,1571.7) | -29 (-52.5,14) |
| Republic of Equatorial Guinea | 3188 (1857,4733) | 1771 (1067.7,2566.2) | 6176 (4905,7839) | 95.2 (75.5,121) | -3.9 (-34.7,38.2) |
| Kingdom of Eswatini | 3644 (2304,4783) | 1342.6 (860.3,1756.1) | 230802 (149618,310699) | 775.3 (509.1,1028.1) | 42.4 (-12.8,112.2) |
| Republic of India | 1363016 (850010,1939926) | 318.2 (198.6,453) | 26428 (22079,29383) | 86 (73.6,94.9) | -10.2 (-39.5,32.9) |
| Kingdom of Denmark | 3629 (3325,3905) | 42.8 (39.3,45.9) | 76199 (32404,120809) | 574.8 (233.3,913.4) | 17.9 (3.1,33.2) |
| Republic of Cameroon | 33800 (17417,45610) | 761.4 (390.1,1022.8) | 26829 (22203,29681) | 183.2 (156.1,200.3) | 32.1 (-10.9,76.8) |
| Republic of Singapore | 6307 (5996,6614) | 298.1 (281.8,312.7) | 4521 (2884,6981) | 823.2 (534.1,1229.1) | -48.4 (-52.2,-44.7) |
| Islamic Republic of Iran | 166924 (139207,214500) | 745.6 (616.4,982) | 86792 (41806,128437) | 669.8 (321.9,976.9) | -49.2 (-53,-45.5) |
| Republic of Zimbabwe | 26400 (21266,33775) | 727 (583.5,934.2) | 436556 (256322,582964) | 482.8 (296.3,635.4) | -1.9 (-25.8,27.5) |
| Republic of Liberia | 8807 (6046,11783) | 771.6 (539,1016.5) | 11 (7,14) | 496.2 (326.1,642.5) | -17.6 (-24,-13.3) |
| Gabonese Republic | 8001 (5144,11025) | 1498.8 (981.7,2039.1) | 32990 (26280,43723) | 564 (451.6,754.5) | -39.4 (-61.4,0.8) |
| Kingdom of Sweden | 5050 (4545,5509) | 30.5 (27.5,33.2) | 617723 (491897,778557) | 1145.8 (932.2,1485.9) | -4.9 (-12.7,1.4) |
| Republic of Albania | 4366 (3178,5911) | 243.1 (177.5,324.7) | 73435 (45646,99542) | 631.4 (392.2,834.5) | -25.7 (-31.5,-19.8) |
| Republic of the Niger | 16456 (6970,24400) | 608.4 (261.9,905.1) | 270308 (141519,395142) | 575.3 (305.5,831.1) | -55.1 (-70.1,-6.2) |
| Republic of Tunisia | 34028 (23780,44866) | 772.1 (546.3,1004) | 5185 (4244,6485) | 1282.2 (1053.7,1589.9) | -25.4 (-33.3,-18.1) |
| Sultanate of Oman | 5949 (3977,8585) | 925.6 (404,1458.2) | 8477 (6561,10748) | 1330.6 (1042.4,1655.1) | 71.3 (55.8,86) |
| Kingdom of Bhutan | 968 (506,1399) | 940 (646,1361.8) | 377413 (243730,557095) | 410.4 (269,590.4) | -38.9 (-44.4,-33.5) |
| Czech Republic | 10560 (9941,11148) | 452.3 (233.2,661.1) | 31620 (27248,35555) | 1037.8 (900.2,1168.8) | -35.1 (-41.6,-30.3) |
| Republic of Nauru | 47 (23,68) | 959.2 (493.2,1349.8) | 3954 (2899,5284) | 527 (400.8,686.7) | 8.8 (1.6,16.2) |
| Republic of South Sudan | 24760 (12095,38000) | 1020.9 (521.5,1519.9) | 2762 (2097,3611) | 440.2 (334.6,570.6) | -11.6 (-21.7,-0.3) |
| Democratic Republic of Timor-Leste | 2096 (1078,3001) | 793.9 (426.4,1126.9) | 62957 (42020,90041) | 960.2 (661,1351.3) | -41.3 (-48.1,-35.9) |
| Republic of Kazakhstan | 28297 (25647,31015) | 222.7 (201.9,244.7) | 118 (85,152) | 101.5 (73.9,130.5) | -58.7 (-62.3,-55.7) |
| United Mexican States | 79022 (76158,81234) | 215.4 (205.9,221.6) | 18946 (10406,27836) | 841.9 (480.2,1233.5) | -3.9 (-10.7,1.5) |
| Republic of Angola | 58274 (34695,81626) | 1633.4 (1044.1,2221.3) | 26233 (20947,29507) | 110.4 (90.8,122.8) | -21.8 (-30.1,-13.3) |
| French Republic | 89260 (80672,96308) | 101.2 (92,109) | 40271 (35042,43715) | 129.1 (115,138.7) | -23.7 (-31.7,-15.5) |
| Kingdom of Spain | 42056 (37789,44965) | 79.2 (71,84.8) | 34522 (23855,47618) | 375.7 (261.3,521) | 7.8 (-34.5,56.1) |
| Hashemite Kingdom of Jordan | 12512 (9363,16229) | 1045 (786.9,1371.7) | 422462 (265204,930838) | 340.7 (213.7,742.7) | 3.4 (-4.5,10.5) |
| Republic of Rwanda | 42778 (9169,60804) | 1589.7 (383.8,2189.2) | 34136 (28865,37449) | 151.5 (130.3,165.3) | -35.5 (-41.1,-30.5) |
| Republic of Costa Rica | 3710 (3438,3943) | 219.3 (201.9,233.6) | 71372 (59473,85304) | 127.7 (106.3,152.8) | -31.1 (-36.9,-25.5) |
| Islamic Republic of Pakistan | 211315 (134457,279293) | 403.6 (260.9,539.6) | 10795 (6658,15645) | 1164.2 (723.3,1682.4) | -40.1 (-59.7,-13.2) |
| State of Eritrea | 17472 (8708,25400) | 1699.6 (970,2302.9) | 354428 (302036,404642) | 495.3 (420.8,571.8) | 9.6 (-0.2,19.3) |
| Republic of Kiribati | 154 (102,191) | 399.1 (270.4,497) | 847 (568,1088) | 581.8 (393.5,742.7) | 172.9 (144,200) |
| Republic of Benin | 10630 (7041,14059) | 533.7 (352.1,703.9) | 29802 (20176,42226) | 591.3 (410.1,820.4) | -6.9 (-17.7,3.3) |
| Islamic Republic of Mauritania | 9496 (5956,13225) | 962.5 (615.6,1335.6) | 853 (731,957) | 70.1 (60.7,78.3) | -2.5 (-7.4,2.3) |
| Democratic Republic of Sao Tome and Principe | 250 (201,300) | 395.9 (320.3,473.9) | 11620 (10437,12495) | 187.5 (170.5,200.5) | -17.1 (-43.7,7.8) |
| United Kingdom of Great Britain and Northern Ireland | 54967 (52268,56511) | 61.9 (59,63.6) | 26615 (16745,36594) | 509.7 (326.7,678.7) | -4.5 (-37,35.4) |
| Republic of Panama | 1638 (1508,1750) | 113.4 (104.2,121.5) | 15838 (9346,24547) | 743.4 (446,1141.9) | 12.2 (-25.1,67.1) |
| Republic of Cabo Verde | 1278 (960,1673) | 557.7 (419,729.1) | 18759 (16386,21213) | 199.3 (174.7,226.2) | -22.3 (-43,4.8) |
| Federal Republic of Somalia | 34682 (17684,51379) | 1493.6 (841.3,2102.1) | 2857 (2352,3445) | 179 (144.3,214.6) | -12 (-43.9,33.3) |
| Republic of the Marshall Islands | 147 (95,207) | 896.1 (582.2,1245.4) | 155328 (119875,186057) | 131.7 (102.5,156.8) | 8.6 (-25.8,48.9) |
| Republic of Botswana | 5907 (4236,8015) | 1160.1 (848.2,1568.6) | 40062 (31037,50647) | 631.5 (490.8,792.3) | 6.1 (-32.4,83.3) |
| Kyrgyz Republic | 6984 (5979,8125) | 239.1 (205.2,280.4) | 38850 (11976,55907) | 714.2 (221.1,1044.3) | 25.3 (-16.3,87.1) |
| Republic of Kenya | 50601 (37992,61751) | 659.3 (497.7,809.4) | 79469 (42862,132889) | 641 (350,1077.6) | -20.2 (-48.7,23.7) |
| Republic of Turkey | 195520 (132162,260816) | 658.1 (453.4,856.9) | 120530 (79365,171997) | 1178.8 (793.2,1610.8) | 3.4 (-35.5,62.4) |
| Republic of Trinidad and Tobago | 6032 (5707,6332) | 767.7 (724.7,803.3) | 26672 (16000,39016) | 1126.4 (708.6,1569.5) | -2.4 (-32.6,40.2) |
| Republic of Iraq | 56407 (35213,75400) | 725.4 (455.2,974) | 15240 (12909,16730) | 38.7 (33.1,42.3) | 9.1 (-31.9,71.2) |
| Federal Democratic Republic of Ethiopia | 258067 (140730,340615) | 1367.1 (795.1,1779.3) | 78773 (46914,111365) | 682.6 (430.2,950.9) | -15.8 (-40.6,17.8) |
| Federated States of Micronesia | 482 (291,665) | 994 (613.3,1353.9) | 23875 (13127,34085) | 614.2 (350.5,855.9) | -22.8 (-51.2,24.5) |
| State of Israel | 3896 (3598,4144) | 83.7 (76.7,89) | 6210 (4937,7329) | 138.2 (109.6,163.1) | -10.1 (-41.5,73.7) |
| Argentine Republic | 100152 (94926,104772) | 321.1 (303.9,335.8) | 180651 (160601,197673) | 246 (219.1,269.9) | -31.6 (-58.1,-6.6) |
| Republic of Côte d'Ivoire | 25748 (15136,34323) | 643.6 (372.4,846.7) | 59347 (28541,91889) | 1069.5 (529.3,1587.9) | -6.4 (-30.1,26) |
| Republic of Sierra Leone | 11756 (7643,16026) | 578.6 (381,776.7) | 134952 (91479,172727) | 682.6 (454.8,875.1) | 3.4 (-27,44.9) |
| Plurinational State of Bolivia | 11078 (4817,16173) | 371.3 (164.6,537) | 66664 (50142,83374) | 388 (290.8,483.9) | 6.2 (-28.2,48.2) |
| Republic of Nicaragua | 3614 (3176,4284) | 250.9 (219.8,302.4) | 10980 (8204,15464) | 823.7 (633.5,1105.6) | 10.8 (-25.6,57.1) |

Table S4 APC model data for the predicted trends in the age-standardized prevalence, age-standardized mortality rate and DALYs for hypertensive heart disease (HHD) globally by sex 2022-2046

| year | sex | Age-standardized prevalence rate | Numer of prevalence cases | Age-standardized deaths rate | Numer of deaths cases | Age-standardized DALYs rate | Numer of DALYs cases |
| --- | --- | --- | --- | --- | --- | --- | --- |
| 2022 | Female | 150.98 | 7205598.738 | 16.92 | 811126.6298 | 303.63 | 14411735.34 |
| 2023 | Female | 152.04 | 7463312.112 | 16.81 | 830069.7809 | 302.03 | 14728848.39 |
| 2024 | Female | 153.1 | 7738355.483 | 16.71 | 851867.956 | 300.44 | 15072730.12 |
| 2025 | Female | 153.79 | 8006976.345 | 16.6 | 874592.7607 | 298.92 | 15431964.91 |
| 2026 | Female | 154.48 | 8282389.202 | 16.49 | 897160.9414 | 297.39 | 15790264.28 |
| 2027 | Female | 155.18 | 8560913.319 | 16.38 | 918770.5212 | 295.87 | 16138996.04 |
| 2028 | Female | 155.87 | 8850838.249 | 16.27 | 941434.0931 | 294.34 | 16496568.77 |
| 2029 | Female | 156.57 | 9158737.409 | 16.17 | 966522.1176 | 292.82 | 16875994.98 |
| 2030 | Female | 156.86 | 9455287.99 | 16.07 | 993594.9641 | 291.5 | 17278281.12 |
| 2031 | Female | 157.15 | 9758094.345 | 15.98 | 1020964.866 | 290.17 | 17680803.1 |
| 2032 | Female | 157.45 | 10062382.76 | 15.88 | 1047855.318 | 288.85 | 18075152.37 |
| 2033 | Female | 157.74 | 10376477.14 | 15.78 | 1075804.869 | 287.52 | 18475861.93 |
| 2034 | Female | 158.04 | 10706744.05 | 15.69 | 1105925.686 | 286.2 | 18894150.74 |
| 2035 | Female | 157.95 | 11019474.9 | 15.61 | 1138649.726 | 285.13 | 19338072.85 |
| 2036 | Female | 157.87 | 11331998 | 15.54 | 1171468.698 | 284.07 | 19778246.84 |
| 2037 | Female | 157.78 | 11637501.53 | 15.46 | 1203491.344 | 283.01 | 20205479.1 |
| 2038 | Female | 157.69 | 11942972.08 | 15.39 | 1236130.272 | 281.95 | 20633052.17 |
| 2039 | Female | 157.61 | 12255941.83 | 15.32 | 1270476.216 | 280.89 | 21072106.75 |
| 2040 | Female | 157.5 | 12568031.87 | 15.26 | 1306933.679 | 280.09 | 21534071.77 |
| 2041 | Female | 157.39 | 12875957.1 | 15.2 | 1342969.584 | 279.29 | 21987608.06 |
| 2042 | Female | 157.29 | 13174781.52 | 15.14 | 1377673.093 | 278.49 | 22424252.17 |
| 2043 | Female | 157.18 | 13469892.83 | 15.09 | 1412494.696 | 277.69 | 22857425.09 |
| 2044 | Female | 157.08 | 13767856.36 | 15.03 | 1448518.351 | 276.89 | 23297644.84 |
| 2045 | Female | 156.97 | 14066021.24 | 14.97 | 1484602.02 | 276.09 | 23734568.68 |
| 2046 | Female | 156.87 | 14361900.15 | 14.92 | 1519795.146 | 275.29 | 24159368.33 |
| 2022 | Male | 149.63 | 5916383.639 | 16.51 | 601172.8298 | 310.65 | 12374406.51 |
| 2023 | Male | 150.3 | 6112298.534 | 16.43 | 616772.4098 | 309.35 | 12650586.84 |
| 2024 | Male | 150.97 | 6320244.255 | 16.36 | 634416.3014 | 308.06 | 12946192.82 |
| 2025 | Male | 151.4 | 6526161.243 | 16.28 | 652926.5408 | 306.86 | 13256986.32 |
| 2026 | Male | 151.84 | 6735615.596 | 16.2 | 671283.1769 | 305.65 | 13565931.77 |
| 2027 | Male | 152.28 | 6945568.962 | 16.12 | 688845.6623 | 304.45 | 13865664.15 |
| 2028 | Male | 152.71 | 7162840.296 | 16.04 | 707062.5053 | 303.25 | 14169753.92 |
| 2029 | Male | 153.15 | 7392529.606 | 15.96 | 726971.5102 | 302.04 | 14488682.57 |
| 2030 | Male | 153.36 | 7618545.819 | 15.89 | 748130.0657 | 301.02 | 14822801.5 |
| 2031 | Male | 153.57 | 7847054.285 | 15.82 | 769372.3785 | 299.99 | 15155542.78 |
| 2032 | Male | 153.79 | 8074004.692 | 15.74 | 790107.6636 | 298.97 | 15480458.88 |
| 2033 | Male | 154 | 8305976.633 | 15.67 | 811546.1104 | 297.94 | 15809263.4 |
| 2034 | Male | 154.21 | 8548396.962 | 15.6 | 834553.7205 | 296.91 | 16151056.85 |
| 2035 | Male | 154.22 | 8784013.16 | 15.54 | 858919.8366 | 296.09 | 16508047.1 |
| 2036 | Male | 154.23 | 9018392.903 | 15.49 | 883198.4557 | 295.27 | 16861188.16 |
| 2037 | Male | 154.24 | 9246580.902 | 15.43 | 906717.1793 | 294.45 | 17203291.1 |
| 2038 | Male | 154.24 | 9473860.164 | 15.37 | 930625.3612 | 293.63 | 17545288.14 |
| 2039 | Male | 154.25 | 9706156.75 | 15.31 | 955801.4408 | 292.81 | 17896411.54 |
| 2040 | Male | 154.26 | 9939994.952 | 15.27 | 982169.2547 | 292.21 | 18262419.43 |
| 2041 | Male | 154.28 | 10171213.6 | 15.22 | 1008189.612 | 291.6 | 18622136.02 |
| 2042 | Male | 154.29 | 10396497.51 | 15.18 | 1033192.516 | 290.99 | 18968931.98 |
| 2043 | Male | 154.3 | 10619701 | 15.13 | 1058148.386 | 290.38 | 19311956.16 |
| 2044 | Male | 154.31 | 10845457.12 | 15.09 | 1083941.896 | 289.77 | 19660035.58 |
| 2045 | Male | 154.33 | 11071738.7 | 15.04 | 1109929.23 | 289.16 | 20006959.24 |
| 2046 | Male | 154.34 | 11296685.17 | 14.99 | 1135558.744 | 288.55 | 20347288.09 |

Table S5 Age-standardized prevalence and burden of hypertensive heart disease (HHD) worldwide (1990–2021) and frontier analysis results

|  | | | | | |
| --- | --- | --- | --- | --- | --- |
|  | location | year | ASR | SDI | frontier |
| 1 | Afghanistan | 1990 | 260.7882093 | 0.173832165 | 245.5041638 |
| 2 | Afghanistan | 1992 | 258.7317303 | 0.179633724 | 238.7663549 |
| 3 | Afghanistan | 1993 | 257.9990697 | 0.180183713 | 238.7663549 |
| 4 | Afghanistan | 1995 | 257.0817581 | 0.178279128 | 238.7663549 |
| 5 | Afghanistan | 1996 | 256.8990216 | 0.178085404 | 238.7663549 |
| 6 | Afghanistan | 1997 | 256.7964854 | 0.177884124 | 238.7663549 |
| 7 | Afghanistan | 1999 | 256.7144031 | 0.177075239 | 239.0367772 |
| 8 | Afghanistan | 2000 | 256.6832755 | 0.177025772 | 239.0367772 |
| 9 | Afghanistan | 2001 | 256.8295627 | 0.177773144 | 238.7663549 |
| 10 | Afghanistan | 2008 | 258.4255402 | 0.228089171 | 101.1599627 |
| 11 | Afghanistan | 2009 | 258.2039682 | 0.237900824 | 101.1599627 |
| 12 | Afghanistan | 2010 | 258.0159164 | 0.247759949 | 100.8275782 |
| 13 | Afghanistan | 2011 | 257.6351359 | 0.257041676 | 100.8275782 |
| 14 | Afghanistan | 2013 | 256.2155873 | 0.275636558 | 100.8275782 |
| 15 | Afghanistan | 2015 | 255.0434224 | 0.291849505 | 100.8275782 |
| 16 | Afghanistan | 2016 | 254.785459 | 0.299630696 | 100.8275782 |
| 17 | Afghanistan | 2018 | 254.2287226 | 0.314866093 | 67.17014806 |
| 18 | Afghanistan | 2019 | 253.9504015 | 0.322454047 | 66.46502371 |
| 19 | Afghanistan | 2020 | 253.6344115 | 0.329830068 | 65.82455226 |
| 20 | Afghanistan | 2021 | 252.5576153 | 0.337199998 | 65.82455226 |
| 21 | Albania | 1990 | 82.0439689 | 0.5577733 | 51.75814966 |
| 22 | Albania | 1991 | 82.41428837 | 0.555629412 | 51.75814966 |
| 23 | Albania | 1992 | 82.72092085 | 0.553107897 | 51.75814966 |
| 24 | Albania | 1993 | 82.96523502 | 0.552453328 | 51.75814966 |
| 25 | Albania | 1994 | 83.1372182 | 0.553763669 | 51.75814966 |
| 26 | Albania | 1995 | 83.23141541 | 0.557040889 | 51.75814966 |
| 27 | Albania | 1996 | 83.21293016 | 0.562248886 | 51.75814966 |
| 28 | Albania | 1998 | 82.87255146 | 0.572534102 | 51.75814966 |
| 29 | Albania | 1999 | 82.68998815 | 0.578295462 | 51.75814966 |
| 30 | Albania | 2001 | 82.67575659 | 0.592412947 | 51.75814966 |
| 31 | Albania | 2004 | 83.21318685 | 0.613864247 | 51.75814966 |
| 32 | Albania | 2005 | 83.27392073 | 0.620679615 | 32.04043631 |
| 33 | Albania | 2006 | 83.20281913 | 0.627485617 | 22.46369183 |
| 34 | Albania | 2007 | 83.04748311 | 0.634167969 | 22.46369183 |
| 35 | Albania | 2008 | 82.8421637 | 0.640766134 | 22.46369183 |
| 36 | Albania | 2009 | 82.62015251 | 0.64681613 | 22.46369183 |
| 37 | Albania | 2011 | 82.21939839 | 0.658196773 | 22.46369183 |
| 38 | Albania | 2012 | 81.99835499 | 0.66355678 | 22.46369183 |
| 39 | Albania | 2013 | 81.77851446 | 0.668739559 | 22.46369183 |
| 40 | Albania | 2014 | 81.5855665 | 0.674112985 | 22.03195463 |
| 41 | Albania | 2015 | 81.44547246 | 0.679668601 | 22.03195463 |
| 42 | Albania | 2016 | 81.33306201 | 0.685036648 | 17.2540694 |
| 43 | Albania | 2017 | 81.21693791 | 0.690379199 | 17.2540694 |
| 44 | Albania | 2018 | 81.11573606 | 0.695566878 | 17.2540694 |
| 45 | Albania | 2020 | 81.23072686 | 0.703790245 | 17.2540694 |
| 46 | Algeria | 1991 | 268.6539051 | 0.468319197 | 53.58976548 |
| 47 | Algeria | 1992 | 265.4819725 | 0.475950937 | 53.58976548 |
| 48 | Algeria | 1993 | 262.8839247 | 0.483255591 | 53.58976548 |
| 49 | Algeria | 1995 | 260.220577 | 0.497957543 | 53.58976548 |
| 50 | Algeria | 1996 | 260.1075725 | 0.505990967 | 53.58976548 |
| 51 | Algeria | 1997 | 260.3356684 | 0.514056566 | 53.58976548 |
| 52 | Algeria | 1998 | 260.7943112 | 0.522382419 | 53.58976548 |
| 53 | Algeria | 1999 | 261.4173284 | 0.530785583 | 53.58976548 |
| 54 | Algeria | 2003 | 267.7228979 | 0.562536956 | 51.75814966 |
| 55 | Algeria | 2006 | 275.7038698 | 0.582126362 | 51.75814966 |
| 56 | Algeria | 2008 | 282.5316093 | 0.593190973 | 51.75814966 |
| 57 | Algeria | 2010 | 287.0415115 | 0.602823535 | 51.75814966 |
| 58 | Algeria | 2012 | 288.0900088 | 0.612703533 | 51.75814966 |
| 59 | Algeria | 2014 | 288.3510728 | 0.622087292 | 32.04043631 |
| 60 | Algeria | 2015 | 288.5143822 | 0.626745568 | 32.04043631 |
| 61 | Algeria | 2016 | 288.9006698 | 0.631710736 | 22.46369183 |
| 62 | Algeria | 2017 | 289.4738894 | 0.636973176 | 22.46369183 |
| 63 | Algeria | 2018 | 290.0558203 | 0.642500039 | 22.46369183 |
| 64 | Algeria | 2019 | 290.4175608 | 0.648210785 | 22.46369183 |
| 65 | American Samoa | 1990 | 109.9233821 | 0.613633924 | 51.75814966 |
| 66 | American Samoa | 1991 | 108.4406994 | 0.615847612 | 32.04043631 |
| 67 | American Samoa | 1993 | 105.56122 | 0.622078598 | 32.04043631 |
| 68 | American Samoa | 1994 | 104.2882326 | 0.626053241 | 32.04043631 |
| 69 | American Samoa | 1995 | 103.2036772 | 0.629814054 | 22.46369183 |
| 70 | American Samoa | 1996 | 102.2172336 | 0.632706983 | 22.46369183 |
| 71 | American Samoa | 1997 | 101.1955615 | 0.634926685 | 22.46369183 |
| 72 | American Samoa | 1998 | 100.1324551 | 0.636597976 | 22.46369183 |
| 73 | American Samoa | 1999 | 99.01419409 | 0.638322193 | 22.46369183 |
| 74 | American Samoa | 2001 | 96.23327501 | 0.643214421 | 22.46369183 |
| 75 | American Samoa | 2002 | 94.1572246 | 0.646002062 | 22.46369183 |
| 76 | American Samoa | 2003 | 91.94905768 | 0.648951964 | 22.46369183 |
| 77 | American Samoa | 2007 | 86.63751306 | 0.660781176 | 22.46369183 |
| 78 | American Samoa | 2008 | 85.83338879 | 0.664025762 | 22.46369183 |
| 79 | American Samoa | 2013 | 84.73819078 | 0.685833061 | 17.2540694 |
| 80 | American Samoa | 2015 | 85.206486 | 0.697162912 | 17.2540694 |
| 81 | American Samoa | 2016 | 85.67556446 | 0.702726398 | 17.2540694 |
| 82 | American Samoa | 2017 | 86.35391059 | 0.707647909 | 17.2540694 |
| 83 | American Samoa | 2018 | 87.04738697 | 0.712800591 | 17.2540694 |
| 84 | American Samoa | 2019 | 87.55996539 | 0.717308133 | 17.2540694 |
| 85 | American Samoa | 2020 | 88.02907346 | 0.7208385 | 15.06516239 |
| 86 | Andorra | 1990 | 82.85412023 | 0.76146388 | 9.92126395 |
| 87 | Andorra | 1992 | 82.59513325 | 0.767188282 | 9.92126395 |
| 88 | Andorra | 1994 | 82.80039018 | 0.770023765 | 9.92126395 |
| 89 | Andorra | 1996 | 83.35917865 | 0.772403471 | 9.92126395 |
| 90 | Andorra | 1997 | 83.9655347 | 0.774580971 | 9.92126395 |
| 91 | Andorra | 1998 | 84.31410661 | 0.776972578 | 9.92126395 |
| 92 | Andorra | 2000 | 85.24060631 | 0.781976506 | 9.92126395 |
| 93 | Andorra | 2003 | 86.60478876 | 0.797278901 | 8.445315313 |
| 94 | Andorra | 2005 | 87.67717532 | 0.81221398 | 8.445315313 |
| 95 | Andorra | 2006 | 88.10279942 | 0.81978726 | 8.445315313 |
| 96 | Andorra | 2008 | 88.93929416 | 0.831821715 | 8.445315313 |
| 97 | Andorra | 2009 | 89.42745825 | 0.835929887 | 8.445315313 |
| 98 | Andorra | 2010 | 89.81898482 | 0.839489966 | 8.445315313 |
| 99 | Andorra | 2011 | 90.85510762 | 0.842794802 | 8.445315313 |
| 100 | Andorra | 2012 | 91.45451358 | 0.845810831 | 8.445315313 |
| 101 | Andorra | 2016 | 91.32243584 | 0.856257053 | 8.445315313 |
| 102 | Andorra | 2020 | 90.29054379 | 0.867449169 | 8.445315313 |
| 103 | Angola | 1994 | 218.5921305 | 0.284487594 | 100.8275782 |
| 104 | Angola | 1995 | 218.7674647 | 0.287447813 | 100.8275782 |
| 105 | Angola | 1997 | 219.4821121 | 0.295433993 | 100.8275782 |
| 106 | Angola | 2004 | 224.2452849 | 0.327395266 | 65.82455226 |
| 107 | Angola | 2005 | 225.0799015 | 0.333785188 | 65.82455226 |
| 108 | Angola | 2007 | 227.3311364 | 0.348846841 | 65.82455226 |
| 109 | Angola | 2008 | 228.7124308 | 0.357082005 | 64.06460795 |
| 110 | Angola | 2009 | 229.9830075 | 0.364407744 | 64.06460795 |
| 111 | Angola | 2010 | 230.9334234 | 0.372033537 | 64.06460795 |
| 112 | Angola | 2012 | 232.594097 | 0.387891857 | 62.92622144 |
| 113 | Angola | 2013 | 233.4874257 | 0.395796329 | 62.92622144 |
| 114 | Angola | 2016 | 235.9648028 | 0.418822062 | 62.92622144 |
| 115 | Angola | 2017 | 237.149612 | 0.426177082 | 62.92622144 |
| 116 | Angola | 2018 | 238.2829069 | 0.433355005 | 62.92622144 |
| 117 | Angola | 2019 | 239.0278831 | 0.440454598 | 62.92622144 |
| 118 | Angola | 2020 | 238.8550427 | 0.447283992 | 62.92622144 |
| 119 | Angola | 2021 | 240.0535405 | 0.453721949 | 62.92622144 |
| 120 | Antigua and Barbuda | 1990 | 275.5368935 | 0.612104591 | 51.75814966 |
| 121 | Antigua and Barbuda | 1991 | 275.9667492 | 0.618817129 | 32.04043631 |
| 122 | Antigua and Barbuda | 1992 | 276.3935648 | 0.624695818 | 32.04043631 |
| 123 | Antigua and Barbuda | 1993 | 276.7805035 | 0.630075854 | 22.46369183 |
| 124 | Antigua and Barbuda | 1995 | 277.3432625 | 0.638008629 | 22.46369183 |
| 125 | Antigua and Barbuda | 1996 | 277.6847684 | 0.641647192 | 22.46369183 |
| 126 | Antigua and Barbuda | 1997 | 278.2152723 | 0.645895591 | 22.46369183 |
| 127 | Antigua and Barbuda | 2001 | 279.1298938 | 0.665620632 | 22.46369183 |
| 128 | Antigua and Barbuda | 2002 | 277.3689162 | 0.670360801 | 22.46369183 |
| 129 | Antigua and Barbuda | 2003 | 275.2908441 | 0.67531149 | 22.03195463 |
| 130 | Antigua and Barbuda | 2006 | 275.7005721 | 0.690826021 | 17.2540694 |
| 131 | Antigua and Barbuda | 2008 | 283.2182288 | 0.702175362 | 17.2540694 |
| 132 | Antigua and Barbuda | 2009 | 287.5679888 | 0.707128564 | 17.2540694 |
| 133 | Antigua and Barbuda | 2010 | 291.4754265 | 0.711432246 | 17.2540694 |
| 134 | Antigua and Barbuda | 2011 | 295.1453874 | 0.715489989 | 17.2540694 |
| 135 | Antigua and Barbuda | 2012 | 299.1117958 | 0.719509323 | 15.06516239 |
| 136 | Antigua and Barbuda | 2013 | 303.1844096 | 0.722864978 | 15.06516239 |
| 137 | Antigua and Barbuda | 2014 | 307.1541742 | 0.725585978 | 14.77524801 |
| 138 | Antigua and Barbuda | 2015 | 310.8001259 | 0.728372762 | 14.22935117 |
| 139 | Antigua and Barbuda | 2016 | 314.6910114 | 0.731598944 | 14.22935117 |
| 140 | Antigua and Barbuda | 2021 | 309.2925769 | 0.749886887 | 13.44730416 |
| 141 | Argentina | 1990 | 87.28601328 | 0.587397284 | 51.75814966 |
| 142 | Argentina | 1991 | 86.95374768 | 0.59170754 | 51.75814966 |
| 143 | Argentina | 1992 | 86.70682871 | 0.597996324 | 51.75814966 |
| 144 | Argentina | 1993 | 86.54504392 | 0.603739312 | 51.75814966 |
| 145 | Argentina | 1994 | 86.46980186 | 0.60978733 | 51.75814966 |
| 146 | Argentina | 1995 | 86.48120323 | 0.614473706 | 32.04043631 |
| 147 | Argentina | 1996 | 86.60399043 | 0.618942624 | 32.04043631 |
| 148 | Argentina | 1998 | 87.17301494 | 0.628723436 | 22.46369183 |
| 149 | Argentina | 2000 | 87.98323045 | 0.638194472 | 22.46369183 |
| 150 | Argentina | 2003 | 89.78143451 | 0.645906577 | 22.46369183 |
| 151 | Argentina | 2005 | 91.14396522 | 0.65336883 | 22.46369183 |
| 152 | Argentina | 2006 | 91.97454781 | 0.6574459 | 22.46369183 |
| 153 | Argentina | 2009 | 95.50023303 | 0.665911944 | 22.46369183 |
| 154 | Argentina | 2011 | 97.33910221 | 0.675242697 | 22.03195463 |
| 155 | Argentina | 2013 | 99.18331607 | 0.681633215 | 17.2540694 |
| 156 | Argentina | 2014 | 99.9876367 | 0.684974509 | 17.2540694 |
| 157 | Argentina | 2017 | 101.7148834 | 0.705135529 | 17.2540694 |
| 158 | Argentina | 2020 | 102.0063299 | 0.721294303 | 15.06516239 |
| 159 | Argentina | 2021 | 99.27745294 | 0.723122973 | 15.06516239 |
| 160 | Armenia | 1990 | 95.74207152 | 0.544414535 | 51.75814966 |
| 161 | Armenia | 1991 | 95.61111858 | 0.547561863 | 51.75814966 |
| 162 | Armenia | 1993 | 95.83780176 | 0.551214674 | 51.75814966 |
| 163 | Armenia | 1994 | 96.12677221 | 0.553783844 | 51.75814966 |
| 164 | Armenia | 1999 | 101.7606215 | 0.574323064 | 51.75814966 |
| 165 | Armenia | 2001 | 103.9601793 | 0.585419295 | 51.75814966 |
| 166 | Armenia | 2003 | 105.7534274 | 0.60080917 | 51.75814966 |
| 167 | Armenia | 2005 | 107.9306295 | 0.617759634 | 32.04043631 |
| 168 | Armenia | 2006 | 110.2256684 | 0.626688335 | 32.04043631 |
| 169 | Armenia | 2007 | 113.9033516 | 0.635870982 | 22.46369183 |
| 170 | Armenia | 2008 | 118.0470338 | 0.644431591 | 22.46369183 |
| 171 | Armenia | 2009 | 121.6956942 | 0.649714845 | 22.46369183 |
| 172 | Armenia | 2010 | 123.9046197 | 0.654811534 | 22.46369183 |
| 173 | Armenia | 2012 | 125.8847395 | 0.664833143 | 22.46369183 |
| 174 | Armenia | 2014 | 127.1967459 | 0.67393219 | 22.03195463 |
| 175 | Armenia | 2015 | 127.6114654 | 0.678054635 | 22.03195463 |
| 176 | Armenia | 2016 | 127.7051357 | 0.681746395 | 17.2540694 |
| 177 | Armenia | 2017 | 127.4286702 | 0.685980265 | 17.2540694 |
| 178 | Armenia | 2018 | 127.0053611 | 0.690276872 | 17.2540694 |
| 179 | Armenia | 2019 | 126.6131537 | 0.694765962 | 17.2540694 |
| 180 | Armenia | 2020 | 126.2966014 | 0.698268899 | 17.2540694 |
| 181 | Armenia | 2021 | 127.7631341 | 0.701833194 | 17.2540694 |
| 182 | Australia | 1990 | 30.93248476 | 0.725982519 | 14.77524801 |
| 183 | Australia | 1991 | 31.94169217 | 0.729759859 | 14.22935117 |
| 184 | Australia | 1992 | 32.90642889 | 0.733901404 | 14.22935117 |
| 185 | Australia | 1993 | 33.80352401 | 0.738374743 | 13.44730416 |
| 186 | Australia | 1995 | 35.28048388 | 0.747426824 | 13.44730416 |
| 187 | Australia | 2000 | 38.20835019 | 0.770223843 | 9.92126395 |
| 188 | Australia | 2001 | 38.67380085 | 0.774662787 | 9.92126395 |
| 189 | Australia | 2002 | 39.15854544 | 0.77936094 | 9.92126395 |
| 190 | Australia | 2003 | 39.69515835 | 0.783600181 | 8.445315313 |
| 191 | Australia | 2004 | 40.30904531 | 0.787016543 | 8.445315313 |
| 192 | Australia | 2005 | 41.02696812 | 0.789615521 | 8.445315313 |
| 193 | Australia | 2006 | 42.30984049 | 0.791155688 | 8.445315313 |
| 194 | Australia | 2009 | 48.57380041 | 0.799174373 | 8.445315313 |
| 195 | Australia | 2011 | 50.80952983 | 0.80752022 | 8.445315313 |
| 196 | Australia | 2012 | 51.49727768 | 0.811644461 | 8.445315313 |
| 197 | Australia | 2013 | 52.13649811 | 0.816453661 | 8.445315313 |
| 198 | Australia | 2014 | 52.81827427 | 0.82084085 | 8.445315313 |
| 199 | Australia | 2016 | 55.14013961 | 0.828559943 | 8.445315313 |
| 200 | Australia | 2019 | 60.31819956 | 0.839317426 | 8.445315313 |
| 201 | Austria | 1990 | 86.25461319 | 0.749853693 | 13.44730416 |
| 202 | Austria | 1993 | 99.00777019 | 0.758804554 | 9.92126395 |
| 203 | Austria | 1994 | 101.9405392 | 0.764704364 | 9.92126395 |
| 204 | Austria | 1995 | 103.8622864 | 0.769827471 | 9.92126395 |
| 205 | Austria | 1996 | 105.0446082 | 0.774446565 | 9.92126395 |
| 206 | Austria | 1997 | 105.9743337 | 0.779232856 | 9.92126395 |
| 207 | Austria | 1998 | 106.7841515 | 0.783521016 | 8.445315313 |
| 208 | Austria | 2000 | 108.5285814 | 0.79184768 | 8.445315313 |
| 209 | Austria | 2001 | 109.6513662 | 0.795770118 | 8.445315313 |
| 210 | Austria | 2004 | 113.5538379 | 0.804889144 | 8.445315313 |
| 211 | Austria | 2008 | 120.4624822 | 0.818793658 | 8.445315313 |
| 212 | Austria | 2009 | 122.1209264 | 0.821541167 | 8.445315313 |
| 213 | Austria | 2014 | 124.9424397 | 0.835838577 | 8.445315313 |
| 214 | Austria | 2015 | 125.3873593 | 0.837579895 | 8.445315313 |
| 215 | Austria | 2017 | 128.4728712 | 0.843173331 | 8.445315313 |
| 216 | Austria | 2018 | 130.247464 | 0.84661447 | 8.445315313 |
| 217 | Austria | 2019 | 130.9921245 | 0.849862515 | 8.445315313 |
| 218 | Austria | 2020 | 118.2696021 | 0.852020385 | 8.445315313 |
| 219 | Azerbaijan | 1990 | 99.27205187 | 0.595986033 | 51.75814966 |
| 220 | Azerbaijan | 1991 | 99.6319189 | 0.595807712 | 51.75814966 |
| 221 | Azerbaijan | 1993 | 100.2936223 | 0.593542676 | 51.75814966 |
| 222 | Azerbaijan | 1996 | 101.1906456 | 0.586315946 | 51.75814966 |
| 223 | Azerbaijan | 1998 | 101.7779189 | 0.580392893 | 51.75814966 |
| 224 | Azerbaijan | 1999 | 101.9017042 | 0.579192506 | 51.75814966 |
| 225 | Azerbaijan | 2000 | 101.7905355 | 0.580020573 | 51.75814966 |
| 226 | Azerbaijan | 2001 | 101.2241399 | 0.582193708 | 51.75814966 |
| 227 | Azerbaijan | 2002 | 100.2136272 | 0.585716143 | 51.75814966 |
| 228 | Azerbaijan | 2003 | 99.06885276 | 0.590038705 | 51.75814966 |
| 229 | Azerbaijan | 2004 | 98.08583759 | 0.594646085 | 51.75814966 |
| 230 | Azerbaijan | 2005 | 97.5768971 | 0.602485193 | 51.75814966 |
| 231 | Azerbaijan | 2009 | 100.7907204 | 0.645163341 | 22.46369183 |
| 232 | Azerbaijan | 2010 | 101.231837 | 0.652056507 | 22.46369183 |
| 233 | Azerbaijan | 2011 | 101.2707879 | 0.657971629 | 22.46369183 |
| 234 | Azerbaijan | 2012 | 101.2870585 | 0.662897002 | 22.46369183 |
| 235 | Azerbaijan | 2014 | 101.2884479 | 0.672162741 | 22.03195463 |
| 236 | Azerbaijan | 2015 | 101.285184 | 0.676244911 | 22.03195463 |
| 237 | Azerbaijan | 2016 | 101.3365214 | 0.679807643 | 22.03195463 |
| 238 | Azerbaijan | 2017 | 101.4316942 | 0.683263204 | 17.2540694 |
| 239 | Azerbaijan | 2018 | 101.5250134 | 0.686561093 | 17.2540694 |
| 240 | Azerbaijan | 2019 | 101.557286 | 0.689834715 | 17.2540694 |
| 241 | Azerbaijan | 2021 | 98.27238997 | 0.694851274 | 17.2540694 |
| 242 | Bahamas | 1991 | 308.7566455 | 0.689126784 | 17.2540694 |
| 243 | Bahamas | 1993 | 308.8075236 | 0.693816501 | 17.2540694 |
| 244 | Bahamas | 1995 | 309.8401255 | 0.719381607 | 15.06516239 |
| 245 | Bahamas | 1997 | 315.8187852 | 0.734781215 | 14.22935117 |
| 246 | Bahamas | 1998 | 320.676032 | 0.739281293 | 13.44730416 |
| 247 | Bahamas | 1999 | 325.2935487 | 0.741622848 | 13.44730416 |
| 248 | Bahamas | 2000 | 328.459937 | 0.742517946 | 13.44730416 |
| 249 | Bahamas | 2001 | 330.4660903 | 0.743592882 | 13.44730416 |
| 250 | Bahamas | 2002 | 332.4165228 | 0.746250322 | 13.44730416 |
| 251 | Bahamas | 2004 | 336.120022 | 0.753759643 | 10.45018691 |
| 252 | Bahamas | 2005 | 337.8385249 | 0.756782661 | 10.45018691 |
| 253 | Bahamas | 2008 | 346.9000733 | 0.765590503 | 9.92126395 |
| 254 | Bahamas | 2009 | 350.1831902 | 0.769561674 | 9.92126395 |
| 255 | Bahamas | 2010 | 352.5594487 | 0.77411981 | 9.92126395 |
| 256 | Bahamas | 2011 | 354.225368 | 0.778281891 | 9.92126395 |
| 257 | Bahamas | 2012 | 355.7949084 | 0.781838639 | 9.92126395 |
| 258 | Bahamas | 2014 | 358.4693171 | 0.787253492 | 8.445315313 |
| 259 | Bahamas | 2016 | 359.9108235 | 0.792738696 | 8.445315313 |
| 260 | Bahamas | 2021 | 329.8773997 | 0.805020668 | 8.445315313 |
| 261 | Bahrain | 1992 | 137.2059353 | 0.596040229 | 51.75814966 |
| 262 | Bahrain | 1994 | 135.9008428 | 0.608133725 | 51.75814966 |
| 263 | Bahrain | 1995 | 135.7300269 | 0.613850722 | 51.75814966 |
| 264 | Bahrain | 1996 | 136.0578267 | 0.619485376 | 32.04043631 |
| 265 | Bahrain | 1997 | 136.931901 | 0.624596062 | 32.04043631 |
| 266 | Bahrain | 1998 | 138.1338517 | 0.630738301 | 22.46369183 |
| 267 | Bahrain | 2003 | 146.0528015 | 0.669564111 | 22.46369183 |
| 268 | Bahrain | 2005 | 150.8687502 | 0.679790598 | 22.03195463 |
| 269 | Bahrain | 2006 | 153.4895876 | 0.686144289 | 17.2540694 |
| 270 | Bahrain | 2007 | 156.5011318 | 0.693045147 | 17.2540694 |
| 271 | Bahrain | 2008 | 159.6780432 | 0.699806387 | 17.2540694 |
| 272 | Bahrain | 2009 | 162.7936396 | 0.70488584 | 17.2540694 |
| 273 | Bahrain | 2011 | 168.5250416 | 0.708958739 | 17.2540694 |
| 274 | Bahrain | 2012 | 171.7406283 | 0.710362418 | 17.2540694 |
| 275 | Bahrain | 2013 | 174.8395366 | 0.713343156 | 17.2540694 |
| 276 | Bahrain | 2016 | 179.4837234 | 0.723611297 | 15.06516239 |
| 277 | Bahrain | 2017 | 180.045758 | 0.729323103 | 14.22935117 |
| 278 | Bahrain | 2018 | 180.4679052 | 0.736192583 | 13.44730416 |
| 279 | Bahrain | 2019 | 180.751385 | 0.742847804 | 13.44730416 |
| 280 | Bahrain | 2020 | 180.5104962 | 0.748103083 | 13.44730416 |
| 281 | Bangladesh | 1990 | 131.9035725 | 0.228548934 | 101.1599627 |
| 282 | Bangladesh | 1991 | 132.390945 | 0.237207413 | 101.1599627 |
| 283 | Bangladesh | 1994 | 133.6462859 | 0.258997294 | 100.8275782 |
| 284 | Bangladesh | 1995 | 133.9801394 | 0.265293918 | 100.8275782 |
| 285 | Bangladesh | 1996 | 134.2742727 | 0.271177781 | 100.8275782 |
| 286 | Bangladesh | 1997 | 134.5676612 | 0.27777328 | 100.8275782 |
| 287 | Bangladesh | 1998 | 134.8771023 | 0.28451594 | 100.8275782 |
| 288 | Bangladesh | 1999 | 135.2014071 | 0.290951431 | 100.8275782 |
| 289 | Bangladesh | 2000 | 135.5792479 | 0.297251976 | 100.8275782 |
| 290 | Bangladesh | 2001 | 136.1107473 | 0.303188546 | 89.96318282 |
| 291 | Bangladesh | 2002 | 136.7707721 | 0.308640093 | 89.96318282 |
| 292 | Bangladesh | 2003 | 137.4599597 | 0.31436004 | 67.17014806 |
| 293 | Bangladesh | 2005 | 138.8073696 | 0.32809051 | 65.82455226 |
| 294 | Bangladesh | 2006 | 139.4877107 | 0.335690598 | 65.82455226 |
| 295 | Bangladesh | 2007 | 140.2502952 | 0.343932546 | 65.82455226 |
| 296 | Bangladesh | 2010 | 141.7588836 | 0.370598956 | 64.06460795 |
| 297 | Bangladesh | 2012 | 139.802151 | 0.390623552 | 62.92622144 |
| 298 | Bangladesh | 2013 | 138.0185803 | 0.401706478 | 62.92622144 |
| 299 | Bangladesh | 2014 | 136.5441888 | 0.413263169 | 62.92622144 |
| 300 | Bangladesh | 2016 | 135.9908123 | 0.437709906 | 62.92622144 |
| 301 | Bangladesh | 2017 | 136.2759231 | 0.449752413 | 62.92622144 |
| 302 | Bangladesh | 2019 | 137.0851846 | 0.473264998 | 53.58976548 |
| 303 | Bangladesh | 2020 | 138.1755674 | 0.483079169 | 53.58976548 |
| 304 | Bangladesh | 2021 | 139.476863 | 0.492420885 | 53.58976548 |
| 305 | Barbados | 1991 | 178.482485 | 0.656654073 | 22.46369183 |
| 306 | Barbados | 1993 | 178.9372474 | 0.668815012 | 22.46369183 |
| 307 | Barbados | 1997 | 182.7352494 | 0.678644826 | 22.03195463 |
| 308 | Barbados | 1998 | 184.8951988 | 0.679827167 | 22.03195463 |
| 309 | Barbados | 2000 | 189.2062845 | 0.681304199 | 17.2540694 |
| 310 | Barbados | 2006 | 203.8509806 | 0.703856186 | 17.2540694 |
| 311 | Barbados | 2007 | 206.5646088 | 0.705871649 | 17.2540694 |
| 312 | Barbados | 2009 | 211.4890855 | 0.712143362 | 17.2540694 |
| 313 | Barbados | 2011 | 214.080998 | 0.720060687 | 15.06516239 |
| 314 | Barbados | 2012 | 214.9757405 | 0.724162458 | 15.06516239 |
| 315 | Barbados | 2013 | 215.7413163 | 0.72782617 | 14.22935117 |
| 316 | Barbados | 2016 | 217.5277163 | 0.734912535 | 14.22935117 |
| 317 | Barbados | 2017 | 218.2395183 | 0.737157255 | 13.44730416 |
| 318 | Barbados | 2018 | 218.9237783 | 0.739531437 | 13.44730416 |
| 319 | Barbados | 2019 | 219.4221344 | 0.742239309 | 13.44730416 |
| 320 | Barbados | 2020 | 219.8424264 | 0.744366646 | 13.44730416 |
| 321 | Barbados | 2021 | 215.6066038 | 0.746748764 | 13.44730416 |
| 322 | Belarus | 1991 | 22.46369183 | 0.627038267 | 22.46369183 |
| 323 | Belarus | 1993 | 22.55554012 | 0.639394169 | 22.46369183 |
| 324 | Belarus | 1994 | 22.57918481 | 0.644652781 | 22.46369183 |
| 325 | Belarus | 1995 | 22.58594457 | 0.648959504 | 22.46369183 |
| 326 | Belarus | 1996 | 22.58794218 | 0.652855221 | 22.46369183 |
| 327 | Belarus | 1999 | 22.53665794 | 0.66017101 | 22.46369183 |
| 328 | Belarus | 2001 | 22.03195463 | 0.671333269 | 22.03195463 |
| 329 | Belarus | 2004 | 18.74339411 | 0.692502135 | 17.2540694 |
| 330 | Belarus | 2005 | 17.70617769 | 0.698985483 | 17.2540694 |
| 331 | Belarus | 2008 | 15.06516239 | 0.719120871 | 15.06516239 |
| 332 | Belarus | 2009 | 14.22935117 | 0.727292784 | 14.22935117 |
| 333 | Belarus | 2010 | 13.44730416 | 0.734954626 | 13.44730416 |
| 334 | Belarus | 2014 | 10.45018691 | 0.75351148 | 10.45018691 |
| 335 | Belarus | 2015 | 9.92126395 | 0.758205743 | 9.92126395 |
| 336 | Belarus | 2020 | 8.445315313 | 0.782381565 | 8.445315313 |
| 337 | Belgium | 1990 | 19.44948709 | 0.737390656 | 13.44730416 |
| 338 | Belgium | 1991 | 18.91133122 | 0.742173074 | 13.44730416 |
| 339 | Belgium | 1994 | 18.36050276 | 0.758903638 | 9.92126395 |
| 340 | Belgium | 1996 | 18.94558576 | 0.766826075 | 9.92126395 |
| 341 | Belgium | 1997 | 20.05708924 | 0.770813953 | 9.92126395 |
| 342 | Belgium | 1998 | 21.43437842 | 0.774228268 | 9.92126395 |
| 343 | Belgium | 2002 | 24.92230717 | 0.788836054 | 8.445315313 |
| 344 | Belgium | 2003 | 25.46681416 | 0.792014384 | 8.445315313 |
| 345 | Belgium | 2004 | 25.90070858 | 0.795224936 | 8.445315313 |
| 346 | Belgium | 2005 | 26.19405852 | 0.798497236 | 8.445315313 |
| 347 | Belgium | 2008 | 26.67259595 | 0.807757945 | 8.445315313 |
| 348 | Belgium | 2009 | 26.79736006 | 0.811055825 | 8.445315313 |
| 349 | Belgium | 2010 | 26.94185555 | 0.815030207 | 8.445315313 |
| 350 | Belgium | 2011 | 27.11942426 | 0.819470362 | 8.445315313 |
| 351 | Belgium | 2012 | 27.31919109 | 0.824226016 | 8.445315313 |
| 352 | Belgium | 2015 | 28.11458431 | 0.8358784 | 8.445315313 |
| 353 | Belgium | 2016 | 28.77383112 | 0.83946294 | 8.445315313 |
| 354 | Belgium | 2019 | 31.0832887 | 0.849498395 | 8.445315313 |
| 355 | Belgium | 2020 | 25.22284765 | 0.851346649 | 8.445315313 |
| 356 | Belize | 1991 | 183.6594322 | 0.433834392 | 62.92622144 |
| 357 | Belize | 1993 | 182.9598194 | 0.455258577 | 62.92622144 |
| 358 | Belize | 1995 | 183.0464648 | 0.475278511 | 53.58976548 |
| 359 | Belize | 1996 | 183.6425683 | 0.482702165 | 53.58976548 |
| 360 | Belize | 1998 | 186.5780156 | 0.491819335 | 53.58976548 |
| 361 | Belize | 1999 | 188.4899506 | 0.495070196 | 53.58976548 |
| 362 | Belize | 2000 | 190.4446197 | 0.49864373 | 53.58976548 |
| 363 | Belize | 2001 | 192.8671335 | 0.502399434 | 53.58976548 |
| 364 | Belize | 2002 | 196.1258578 | 0.506871655 | 53.58976548 |
| 365 | Belize | 2009 | 226.2820612 | 0.553465869 | 51.75814966 |
| 366 | Belize | 2011 | 235.2247586 | 0.563440363 | 51.75814966 |
| 367 | Belize | 2012 | 239.7695427 | 0.568456236 | 51.75814966 |
| 368 | Belize | 2013 | 244.2325453 | 0.573236457 | 51.75814966 |
| 369 | Belize | 2016 | 255.7500655 | 0.588275912 | 51.75814966 |
| 370 | Belize | 2018 | 262.451443 | 0.598512015 | 51.75814966 |
| 371 | Belize | 2019 | 264.7358407 | 0.603351601 | 51.75814966 |
| 372 | Belize | 2021 | 254.8809433 | 0.610229002 | 51.75814966 |
| 373 | Benin | 1991 | 269.5675232 | 0.222539019 | 101.1599627 |
| 374 | Benin | 1992 | 270.2819749 | 0.226323465 | 101.1599627 |
| 375 | Benin | 1993 | 270.9072708 | 0.230312594 | 101.1599627 |
| 376 | Benin | 1998 | 271.8069905 | 0.249376451 | 100.8275782 |
| 377 | Benin | 2001 | 272.6135274 | 0.261388011 | 100.8275782 |
| 378 | Benin | 2002 | 273.4451095 | 0.265417458 | 100.8275782 |
| 379 | Benin | 2004 | 275.1683541 | 0.273941145 | 100.8275782 |
| 380 | Benin | 2005 | 275.579104 | 0.27840675 | 100.8275782 |
| 381 | Benin | 2006 | 275.7216111 | 0.282933936 | 100.8275782 |
| 382 | Benin | 2009 | 275.9323075 | 0.297702227 | 100.8275782 |
| 383 | Benin | 2010 | 275.8515295 | 0.302809008 | 89.96318282 |
| 384 | Benin | 2011 | 275.4661402 | 0.307925538 | 89.96318282 |
| 385 | Benin | 2012 | 274.734624 | 0.313373588 | 67.91401256 |
| 386 | Benin | 2014 | 273.1268768 | 0.325727272 | 66.46502371 |
| 387 | Benin | 2016 | 272.7779052 | 0.338763921 | 65.82455226 |
| 388 | Benin | 2017 | 273.0434121 | 0.345606759 | 65.82455226 |
| 389 | Benin | 2018 | 273.4457764 | 0.352652696 | 65.82455226 |
| 390 | Benin | 2019 | 273.7708935 | 0.360031163 | 64.06460795 |
| 391 | Bermuda | 1990 | 82.35839848 | 0.696451196 | 17.2540694 |
| 392 | Bermuda | 1991 | 82.70789168 | 0.700276851 | 17.2540694 |
| 393 | Bermuda | 1992 | 83.2451752 | 0.703984095 | 17.2540694 |
| 394 | Bermuda | 1993 | 83.93168791 | 0.707678222 | 17.2540694 |
| 395 | Bermuda | 1994 | 84.73209547 | 0.711068998 | 17.2540694 |
| 396 | Bermuda | 1995 | 85.61088583 | 0.714468828 | 17.2540694 |
| 397 | Bermuda | 1996 | 86.77754438 | 0.717831671 | 17.2540694 |
| 398 | Bermuda | 1997 | 88.35861313 | 0.721642813 | 15.06516239 |
| 399 | Bermuda | 2000 | 94.12514219 | 0.735996502 | 13.44730416 |
| 400 | Bermuda | 2003 | 100.7456791 | 0.753305163 | 13.44730416 |
| 401 | Bermuda | 2004 | 103.2297886 | 0.759008885 | 9.92126395 |
| 402 | Bermuda | 2005 | 105.7587912 | 0.764690903 | 9.92126395 |
| 403 | Bermuda | 2006 | 108.3881981 | 0.77059677 | 9.92126395 |
| 404 | Bermuda | 2007 | 111.2125477 | 0.776114264 | 9.92126395 |
| 405 | Bermuda | 2008 | 114.225746 | 0.780358325 | 9.92126395 |
| 406 | Bermuda | 2010 | 120.7925431 | 0.789181467 | 8.445315313 |
| 407 | Bermuda | 2011 | 124.8751892 | 0.793380524 | 8.445315313 |
| 408 | Bermuda | 2012 | 129.7612981 | 0.79748487 | 8.445315313 |
| 409 | Bermuda | 2013 | 134.795359 | 0.801388201 | 8.445315313 |
| 410 | Bermuda | 2015 | 142.6749484 | 0.807998795 | 8.445315313 |
| 411 | Bermuda | 2016 | 145.1840923 | 0.810550842 | 8.445315313 |
| 412 | Bermuda | 2018 | 149.2780043 | 0.814892802 | 8.445315313 |
| 413 | Bermuda | 2020 | 150.9121355 | 0.81920342 | 8.445315313 |
| 414 | Bhutan | 1990 | 131.6511524 | 0.21503985 | 101.1599627 |
| 415 | Bhutan | 1991 | 131.3885788 | 0.22131244 | 101.1599627 |
| 416 | Bhutan | 1993 | 131.0008452 | 0.236004687 | 101.1599627 |
| 417 | Bhutan | 1994 | 130.8677765 | 0.244331246 | 100.8275782 |
| 418 | Bhutan | 1995 | 130.7591341 | 0.253291957 | 100.8275782 |
| 419 | Bhutan | 1996 | 130.6986872 | 0.262362746 | 100.8275782 |
| 420 | Bhutan | 1997 | 130.7157608 | 0.271487422 | 100.8275782 |
| 421 | Bhutan | 2005 | 131.141483 | 0.346998578 | 65.82455226 |
| 422 | Bhutan | 2006 | 131.1917646 | 0.356824209 | 64.06460795 |
| 423 | Bhutan | 2007 | 131.2939979 | 0.367560498 | 64.06460795 |
| 424 | Bhutan | 2008 | 131.4134938 | 0.378233543 | 63.35324861 |
| 425 | Bhutan | 2009 | 131.5163159 | 0.388977162 | 62.92622144 |
| 426 | Bhutan | 2010 | 131.5584062 | 0.399751936 | 62.92622144 |
| 427 | Bhutan | 2011 | 131.4562408 | 0.410149249 | 62.92622144 |
| 428 | Bhutan | 2013 | 130.9460454 | 0.427790675 | 62.92622144 |
| 429 | Bhutan | 2015 | 130.668681 | 0.441810748 | 62.92622144 |
| 430 | Bhutan | 2016 | 130.7988937 | 0.448401855 | 62.92622144 |
| 431 | Bhutan | 2017 | 131.0379557 | 0.454280497 | 62.92622144 |
| 432 | Bhutan | 2019 | 131.5741924 | 0.464633353 | 62.92622144 |
| 433 | Bhutan | 2021 | 132.8865295 | 0.473062378 | 53.58976548 |
| 434 | Bolivia (Plurinational State of) | 1991 | 132.7790883 | 0.429397795 | 62.92622144 |
| 435 | Bolivia (Plurinational State of) | 1992 | 133.6585584 | 0.434835542 | 62.92622144 |
| 436 | Bolivia (Plurinational State of) | 1993 | 134.5887996 | 0.440601646 | 62.92622144 |
| 437 | Bolivia (Plurinational State of) | 1994 | 135.5502373 | 0.4468225 | 62.92622144 |
| 438 | Bolivia (Plurinational State of) | 1995 | 136.5220357 | 0.453479243 | 62.92622144 |
| 439 | Bolivia (Plurinational State of) | 1996 | 137.6942432 | 0.460220669 | 62.92622144 |
| 440 | Bolivia (Plurinational State of) | 1999 | 142.0283588 | 0.48019431 | 53.58976548 |
| 441 | Bolivia (Plurinational State of) | 2000 | 143.0712379 | 0.486327903 | 53.58976548 |
| 442 | Bolivia (Plurinational State of) | 2001 | 143.8911218 | 0.492030764 | 53.58976548 |
| 443 | Bolivia (Plurinational State of) | 2003 | 145.4564454 | 0.502365803 | 53.58976548 |
| 444 | Bolivia (Plurinational State of) | 2004 | 146.102713 | 0.507273036 | 53.58976548 |
| 445 | Bolivia (Plurinational State of) | 2005 | 146.595608 | 0.512166491 | 53.58976548 |
| 446 | Bolivia (Plurinational State of) | 2006 | 147.0494448 | 0.517146194 | 53.58976548 |
| 447 | Bolivia (Plurinational State of) | 2007 | 147.5609416 | 0.52194613 | 53.58976548 |
| 448 | Bolivia (Plurinational State of) | 2008 | 148.0551926 | 0.52694375 | 53.58976548 |
| 449 | Bolivia (Plurinational State of) | 2009 | 148.4563375 | 0.53179244 | 53.58976548 |
| 450 | Bolivia (Plurinational State of) | 2010 | 148.6887999 | 0.537009068 | 53.31984512 |
| 451 | Bolivia (Plurinational State of) | 2011 | 148.7961956 | 0.542680745 | 53.31984512 |
| 452 | Bolivia (Plurinational State of) | 2013 | 148.9365697 | 0.554788216 | 51.75814966 |
| 453 | Bolivia (Plurinational State of) | 2014 | 148.9911044 | 0.560934581 | 51.75814966 |
| 454 | Bolivia (Plurinational State of) | 2019 | 149.8628647 | 0.590692043 | 51.75814966 |
| 455 | Bolivia (Plurinational State of) | 2020 | 149.6110885 | 0.594854443 | 51.75814966 |
| 456 | Bosnia and Herzegovina | 1990 | 75.58675927 | 0.54113254 | 53.31984512 |
| 457 | Bosnia and Herzegovina | 1991 | 75.0241175 | 0.541346251 | 53.31984512 |
| 458 | Bosnia and Herzegovina | 1992 | 74.63190556 | 0.539303227 | 53.31984512 |
| 459 | Bosnia and Herzegovina | 1993 | 74.41530573 | 0.536031882 | 53.31984512 |
| 460 | Bosnia and Herzegovina | 1994 | 74.33633106 | 0.53385339 | 53.31984512 |
| 461 | Bosnia and Herzegovina | 1997 | 75.42445923 | 0.563565415 | 51.75814966 |
| 462 | Bosnia and Herzegovina | 1998 | 76.43907946 | 0.581829515 | 51.75814966 |
| 463 | Bosnia and Herzegovina | 1999 | 77.578251 | 0.597997469 | 51.75814966 |
| 464 | Bosnia and Herzegovina | 2000 | 78.69926677 | 0.610950685 | 51.75814966 |
| 465 | Bosnia and Herzegovina | 2001 | 80.18475408 | 0.621989464 | 32.04043631 |
| 466 | Bosnia and Herzegovina | 2002 | 82.21101016 | 0.632075518 | 22.46369183 |
| 467 | Bosnia and Herzegovina | 2005 | 87.99796622 | 0.653859137 | 22.46369183 |
| 468 | Bosnia and Herzegovina | 2007 | 90.21752077 | 0.665578735 | 22.46369183 |
| 469 | Bosnia and Herzegovina | 2008 | 91.17399284 | 0.671100814 | 22.46369183 |
| 470 | Bosnia and Herzegovina | 2012 | 93.21586682 | 0.689506566 | 17.2540694 |
| 471 | Bosnia and Herzegovina | 2015 | 93.85124973 | 0.700446047 | 17.2540694 |
| 472 | Bosnia and Herzegovina | 2016 | 93.79310556 | 0.704079344 | 17.2540694 |
| 473 | Bosnia and Herzegovina | 2018 | 93.39713953 | 0.712347302 | 17.2540694 |
| 474 | Bosnia and Herzegovina | 2021 | 87.91921949 | 0.723077893 | 15.06516239 |
| 475 | Botswana | 1990 | 203.0742473 | 0.418077748 | 62.92622144 |
| 476 | Botswana | 1996 | 201.3929132 | 0.478588352 | 53.58976548 |
| 477 | Botswana | 1998 | 199.596416 | 0.496193622 | 53.58976548 |
| 478 | Botswana | 1999 | 198.7163748 | 0.504978826 | 53.58976548 |
| 479 | Botswana | 2001 | 197.9353811 | 0.521370182 | 53.58976548 |
| 480 | Botswana | 2003 | 197.7003194 | 0.537000179 | 53.31984512 |
| 481 | Botswana | 2004 | 197.6704856 | 0.544349223 | 51.75814966 |
| 482 | Botswana | 2006 | 197.8431348 | 0.55922162 | 51.75814966 |
| 483 | Botswana | 2008 | 198.8627306 | 0.573883805 | 51.75814966 |
| 484 | Botswana | 2012 | 201.9085569 | 0.597739553 | 51.75814966 |
| 485 | Botswana | 2013 | 203.1257768 | 0.604064731 | 51.75814966 |
| 486 | Botswana | 2014 | 204.2525263 | 0.610086049 | 51.75814966 |
| 487 | Botswana | 2017 | 206.3227531 | 0.625704953 | 32.04043631 |
| 488 | Botswana | 2019 | 207.4936496 | 0.635324093 | 22.46369183 |
| 489 | Botswana | 2020 | 208.394131 | 0.639275298 | 22.46369183 |
| 490 | Brazil | 1990 | 149.3276723 | 0.500070509 | 53.58976548 |
| 491 | Brazil | 1992 | 149.5255898 | 0.508539943 | 53.58976548 |
| 492 | Brazil | 1994 | 150.0621518 | 0.515760842 | 53.58976548 |
| 493 | Brazil | 1995 | 150.4330176 | 0.519685963 | 53.58976548 |
| 494 | Brazil | 1997 | 151.989175 | 0.528177889 | 53.58976548 |
| 495 | Brazil | 1998 | 153.0481139 | 0.532481169 | 53.58976548 |
| 496 | Brazil | 1999 | 154.0884089 | 0.536972035 | 53.31984512 |
| 497 | Brazil | 2000 | 154.9482392 | 0.542051497 | 53.31984512 |
| 498 | Brazil | 2001 | 155.786837 | 0.54730443 | 51.75814966 |
| 499 | Brazil | 2003 | 157.661067 | 0.55814571 | 51.75814966 |
| 500 | Brazil | 2004 | 158.4443188 | 0.563772907 | 51.75814966 |
| 501 | Brazil | 2005 | 158.9420522 | 0.569458118 | 51.75814966 |
| 502 | Brazil | 2006 | 159.1550521 | 0.575338775 | 51.75814966 |
| 503 | Brazil | 2008 | 159.2223973 | 0.588077818 | 51.75814966 |
| 504 | Brazil | 2011 | 160.0338874 | 0.606777291 | 51.75814966 |
| 505 | Brazil | 2013 | 162.0874408 | 0.618218237 | 32.04043631 |
| 506 | Brazil | 2014 | 162.9688834 | 0.623433814 | 32.04043631 |
| 507 | Brazil | 2015 | 163.3417834 | 0.628149803 | 22.46369183 |
| 508 | Brazil | 2016 | 162.9913794 | 0.632235114 | 22.46369183 |
| 509 | Brazil | 2018 | 161.3281595 | 0.640802974 | 22.46369183 |
| 510 | Brazil | 2019 | 160.8399012 | 0.645298005 | 22.46369183 |
| 511 | Brazil | 2020 | 161.0760364 | 0.649201568 | 22.46369183 |
| 512 | Brazil | 2021 | 165.339856 | 0.653043887 | 22.46369183 |
| 513 | Brunei Darussalam | 1990 | 58.0132564 | 0.666081917 | 22.46369183 |
| 514 | Brunei Darussalam | 1991 | 58.54094033 | 0.671233831 | 22.46369183 |
| 515 | Brunei Darussalam | 1992 | 59.08773078 | 0.676702526 | 22.03195463 |
| 516 | Brunei Darussalam | 1994 | 59.85381922 | 0.687394093 | 17.2540694 |
| 517 | Brunei Darussalam | 1995 | 59.95752243 | 0.692471156 | 17.2540694 |
| 518 | Brunei Darussalam | 1996 | 59.85414292 | 0.697602233 | 17.2540694 |
| 519 | Brunei Darussalam | 1997 | 59.58454526 | 0.702944386 | 17.2540694 |
| 520 | Brunei Darussalam | 1998 | 59.23785458 | 0.708630119 | 17.2540694 |
| 521 | Brunei Darussalam | 2000 | 58.64878278 | 0.721552493 | 15.06516239 |
| 522 | Brunei Darussalam | 2002 | 58.31357893 | 0.735343916 | 13.44730416 |
| 523 | Brunei Darussalam | 2003 | 58.19169102 | 0.741578091 | 13.44730416 |
| 524 | Brunei Darussalam | 2004 | 58.10155806 | 0.747311715 | 13.44730416 |
| 525 | Brunei Darussalam | 2005 | 58.02104589 | 0.752481793 | 13.44730416 |
| 526 | Brunei Darussalam | 2007 | 57.03582619 | 0.761643129 | 9.92126395 |
| 527 | Brunei Darussalam | 2008 | 56.20284014 | 0.76571426 | 9.92126395 |
| 528 | Brunei Darussalam | 2009 | 55.43212309 | 0.769601682 | 9.92126395 |
| 529 | Brunei Darussalam | 2010 | 55.05868784 | 0.773219221 | 9.92126395 |
| 530 | Brunei Darussalam | 2011 | 55.01208986 | 0.776631893 | 9.92126395 |
| 531 | Brunei Darussalam | 2012 | 55.12335734 | 0.780216552 | 9.92126395 |
| 532 | Brunei Darussalam | 2014 | 55.74053683 | 0.787733289 | 8.445315313 |
| 533 | Brunei Darussalam | 2017 | 57.11012965 | 0.798309936 | 8.445315313 |
| 534 | Brunei Darussalam | 2019 | 57.61857354 | 0.804499388 | 8.445315313 |
| 535 | Brunei Darussalam | 2020 | 57.54744897 | 0.807424091 | 8.445315313 |
| 536 | Brunei Darussalam | 2021 | 56.98592503 | 0.810234367 | 8.445315313 |
| 537 | Bulgaria | 1991 | 141.8363818 | 0.643069488 | 22.46369183 |
| 538 | Bulgaria | 1992 | 144.4522245 | 0.651186876 | 22.46369183 |
| 539 | Bulgaria | 1993 | 147.7259858 | 0.659161569 | 22.46369183 |
| 540 | Bulgaria | 1996 | 160.6839895 | 0.678862584 | 22.03195463 |
| 541 | Bulgaria | 1997 | 167.2351234 | 0.680221466 | 17.2540694 |
| 542 | Bulgaria | 1999 | 182.4191535 | 0.677446543 | 22.03195463 |
| 543 | Bulgaria | 2000 | 189.9502648 | 0.681004524 | 17.2540694 |
| 544 | Bulgaria | 2002 | 205.4936644 | 0.691511674 | 17.2540694 |
| 545 | Bulgaria | 2003 | 213.6235181 | 0.695565965 | 17.2540694 |
| 546 | Bulgaria | 2004 | 221.5848487 | 0.699881951 | 17.2540694 |
| 547 | Bulgaria | 2005 | 229.0794991 | 0.704471207 | 17.2540694 |
| 548 | Bulgaria | 2007 | 244.1101104 | 0.713192402 | 17.2540694 |
| 549 | Bulgaria | 2008 | 251.3977835 | 0.71767245 | 17.2540694 |
| 550 | Bulgaria | 2012 | 271.1826877 | 0.739177517 | 13.44730416 |
| 551 | Bulgaria | 2013 | 274.6570358 | 0.742135948 | 13.44730416 |
| 552 | Bulgaria | 2014 | 277.3532115 | 0.745372643 | 13.44730416 |
| 553 | Bulgaria | 2015 | 279.0190332 | 0.748979058 | 13.44730416 |
| 554 | Bulgaria | 2016 | 280.0023106 | 0.752259199 | 13.44730416 |
| 555 | Bulgaria | 2017 | 280.7984301 | 0.755665865 | 10.45018691 |
| 556 | Bulgaria | 2018 | 281.3699288 | 0.75914846 | 9.92126395 |
| 557 | Bulgaria | 2019 | 281.6857739 | 0.762359791 | 9.92126395 |
| 558 | Bulgaria | 2020 | 280.6520517 | 0.765089711 | 9.92126395 |
| 559 | Bulgaria | 2021 | 239.0346679 | 0.768150939 | 9.92126395 |
| 560 | Burkina Faso | 1992 | 268.0704728 | 0.136594798 | 245.5041638 |
| 561 | Burkina Faso | 1993 | 268.4648856 | 0.139825465 | 245.5041638 |
| 562 | Burkina Faso | 1996 | 268.8695667 | 0.150041686 | 245.5041638 |
| 563 | Burkina Faso | 1997 | 268.6792174 | 0.154228401 | 245.5041638 |
| 564 | Burkina Faso | 1998 | 268.4824352 | 0.158707048 | 245.5041638 |
| 565 | Burkina Faso | 2003 | 272.2020619 | 0.183253539 | 238.2077266 |
| 566 | Burkina Faso | 2004 | 273.6869196 | 0.188564995 | 237.6563045 |
| 567 | Burkina Faso | 2009 | 276.6171821 | 0.215423374 | 101.1599627 |
| 568 | Burkina Faso | 2012 | 278.3523689 | 0.232589135 | 101.1599627 |
| 569 | Burkina Faso | 2013 | 279.1525279 | 0.238497491 | 101.1599627 |
| 570 | Burkina Faso | 2014 | 279.8174547 | 0.244378901 | 100.8275782 |
| 571 | Burkina Faso | 2015 | 280.1363116 | 0.250208261 | 100.8275782 |
| 572 | Burkina Faso | 2016 | 280.0787402 | 0.256081774 | 100.8275782 |
| 573 | Burkina Faso | 2018 | 279.3833094 | 0.268102073 | 100.8275782 |
| 574 | Burkina Faso | 2020 | 281.0527721 | 0.279871317 | 100.8275782 |
| 575 | Burkina Faso | 2021 | 281.8734318 | 0.285118402 | 100.8275782 |
| 576 | Burundi | 1991 | 277.6394154 | 0.20849572 | 101.1599627 |
| 577 | Burundi | 1992 | 277.2155244 | 0.210864619 | 101.1599627 |
| 578 | Burundi | 1993 | 276.8711882 | 0.213249386 | 101.1599627 |
| 579 | Burundi | 1994 | 276.6572082 | 0.215222973 | 101.1599627 |
| 580 | Burundi | 2000 | 276.6366765 | 0.216869858 | 101.1599627 |
| 581 | Burundi | 2001 | 277.0707729 | 0.216988726 | 101.1599627 |
| 582 | Burundi | 2002 | 277.8231197 | 0.217782477 | 101.1599627 |
| 583 | Burundi | 2003 | 278.7164193 | 0.218961537 | 101.1599627 |
| 584 | Burundi | 2004 | 279.5604459 | 0.220918709 | 101.1599627 |
| 585 | Burundi | 2006 | 280.732742 | 0.22582189 | 101.1599627 |
| 586 | Burundi | 2008 | 281.9336227 | 0.232853551 | 101.1599627 |
| 587 | Burundi | 2009 | 282.5653685 | 0.23694304 | 101.1599627 |
| 588 | Burundi | 2010 | 283.1483559 | 0.241447786 | 100.8275782 |
| 589 | Burundi | 2011 | 283.6447496 | 0.246277277 | 100.8275782 |
| 590 | Burundi | 2012 | 284.3067988 | 0.251572303 | 100.8275782 |
| 591 | Burundi | 2013 | 285.0198315 | 0.257191355 | 100.8275782 |
| 592 | Burundi | 2014 | 285.6414314 | 0.262794722 | 100.8275782 |
| 593 | Burundi | 2017 | 286.3058613 | 0.275840505 | 100.8275782 |
| 594 | Burundi | 2019 | 286.6128086 | 0.283442515 | 100.8275782 |
| 595 | Burundi | 2020 | 287.8742396 | 0.286496 | 100.8275782 |
| 596 | Burundi | 2021 | 288.8691329 | 0.289374365 | 100.8275782 |
| 597 | Cabo Verde | 1990 | 297.0144048 | 0.276723367 | 100.8275782 |
| 598 | Cabo Verde | 1991 | 296.6354352 | 0.282931722 | 100.8275782 |
| 599 | Cabo Verde | 1992 | 296.297682 | 0.288460371 | 100.8275782 |
| 600 | Cabo Verde | 1993 | 295.9957189 | 0.294517286 | 100.8275782 |
| 601 | Cabo Verde | 1994 | 295.7389915 | 0.302216794 | 89.96318282 |
| 602 | Cabo Verde | 1995 | 295.553944 | 0.310675634 | 89.96318282 |
| 603 | Cabo Verde | 1997 | 295.1469982 | 0.329049585 | 65.82455226 |
| 604 | Cabo Verde | 1998 | 294.9305243 | 0.338786069 | 65.82455226 |
| 605 | Cabo Verde | 2001 | 295.357533 | 0.370070276 | 64.06460795 |
| 606 | Cabo Verde | 2002 | 296.5084924 | 0.379846674 | 63.35324861 |
| 607 | Cabo Verde | 2003 | 297.8681827 | 0.389419786 | 62.92622144 |
| 608 | Cabo Verde | 2004 | 299.0358257 | 0.398838732 | 62.92622144 |
| 609 | Cabo Verde | 2007 | 299.9496341 | 0.427379787 | 62.92622144 |
| 610 | Cabo Verde | 2008 | 300.1108109 | 0.43737213 | 62.92622144 |
| 611 | Cabo Verde | 2009 | 300.2471974 | 0.446691506 | 62.92622144 |
| 612 | Cabo Verde | 2011 | 300.3586877 | 0.464473969 | 62.92622144 |
| 613 | Cabo Verde | 2013 | 300.0503957 | 0.480796481 | 53.58976548 |
| 614 | Cabo Verde | 2014 | 299.9721253 | 0.488259896 | 53.58976548 |
| 615 | Cabo Verde | 2015 | 300.1185643 | 0.495229489 | 53.58976548 |
| 616 | Cabo Verde | 2016 | 300.6043658 | 0.501855424 | 53.58976548 |
| 617 | Cabo Verde | 2017 | 301.2956571 | 0.508435792 | 53.58976548 |
| 618 | Cabo Verde | 2018 | 301.7416978 | 0.515285886 | 53.58976548 |
| 619 | Cabo Verde | 2020 | 303.9386364 | 0.528461595 | 53.58976548 |
| 620 | Cabo Verde | 2021 | 306.1389172 | 0.533534539 | 53.31984512 |
| 621 | Cambodia | 1991 | 193.6850202 | 0.2924792 | 100.8275782 |
| 622 | Cambodia | 1993 | 193.4419704 | 0.301627202 | 89.96318282 |
| 623 | Cambodia | 1994 | 193.2850537 | 0.305722592 | 89.96318282 |
| 624 | Cambodia | 1995 | 193.1280644 | 0.310678881 | 89.96318282 |
| 625 | Cambodia | 1998 | 191.581943 | 0.324974299 | 66.46502371 |
| 626 | Cambodia | 2000 | 190.5087377 | 0.33607503 | 65.82455226 |
| 627 | Cambodia | 2002 | 190.2115125 | 0.349465712 | 65.82455226 |
| 628 | Cambodia | 2003 | 190.228814 | 0.35692966 | 64.06460795 |
| 629 | Cambodia | 2004 | 190.2923818 | 0.364854645 | 64.06460795 |
| 630 | Cambodia | 2006 | 190.4450971 | 0.381698166 | 63.35324861 |
| 631 | Cambodia | 2008 | 190.8535896 | 0.39767952 | 62.92622144 |
| 632 | Cambodia | 2010 | 191.5297705 | 0.410211121 | 62.92622144 |
| 633 | Cambodia | 2013 | 193.6139029 | 0.42771506 | 62.92622144 |
| 634 | Cambodia | 2014 | 194.290584 | 0.433597749 | 62.92622144 |
| 635 | Cambodia | 2015 | 194.692651 | 0.439487358 | 62.92622144 |
| 636 | Cambodia | 2016 | 194.8802372 | 0.445358172 | 62.92622144 |
| 637 | Cambodia | 2018 | 195.1372012 | 0.45734149 | 62.92622144 |
| 638 | Cambodia | 2019 | 195.2326618 | 0.46364343 | 62.92622144 |
| 639 | Cambodia | 2020 | 195.5582412 | 0.468876054 | 53.58976548 |
| 640 | Cambodia | 2021 | 196.6782206 | 0.473621491 | 53.58976548 |
| 641 | Cameroon | 1990 | 281.8333564 | 0.303055334 | 89.96318282 |
| 642 | Cameroon | 1993 | 275.0572434 | 0.320831654 | 66.46502371 |
| 643 | Cameroon | 1994 | 273.0109341 | 0.325410123 | 66.46502371 |
| 644 | Cameroon | 1997 | 266.8179366 | 0.337346001 | 65.82455226 |
| 645 | Cameroon | 1998 | 264.5999804 | 0.34119709 | 65.82455226 |
| 646 | Cameroon | 1999 | 262.9038036 | 0.344955364 | 65.82455226 |
| 647 | Cameroon | 2000 | 262.1349979 | 0.348814789 | 65.82455226 |
| 648 | Cameroon | 2002 | 262.83919 | 0.356511817 | 64.06460795 |
| 649 | Cameroon | 2003 | 263.718067 | 0.360668581 | 64.06460795 |
| 650 | Cameroon | 2005 | 265.1654044 | 0.369545756 | 64.06460795 |
| 651 | Cameroon | 2006 | 265.607338 | 0.374355878 | 64.06460795 |
| 652 | Cameroon | 2008 | 266.6207805 | 0.384838304 | 63.35324861 |
| 653 | Cameroon | 2010 | 267.8612266 | 0.396067145 | 62.92622144 |
| 654 | Cameroon | 2011 | 268.8671771 | 0.402092799 | 62.92622144 |
| 655 | Cameroon | 2013 | 272.0405567 | 0.415607965 | 62.92622144 |
| 656 | Cameroon | 2014 | 273.538147 | 0.42303356 | 62.92622144 |
| 657 | Cameroon | 2016 | 274.6153874 | 0.438878945 | 62.92622144 |
| 658 | Cameroon | 2018 | 274.4703487 | 0.455687847 | 62.92622144 |
| 659 | Cameroon | 2019 | 274.7117755 | 0.464246473 | 62.92622144 |
| 660 | Canada | 1990 | 38.65285878 | 0.781977864 | 9.92126395 |
| 661 | Canada | 1991 | 39.47149671 | 0.78415936 | 8.445315313 |
| 662 | Canada | 1993 | 40.95826568 | 0.789716593 | 8.445315313 |
| 663 | Canada | 1994 | 41.58607727 | 0.792598518 | 8.445315313 |
| 664 | Canada | 1995 | 42.10711873 | 0.796521166 | 8.445315313 |
| 665 | Canada | 1996 | 42.50434452 | 0.80106497 | 8.445315313 |
| 666 | Canada | 1997 | 42.82339891 | 0.804833356 | 8.445315313 |
| 667 | Canada | 1999 | 43.55549815 | 0.81226594 | 8.445315313 |
| 668 | Canada | 2000 | 44.12811326 | 0.816685059 | 8.445315313 |
| 669 | Canada | 2002 | 47.08981545 | 0.824402272 | 8.445315313 |
| 670 | Canada | 2003 | 49.39779301 | 0.827801096 | 8.445315313 |
| 671 | Canada | 2005 | 54.42028334 | 0.833288294 | 8.445315313 |
| 672 | Canada | 2006 | 57.29944628 | 0.83491566 | 8.445315313 |
| 673 | Canada | 2007 | 60.85731969 | 0.836625096 | 8.445315313 |
| 674 | Canada | 2011 | 73.67136138 | 0.848017833 | 8.445315313 |
| 675 | Canada | 2012 | 75.83505598 | 0.850978048 | 8.445315313 |
| 676 | Canada | 2014 | 80.44485482 | 0.856759934 | 8.445315313 |
| 677 | Canada | 2015 | 83.33820545 | 0.859532904 | 8.445315313 |
| 678 | Canada | 2020 | 93.15885881 | 0.871992631 | 8.445315313 |
| 679 | Central African Republic | 1990 | 198.6126113 | 0.216825191 | 101.1599627 |
| 680 | Central African Republic | 1991 | 198.9000798 | 0.220191656 | 101.1599627 |
| 681 | Central African Republic | 1993 | 199.3996429 | 0.225451125 | 101.1599627 |
| 682 | Central African Republic | 1995 | 199.7318564 | 0.231696792 | 101.1599627 |
| 683 | Central African Republic | 1996 | 199.7167245 | 0.233989591 | 101.1599627 |
| 684 | Central African Republic | 1998 | 199.6927283 | 0.239743529 | 101.1599627 |
| 685 | Central African Republic | 2000 | 199.7564961 | 0.246021132 | 100.8275782 |
| 686 | Central African Republic | 2001 | 199.7951748 | 0.24924674 | 100.8275782 |
| 687 | Central African Republic | 2002 | 199.9195567 | 0.252524369 | 100.8275782 |
| 688 | Central African Republic | 2004 | 200.3072725 | 0.257778778 | 100.8275782 |
| 689 | Central African Republic | 2005 | 200.5226102 | 0.260451918 | 100.8275782 |
| 690 | Central African Republic | 2006 | 200.9246862 | 0.263712446 | 100.8275782 |
| 691 | Central African Republic | 2007 | 201.617079 | 0.267332195 | 100.8275782 |
| 692 | Central African Republic | 2008 | 202.4281985 | 0.271158118 | 100.8275782 |
| 693 | Central African Republic | 2010 | 203.6702351 | 0.279981382 | 100.8275782 |
| 694 | Central African Republic | 2011 | 204.1432839 | 0.285097245 | 100.8275782 |
| 695 | Central African Republic | 2012 | 204.7304299 | 0.290460023 | 100.8275782 |
| 696 | Central African Republic | 2013 | 205.3288209 | 0.29010493 | 100.8275782 |
| 697 | Central African Republic | 2014 | 205.7883003 | 0.289999498 | 100.8275782 |
| 698 | Central African Republic | 2015 | 206.0640966 | 0.290827871 | 100.8275782 |
| 699 | Central African Republic | 2016 | 206.2622026 | 0.2924304 | 100.8275782 |
| 700 | Central African Republic | 2018 | 206.6737186 | 0.298011223 | 100.8275782 |
| 701 | Central African Republic | 2019 | 206.7552561 | 0.301953064 | 89.96318282 |
| 702 | Central African Republic | 2020 | 206.3659682 | 0.305426298 | 89.96318282 |
| 703 | Central African Republic | 2021 | 207.6542157 | 0.30916769 | 89.96318282 |
| 704 | Chad | 1990 | 251.316106 | 0.114638829 | 245.5041638 |
| 705 | Chad | 1991 | 251.571771 | 0.117954634 | 245.5041638 |
| 706 | Chad | 1992 | 251.7818793 | 0.121132832 | 245.5041638 |
| 707 | Chad | 1994 | 252.0674083 | 0.126080572 | 245.5041638 |
| 708 | Chad | 1995 | 252.1637856 | 0.128289492 | 245.5041638 |
| 709 | Chad | 1996 | 252.1514044 | 0.130169484 | 245.5041638 |
| 710 | Chad | 1997 | 252.0001626 | 0.132136021 | 245.5041638 |
| 711 | Chad | 1998 | 251.8226243 | 0.134090304 | 245.5041638 |
| 712 | Chad | 1999 | 251.7036471 | 0.135825261 | 245.5041638 |
| 713 | Chad | 2000 | 251.748002 | 0.137412374 | 245.5041638 |
| 714 | Chad | 2001 | 252.1222264 | 0.139663692 | 245.5041638 |
| 715 | Chad | 2002 | 252.8016871 | 0.1420417 | 245.5041638 |
| 716 | Chad | 2003 | 253.6300418 | 0.145348052 | 245.5041638 |
| 717 | Chad | 2004 | 254.418825 | 0.150910868 | 245.5041638 |
| 718 | Chad | 2006 | 255.6598297 | 0.162576402 | 245.5041638 |
| 719 | Chad | 2009 | 258.2689515 | 0.178335361 | 238.7663549 |
| 720 | Chad | 2011 | 259.5013571 | 0.189591609 | 237.6563045 |
| 721 | Chad | 2012 | 260.236046 | 0.195262749 | 237.6563045 |
| 722 | Chad | 2013 | 261.0177294 | 0.200952884 | 101.1599627 |
| 723 | Chad | 2015 | 262.2531686 | 0.212428697 | 101.1599627 |
| 724 | Chad | 2016 | 262.7548092 | 0.21753218 | 101.1599627 |
| 725 | Chad | 2017 | 263.3893278 | 0.22220695 | 101.1599627 |
| 726 | Chad | 2018 | 264.0755917 | 0.226913881 | 101.1599627 |
| 727 | Chad | 2021 | 267.4391306 | 0.240436019 | 101.1599627 |
| 728 | Chile | 1990 | 102.3231622 | 0.5864951 | 51.75814966 |
| 729 | Chile | 1992 | 102.4780415 | 0.599106083 | 51.75814966 |
| 730 | Chile | 1993 | 102.922759 | 0.604828509 | 51.75814966 |
| 731 | Chile | 1994 | 103.5269529 | 0.610673933 | 51.75814966 |
| 732 | Chile | 1995 | 104.2293397 | 0.617234677 | 32.04043631 |
| 733 | Chile | 1996 | 105.7322255 | 0.62369446 | 32.04043631 |
| 734 | Chile | 1997 | 108.3848742 | 0.630540085 | 22.46369183 |
| 735 | Chile | 1998 | 111.5933283 | 0.638082758 | 22.46369183 |
| 736 | Chile | 2003 | 122.7525128 | 0.673445927 | 22.03195463 |
| 737 | Chile | 2004 | 124.3379956 | 0.678258212 | 22.03195463 |
| 738 | Chile | 2005 | 125.8118198 | 0.682589191 | 17.2540694 |
| 739 | Chile | 2006 | 127.2547651 | 0.685892748 | 17.2540694 |
| 740 | Chile | 2007 | 128.7122743 | 0.688930323 | 17.2540694 |
| 741 | Chile | 2008 | 130.1372408 | 0.692359787 | 17.2540694 |
| 742 | Chile | 2011 | 133.8795665 | 0.708597936 | 17.2540694 |
| 743 | Chile | 2017 | 142.382192 | 0.752058077 | 13.44730416 |
| 744 | Chile | 2018 | 144.2709431 | 0.758625714 | 9.92126395 |
| 745 | Chile | 2019 | 145.4701671 | 0.765120323 | 9.92126395 |
| 746 | Chile | 2020 | 145.7865113 | 0.769213676 | 9.92126395 |
| 747 | Chile | 2021 | 139.5197123 | 0.771514716 | 9.92126395 |
| 748 | China | 1993 | 217.4730824 | 0.484353831 | 53.58976548 |
| 749 | China | 1994 | 216.7201809 | 0.492578645 | 53.58976548 |
| 750 | China | 1998 | 208.4344581 | 0.531379484 | 53.58976548 |
| 751 | China | 2005 | 173.5970126 | 0.588052194 | 51.75814966 |
| 752 | China | 2006 | 173.2147155 | 0.59875817 | 51.75814966 |
| 753 | China | 2007 | 173.063749 | 0.609520246 | 51.75814966 |
| 754 | China | 2009 | 173.3088606 | 0.629307727 | 22.46369183 |
| 755 | China | 2011 | 174.7391528 | 0.651447978 | 22.46369183 |
| 756 | China | 2015 | 181.8423417 | 0.671940111 | 22.03195463 |
| 757 | China | 2016 | 183.109949 | 0.676218437 | 22.03195463 |
| 758 | China | 2019 | 186.9301478 | 0.703686824 | 17.2540694 |
| 759 | Colombia | 1990 | 187.8538065 | 0.480720054 | 53.58976548 |
| 760 | Colombia | 1991 | 188.2772334 | 0.482339894 | 53.58976548 |
| 761 | Colombia | 1992 | 188.3945311 | 0.484572359 | 53.58976548 |
| 762 | Colombia | 1993 | 188.2655832 | 0.488229373 | 53.58976548 |
| 763 | Colombia | 1994 | 187.9512189 | 0.493278182 | 53.58976548 |
| 764 | Colombia | 1995 | 187.5132528 | 0.499102757 | 53.58976548 |
| 765 | Colombia | 1999 | 179.2137685 | 0.52243594 | 53.58976548 |
| 766 | Colombia | 2000 | 176.3817464 | 0.527839094 | 53.58976548 |
| 767 | Colombia | 2002 | 169.1196099 | 0.538189342 | 53.31984512 |
| 768 | Colombia | 2004 | 160.4320115 | 0.548601832 | 51.75814966 |
| 769 | Colombia | 2005 | 156.5725194 | 0.554089191 | 51.75814966 |
| 770 | Colombia | 2006 | 153.0693596 | 0.560182735 | 51.75814966 |
| 771 | Colombia | 2007 | 149.6023016 | 0.566822644 | 51.75814966 |
| 772 | Colombia | 2015 | 129.1238922 | 0.620889228 | 32.04043631 |
| 773 | Colombia | 2016 | 128.7454511 | 0.627554497 | 22.46369183 |
| 774 | Colombia | 2017 | 128.5927328 | 0.634130359 | 22.46369183 |
| 775 | Colombia | 2018 | 128.5337062 | 0.640255072 | 22.46369183 |
| 776 | Colombia | 2019 | 128.4369233 | 0.646038852 | 22.46369183 |
| 777 | Colombia | 2021 | 132.097932 | 0.655442913 | 22.46369183 |
| 778 | Comoros | 1990 | 297.571329 | 0.270048116 | 100.8275782 |
| 779 | Comoros | 1993 | 297.6942381 | 0.297607417 | 100.8275782 |
| 780 | Comoros | 1994 | 297.6240995 | 0.305706587 | 89.96318282 |
| 781 | Comoros | 1997 | 296.6979835 | 0.330148125 | 65.82455226 |
| 782 | Comoros | 1998 | 296.1193544 | 0.337517914 | 65.82455226 |
| 783 | Comoros | 2003 | 297.6674566 | 0.373410566 | 64.06460795 |
| 784 | Comoros | 2004 | 298.5461282 | 0.380035976 | 63.35324861 |
| 785 | Comoros | 2006 | 299.1334916 | 0.393521434 | 62.92622144 |
| 786 | Comoros | 2007 | 299.2155114 | 0.399051094 | 62.92622144 |
| 787 | Comoros | 2010 | 299.2113153 | 0.415543376 | 62.92622144 |
| 788 | Comoros | 2012 | 299.1603815 | 0.426729957 | 62.92622144 |
| 789 | Comoros | 2013 | 299.1746169 | 0.432789932 | 62.92622144 |
| 790 | Comoros | 2014 | 299.2109566 | 0.438844505 | 62.92622144 |
| 791 | Comoros | 2018 | 300.7432394 | 0.461951344 | 62.92622144 |
| 792 | Comoros | 2019 | 301.3688129 | 0.467388014 | 53.58976548 |
| 793 | Comoros | 2020 | 302.8213509 | 0.472003132 | 53.58976548 |
| 794 | Comoros | 2021 | 304.144365 | 0.475978688 | 53.58976548 |
| 795 | Congo | 1991 | 216.8066008 | 0.426734374 | 62.92622144 |
| 796 | Congo | 1993 | 217.7077224 | 0.436606594 | 62.92622144 |
| 797 | Congo | 1994 | 218.0764188 | 0.440168347 | 62.92622144 |
| 798 | Congo | 1996 | 218.386196 | 0.447566642 | 62.92622144 |
| 799 | Congo | 1997 | 218.5011929 | 0.451026961 | 62.92622144 |
| 800 | Congo | 2000 | 218.9252207 | 0.460764814 | 62.92622144 |
| 801 | Congo | 2001 | 219.27481 | 0.463751682 | 62.92622144 |
| 802 | Congo | 2003 | 220.6023033 | 0.470134374 | 53.58976548 |
| 803 | Congo | 2004 | 221.3448165 | 0.473612436 | 53.58976548 |
| 804 | Congo | 2005 | 221.9480198 | 0.478137627 | 53.58976548 |
| 805 | Congo | 2006 | 222.5827499 | 0.483355822 | 53.58976548 |
| 806 | Congo | 2008 | 224.3173937 | 0.492822855 | 53.58976548 |
| 807 | Congo | 2009 | 225.2349631 | 0.498299969 | 53.58976548 |
| 808 | Congo | 2010 | 226.0599567 | 0.505542849 | 53.58976548 |
| 809 | Congo | 2014 | 231.54702 | 0.540594486 | 53.31984512 |
| 810 | Congo | 2016 | 232.8192758 | 0.555108608 | 51.75814966 |
| 811 | Congo | 2017 | 233.1388024 | 0.5618341 | 51.75814966 |
| 812 | Congo | 2018 | 233.3994094 | 0.56779397 | 51.75814966 |
| 813 | Congo | 2019 | 233.6159024 | 0.573373665 | 51.75814966 |
| 814 | Congo | 2020 | 233.6676384 | 0.578451546 | 51.75814966 |
| 815 | Congo | 2021 | 234.8173513 | 0.583075236 | 51.75814966 |
| 816 | Cook Islands | 1991 | 333.9386241 | 0.570622776 | 51.75814966 |
| 817 | Cook Islands | 1993 | 335.8315983 | 0.584059175 | 51.75814966 |
| 818 | Cook Islands | 1995 | 336.9520415 | 0.598789968 | 51.75814966 |
| 819 | Cook Islands | 1997 | 338.0428039 | 0.613991564 | 51.75814966 |
| 820 | Cook Islands | 1998 | 338.8106242 | 0.621202228 | 32.04043631 |
| 821 | Cook Islands | 1999 | 339.535047 | 0.62802903 | 22.46369183 |
| 822 | Cook Islands | 2002 | 340.6887522 | 0.652790488 | 22.46369183 |
| 823 | Cook Islands | 2003 | 340.8110599 | 0.661465038 | 22.46369183 |
| 824 | Cook Islands | 2004 | 340.8527868 | 0.668769675 | 22.46369183 |
| 825 | Cook Islands | 2005 | 340.8554548 | 0.674004103 | 22.03195463 |
| 826 | Cook Islands | 2007 | 340.7665217 | 0.687505191 | 17.2540694 |
| 827 | Cook Islands | 2008 | 340.6384951 | 0.695592069 | 17.2540694 |
| 828 | Cook Islands | 2009 | 340.4836672 | 0.70377698 | 17.2540694 |
| 829 | Cook Islands | 2010 | 340.3504611 | 0.711861612 | 17.2540694 |
| 830 | Cook Islands | 2012 | 339.6730228 | 0.726963385 | 14.77524801 |
| 831 | Cook Islands | 2016 | 338.5493173 | 0.752353087 | 13.44730416 |
| 832 | Cook Islands | 2019 | 339.0366773 | 0.769795913 | 9.92126395 |
| 833 | Cook Islands | 2020 | 339.2320831 | 0.774700578 | 9.92126395 |
| 834 | Costa Rica | 1990 | 139.3324935 | 0.534125181 | 53.31984512 |
| 835 | Costa Rica | 1991 | 139.7179573 | 0.539342164 | 53.31984512 |
| 836 | Costa Rica | 1992 | 140.4896591 | 0.544624238 | 51.75814966 |
| 837 | Costa Rica | 1993 | 141.5706401 | 0.549946626 | 51.75814966 |
| 838 | Costa Rica | 1994 | 142.8801305 | 0.55517125 | 51.75814966 |
| 839 | Costa Rica | 1995 | 144.342412 | 0.560547086 | 51.75814966 |
| 840 | Costa Rica | 1997 | 150.9073913 | 0.570128417 | 51.75814966 |
| 841 | Costa Rica | 1999 | 159.0526569 | 0.582229492 | 51.75814966 |
| 842 | Costa Rica | 2000 | 161.1477282 | 0.588243191 | 51.75814966 |
| 843 | Costa Rica | 2001 | 161.8932618 | 0.594889115 | 51.75814966 |
| 844 | Costa Rica | 2003 | 162.4215578 | 0.605765687 | 51.75814966 |
| 845 | Costa Rica | 2004 | 162.2596073 | 0.610315699 | 51.75814966 |
| 846 | Costa Rica | 2005 | 161.8500442 | 0.614412619 | 32.04043631 |
| 847 | Costa Rica | 2006 | 161.0263445 | 0.617850162 | 32.04043631 |
| 848 | Costa Rica | 2007 | 159.7720262 | 0.621200571 | 32.04043631 |
| 849 | Costa Rica | 2008 | 158.3374186 | 0.62570391 | 32.04043631 |
| 850 | Costa Rica | 2009 | 156.9757494 | 0.631291379 | 22.46369183 |
| 851 | Costa Rica | 2010 | 155.9431882 | 0.636595426 | 22.46369183 |
| 852 | Costa Rica | 2012 | 154.1626806 | 0.646574093 | 22.46369183 |
| 853 | Costa Rica | 2016 | 151.5949684 | 0.667430092 | 22.46369183 |
| 854 | Costa Rica | 2019 | 150.1677589 | 0.690049088 | 17.2540694 |
| 855 | Costa Rica | 2020 | 150.0445273 | 0.696194605 | 17.2540694 |
| 856 | Costa Rica | 2021 | 156.4652191 | 0.700340477 | 17.2540694 |
| 857 | Croatia | 1990 | 91.89788303 | 0.668906358 | 22.46369183 |
| 858 | Croatia | 1991 | 91.44964026 | 0.6747246 | 22.03195463 |
| 859 | Croatia | 1992 | 90.72089801 | 0.678093133 | 22.03195463 |
| 860 | Croatia | 1994 | 88.52079927 | 0.677548133 | 22.03195463 |
| 861 | Croatia | 1995 | 87.06767176 | 0.677029465 | 22.03195463 |
| 862 | Croatia | 1997 | 81.97405923 | 0.68536501 | 17.2540694 |
| 863 | Croatia | 1998 | 78.87494614 | 0.691365519 | 17.2540694 |
| 864 | Croatia | 1999 | 76.29900923 | 0.696636411 | 17.2540694 |
| 865 | Croatia | 2000 | 74.93493782 | 0.703039899 | 17.2540694 |
| 866 | Croatia | 2001 | 74.68302311 | 0.709536841 | 17.2540694 |
| 867 | Croatia | 2003 | 74.97353832 | 0.721130377 | 15.06516239 |
| 868 | Croatia | 2008 | 69.54397462 | 0.748469602 | 13.44730416 |
| 869 | Croatia | 2009 | 67.5615927 | 0.754065605 | 10.45018691 |
| 870 | Croatia | 2010 | 66.63986392 | 0.759472252 | 9.92126395 |
| 871 | Croatia | 2011 | 66.70074672 | 0.763686381 | 9.92126395 |
| 872 | Croatia | 2012 | 67.04276636 | 0.767405336 | 9.92126395 |
| 873 | Croatia | 2014 | 67.8312936 | 0.774479241 | 9.92126395 |
| 874 | Croatia | 2016 | 67.71904966 | 0.781443605 | 9.92126395 |
| 875 | Croatia | 2017 | 67.44316371 | 0.785074537 | 8.445315313 |
| 876 | Croatia | 2018 | 67.27794214 | 0.78867141 | 8.445315313 |
| 877 | Croatia | 2019 | 67.41398293 | 0.792475415 | 8.445315313 |
| 878 | Croatia | 2021 | 72.97850703 | 0.798341027 | 8.445315313 |
| 879 | Cuba | 1990 | 68.53134284 | 0.558019071 | 51.75814966 |
| 880 | Cuba | 1991 | 69.96958448 | 0.563413638 | 51.75814966 |
| 881 | Cuba | 1992 | 71.76809306 | 0.566169006 | 51.75814966 |
| 882 | Cuba | 1994 | 76.20135577 | 0.563186313 | 51.75814966 |
| 883 | Cuba | 1995 | 78.72085766 | 0.560765902 | 51.75814966 |
| 884 | Cuba | 1996 | 81.59057751 | 0.559346329 | 51.75814966 |
| 885 | Cuba | 1998 | 88.69249905 | 0.55936322 | 51.75814966 |
| 886 | Cuba | 2000 | 96.93875738 | 0.566676902 | 51.75814966 |
| 887 | Cuba | 2001 | 101.6610852 | 0.572753861 | 51.75814966 |
| 888 | Cuba | 2002 | 107.0293124 | 0.57996829 | 51.75814966 |
| 889 | Cuba | 2003 | 112.6751072 | 0.587647762 | 51.75814966 |
| 890 | Cuba | 2004 | 118.2293447 | 0.594348715 | 51.75814966 |
| 891 | Cuba | 2005 | 123.32108 | 0.599863799 | 51.75814966 |
| 892 | Cuba | 2006 | 128.0126573 | 0.604970567 | 51.75814966 |
| 893 | Cuba | 2007 | 132.5958845 | 0.607618099 | 51.75814966 |
| 894 | Cuba | 2008 | 137.0481887 | 0.609400923 | 51.75814966 |
| 895 | Cuba | 2011 | 149.6603747 | 0.62038669 | 32.04043631 |
| 896 | Cuba | 2013 | 158.0290009 | 0.631839801 | 22.46369183 |
| 897 | Cuba | 2016 | 166.6705972 | 0.647907866 | 22.46369183 |
| 898 | Cuba | 2018 | 170.4976761 | 0.656858654 | 22.46369183 |
| 899 | Cuba | 2019 | 171.5187467 | 0.661674717 | 22.46369183 |
| 900 | Cuba | 2021 | 174.3776465 | 0.668729864 | 22.46369183 |
| 901 | Cyprus | 1991 | 56.72132562 | 0.655726169 | 22.46369183 |
| 902 | Cyprus | 1994 | 56.84112754 | 0.688829361 | 17.2540694 |
| 903 | Cyprus | 1997 | 60.14654536 | 0.718864244 | 17.2540694 |
| 904 | Cyprus | 2001 | 65.4237217 | 0.753594717 | 10.45018691 |
| 905 | Cyprus | 2002 | 64.54553738 | 0.761502465 | 9.92126395 |
| 906 | Cyprus | 2006 | 63.24228046 | 0.784560965 | 8.445315313 |
| 907 | Cyprus | 2007 | 64.66825129 | 0.792068383 | 8.445315313 |
| 908 | Cyprus | 2008 | 66.29071867 | 0.799454089 | 8.445315313 |
| 909 | Cyprus | 2009 | 67.64208784 | 0.805112085 | 8.445315313 |
| 910 | Cyprus | 2010 | 68.25518524 | 0.809840188 | 8.445315313 |
| 911 | Cyprus | 2011 | 67.57500777 | 0.813726132 | 8.445315313 |
| 912 | Cyprus | 2013 | 63.78809739 | 0.819380602 | 8.445315313 |
| 913 | Cyprus | 2015 | 61.29931042 | 0.822732851 | 8.445315313 |
| 914 | Cyprus | 2016 | 61.27499759 | 0.824283624 | 8.445315313 |
| 915 | Cyprus | 2017 | 61.3214527 | 0.826139878 | 8.445315313 |
| 916 | Cyprus | 2018 | 61.4944924 | 0.828343243 | 8.445315313 |
| 917 | Cyprus | 2019 | 61.83603216 | 0.830980728 | 8.445315313 |
| 918 | Czechia | 1990 | 31.96038443 | 0.681848021 | 17.2540694 |
| 919 | Czechia | 1991 | 33.76629346 | 0.688002859 | 17.2540694 |
| 920 | Czechia | 1993 | 38.74030374 | 0.710853416 | 17.2540694 |
| 921 | Czechia | 1994 | 41.74841025 | 0.725969686 | 14.77524801 |
| 922 | Czechia | 1995 | 44.99929053 | 0.737981722 | 13.44730416 |
| 923 | Czechia | 1996 | 48.872485 | 0.74604138 | 13.44730416 |
| 924 | Czechia | 1997 | 53.55267291 | 0.751680344 | 13.44730416 |
| 925 | Czechia | 1999 | 63.85746135 | 0.76242888 | 9.92126395 |
| 926 | Czechia | 2001 | 73.89752407 | 0.77216724 | 9.92126395 |
| 927 | Czechia | 2003 | 85.16842835 | 0.782448081 | 8.445315313 |
| 928 | Czechia | 2004 | 89.75818761 | 0.787126405 | 8.445315313 |
| 929 | Czechia | 2005 | 92.63373558 | 0.791333948 | 8.445315313 |
| 930 | Czechia | 2009 | 97.36460191 | 0.804962225 | 8.445315313 |
| 931 | Czechia | 2010 | 98.57774589 | 0.808747328 | 8.445315313 |
| 932 | Czechia | 2011 | 100.1770546 | 0.812054784 | 8.445315313 |
| 933 | Czechia | 2013 | 104.4643575 | 0.815615361 | 8.445315313 |
| 934 | Czechia | 2014 | 106.9893606 | 0.816227257 | 8.445315313 |
| 935 | Czechia | 2015 | 109.6547723 | 0.816564684 | 8.445315313 |
| 936 | Czechia | 2016 | 113.4471367 | 0.817153026 | 8.445315313 |
| 937 | Czechia | 2017 | 118.2670353 | 0.81906768 | 8.445315313 |
| 938 | Czechia | 2018 | 122.413273 | 0.822034486 | 8.445315313 |
| 939 | Czechia | 2019 | 124.1826646 | 0.824784473 | 8.445315313 |
| 940 | Democratic People's Republic of Korea | 1990 | 160.2514685 | 0.497780128 | 53.58976548 |
| 941 | Democratic People's Republic of Korea | 1991 | 160.2069076 | 0.499120512 | 53.58976548 |
| 942 | Democratic People's Republic of Korea | 1992 | 160.1542038 | 0.499629841 | 53.58976548 |
| 943 | Democratic People's Republic of Korea | 1993 | 160.1005807 | 0.499700646 | 53.58976548 |
| 944 | Democratic People's Republic of Korea | 1996 | 159.9912686 | 0.498429014 | 53.58976548 |
| 945 | Democratic People's Republic of Korea | 1997 | 159.8852607 | 0.497136869 | 53.58976548 |
| 946 | Democratic People's Republic of Korea | 1999 | 159.7873178 | 0.496484754 | 53.58976548 |
| 947 | Democratic People's Republic of Korea | 2001 | 160.3943205 | 0.499674043 | 53.58976548 |
| 948 | Democratic People's Republic of Korea | 2002 | 161.2828714 | 0.502633569 | 53.58976548 |
| 949 | Democratic People's Republic of Korea | 2003 | 162.4169514 | 0.506165243 | 53.58976548 |
| 950 | Democratic People's Republic of Korea | 2005 | 164.7999361 | 0.515097763 | 53.58976548 |
| 951 | Democratic People's Republic of Korea | 2006 | 166.0975032 | 0.51974144 | 53.58976548 |
| 952 | Democratic People's Republic of Korea | 2007 | 167.67601 | 0.524066965 | 53.58976548 |
| 953 | Democratic People's Republic of Korea | 2008 | 169.3210812 | 0.528813498 | 53.58976548 |
| 954 | Democratic People's Republic of Korea | 2011 | 172.8704972 | 0.54121227 | 53.31984512 |
| 955 | Democratic People's Republic of Korea | 2013 | 174.3719226 | 0.549483297 | 51.75814966 |
| 956 | Democratic People's Republic of Korea | 2014 | 175.0382246 | 0.55360356 | 51.75814966 |
| 957 | Democratic People's Republic of Korea | 2015 | 175.6964608 | 0.557321271 | 51.75814966 |
| 958 | Democratic People's Republic of Korea | 2016 | 176.5588974 | 0.560743563 | 51.75814966 |
| 959 | Democratic People's Republic of Korea | 2018 | 178.6477834 | 0.565161484 | 51.75814966 |
| 960 | Democratic People's Republic of Korea | 2020 | 179.864466 | 0.568188551 | 51.75814966 |
| 961 | Democratic People's Republic of Korea | 2021 | 181.4114532 | 0.569854634 | 51.75814966 |
| 962 | Democratic Republic of the Congo | 1991 | 223.0530965 | 0.290431567 | 100.8275782 |
| 963 | Democratic Republic of the Congo | 1992 | 222.5752649 | 0.290959242 | 100.8275782 |
| 964 | Democratic Republic of the Congo | 1994 | 221.7251137 | 0.286859146 | 100.8275782 |
| 965 | Democratic Republic of the Congo | 1996 | 220.9832552 | 0.28041316 | 100.8275782 |
| 966 | Democratic Republic of the Congo | 1998 | 220.1544575 | 0.271813823 | 100.8275782 |
| 967 | Democratic Republic of the Congo | 1999 | 219.7745203 | 0.267367662 | 100.8275782 |
| 968 | Democratic Republic of the Congo | 2002 | 219.2588946 | 0.25413636 | 100.8275782 |
| 969 | Democratic Republic of the Congo | 2003 | 219.308949 | 0.252333885 | 100.8275782 |
| 970 | Democratic Republic of the Congo | 2004 | 219.436059 | 0.252470919 | 100.8275782 |
| 971 | Democratic Republic of the Congo | 2006 | 220.0248428 | 0.257355123 | 100.8275782 |
| 972 | Democratic Republic of the Congo | 2007 | 220.7686143 | 0.262272267 | 100.8275782 |
| 973 | Democratic Republic of the Congo | 2008 | 221.7432715 | 0.268103872 | 100.8275782 |
| 974 | Democratic Republic of the Congo | 2009 | 222.847456 | 0.274361144 | 100.8275782 |
| 975 | Democratic Republic of the Congo | 2011 | 225.6026914 | 0.290872953 | 100.8275782 |
| 976 | Democratic Republic of the Congo | 2013 | 230.554127 | 0.310831256 | 89.96318282 |
| 977 | Democratic Republic of the Congo | 2015 | 234.5839853 | 0.332421307 | 65.82455226 |
| 978 | Democratic Republic of the Congo | 2017 | 237.0732977 | 0.351102035 | 65.82455226 |
| 979 | Democratic Republic of the Congo | 2018 | 238.1090238 | 0.360287677 | 64.06460795 |
| 980 | Democratic Republic of the Congo | 2019 | 238.7841278 | 0.369340684 | 64.06460795 |
| 981 | Democratic Republic of the Congo | 2021 | 240.212843 | 0.383179849 | 63.35324861 |
| 982 | Denmark | 1991 | 14.11548689 | 0.80454035 | 8.445315313 |
| 983 | Denmark | 1995 | 16.69615444 | 0.819104612 | 8.445315313 |
| 984 | Denmark | 1996 | 17.37316625 | 0.823375243 | 8.445315313 |
| 985 | Denmark | 1997 | 18.14835202 | 0.827679979 | 8.445315313 |
| 986 | Denmark | 1999 | 19.84513919 | 0.835381543 | 8.445315313 |
| 987 | Denmark | 2000 | 20.69468648 | 0.839184298 | 8.445315313 |
| 988 | Denmark | 2002 | 22.74055249 | 0.847086357 | 8.445315313 |
| 989 | Denmark | 2003 | 23.91131001 | 0.850441326 | 8.445315313 |
| 990 | Denmark | 2004 | 25.05901077 | 0.853304103 | 8.445315313 |
| 991 | Denmark | 2006 | 27.00609289 | 0.85803321 | 8.445315313 |
| 992 | Denmark | 2008 | 28.70934826 | 0.861782061 | 8.445315313 |
| 993 | Denmark | 2011 | 31.78394146 | 0.869778682 | 8.445315313 |
| 994 | Denmark | 2012 | 33.64081022 | 0.87251751 | 8.445315313 |
| 995 | Denmark | 2013 | 35.54947391 | 0.874932974 | 8.445315313 |
| 996 | Denmark | 2014 | 37.03648921 | 0.876814932 | 8.445315313 |
| 997 | Denmark | 2018 | 32.3925876 | 0.887529846 | 8.445315313 |
| 998 | Denmark | 2019 | 31.26050158 | 0.891624571 | 8.445315313 |
| 999 | Denmark | 2020 | 31.18936383 | 0.894368297 | 8.445315313 |
| 1000 | Djibouti | 1991 | 299.7789273 | 0.338770011 | 65.82455226 |
| 1001 | Djibouti | 1992 | 300.2300877 | 0.340986061 | 65.82455226 |
| 1002 | Djibouti | 1993 | 300.4479798 | 0.344904394 | 65.82455226 |
| 1003 | Djibouti | 1994 | 300.6184022 | 0.347981404 | 65.82455226 |
| 1004 | Djibouti | 1997 | 299.3065045 | 0.358096401 | 64.06460795 |
| 1005 | Djibouti | 1998 | 298.1721491 | 0.361596764 | 64.06460795 |
| 1006 | Djibouti | 1999 | 297.2398621 | 0.365305211 | 64.06460795 |
| 1007 | Djibouti | 2000 | 296.7891493 | 0.369015095 | 64.06460795 |
| 1008 | Djibouti | 2003 | 297.5965696 | 0.380686615 | 63.35324861 |
| 1009 | Djibouti | 2004 | 297.9437752 | 0.384987706 | 63.35324861 |
| 1010 | Djibouti | 2005 | 298.0848975 | 0.389568706 | 62.92622144 |
| 1011 | Djibouti | 2006 | 297.983073 | 0.395037471 | 62.92622144 |
| 1012 | Djibouti | 2007 | 297.6529222 | 0.400618546 | 62.92622144 |
| 1013 | Djibouti | 2008 | 297.2389425 | 0.406658303 | 62.92622144 |
| 1014 | Djibouti | 2011 | 296.6942002 | 0.42231614 | 62.92622144 |
| 1015 | Djibouti | 2012 | 296.6751624 | 0.42771144 | 62.92622144 |
| 1016 | Djibouti | 2014 | 296.7074344 | 0.439455722 | 62.92622144 |
| 1017 | Djibouti | 2015 | 296.7276278 | 0.445855285 | 62.92622144 |
| 1018 | Djibouti | 2018 | 298.2083311 | 0.467705672 | 53.58976548 |
| 1019 | Djibouti | 2020 | 300.1627753 | 0.481879945 | 53.58976548 |
| 1020 | Dominica | 1991 | 251.6989774 | 0.565237843 | 51.75814966 |
| 1021 | Dominica | 1992 | 251.9255168 | 0.569570684 | 51.75814966 |
| 1022 | Dominica | 1993 | 252.1867573 | 0.578811811 | 51.75814966 |
| 1023 | Dominica | 1996 | 253.2042624 | 0.61438205 | 32.04043631 |
| 1024 | Dominica | 1998 | 254.8067233 | 0.633017994 | 22.46369183 |
| 1025 | Dominica | 2001 | 256.2288646 | 0.652368567 | 22.46369183 |
| 1026 | Dominica | 2003 | 256.9597019 | 0.663678868 | 22.46369183 |
| 1027 | Dominica | 2004 | 257.3968733 | 0.668952132 | 22.46369183 |
| 1028 | Dominica | 2005 | 257.8550988 | 0.674549077 | 22.03195463 |
| 1029 | Dominica | 2006 | 258.6136182 | 0.680342482 | 17.2540694 |
| 1030 | Dominica | 2007 | 259.819192 | 0.685415917 | 17.2540694 |
| 1031 | Dominica | 2010 | 263.6795539 | 0.698489193 | 17.2540694 |
| 1032 | Dominica | 2011 | 264.6147445 | 0.70246326 | 17.2540694 |
| 1033 | Dominica | 2012 | 265.6615061 | 0.706870608 | 17.2540694 |
| 1034 | Dominica | 2013 | 266.7307369 | 0.712210475 | 17.2540694 |
| 1035 | Dominica | 2016 | 269.2342903 | 0.731760801 | 14.22935117 |
| 1036 | Dominica | 2018 | 270.7214115 | 0.738935954 | 13.44730416 |
| 1037 | Dominica | 2019 | 271.3248645 | 0.742132066 | 13.44730416 |
| 1038 | Dominica | 2020 | 271.5787644 | 0.744419944 | 13.44730416 |
| 1039 | Dominican Republic | 1990 | 191.781063 | 0.442654076 | 62.92622144 |
| 1040 | Dominican Republic | 1991 | 190.1074396 | 0.446144578 | 62.92622144 |
| 1041 | Dominican Republic | 1992 | 188.6088991 | 0.449953081 | 62.92622144 |
| 1042 | Dominican Republic | 1993 | 187.3858382 | 0.454229297 | 62.92622144 |
| 1043 | Dominican Republic | 1996 | 185.9389454 | 0.468516938 | 53.58976548 |
| 1044 | Dominican Republic | 1998 | 186.0530241 | 0.481170939 | 53.58976548 |
| 1045 | Dominican Republic | 1999 | 186.0893654 | 0.488178541 | 53.58976548 |
| 1046 | Dominican Republic | 2000 | 185.9567406 | 0.495468586 | 53.58976548 |
| 1047 | Dominican Republic | 2001 | 185.439933 | 0.502775415 | 53.58976548 |
| 1048 | Dominican Republic | 2002 | 184.5099294 | 0.510298019 | 53.58976548 |
| 1049 | Dominican Republic | 2004 | 182.4855943 | 0.524543275 | 53.58976548 |
| 1050 | Dominican Republic | 2006 | 181.7601414 | 0.54022269 | 53.31984512 |
| 1051 | Dominican Republic | 2007 | 181.8774007 | 0.548187021 | 51.75814966 |
| 1052 | Dominican Republic | 2008 | 182.0426603 | 0.555371313 | 51.75814966 |
| 1053 | Dominican Republic | 2009 | 182.1089674 | 0.561783754 | 51.75814966 |
| 1054 | Dominican Republic | 2011 | 181.0083754 | 0.573834813 | 51.75814966 |
| 1055 | Dominican Republic | 2012 | 179.6747518 | 0.578966381 | 51.75814966 |
| 1056 | Dominican Republic | 2013 | 178.1738644 | 0.583625825 | 51.75814966 |
| 1057 | Dominican Republic | 2014 | 176.9081185 | 0.588236743 | 51.75814966 |
| 1058 | Dominican Republic | 2019 | 176.6046736 | 0.611913021 | 51.75814966 |
| 1059 | Ecuador | 1991 | 210.5148171 | 0.517679421 | 53.58976548 |
| 1060 | Ecuador | 1992 | 208.3303871 | 0.51837907 | 53.58976548 |
| 1061 | Ecuador | 1994 | 205.658568 | 0.524908291 | 53.58976548 |
| 1062 | Ecuador | 1995 | 205.2803413 | 0.529380752 | 53.58976548 |
| 1063 | Ecuador | 1996 | 205.6321748 | 0.532691399 | 53.31984512 |
| 1064 | Ecuador | 1998 | 207.9902217 | 0.53719633 | 53.31984512 |
| 1065 | Ecuador | 1999 | 209.5553993 | 0.539032308 | 53.31984512 |
| 1066 | Ecuador | 2000 | 211.0733669 | 0.543044779 | 53.31984512 |
| 1067 | Ecuador | 2002 | 215.265198 | 0.554897325 | 51.75814966 |
| 1068 | Ecuador | 2003 | 217.9637287 | 0.560955485 | 51.75814966 |
| 1069 | Ecuador | 2005 | 223.1004245 | 0.569927345 | 51.75814966 |
| 1070 | Ecuador | 2007 | 228.9027541 | 0.574704702 | 51.75814966 |
| 1071 | Ecuador | 2008 | 231.9563946 | 0.57812504 | 51.75814966 |
| 1072 | Ecuador | 2009 | 234.255018 | 0.582484258 | 51.75814966 |
| 1073 | Ecuador | 2014 | 227.9404452 | 0.622027433 | 32.04043631 |
| 1074 | Ecuador | 2015 | 225.2226611 | 0.62854671 | 22.46369183 |
| 1075 | Ecuador | 2016 | 221.0258592 | 0.634281143 | 22.46369183 |
| 1076 | Ecuador | 2017 | 215.0848511 | 0.640011219 | 22.46369183 |
| 1077 | Ecuador | 2019 | 206.2267236 | 0.651787987 | 22.46369183 |
| 1078 | Ecuador | 2020 | 205.8226792 | 0.656714457 | 22.46369183 |
| 1079 | Ecuador | 2021 | 193.0301525 | 0.661017053 | 22.46369183 |
| 1080 | Egypt | 1990 | 241.4054254 | 0.417182742 | 62.92622144 |
| 1081 | Egypt | 1992 | 241.4029081 | 0.437744555 | 62.92622144 |
| 1082 | Egypt | 1994 | 240.8539697 | 0.458727004 | 62.92622144 |
| 1083 | Egypt | 1996 | 239.5918685 | 0.476116627 | 53.58976548 |
| 1084 | Egypt | 1998 | 236.4504175 | 0.489184071 | 53.58976548 |
| 1085 | Egypt | 1999 | 234.7229542 | 0.495502619 | 53.58976548 |
| 1086 | Egypt | 2000 | 233.311521 | 0.502169886 | 53.58976548 |
| 1087 | Egypt | 2002 | 230.56997 | 0.515038129 | 53.58976548 |
| 1088 | Egypt | 2004 | 227.5386944 | 0.525181678 | 53.58976548 |
| 1089 | Egypt | 2005 | 226.2638302 | 0.528665483 | 53.58976548 |
| 1090 | Egypt | 2006 | 224.954289 | 0.530395807 | 53.58976548 |
| 1091 | Egypt | 2007 | 223.4406036 | 0.529457594 | 53.58976548 |
| 1092 | Egypt | 2008 | 222.035281 | 0.524743833 | 53.58976548 |
| 1093 | Egypt | 2010 | 220.701886 | 0.507715477 | 53.58976548 |
| 1094 | Egypt | 2011 | 221.1705726 | 0.504594044 | 53.58976548 |
| 1095 | Egypt | 2012 | 222.1545699 | 0.509157068 | 53.58976548 |
| 1096 | Egypt | 2015 | 225.4017641 | 0.544370149 | 51.75814966 |
| 1097 | Egypt | 2019 | 227.3661179 | 0.587736877 | 51.75814966 |
| 1098 | Egypt | 2020 | 227.7774345 | 0.597363341 | 51.75814966 |
| 1099 | El Salvador | 1991 | 84.28525629 | 0.374855325 | 63.35324861 |
| 1100 | El Salvador | 1992 | 83.58332203 | 0.377076643 | 63.35324861 |
| 1101 | El Salvador | 1994 | 82.43792235 | 0.385149094 | 63.35324861 |
| 1102 | El Salvador | 1996 | 81.74003973 | 0.400178468 | 62.92622144 |
| 1103 | El Salvador | 1997 | 81.47307348 | 0.410455826 | 62.92622144 |
| 1104 | El Salvador | 1998 | 81.22456955 | 0.42142119 | 62.92622144 |
| 1105 | El Salvador | 2001 | 80.18284931 | 0.454177659 | 62.92622144 |
| 1106 | El Salvador | 2003 | 78.88714401 | 0.470397413 | 53.58976548 |
| 1107 | El Salvador | 2005 | 77.88161895 | 0.482041719 | 53.58976548 |
| 1108 | El Salvador | 2007 | 77.28897879 | 0.490809752 | 53.58976548 |
| 1109 | El Salvador | 2008 | 76.99618361 | 0.495077739 | 53.58976548 |
| 1110 | El Salvador | 2010 | 76.5322915 | 0.504171335 | 53.58976548 |
| 1111 | El Salvador | 2011 | 76.30458224 | 0.508969239 | 53.58976548 |
| 1112 | El Salvador | 2014 | 75.47264973 | 0.523468901 | 53.58976548 |
| 1113 | El Salvador | 2015 | 75.37773472 | 0.52938695 | 53.58976548 |
| 1114 | El Salvador | 2016 | 75.48046894 | 0.535650269 | 53.31984512 |
| 1115 | El Salvador | 2017 | 75.71784816 | 0.541998957 | 53.31984512 |
| 1116 | El Salvador | 2018 | 75.98357973 | 0.548143174 | 51.75814966 |
| 1117 | El Salvador | 2019 | 76.15490436 | 0.554031648 | 51.75814966 |
| 1118 | El Salvador | 2021 | 77.04220211 | 0.563775188 | 51.75814966 |
| 1119 | Equatorial Guinea | 1991 | 214.9754666 | 0.275296992 | 100.8275782 |
| 1120 | Equatorial Guinea | 1994 | 217.6741946 | 0.30068634 | 100.8275782 |
| 1121 | Equatorial Guinea | 1995 | 218.6220028 | 0.310443551 | 89.96318282 |
| 1122 | Equatorial Guinea | 1996 | 219.8246153 | 0.324601221 | 66.46502371 |
| 1123 | Equatorial Guinea | 1999 | 224.8203758 | 0.391128023 | 62.92622144 |
| 1124 | Equatorial Guinea | 2004 | 232.4951783 | 0.483879612 | 53.58976548 |
| 1125 | Equatorial Guinea | 2006 | 235.0577754 | 0.511579828 | 53.58976548 |
| 1126 | Equatorial Guinea | 2010 | 239.9135562 | 0.559109377 | 51.75814966 |
| 1127 | Equatorial Guinea | 2011 | 240.4880833 | 0.570277424 | 51.75814966 |
| 1128 | Equatorial Guinea | 2012 | 241.0333991 | 0.581833899 | 51.75814966 |
| 1129 | Equatorial Guinea | 2014 | 241.8311795 | 0.603473167 | 51.75814966 |
| 1130 | Equatorial Guinea | 2015 | 242.0236593 | 0.613134624 | 51.75814966 |
| 1131 | Equatorial Guinea | 2017 | 242.1678828 | 0.630234538 | 22.46369183 |
| 1132 | Equatorial Guinea | 2018 | 242.1652318 | 0.638022823 | 22.46369183 |
| 1133 | Equatorial Guinea | 2019 | 242.1825358 | 0.645303093 | 22.46369183 |
| 1134 | Equatorial Guinea | 2020 | 242.5357675 | 0.652124903 | 22.46369183 |
| 1135 | Equatorial Guinea | 2021 | 243.5611247 | 0.657857456 | 22.46369183 |
| 1136 | Eritrea | 1992 | 276.0824776 | 0.230296066 | 101.1599627 |
| 1137 | Eritrea | 1993 | 276.8613619 | 0.239385041 | 101.1599627 |
| 1138 | Eritrea | 1994 | 277.4851867 | 0.250752541 | 100.8275782 |
| 1139 | Eritrea | 1995 | 277.9058079 | 0.261498485 | 100.8275782 |
| 1140 | Eritrea | 1998 | 279.1893122 | 0.293764828 | 100.8275782 |
| 1141 | Eritrea | 1999 | 279.5274604 | 0.30314234 | 89.96318282 |
| 1142 | Eritrea | 2000 | 279.6686325 | 0.310248162 | 89.96318282 |
| 1143 | Eritrea | 2002 | 279.4948072 | 0.323566585 | 66.46502371 |
| 1144 | Eritrea | 2003 | 279.3339316 | 0.328593581 | 65.82455226 |
| 1145 | Eritrea | 2005 | 279.1429461 | 0.336682511 | 65.82455226 |
| 1146 | Eritrea | 2006 | 279.1705519 | 0.339835824 | 65.82455226 |
| 1147 | Eritrea | 2011 | 279.9824788 | 0.354163147 | 65.82455226 |
| 1148 | Eritrea | 2012 | 280.578841 | 0.360112751 | 64.06460795 |
| 1149 | Eritrea | 2015 | 282.4941512 | 0.376935162 | 63.35324861 |
| 1150 | Eritrea | 2016 | 283.0721577 | 0.382171666 | 63.35324861 |
| 1151 | Eritrea | 2018 | 284.7684589 | 0.391154884 | 62.92622144 |
| 1152 | Eritrea | 2019 | 285.5796095 | 0.396330558 | 62.92622144 |
| 1153 | Eritrea | 2021 | 288.1275374 | 0.403863943 | 62.92622144 |
| 1154 | Estonia | 1991 | 65.89736803 | 0.685223089 | 17.2540694 |
| 1155 | Estonia | 1992 | 66.83260262 | 0.695586674 | 17.2540694 |
| 1156 | Estonia | 1993 | 68.10064278 | 0.70369315 | 17.2540694 |
| 1157 | Estonia | 1995 | 71.41594267 | 0.713172725 | 17.2540694 |
| 1158 | Estonia | 1996 | 74.59854134 | 0.718813024 | 17.2540694 |
| 1159 | Estonia | 1997 | 80.01419559 | 0.72465244 | 15.06516239 |
| 1160 | Estonia | 1999 | 94.62059181 | 0.733250616 | 14.22935117 |
| 1161 | Estonia | 2001 | 110.3795018 | 0.746602207 | 13.44730416 |
| 1162 | Estonia | 2002 | 119.4020219 | 0.752629898 | 13.44730416 |
| 1163 | Estonia | 2003 | 129.2867147 | 0.757815678 | 10.45018691 |
| 1164 | Estonia | 2005 | 151.2320063 | 0.769112182 | 9.92126395 |
| 1165 | Estonia | 2006 | 164.2116045 | 0.774451842 | 9.92126395 |
| 1166 | Estonia | 2009 | 210.2360232 | 0.794728213 | 8.445315313 |
| 1167 | Estonia | 2010 | 223.9058046 | 0.800829718 | 8.445315313 |
| 1168 | Estonia | 2011 | 237.6134649 | 0.806633078 | 8.445315313 |
| 1169 | Estonia | 2012 | 252.3907411 | 0.811109358 | 8.445315313 |
| 1170 | Estonia | 2013 | 265.9119521 | 0.814887177 | 8.445315313 |
| 1171 | Estonia | 2015 | 280.3817774 | 0.823221278 | 8.445315313 |
| 1172 | Estonia | 2020 | 262.6477431 | 0.841817139 | 8.445315313 |
| 1173 | Eswatini | 1991 | 205.845096 | 0.408269969 | 62.92622144 |
| 1174 | Eswatini | 1993 | 206.1801786 | 0.424802995 | 62.92622144 |
| 1175 | Eswatini | 1995 | 206.141001 | 0.440127047 | 62.92622144 |
| 1176 | Eswatini | 1996 | 205.8230715 | 0.447399238 | 62.92622144 |
| 1177 | Eswatini | 1998 | 204.4061535 | 0.460136984 | 62.92622144 |
| 1178 | Eswatini | 1999 | 203.692057 | 0.465990845 | 62.92622144 |
| 1179 | Eswatini | 2000 | 203.2395663 | 0.471852843 | 53.58976548 |
| 1180 | Eswatini | 2001 | 202.8601241 | 0.477233374 | 53.58976548 |
| 1181 | Eswatini | 2002 | 202.3335659 | 0.482474423 | 53.58976548 |
| 1182 | Eswatini | 2004 | 201.3542882 | 0.492669159 | 53.58976548 |
| 1183 | Eswatini | 2005 | 201.1589321 | 0.498043849 | 53.58976548 |
| 1184 | Eswatini | 2008 | 201.5794866 | 0.514730896 | 53.58976548 |
| 1185 | Eswatini | 2009 | 201.8685907 | 0.520427398 | 53.58976548 |
| 1186 | Eswatini | 2011 | 202.8845791 | 0.53191391 | 53.58976548 |
| 1187 | Eswatini | 2015 | 207.3840028 | 0.555373739 | 51.75814966 |
| 1188 | Eswatini | 2017 | 208.2703845 | 0.566698493 | 51.75814966 |
| 1189 | Eswatini | 2018 | 208.6523528 | 0.572154807 | 51.75814966 |
| 1190 | Eswatini | 2021 | 210.4261555 | 0.585459713 | 51.75814966 |
| 1191 | Ethiopia | 1990 | 270.7680479 | 0.148033885 | 245.5041638 |
| 1192 | Ethiopia | 1993 | 274.0691448 | 0.151582364 | 245.5041638 |
| 1193 | Ethiopia | 1994 | 275.07408 | 0.152982637 | 245.5041638 |
| 1194 | Ethiopia | 1995 | 275.9685323 | 0.155085605 | 245.5041638 |
| 1195 | Ethiopia | 1996 | 276.8582829 | 0.158127229 | 245.5041638 |
| 1196 | Ethiopia | 1998 | 279.0058858 | 0.16507771 | 245.5041638 |
| 1197 | Ethiopia | 2000 | 280.9389289 | 0.17341853 | 245.5041638 |
| 1198 | Ethiopia | 2001 | 281.8153833 | 0.178972969 | 238.7663549 |
| 1199 | Ethiopia | 2002 | 282.9260449 | 0.184138013 | 238.2077266 |
| 1200 | Ethiopia | 2003 | 284.1417274 | 0.188694814 | 237.6563045 |
| 1201 | Ethiopia | 2004 | 285.3317011 | 0.195030701 | 237.6563045 |
| 1202 | Ethiopia | 2007 | 288.6840149 | 0.221819931 | 101.1599627 |
| 1203 | Ethiopia | 2009 | 291.5256943 | 0.244265818 | 100.8275782 |
| 1204 | Ethiopia | 2011 | 292.9449257 | 0.267880862 | 100.8275782 |
| 1205 | Ethiopia | 2012 | 293.487337 | 0.279006958 | 100.8275782 |
| 1206 | Ethiopia | 2014 | 294.4452666 | 0.300484548 | 100.8275782 |
| 1207 | Ethiopia | 2015 | 294.8984446 | 0.310736419 | 89.96318282 |
| 1208 | Ethiopia | 2016 | 295.5721735 | 0.320431867 | 66.46502371 |
| 1209 | Ethiopia | 2018 | 297.9705983 | 0.338050075 | 65.82455226 |
| 1210 | Ethiopia | 2019 | 298.987587 | 0.346422664 | 65.82455226 |
| 1211 | Ethiopia | 2020 | 300.169764 | 0.35321635 | 65.82455226 |
| 1212 | Ethiopia | 2021 | 301.9138832 | 0.358823295 | 64.06460795 |
| 1213 | Fiji | 1990 | 121.2675047 | 0.534648908 | 53.31984512 |
| 1214 | Fiji | 1991 | 121.2170718 | 0.53877826 | 53.31984512 |
| 1215 | Fiji | 1992 | 121.2319927 | 0.543188552 | 53.31984512 |
| 1216 | Fiji | 1993 | 121.3031392 | 0.547776474 | 51.75814966 |
| 1217 | Fiji | 1994 | 121.4335316 | 0.55284902 | 51.75814966 |
| 1218 | Fiji | 1996 | 121.9513084 | 0.565041879 | 51.75814966 |
| 1219 | Fiji | 1997 | 122.3232159 | 0.571187753 | 51.75814966 |
| 1220 | Fiji | 1998 | 122.6586023 | 0.577172354 | 51.75814966 |
| 1221 | Fiji | 2000 | 123.0914668 | 0.589196631 | 51.75814966 |
| 1222 | Fiji | 2001 | 122.95264 | 0.594497419 | 51.75814966 |
| 1223 | Fiji | 2003 | 121.9289597 | 0.603489622 | 51.75814966 |
| 1224 | Fiji | 2009 | 118.2429221 | 0.620883465 | 32.04043631 |
| 1225 | Fiji | 2010 | 117.6403795 | 0.623308786 | 32.04043631 |
| 1226 | Fiji | 2011 | 116.942952 | 0.626438251 | 32.04043631 |
| 1227 | Fiji | 2013 | 115.3245186 | 0.634278114 | 22.46369183 |
| 1228 | Fiji | 2014 | 114.7255412 | 0.639390066 | 22.46369183 |
| 1229 | Fiji | 2015 | 114.4816154 | 0.645026353 | 22.46369183 |
| 1230 | Fiji | 2017 | 114.9429268 | 0.65646401 | 22.46369183 |
| 1231 | Fiji | 2019 | 115.7208123 | 0.667060979 | 22.46369183 |
| 1232 | Fiji | 2021 | 117.4449287 | 0.675051631 | 22.03195463 |
| 1233 | Finland | 1991 | 37.35720684 | 0.758410687 | 9.92126395 |
| 1234 | Finland | 1992 | 38.30513893 | 0.7608041 | 9.92126395 |
| 1235 | Finland | 1995 | 42.02953916 | 0.771163742 | 9.92126395 |
| 1236 | Finland | 1997 | 45.56897004 | 0.778814215 | 9.92126395 |
| 1237 | Finland | 1999 | 50.17147496 | 0.785773988 | 8.445315313 |
| 1238 | Finland | 2002 | 58.54911987 | 0.798770274 | 8.445315313 |
| 1239 | Finland | 2003 | 61.93750452 | 0.802325431 | 8.445315313 |
| 1240 | Finland | 2004 | 65.53308806 | 0.805694227 | 8.445315313 |
| 1241 | Finland | 2005 | 69.23782774 | 0.808936787 | 8.445315313 |
| 1242 | Finland | 2006 | 73.31653155 | 0.812081482 | 8.445315313 |
| 1243 | Finland | 2008 | 82.71041662 | 0.818875544 | 8.445315313 |
| 1244 | Finland | 2009 | 87.53852595 | 0.821457588 | 8.445315313 |
| 1245 | Finland | 2010 | 92.09912261 | 0.824844982 | 8.445315313 |
| 1246 | Finland | 2011 | 96.45514739 | 0.828270577 | 8.445315313 |
| 1247 | Finland | 2014 | 109.4611101 | 0.837431872 | 8.445315313 |
| 1248 | Finland | 2015 | 113.6551023 | 0.840878477 | 8.445315313 |
| 1249 | Finland | 2016 | 118.6690788 | 0.844622338 | 8.445315313 |
| 1250 | Finland | 2017 | 124.3770645 | 0.848586128 | 8.445315313 |
| 1251 | Finland | 2018 | 129.0982315 | 0.852202943 | 8.445315313 |
| 1252 | Finland | 2020 | 113.1378326 | 0.857655553 | 8.445315313 |
| 1253 | Finland | 2021 | 107.5958836 | 0.859831368 | 8.445315313 |
| 1254 | France | 1990 | 118.9140091 | 0.730747466 | 14.22935117 |
| 1255 | France | 1991 | 123.3715931 | 0.736452382 | 13.44730416 |
| 1256 | France | 1993 | 130.9167781 | 0.748582327 | 13.44730416 |
| 1257 | France | 1994 | 133.5186075 | 0.753176132 | 13.44730416 |
| 1258 | France | 1996 | 135.6597403 | 0.760899579 | 9.92126395 |
| 1259 | France | 1998 | 136.4165515 | 0.767852561 | 9.92126395 |
| 1260 | France | 1999 | 136.8553546 | 0.770304363 | 9.92126395 |
| 1261 | France | 2000 | 137.5662877 | 0.772792033 | 9.92126395 |
| 1262 | France | 2001 | 140.0022688 | 0.776225157 | 9.92126395 |
| 1263 | France | 2002 | 144.5898786 | 0.780088872 | 9.92126395 |
| 1264 | France | 2003 | 149.945983 | 0.783178757 | 8.445315313 |
| 1265 | France | 2005 | 157.446718 | 0.789042086 | 8.445315313 |
| 1266 | France | 2006 | 157.499953 | 0.79232192 | 8.445315313 |
| 1267 | France | 2008 | 153.1201274 | 0.79817743 | 8.445315313 |
| 1268 | France | 2009 | 150.6423489 | 0.80050867 | 8.445315313 |
| 1269 | France | 2010 | 149.2833254 | 0.803240247 | 8.445315313 |
| 1270 | France | 2012 | 148.9488547 | 0.809615389 | 8.445315313 |
| 1271 | France | 2013 | 149.0390894 | 0.812984015 | 8.445315313 |
| 1272 | France | 2014 | 149.1080329 | 0.816551448 | 8.445315313 |
| 1273 | France | 2015 | 149.00543 | 0.820238385 | 8.445315313 |
| 1274 | France | 2017 | 147.6737877 | 0.827098441 | 8.445315313 |
| 1275 | France | 2020 | 146.5904845 | 0.836049443 | 8.445315313 |
| 1276 | France | 2021 | 145.1431033 | 0.838364875 | 8.445315313 |
| 1277 | Gabon | 1991 | 242.6371438 | 0.461563098 | 62.92622144 |
| 1278 | Gabon | 1992 | 242.8760756 | 0.467024048 | 53.58976548 |
| 1279 | Gabon | 1993 | 243.0810249 | 0.472394463 | 53.58976548 |
| 1280 | Gabon | 1995 | 243.3250474 | 0.483826218 | 53.58976548 |
| 1281 | Gabon | 1996 | 243.4214481 | 0.48946146 | 53.58976548 |
| 1282 | Gabon | 1999 | 243.7396713 | 0.50480635 | 53.58976548 |
| 1283 | Gabon | 2001 | 244.1007149 | 0.513045709 | 53.58976548 |
| 1284 | Gabon | 2003 | 244.9787621 | 0.521051412 | 53.58976548 |
| 1285 | Gabon | 2004 | 245.4408494 | 0.525479071 | 53.58976548 |
| 1286 | Gabon | 2006 | 246.2611003 | 0.534797716 | 53.31984512 |
| 1287 | Gabon | 2007 | 246.9747982 | 0.539441424 | 53.31984512 |
| 1288 | Gabon | 2008 | 247.7789829 | 0.544110892 | 51.75814966 |
| 1289 | Gabon | 2010 | 248.9868902 | 0.554022706 | 51.75814966 |
| 1290 | Gabon | 2011 | 249.4382357 | 0.560232082 | 51.75814966 |
| 1291 | Gabon | 2012 | 250.0320837 | 0.566842964 | 51.75814966 |
| 1292 | Gabon | 2014 | 251.2266686 | 0.580915656 | 51.75814966 |
| 1293 | Gabon | 2015 | 251.6392505 | 0.588365 | 51.75814966 |
| 1294 | Gabon | 2016 | 252.1403394 | 0.596062305 | 51.75814966 |
| 1295 | Gabon | 2019 | 253.8549463 | 0.619904727 | 32.04043631 |
| 1296 | Gabon | 2020 | 254.0373975 | 0.627609647 | 22.46369183 |
| 1297 | Gabon | 2021 | 254.8114832 | 0.634691393 | 22.46369183 |
| 1298 | Gambia | 1990 | 256.7336663 | 0.238714846 | 101.1599627 |
| 1299 | Gambia | 1991 | 256.4185916 | 0.245010164 | 100.8275782 |
| 1300 | Gambia | 1992 | 256.1013378 | 0.251087863 | 100.8275782 |
| 1301 | Gambia | 1993 | 255.807441 | 0.256996141 | 100.8275782 |
| 1302 | Gambia | 1994 | 255.562431 | 0.262424792 | 100.8275782 |
| 1303 | Gambia | 1995 | 255.3797681 | 0.267696507 | 100.8275782 |
| 1304 | Gambia | 1996 | 255.1299795 | 0.27295019 | 100.8275782 |
| 1305 | Gambia | 1998 | 254.33311 | 0.283946056 | 100.8275782 |
| 1306 | Gambia | 2001 | 254.0058136 | 0.302543257 | 89.96318282 |
| 1307 | Gambia | 2002 | 254.3935936 | 0.307712927 | 89.96318282 |
| 1308 | Gambia | 2003 | 254.8882748 | 0.312930916 | 67.91401256 |
| 1309 | Gambia | 2010 | 257.9871622 | 0.34799783 | 65.82455226 |
| 1310 | Gambia | 2012 | 259.3772897 | 0.35837365 | 64.06460795 |
| 1311 | Gambia | 2014 | 261.0951351 | 0.3699013 | 64.06460795 |
| 1312 | Gambia | 2015 | 261.4975464 | 0.375840485 | 63.35324861 |
| 1313 | Gambia | 2018 | 261.1180871 | 0.393155679 | 62.92622144 |
| 1314 | Gambia | 2020 | 262.4703531 | 0.404524194 | 62.92622144 |
| 1315 | Georgia | 1990 | 78.28672785 | 0.656136044 | 22.46369183 |
| 1316 | Georgia | 1991 | 77.25538105 | 0.662113186 | 22.46369183 |
| 1317 | Georgia | 1992 | 76.72781418 | 0.665021428 | 22.46369183 |
| 1318 | Georgia | 1992 | 76.72781418 | 0.748286271 | 13.44730416 |
| 1319 | Georgia | 1993 | 76.57758519 | 0.663466862 | 22.46369183 |
| 1320 | Georgia | 1993 | 76.57758519 | 0.752772815 | 13.44730416 |
| 1321 | Georgia | 1995 | 76.94211024 | 0.651363604 | 22.46369183 |
| 1322 | Georgia | 1996 | 79.84416008 | 0.761496988 | 9.92126395 |
| 1323 | Georgia | 1997 | 86.80148458 | 0.638525631 | 22.46369183 |
| 1324 | Georgia | 1998 | 96.08812997 | 0.633347931 | 22.46369183 |
| 1325 | Georgia | 1998 | 96.08812997 | 0.764068933 | 9.92126395 |
| 1326 | Georgia | 1999 | 105.9855615 | 0.630277946 | 22.46369183 |
| 1327 | Georgia | 1999 | 105.9855615 | 0.765608241 | 9.92126395 |
| 1328 | Georgia | 2000 | 114.7730212 | 0.768348485 | 9.92126395 |
| 1329 | Georgia | 2001 | 122.3383051 | 0.6333883 | 22.46369183 |
| 1330 | Georgia | 2002 | 129.8560256 | 0.638370108 | 22.46369183 |
| 1331 | Georgia | 2002 | 129.8560256 | 0.774881297 | 9.92126395 |
| 1332 | Georgia | 2004 | 145.6016439 | 0.779001306 | 9.92126395 |
| 1333 | Georgia | 2005 | 154.2493539 | 0.653616005 | 22.46369183 |
| 1334 | Georgia | 2005 | 154.2493539 | 0.779955806 | 9.92126395 |
| 1335 | Georgia | 2006 | 165.4335792 | 0.658403372 | 22.46369183 |
| 1336 | Georgia | 2007 | 179.1464565 | 0.663697966 | 22.46369183 |
| 1337 | Georgia | 2008 | 192.6362523 | 0.668442496 | 22.46369183 |
| 1338 | Georgia | 2011 | 207.4964012 | 0.681567044 | 17.2540694 |
| 1339 | Georgia | 2011 | 207.4964012 | 0.81150306 | 8.445315313 |
| 1340 | Georgia | 2013 | 200.821472 | 0.82006035 | 8.445315313 |
| 1341 | Georgia | 2014 | 197.1766924 | 0.692155207 | 17.2540694 |
| 1342 | Georgia | 2015 | 195.0321005 | 0.697316133 | 17.2540694 |
| 1343 | Georgia | 2015 | 195.0321005 | 0.827363984 | 8.445315313 |
| 1344 | Georgia | 2017 | 193.6113167 | 0.710874718 | 17.2540694 |
| 1345 | Georgia | 2017 | 193.6113167 | 0.834853877 | 8.445315313 |
| 1346 | Georgia | 2018 | 193.1654185 | 0.838524146 | 8.445315313 |
| 1347 | Georgia | 2019 | 192.8029242 | 0.724536462 | 15.06516239 |
| 1348 | Georgia | 2019 | 192.8029242 | 0.842234949 | 8.445315313 |
| 1349 | Georgia | 2020 | 190.8547949 | 0.729125889 | 14.22935117 |
| 1350 | Georgia | 2020 | 190.8547949 | 0.844594925 | 8.445315313 |
| 1351 | Georgia | 2021 | 152.157834 | 0.84656427 | 8.445315313 |
| 1352 | Germany | 1992 | 84.17944856 | 0.828323503 | 8.445315313 |
| 1353 | Germany | 1993 | 85.89264922 | 0.832286809 | 8.445315313 |
| 1354 | Germany | 1994 | 86.99492541 | 0.835561963 | 8.445315313 |
| 1355 | Germany | 1995 | 87.41820006 | 0.837643152 | 8.445315313 |
| 1356 | Germany | 1996 | 86.96779963 | 0.839188129 | 8.445315313 |
| 1357 | Germany | 1998 | 84.48265376 | 0.843854978 | 8.445315313 |
| 1358 | Germany | 1999 | 83.46305775 | 0.846232398 | 8.445315313 |
| 1359 | Germany | 2000 | 83.28347948 | 0.848925756 | 8.445315313 |
| 1360 | Germany | 2001 | 88.08701521 | 0.852070413 | 8.445315313 |
| 1361 | Germany | 2002 | 99.17842183 | 0.855066793 | 8.445315313 |
| 1362 | Germany | 2004 | 125.5431211 | 0.860240333 | 8.445315313 |
| 1363 | Germany | 2005 | 133.5725215 | 0.862894178 | 8.445315313 |
| 1364 | Germany | 2009 | 146.2317518 | 0.875087911 | 8.445315313 |
| 1365 | Germany | 2010 | 148.5750275 | 0.87794671 | 8.445315313 |
| 1366 | Germany | 2011 | 151.2048056 | 0.881027508 | 8.445315313 |
| 1367 | Germany | 2013 | 156.1746424 | 0.885926953 | 8.445315313 |
| 1368 | Germany | 2014 | 158.2294925 | 0.887651614 | 8.445315313 |
| 1369 | Germany | 2015 | 159.7125489 | 0.888867854 | 8.445315313 |
| 1370 | Germany | 2017 | 162.8358648 | 0.894050472 | 8.445315313 |
| 1371 | Germany | 2018 | 164.1501371 | 0.896975545 | 8.445315313 |
| 1372 | Germany | 2019 | 164.5519087 | 0.899703158 | 8.445315313 |
| 1373 | Germany | 2020 | 136.0096357 | 0.901438614 | 8.445315313 |
| 1374 | Ghana | 1990 | 273.9377456 | 0.373112005 | 64.06460795 |
| 1375 | Ghana | 1991 | 274.968702 | 0.379239256 | 63.35324861 |
| 1376 | Ghana | 1992 | 275.8427147 | 0.384856767 | 63.35324861 |
| 1377 | Ghana | 1993 | 276.5254785 | 0.389968494 | 62.92622144 |
| 1378 | Ghana | 1995 | 277.1819779 | 0.399930204 | 62.92622144 |
| 1379 | Ghana | 1997 | 276.8123755 | 0.410038073 | 62.92622144 |
| 1380 | Ghana | 1998 | 276.4320675 | 0.414888532 | 62.92622144 |
| 1381 | Ghana | 2000 | 275.8868598 | 0.424725212 | 62.92622144 |
| 1382 | Ghana | 2002 | 275.7012451 | 0.434104128 | 62.92622144 |
| 1383 | Ghana | 2003 | 275.567696 | 0.439118213 | 62.92622144 |
| 1384 | Ghana | 2004 | 275.3976824 | 0.444236977 | 62.92622144 |
| 1385 | Ghana | 2005 | 275.1953014 | 0.44936002 | 62.92622144 |
| 1386 | Ghana | 2006 | 274.8412159 | 0.454692737 | 62.92622144 |
| 1387 | Ghana | 2010 | 272.8845452 | 0.480777892 | 53.58976548 |
| 1388 | Ghana | 2011 | 272.7678294 | 0.489020974 | 53.58976548 |
| 1389 | Ghana | 2015 | 272.8936258 | 0.521539202 | 53.58976548 |
| 1390 | Ghana | 2016 | 273.1053897 | 0.528629586 | 53.58976548 |
| 1391 | Ghana | 2017 | 273.5498042 | 0.53616994 | 53.31984512 |
| 1392 | Ghana | 2018 | 274.0855766 | 0.543840539 | 53.31984512 |
| 1393 | Ghana | 2019 | 274.5721576 | 0.551632027 | 51.75814966 |
| 1394 | Greece | 1990 | 40.20704961 | 0.674186465 | 22.03195463 |
| 1395 | Greece | 1991 | 40.51805775 | 0.68031648 | 17.2540694 |
| 1396 | Greece | 1992 | 40.86296618 | 0.687055389 | 17.2540694 |
| 1397 | Greece | 1993 | 41.22459428 | 0.693328926 | 17.2540694 |
| 1398 | Greece | 1995 | 41.91966556 | 0.704862873 | 17.2540694 |
| 1399 | Greece | 1997 | 42.95394506 | 0.715687268 | 17.2540694 |
| 1400 | Greece | 1998 | 43.60156042 | 0.721593982 | 15.06516239 |
| 1401 | Greece | 2001 | 44.57075062 | 0.737154008 | 13.44730416 |
| 1402 | Greece | 2003 | 43.37788644 | 0.747185956 | 13.44730416 |
| 1403 | Greece | 2004 | 42.69504133 | 0.752418607 | 13.44730416 |
| 1404 | Greece | 2005 | 42.30163505 | 0.756384396 | 10.45018691 |
| 1405 | Greece | 2007 | 41.91789631 | 0.763927173 | 9.92126395 |
| 1406 | Greece | 2008 | 41.73839407 | 0.767527658 | 9.92126395 |
| 1407 | Greece | 2010 | 41.61198435 | 0.775155815 | 9.92126395 |
| 1408 | Greece | 2011 | 41.77333602 | 0.778525867 | 9.92126395 |
| 1409 | Greece | 2014 | 42.72510637 | 0.782442013 | 8.445315313 |
| 1410 | Greece | 2016 | 42.76018596 | 0.782798443 | 8.445315313 |
| 1411 | Greece | 2017 | 42.45078993 | 0.784108594 | 8.445315313 |
| 1412 | Greece | 2019 | 41.94304822 | 0.788138138 | 8.445315313 |
| 1413 | Greece | 2021 | 41.10969279 | 0.791854408 | 8.445315313 |
| 1414 | Greenland | 1990 | 63.63651564 | 0.732258252 | 14.22935117 |
| 1415 | Greenland | 1991 | 64.02330692 | 0.730626532 | 14.22935117 |
| 1416 | Greenland | 1992 | 64.5062737 | 0.729781103 | 14.22935117 |
| 1417 | Greenland | 1993 | 65.24172489 | 0.729894842 | 14.22935117 |
| 1418 | Greenland | 1996 | 67.48225657 | 0.730216225 | 14.22935117 |
| 1419 | Greenland | 1998 | 70.1794774 | 0.733242588 | 14.22935117 |
| 1420 | Greenland | 2001 | 74.71251604 | 0.746289605 | 13.44730416 |
| 1421 | Greenland | 2002 | 76.47911159 | 0.752878371 | 13.44730416 |
| 1422 | Greenland | 2003 | 78.30312563 | 0.759444152 | 9.92126395 |
| 1423 | Greenland | 2004 | 79.81993193 | 0.765401567 | 9.92126395 |
| 1424 | Greenland | 2005 | 81.40831979 | 0.77209958 | 9.92126395 |
| 1425 | Greenland | 2006 | 82.72820269 | 0.779141406 | 9.92126395 |
| 1426 | Greenland | 2007 | 84.02785165 | 0.785321922 | 8.445315313 |
| 1427 | Greenland | 2008 | 85.30515227 | 0.791441257 | 8.445315313 |
| 1428 | Greenland | 2010 | 86.3156662 | 0.803003131 | 8.445315313 |
| 1429 | Greenland | 2011 | 86.62771531 | 0.808105185 | 8.445315313 |
| 1430 | Greenland | 2012 | 86.72947517 | 0.811684562 | 8.445315313 |
| 1431 | Greenland | 2014 | 86.36683516 | 0.81652706 | 8.445315313 |
| 1432 | Greenland | 2015 | 86.1559171 | 0.817719189 | 8.445315313 |
| 1433 | Greenland | 2017 | 85.62634401 | 0.819781101 | 8.445315313 |
| 1434 | Greenland | 2020 | 84.96050938 | 0.824359028 | 8.445315313 |
| 1435 | Grenada | 1990 | 175.177131 | 0.436734419 | 62.92622144 |
| 1436 | Grenada | 1991 | 176.5106626 | 0.445887953 | 62.92622144 |
| 1437 | Grenada | 1993 | 179.2108766 | 0.46599489 | 62.92622144 |
| 1438 | Grenada | 1994 | 180.4628297 | 0.476410341 | 53.58976548 |
| 1439 | Grenada | 1996 | 182.6331983 | 0.496589808 | 53.58976548 |
| 1440 | Grenada | 1997 | 183.7473687 | 0.506217602 | 53.58976548 |
| 1441 | Grenada | 1998 | 184.9433972 | 0.516590019 | 53.58976548 |
| 1442 | Grenada | 2001 | 189.6314731 | 0.54905897 | 51.75814966 |
| 1443 | Grenada | 2002 | 192.3812849 | 0.559473128 | 51.75814966 |
| 1444 | Grenada | 2005 | 201.4028923 | 0.587158877 | 51.75814966 |
| 1445 | Grenada | 2006 | 203.8714949 | 0.59405452 | 51.75814966 |
| 1446 | Grenada | 2007 | 206.4288543 | 0.600766336 | 51.75814966 |
| 1447 | Grenada | 2008 | 208.932692 | 0.606879282 | 51.75814966 |
| 1448 | Grenada | 2011 | 215.1166703 | 0.620978521 | 32.04043631 |
| 1449 | Grenada | 2012 | 217.0078509 | 0.624977512 | 32.04043631 |
| 1450 | Grenada | 2014 | 220.526519 | 0.633734696 | 22.46369183 |
| 1451 | Grenada | 2015 | 222.0207992 | 0.638936084 | 22.46369183 |
| 1452 | Grenada | 2016 | 223.7299016 | 0.644335234 | 22.46369183 |
| 1453 | Grenada | 2019 | 229.1430184 | 0.660808512 | 22.46369183 |
| 1454 | Grenada | 2020 | 229.7272136 | 0.665086347 | 22.46369183 |
| 1455 | Grenada | 2021 | 219.6857712 | 0.668993028 | 22.46369183 |
| 1456 | Guam | 1992 | 202.1770168 | 0.66635634 | 22.46369183 |
| 1457 | Guam | 1994 | 193.2790298 | 0.672111712 | 22.03195463 |
| 1458 | Guam | 1996 | 185.7417778 | 0.684173735 | 17.2540694 |
| 1459 | Guam | 1999 | 175.9201974 | 0.713641593 | 17.2540694 |
| 1460 | Guam | 2000 | 171.8448671 | 0.724802357 | 15.06516239 |
| 1461 | Guam | 2001 | 166.1805126 | 0.734267376 | 14.22935117 |
| 1462 | Guam | 2003 | 151.3442773 | 0.74591153 | 13.44730416 |
| 1463 | Guam | 2004 | 144.6081876 | 0.74901652 | 13.44730416 |
| 1464 | Guam | 2005 | 139.9587526 | 0.750865875 | 13.44730416 |
| 1465 | Guam | 2006 | 137.0973775 | 0.751176245 | 13.44730416 |
| 1466 | Guam | 2008 | 132.739285 | 0.755152408 | 10.45018691 |
| 1467 | Guam | 2010 | 128.7656652 | 0.761484317 | 9.92126395 |
| 1468 | Guam | 2012 | 123.5785061 | 0.766845984 | 9.92126395 |
| 1469 | Guam | 2013 | 120.8874938 | 0.770224957 | 9.92126395 |
| 1470 | Guam | 2014 | 118.6527169 | 0.773441684 | 9.92126395 |
| 1471 | Guam | 2015 | 117.28635 | 0.776723614 | 9.92126395 |
| 1472 | Guam | 2017 | 116.2657858 | 0.78555492 | 8.445315313 |
| 1473 | Guam | 2021 | 119.6517204 | 0.803982203 | 8.445315313 |
| 1474 | Guatemala | 1990 | 67.91401256 | 0.311792455 | 67.91401256 |
| 1475 | Guatemala | 1991 | 67.17014806 | 0.314329484 | 67.17014806 |
| 1476 | Guatemala | 1992 | 66.46502371 | 0.319165481 | 66.46502371 |
| 1477 | Guatemala | 1993 | 65.82455226 | 0.326840819 | 65.82455226 |
| 1478 | Guatemala | 1997 | 64.06460795 | 0.355995963 | 64.06460795 |
| 1479 | Guatemala | 1999 | 63.35324861 | 0.374455316 | 63.35324861 |
| 1480 | Guatemala | 2001 | 62.92622144 | 0.386927686 | 62.92622144 |
| 1481 | Guatemala | 2004 | 62.98038112 | 0.419777065 | 62.92622144 |
| 1482 | Guatemala | 2005 | 63.11084654 | 0.430327529 | 62.92622144 |
| 1483 | Guatemala | 2006 | 63.31618568 | 0.438856376 | 62.92622144 |
| 1484 | Guatemala | 2009 | 64.1478119 | 0.464003077 | 62.92622144 |
| 1485 | Guatemala | 2012 | 64.1436119 | 0.481862853 | 53.58976548 |
| 1486 | Guatemala | 2013 | 63.96017307 | 0.487286692 | 53.58976548 |
| 1487 | Guatemala | 2015 | 63.72798222 | 0.500655607 | 53.58976548 |
| 1488 | Guatemala | 2016 | 63.77779609 | 0.507149792 | 53.58976548 |
| 1489 | Guatemala | 2017 | 63.89097838 | 0.513735682 | 53.58976548 |
| 1490 | Guatemala | 2018 | 64.01747297 | 0.520995469 | 53.58976548 |
| 1491 | Guatemala | 2021 | 63.9675962 | 0.539972424 | 53.31984512 |
| 1492 | Guinea | 1990 | 269.3542327 | 0.178295421 | 238.7663549 |
| 1493 | Guinea | 1991 | 269.4215715 | 0.181459391 | 238.7663549 |
| 1494 | Guinea | 1993 | 269.3117062 | 0.187686242 | 237.6563045 |
| 1495 | Guinea | 1994 | 269.1461032 | 0.191128837 | 237.6563045 |
| 1496 | Guinea | 1995 | 268.9299536 | 0.194573245 | 237.6563045 |
| 1497 | Guinea | 1996 | 268.5664203 | 0.198633755 | 236.3318337 |
| 1498 | Guinea | 1998 | 267.3795281 | 0.208003383 | 101.1599627 |
| 1499 | Guinea | 1999 | 266.8351295 | 0.212412705 | 101.1599627 |
| 1500 | Guinea | 2000 | 266.5085759 | 0.216649792 | 101.1599627 |
| 1501 | Guinea | 2003 | 266.9880751 | 0.230522594 | 101.1599627 |
| 1502 | Guinea | 2004 | 267.2470537 | 0.23513497 | 101.1599627 |
| 1503 | Guinea | 2005 | 267.3355821 | 0.239691845 | 101.1599627 |
| 1504 | Guinea | 2006 | 267.2738755 | 0.244010127 | 100.8275782 |
| 1505 | Guinea | 2011 | 266.9085586 | 0.2676246 | 100.8275782 |
| 1506 | Guinea | 2012 | 266.9609301 | 0.273428319 | 100.8275782 |
| 1507 | Guinea | 2014 | 267.1435213 | 0.285961264 | 100.8275782 |
| 1508 | Guinea | 2015 | 267.2711209 | 0.292363614 | 100.8275782 |
| 1509 | Guinea | 2016 | 267.5965905 | 0.299112412 | 100.8275782 |
| 1510 | Guinea | 2018 | 268.6996538 | 0.314839588 | 67.17014806 |
| 1511 | Guinea | 2019 | 269.2640386 | 0.322839848 | 66.46502371 |
| 1512 | Guinea | 2020 | 270.9210599 | 0.329877997 | 65.82455226 |
| 1513 | Guinea-Bissau | 1990 | 244.6096329 | 0.207614839 | 101.1599627 |
| 1514 | Guinea-Bissau | 1991 | 244.9232445 | 0.212505984 | 101.1599627 |
| 1515 | Guinea-Bissau | 1993 | 245.3729705 | 0.221678978 | 101.1599627 |
| 1516 | Guinea-Bissau | 1994 | 245.4908687 | 0.226434933 | 101.1599627 |
| 1517 | Guinea-Bissau | 1996 | 245.4682213 | 0.236106374 | 101.1599627 |
| 1518 | Guinea-Bissau | 1997 | 245.3648635 | 0.241360584 | 100.8275782 |
| 1519 | Guinea-Bissau | 1998 | 245.2452629 | 0.244371797 | 100.8275782 |
| 1520 | Guinea-Bissau | 1999 | 245.1357104 | 0.248217317 | 100.8275782 |
| 1521 | Guinea-Bissau | 2002 | 245.5286051 | 0.259321816 | 100.8275782 |
| 1522 | Guinea-Bissau | 2003 | 245.9363181 | 0.262747724 | 100.8275782 |
| 1523 | Guinea-Bissau | 2005 | 246.3493427 | 0.270056132 | 100.8275782 |
| 1524 | Guinea-Bissau | 2007 | 246.0204959 | 0.277805017 | 100.8275782 |
| 1525 | Guinea-Bissau | 2008 | 245.8266866 | 0.282151502 | 100.8275782 |
| 1526 | Guinea-Bissau | 2009 | 245.6967883 | 0.286423345 | 100.8275782 |
| 1527 | Guinea-Bissau | 2010 | 245.6987889 | 0.291141639 | 100.8275782 |
| 1528 | Guinea-Bissau | 2012 | 246.5832847 | 0.301562342 | 89.96318282 |
| 1529 | Guinea-Bissau | 2015 | 248.2592728 | 0.317090015 | 67.17014806 |
| 1530 | Guinea-Bissau | 2016 | 248.3997522 | 0.32297967 | 66.46502371 |
| 1531 | Guinea-Bissau | 2017 | 248.5183138 | 0.329326492 | 65.82455226 |
| 1532 | Guinea-Bissau | 2018 | 248.6559682 | 0.335667833 | 65.82455226 |
| 1533 | Guinea-Bissau | 2020 | 250.4843073 | 0.347798998 | 65.82455226 |
| 1534 | Guyana | 1991 | 250.4146009 | 0.462677069 | 62.92622144 |
| 1535 | Guyana | 1992 | 252.3605933 | 0.466753989 | 53.58976548 |
| 1536 | Guyana | 1997 | 250.4096984 | 0.504362779 | 53.58976548 |
| 1537 | Guyana | 1999 | 243.7737808 | 0.521595978 | 53.58976548 |
| 1538 | Guyana | 2000 | 242.4672532 | 0.528693992 | 53.58976548 |
| 1539 | Guyana | 2001 | 242.9405547 | 0.534869806 | 53.31984512 |
| 1540 | Guyana | 2004 | 246.9735621 | 0.55032637 | 51.75814966 |
| 1541 | Guyana | 2005 | 247.7233603 | 0.554844797 | 51.75814966 |
| 1542 | Guyana | 2006 | 247.4991191 | 0.559465611 | 51.75814966 |
| 1543 | Guyana | 2008 | 245.383623 | 0.569595206 | 51.75814966 |
| 1544 | Guyana | 2011 | 246.315598 | 0.585688269 | 51.75814966 |
| 1545 | Guyana | 2012 | 249.7141687 | 0.591336582 | 51.75814966 |
| 1546 | Guyana | 2016 | 262.744504 | 0.61391436 | 51.75814966 |
| 1547 | Guyana | 2019 | 267.6561694 | 0.633640347 | 22.46369183 |
| 1548 | Guyana | 2020 | 268.0833851 | 0.642284645 | 22.46369183 |
| 1549 | Guyana | 2021 | 264.7823014 | 0.650812335 | 22.46369183 |
| 1550 | Haiti | 1990 | 159.6964381 | 0.31033463 | 89.96318282 |
| 1551 | Haiti | 1991 | 159.5166933 | 0.315455441 | 67.17014806 |
| 1552 | Haiti | 1992 | 159.4375304 | 0.320389979 | 66.46502371 |
| 1553 | Haiti | 1993 | 159.4621114 | 0.324895851 | 66.46502371 |
| 1554 | Haiti | 1995 | 159.7619552 | 0.332999152 | 65.82455226 |
| 1555 | Haiti | 1996 | 160.2060398 | 0.338229396 | 65.82455226 |
| 1556 | Haiti | 1997 | 160.9477778 | 0.343880049 | 65.82455226 |
| 1557 | Haiti | 1998 | 161.7919563 | 0.349698073 | 65.82455226 |
| 1558 | Haiti | 1999 | 162.5233687 | 0.355980743 | 65.82455226 |
| 1559 | Haiti | 2002 | 162.9117342 | 0.374473325 | 63.35324861 |
| 1560 | Haiti | 2003 | 162.7051689 | 0.380067255 | 63.35324861 |
| 1561 | Haiti | 2004 | 162.4605806 | 0.385061512 | 63.35324861 |
| 1562 | Haiti | 2005 | 162.2471737 | 0.38984542 | 62.92622144 |
| 1563 | Haiti | 2006 | 162.0267148 | 0.394322001 | 62.92622144 |
| 1564 | Haiti | 2007 | 161.7438583 | 0.398973441 | 62.92622144 |
| 1565 | Haiti | 2009 | 161.1365917 | 0.407944424 | 62.92622144 |
| 1566 | Haiti | 2010 | 160.8977798 | 0.411840105 | 62.92622144 |
| 1567 | Haiti | 2013 | 160.3291225 | 0.423985549 | 62.92622144 |
| 1568 | Haiti | 2015 | 160.1033895 | 0.43175676 | 62.92622144 |
| 1569 | Haiti | 2016 | 160.0633837 | 0.435288722 | 62.92622144 |
| 1570 | Haiti | 2017 | 160.0466201 | 0.438541003 | 62.92622144 |
| 1571 | Haiti | 2021 | 160.5711745 | 0.448278285 | 62.92622144 |
| 1572 | Honduras | 1991 | 194.4885121 | 0.337034649 | 65.82455226 |
| 1573 | Honduras | 1995 | 192.3232949 | 0.358787268 | 64.06460795 |
| 1574 | Honduras | 1997 | 192.7162416 | 0.369983894 | 64.06460795 |
| 1575 | Honduras | 1999 | 193.3807673 | 0.381752316 | 63.35324861 |
| 1576 | Honduras | 2002 | 192.746572 | 0.400371798 | 62.92622144 |
| 1577 | Honduras | 2003 | 192.1083134 | 0.406668381 | 62.92622144 |
| 1578 | Honduras | 2005 | 191.0459545 | 0.419612332 | 62.92622144 |
| 1579 | Honduras | 2006 | 190.6418589 | 0.426352997 | 62.92622144 |
| 1580 | Honduras | 2008 | 189.5500247 | 0.439998164 | 62.92622144 |
| 1581 | Honduras | 2010 | 188.553117 | 0.452382226 | 62.92622144 |
| 1582 | Honduras | 2011 | 188.0012247 | 0.458700329 | 62.92622144 |
| 1583 | Honduras | 2014 | 185.79407 | 0.476587799 | 53.58976548 |
| 1584 | Honduras | 2015 | 185.4711156 | 0.482294176 | 53.58976548 |
| 1585 | Honduras | 2016 | 185.8496003 | 0.487705932 | 53.58976548 |
| 1586 | Honduras | 2018 | 187.9471319 | 0.498657227 | 53.58976548 |
| 1587 | Honduras | 2019 | 188.6015154 | 0.504156505 | 53.58976548 |
| 1588 | Honduras | 2021 | 195.0327416 | 0.513037248 | 53.58976548 |
| 1589 | Hungary | 1990 | 95.97172508 | 0.649419991 | 22.46369183 |
| 1590 | Hungary | 1993 | 95.24291049 | 0.667543556 | 22.46369183 |
| 1591 | Hungary | 1994 | 95.18547446 | 0.675051406 | 22.03195463 |
| 1592 | Hungary | 1995 | 95.19282119 | 0.683186412 | 17.2540694 |
| 1593 | Hungary | 1997 | 95.89050242 | 0.697935514 | 17.2540694 |
| 1594 | Hungary | 2000 | 98.17312971 | 0.715805281 | 17.2540694 |
| 1595 | Hungary | 2001 | 99.24104663 | 0.722099055 | 15.06516239 |
| 1596 | Hungary | 2002 | 100.7475545 | 0.728384596 | 14.22935117 |
| 1597 | Hungary | 2005 | 106.7468496 | 0.745356806 | 13.44730416 |
| 1598 | Hungary | 2006 | 109.2424895 | 0.750515381 | 13.44730416 |
| 1599 | Hungary | 2007 | 112.2644352 | 0.754942942 | 10.45018691 |
| 1600 | Hungary | 2008 | 115.5573787 | 0.759317468 | 9.92126395 |
| 1601 | Hungary | 2009 | 118.8473347 | 0.763563687 | 9.92126395 |
| 1602 | Hungary | 2010 | 121.8697576 | 0.767328649 | 9.92126395 |
| 1603 | Hungary | 2014 | 132.8742177 | 0.772575046 | 9.92126395 |
| 1604 | Hungary | 2015 | 135.4457171 | 0.774047383 | 9.92126395 |
| 1605 | Hungary | 2017 | 141.4674539 | 0.779116417 | 9.92126395 |
| 1606 | Hungary | 2018 | 144.1564045 | 0.782083661 | 9.92126395 |
| 1607 | Hungary | 2019 | 145.6751348 | 0.784910309 | 8.445315313 |
| 1608 | Hungary | 2020 | 145.3179932 | 0.78762039 | 8.445315313 |
| 1609 | Hungary | 2021 | 142.3677063 | 0.790754768 | 8.445315313 |
| 1610 | Iceland | 1991 | 13.64964078 | 0.769027393 | 9.92126395 |
| 1611 | Iceland | 1993 | 15.76466264 | 0.776389423 | 9.92126395 |
| 1612 | Iceland | 1994 | 16.39463257 | 0.779967961 | 9.92126395 |
| 1613 | Iceland | 1998 | 15.84905163 | 0.792626368 | 8.445315313 |
| 1614 | Iceland | 2001 | 15.95634088 | 0.808965786 | 8.445315313 |
| 1615 | Iceland | 2004 | 19.35559099 | 0.819528087 | 8.445315313 |
| 1616 | Iceland | 2006 | 21.37634171 | 0.826244743 | 8.445315313 |
| 1617 | Iceland | 2007 | 22.43276629 | 0.83028989 | 8.445315313 |
| 1618 | Iceland | 2009 | 24.14131988 | 0.838121013 | 8.445315313 |
| 1619 | Iceland | 2010 | 24.46931247 | 0.841384642 | 8.445315313 |
| 1620 | Iceland | 2011 | 24.38377729 | 0.84461993 | 8.445315313 |
| 1621 | Iceland | 2013 | 23.69395765 | 0.850173948 | 8.445315313 |
| 1622 | Iceland | 2014 | 23.37926725 | 0.853558697 | 8.445315313 |
| 1623 | Iceland | 2016 | 24.63249051 | 0.861844467 | 8.445315313 |
| 1624 | Iceland | 2017 | 27.54338059 | 0.865702891 | 8.445315313 |
| 1625 | Iceland | 2018 | 30.44221344 | 0.869219822 | 8.445315313 |
| 1626 | Iceland | 2019 | 31.75963149 | 0.87242558 | 8.445315313 |
| 1627 | Iceland | 2020 | 25.74687748 | 0.874323153 | 8.445315313 |
| 1628 | Iceland | 2021 | 24.23966962 | 0.87636168 | 8.445315313 |
| 1629 | India | 1992 | 97.51131078 | 0.344591727 | 65.82455226 |
| 1630 | India | 1993 | 97.42114666 | 0.350685397 | 65.82455226 |
| 1631 | India | 1995 | 97.37939383 | 0.363418501 | 64.06460795 |
| 1632 | India | 1996 | 97.50369459 | 0.370018644 | 64.06460795 |
| 1633 | India | 1997 | 97.94128525 | 0.376358763 | 63.35324861 |
| 1634 | India | 2000 | 99.5988514 | 0.395752331 | 62.92622144 |
| 1635 | India | 2001 | 100.0517814 | 0.401837472 | 62.92622144 |
| 1636 | India | 2002 | 100.5358115 | 0.407411414 | 62.92622144 |
| 1637 | India | 2004 | 101.3766512 | 0.419184961 | 62.92622144 |
| 1638 | India | 2007 | 101.8144529 | 0.44135146 | 62.92622144 |
| 1639 | India | 2008 | 101.8862926 | 0.449235851 | 62.92622144 |
| 1640 | India | 2009 | 101.9739214 | 0.457672332 | 62.92622144 |
| 1641 | India | 2010 | 102.1007156 | 0.467018996 | 53.58976548 |
| 1642 | India | 2012 | 102.1487086 | 0.487210876 | 53.58976548 |
| 1643 | India | 2014 | 102.1416852 | 0.5097221 | 53.58976548 |
| 1644 | India | 2015 | 102.3615747 | 0.521242719 | 53.58976548 |
| 1645 | India | 2017 | 103.5622486 | 0.542388507 | 53.31984512 |
| 1646 | India | 2021 | 106.1621256 | 0.575401649 | 51.75814966 |
| 1647 | Indonesia | 1991 | 190.0442986 | 0.466505583 | 62.92622144 |
| 1648 | Indonesia | 1993 | 190.8034469 | 0.484775241 | 53.58976548 |
| 1649 | Indonesia | 1994 | 190.9866318 | 0.4936071 | 53.58976548 |
| 1650 | Indonesia | 1995 | 191.0258493 | 0.502008381 | 53.58976548 |
| 1651 | Indonesia | 1996 | 190.8992014 | 0.510108146 | 53.58976548 |
| 1652 | Indonesia | 1997 | 190.5947996 | 0.518102189 | 53.58976548 |
| 1653 | Indonesia | 1998 | 190.1977673 | 0.524045604 | 53.58976548 |
| 1654 | Indonesia | 2001 | 189.0255216 | 0.537977981 | 53.31984512 |
| 1655 | Indonesia | 2002 | 188.3934721 | 0.542245676 | 53.31984512 |
| 1656 | Indonesia | 2004 | 187.0261923 | 0.550902314 | 51.75814966 |
| 1657 | Indonesia | 2005 | 186.5399549 | 0.555424913 | 51.75814966 |
| 1658 | Indonesia | 2008 | 185.4330516 | 0.572225267 | 51.75814966 |
| 1659 | Indonesia | 2009 | 185.1288264 | 0.578866902 | 51.75814966 |
| 1660 | Indonesia | 2010 | 184.906948 | 0.585982419 | 51.75814966 |
| 1661 | Indonesia | 2011 | 184.7279914 | 0.593652985 | 51.75814966 |
| 1662 | Indonesia | 2012 | 184.5643544 | 0.601185701 | 51.75814966 |
| 1663 | Indonesia | 2013 | 184.4541386 | 0.608509904 | 51.75814966 |
| 1664 | Indonesia | 2014 | 184.4344556 | 0.615499887 | 32.04043631 |
| 1665 | Indonesia | 2015 | 184.5391229 | 0.622222469 | 32.04043631 |
| 1666 | Indonesia | 2021 | 190.3931051 | 0.656868336 | 22.46369183 |
| 1667 | Iran (Islamic Republic of) | 1990 | 226.1284587 | 0.453799944 | 62.92622144 |
| 1668 | Iran (Islamic Republic of) | 1991 | 224.567462 | 0.468982858 | 53.58976548 |
| 1669 | Iran (Islamic Republic of) | 1992 | 223.2208192 | 0.480626697 | 53.58976548 |
| 1670 | Iran (Islamic Republic of) | 1993 | 222.1514526 | 0.492277025 | 53.58976548 |
| 1671 | Iran (Islamic Republic of) | 1994 | 221.3951045 | 0.505072857 | 53.58976548 |
| 1672 | Iran (Islamic Republic of) | 1995 | 220.9880387 | 0.517531494 | 53.58976548 |
| 1673 | Iran (Islamic Republic of) | 1996 | 220.8679451 | 0.52860072 | 53.58976548 |
| 1674 | Iran (Islamic Republic of) | 1997 | 220.9418041 | 0.537727779 | 53.31984512 |
| 1675 | Iran (Islamic Republic of) | 1999 | 221.6230587 | 0.555624332 | 51.75814966 |
| 1676 | Iran (Islamic Republic of) | 2000 | 222.1606652 | 0.565474561 | 51.75814966 |
| 1677 | Iran (Islamic Republic of) | 2002 | 224.3693581 | 0.584097268 | 51.75814966 |
| 1678 | Iran (Islamic Republic of) | 2004 | 227.8301569 | 0.60371745 | 51.75814966 |
| 1679 | Iran (Islamic Republic of) | 2005 | 229.6047189 | 0.613291056 | 51.75814966 |
| 1680 | Iran (Islamic Republic of) | 2007 | 234.3783958 | 0.627749442 | 22.46369183 |
| 1681 | Iran (Islamic Republic of) | 2008 | 237.1832713 | 0.63286223 | 22.46369183 |
| 1682 | Iran (Islamic Republic of) | 2009 | 239.7489138 | 0.63739838 | 22.46369183 |
| 1683 | Iran (Islamic Republic of) | 2010 | 241.6714883 | 0.64255917 | 22.46369183 |
| 1684 | Iran (Islamic Republic of) | 2011 | 243.1849839 | 0.647899089 | 22.46369183 |
| 1685 | Iran (Islamic Republic of) | 2012 | 244.6951141 | 0.651500587 | 22.46369183 |
| 1686 | Iran (Islamic Republic of) | 2015 | 248.0433938 | 0.662057987 | 22.46369183 |
| 1687 | Iran (Islamic Republic of) | 2016 | 248.6500866 | 0.667652445 | 22.46369183 |
| 1688 | Iran (Islamic Republic of) | 2017 | 249.2854168 | 0.674343462 | 22.03195463 |
| 1689 | Iran (Islamic Republic of) | 2018 | 249.7897086 | 0.680909621 | 17.2540694 |
| 1690 | Iran (Islamic Republic of) | 2020 | 249.3703909 | 0.691918763 | 17.2540694 |
| 1691 | Iraq | 1990 | 206.6134913 | 0.412044173 | 62.92622144 |
| 1692 | Iraq | 1991 | 205.3057587 | 0.415814116 | 62.92622144 |
| 1693 | Iraq | 1995 | 202.2479427 | 0.432075545 | 62.92622144 |
| 1694 | Iraq | 1996 | 202.2433324 | 0.436818479 | 62.92622144 |
| 1695 | Iraq | 1999 | 202.9337363 | 0.462736441 | 62.92622144 |
| 1696 | Iraq | 2000 | 202.8953609 | 0.472376399 | 53.58976548 |
| 1697 | Iraq | 2001 | 202.3891286 | 0.48145832 | 53.58976548 |
| 1698 | Iraq | 2003 | 200.3842362 | 0.492174445 | 53.58976548 |
| 1699 | Iraq | 2004 | 199.3928164 | 0.498933881 | 53.58976548 |
| 1700 | Iraq | 2006 | 198.3616698 | 0.510918049 | 53.58976548 |
| 1701 | Iraq | 2008 | 197.6362818 | 0.525242908 | 53.58976548 |
| 1702 | Iraq | 2009 | 197.2743556 | 0.533200914 | 53.31984512 |
| 1703 | Iraq | 2012 | 195.066511 | 0.564745467 | 51.75814966 |
| 1704 | Iraq | 2013 | 193.9008729 | 0.576968998 | 51.75814966 |
| 1705 | Iraq | 2015 | 192.5418816 | 0.599494007 | 51.75814966 |
| 1706 | Iraq | 2017 | 193.1621662 | 0.622674532 | 32.04043631 |
| 1707 | Iraq | 2018 | 193.6924253 | 0.633232108 | 22.46369183 |
| 1708 | Iraq | 2019 | 194.0492459 | 0.643635318 | 22.46369183 |
| 1709 | Iraq | 2021 | 194.8663555 | 0.662626231 | 22.46369183 |
| 1710 | Ireland | 1991 | 14.77524801 | 0.725195019 | 14.77524801 |
| 1711 | Ireland | 1992 | 14.92870046 | 0.731918691 | 14.22935117 |
| 1712 | Ireland | 1995 | 15.58365865 | 0.748921575 | 13.44730416 |
| 1713 | Ireland | 1997 | 16.24952469 | 0.759970384 | 9.92126395 |
| 1714 | Ireland | 1998 | 16.70596735 | 0.766488721 | 9.92126395 |
| 1715 | Ireland | 1999 | 17.23504503 | 0.773538834 | 9.92126395 |
| 1716 | Ireland | 2001 | 18.66130866 | 0.786353741 | 8.445315313 |
| 1717 | Ireland | 2002 | 19.81283568 | 0.793010606 | 8.445315313 |
| 1718 | Ireland | 2003 | 21.12984675 | 0.800005865 | 8.445315313 |
| 1719 | Ireland | 2005 | 23.64159789 | 0.811713818 | 8.445315313 |
| 1720 | Ireland | 2006 | 24.71755189 | 0.814505479 | 8.445315313 |
| 1721 | Ireland | 2007 | 25.79586678 | 0.816931026 | 8.445315313 |
| 1722 | Ireland | 2008 | 26.83721047 | 0.819932128 | 8.445315313 |
| 1723 | Ireland | 2009 | 27.79948199 | 0.823929666 | 8.445315313 |
| 1724 | Ireland | 2010 | 28.64312376 | 0.828103848 | 8.445315313 |
| 1725 | Ireland | 2011 | 29.46005792 | 0.831804792 | 8.445315313 |
| 1726 | Ireland | 2012 | 30.30057776 | 0.835325359 | 8.445315313 |
| 1727 | Ireland | 2013 | 31.05866858 | 0.838644433 | 8.445315313 |
| 1728 | Ireland | 2015 | 31.8900913 | 0.847437887 | 8.445315313 |
| 1729 | Ireland | 2017 | 31.84694345 | 0.857816164 | 8.445315313 |
| 1730 | Ireland | 2018 | 31.74960754 | 0.863925284 | 8.445315313 |
| 1731 | Israel | 1990 | 24.5512353 | 0.709178347 | 17.2540694 |
| 1732 | Israel | 1991 | 24.22812793 | 0.713371633 | 17.2540694 |
| 1733 | Israel | 1993 | 23.79007638 | 0.722250176 | 15.06516239 |
| 1734 | Israel | 1994 | 23.68402666 | 0.726654736 | 14.77524801 |
| 1735 | Israel | 1995 | 23.66143894 | 0.731127859 | 14.22935117 |
| 1736 | Israel | 1996 | 23.74531447 | 0.734714026 | 14.22935117 |
| 1737 | Israel | 1998 | 24.19808847 | 0.74192719 | 13.44730416 |
| 1738 | Israel | 2000 | 24.76857316 | 0.749184084 | 13.44730416 |
| 1739 | Israel | 2001 | 25.0635754 | 0.752834297 | 13.44730416 |
| 1740 | Israel | 2002 | 25.41354736 | 0.755988076 | 10.45018691 |
| 1741 | Israel | 2003 | 25.81415405 | 0.758875409 | 9.92126395 |
| 1742 | Israel | 2004 | 26.25304695 | 0.762213291 | 9.92126395 |
| 1743 | Israel | 2006 | 27.61324974 | 0.768733898 | 9.92126395 |
| 1744 | Israel | 2008 | 30.77156619 | 0.772011615 | 9.92126395 |
| 1745 | Israel | 2009 | 32.33874233 | 0.773303913 | 9.92126395 |
| 1746 | Israel | 2010 | 33.43340399 | 0.77525953 | 9.92126395 |
| 1747 | Israel | 2012 | 34.70891342 | 0.780403415 | 9.92126395 |
| 1748 | Israel | 2013 | 35.25804299 | 0.783267167 | 8.445315313 |
| 1749 | Israel | 2014 | 35.80575525 | 0.786151084 | 8.445315313 |
| 1750 | Israel | 2016 | 37.28845756 | 0.791772445 | 8.445315313 |
| 1751 | Israel | 2018 | 39.53079311 | 0.799124994 | 8.445315313 |
| 1752 | Israel | 2019 | 40.132087 | 0.803232991 | 8.445315313 |
| 1753 | Israel | 2020 | 40.1530803 | 0.806351589 | 8.445315313 |
| 1754 | Israel | 2021 | 35.86632356 | 0.809011652 | 8.445315313 |
| 1755 | Italy | 1990 | 100.8968437 | 0.706255224 | 17.2540694 |
| 1756 | Italy | 1991 | 98.62384225 | 0.711457349 | 17.2540694 |
| 1757 | Italy | 1992 | 97.59485205 | 0.716649746 | 17.2540694 |
| 1758 | Italy | 1993 | 97.53757981 | 0.721720992 | 15.06516239 |
| 1759 | Italy | 1995 | 99.13699661 | 0.731644082 | 14.22935117 |
| 1760 | Italy | 1996 | 106.0317166 | 0.73605087 | 13.44730416 |
| 1761 | Italy | 1997 | 121.3105231 | 0.739972336 | 13.44730416 |
| 1762 | Italy | 1998 | 140.0719456 | 0.743101596 | 13.44730416 |
| 1763 | Italy | 1999 | 157.3951038 | 0.746074084 | 13.44730416 |
| 1764 | Italy | 2001 | 174.1301125 | 0.753554839 | 10.45018691 |
| 1765 | Italy | 2002 | 179.3516804 | 0.757191479 | 10.45018691 |
| 1766 | Italy | 2003 | 183.8568216 | 0.760093744 | 9.92126395 |
| 1767 | Italy | 2004 | 187.4893369 | 0.762787749 | 9.92126395 |
| 1768 | Italy | 2007 | 193.9755233 | 0.77069838 | 9.92126395 |
| 1769 | Italy | 2010 | 196.6831004 | 0.778109206 | 9.92126395 |
| 1770 | Italy | 2011 | 195.1854132 | 0.780703468 | 9.92126395 |
| 1771 | Italy | 2013 | 187.807956 | 0.785356877 | 8.445315313 |
| 1772 | Italy | 2014 | 184.3840807 | 0.787533956 | 8.445315313 |
| 1773 | Italy | 2015 | 182.7519086 | 0.789902119 | 8.445315313 |
| 1774 | Italy | 2016 | 182.7917449 | 0.792477244 | 8.445315313 |
| 1775 | Italy | 2018 | 183.6645076 | 0.79836269 | 8.445315313 |
| 1776 | Italy | 2021 | 169.8940849 | 0.805773534 | 8.445315313 |
| 1777 | Jamaica | 1990 | 330.5055222 | 0.534781234 | 53.31984512 |
| 1778 | Jamaica | 1992 | 321.5281571 | 0.545088753 | 51.75814966 |
| 1779 | Jamaica | 1993 | 317.7521362 | 0.551218492 | 51.75814966 |
| 1780 | Jamaica | 1994 | 314.765056 | 0.557643773 | 51.75814966 |
| 1781 | Jamaica | 1995 | 312.7056823 | 0.564315038 | 51.75814966 |
| 1782 | Jamaica | 1998 | 308.9613579 | 0.583048035 | 51.75814966 |
| 1783 | Jamaica | 2000 | 307.6057121 | 0.594592917 | 51.75814966 |
| 1784 | Jamaica | 2004 | 305.1871895 | 0.615836278 | 32.04043631 |
| 1785 | Jamaica | 2005 | 305.4063328 | 0.620400057 | 32.04043631 |
| 1786 | Jamaica | 2007 | 312.8803119 | 0.62954947 | 22.46369183 |
| 1787 | Jamaica | 2008 | 318.7993522 | 0.633967851 | 22.46369183 |
| 1788 | Jamaica | 2010 | 325.8655914 | 0.642129388 | 22.46369183 |
| 1789 | Jamaica | 2011 | 326.0209455 | 0.64639583 | 22.46369183 |
| 1790 | Jamaica | 2012 | 325.6488115 | 0.65058376 | 22.46369183 |
| 1791 | Jamaica | 2016 | 325.5883386 | 0.666469279 | 22.46369183 |
| 1792 | Jamaica | 2017 | 327.1215391 | 0.670291045 | 22.46369183 |
| 1793 | Jamaica | 2018 | 328.8159038 | 0.674075313 | 22.03195463 |
| 1794 | Jamaica | 2019 | 330.0068719 | 0.677778539 | 22.03195463 |
| 1795 | Jamaica | 2021 | 317.0428921 | 0.683263064 | 17.2540694 |
| 1796 | Japan | 1991 | 48.75986135 | 0.794616189 | 8.445315313 |
| 1797 | Japan | 1998 | 45.47244814 | 0.819016202 | 8.445315313 |
| 1798 | Japan | 2000 | 43.42143951 | 0.822938999 | 8.445315313 |
| 1799 | Japan | 2001 | 42.44438418 | 0.824723241 | 8.445315313 |
| 1800 | Japan | 2003 | 40.13709662 | 0.829392223 | 8.445315313 |
| 1801 | Japan | 2004 | 39.18237364 | 0.832037381 | 8.445315313 |
| 1802 | Japan | 2005 | 38.60992416 | 0.834288685 | 8.445315313 |
| 1803 | Japan | 2006 | 38.30528012 | 0.836222264 | 8.445315313 |
| 1804 | Japan | 2007 | 38.03868098 | 0.83829416 | 8.445315313 |
| 1805 | Japan | 2010 | 37.70850409 | 0.844361811 | 8.445315313 |
| 1806 | Japan | 2013 | 39.16741165 | 0.851356994 | 8.445315313 |
| 1807 | Japan | 2015 | 40.2877808 | 0.856097499 | 8.445315313 |
| 1808 | Japan | 2016 | 40.65079242 | 0.858698173 | 8.445315313 |
| 1809 | Japan | 2017 | 41.02635079 | 0.861393309 | 8.445315313 |
| 1810 | Japan | 2018 | 41.35404818 | 0.864237137 | 8.445315313 |
| 1811 | Japan | 2020 | 41.28629249 | 0.869106879 | 8.445315313 |
| 1812 | Jordan | 1990 | 305.7408856 | 0.539147468 | 53.31984512 |
| 1813 | Jordan | 1991 | 304.8695122 | 0.542819411 | 53.31984512 |
| 1814 | Jordan | 1992 | 304.1664844 | 0.547458846 | 51.75814966 |
| 1815 | Jordan | 1993 | 303.6435328 | 0.55218235 | 51.75814966 |
| 1816 | Jordan | 1994 | 303.3151813 | 0.557254335 | 51.75814966 |
| 1817 | Jordan | 1995 | 303.2017942 | 0.562571536 | 51.75814966 |
| 1818 | Jordan | 1997 | 303.6423393 | 0.572581508 | 51.75814966 |
| 1819 | Jordan | 1998 | 304.1725125 | 0.57764357 | 51.75814966 |
| 1820 | Jordan | 2001 | 307.5499466 | 0.593182803 | 51.75814966 |
| 1821 | Jordan | 2002 | 310.5336752 | 0.599000385 | 51.75814966 |
| 1822 | Jordan | 2003 | 314.1801679 | 0.60494595 | 51.75814966 |
| 1823 | Jordan | 2004 | 318.0295437 | 0.611934404 | 51.75814966 |
| 1824 | Jordan | 2005 | 321.7258391 | 0.619909228 | 32.04043631 |
| 1825 | Jordan | 2006 | 325.4580619 | 0.627803813 | 22.46369183 |
| 1826 | Jordan | 2008 | 333.3767567 | 0.643892693 | 22.46369183 |
| 1827 | Jordan | 2009 | 337.0785144 | 0.652196319 | 22.46369183 |
| 1828 | Jordan | 2011 | 343.6979167 | 0.66739668 | 22.46369183 |
| 1829 | Jordan | 2014 | 354.2213859 | 0.68713404 | 17.2540694 |
| 1830 | Jordan | 2017 | 356.202488 | 0.703923826 | 17.2540694 |
| 1831 | Jordan | 2019 | 356.054768 | 0.714998946 | 17.2540694 |
| 1832 | Jordan | 2020 | 355.6332154 | 0.72006824 | 15.06516239 |
| 1833 | Jordan | 2021 | 341.8945792 | 0.725307227 | 14.77524801 |
| 1834 | Kazakhstan | 1990 | 66.2499713 | 0.589435804 | 51.75814966 |
| 1835 | Kazakhstan | 1991 | 66.44370807 | 0.590967832 | 51.75814966 |
| 1836 | Kazakhstan | 1993 | 67.43563414 | 0.603138328 | 51.75814966 |
| 1837 | Kazakhstan | 1996 | 71.15973893 | 0.626013138 | 32.04043631 |
| 1838 | Kazakhstan | 1997 | 75.25064333 | 0.631166626 | 22.46369183 |
| 1839 | Kazakhstan | 1999 | 84.29219262 | 0.640180485 | 22.46369183 |
| 1840 | Kazakhstan | 2002 | 88.132731 | 0.651529335 | 22.46369183 |
| 1841 | Kazakhstan | 2004 | 87.76567432 | 0.658733071 | 22.46369183 |
| 1842 | Kazakhstan | 2006 | 86.33252199 | 0.667048858 | 22.46369183 |
| 1843 | Kazakhstan | 2007 | 85.18844641 | 0.671367208 | 22.03195463 |
| 1844 | Kazakhstan | 2009 | 82.47888721 | 0.681606839 | 17.2540694 |
| 1845 | Kazakhstan | 2010 | 81.13993452 | 0.686996643 | 17.2540694 |
| 1846 | Kazakhstan | 2013 | 73.97786482 | 0.699053593 | 17.2540694 |
| 1847 | Kazakhstan | 2014 | 70.95697353 | 0.703511921 | 17.2540694 |
| 1848 | Kazakhstan | 2016 | 65.28256733 | 0.71256904 | 17.2540694 |
| 1849 | Kazakhstan | 2020 | 56.44488609 | 0.722788768 | 15.06516239 |
| 1850 | Kazakhstan | 2021 | 58.67533131 | 0.725144495 | 15.06516239 |
| 1851 | Kenya | 1990 | 290.2882308 | 0.333850293 | 65.82455226 |
| 1852 | Kenya | 1992 | 312.0654708 | 0.346298636 | 65.82455226 |
| 1853 | Kenya | 1994 | 327.018411 | 0.356779194 | 64.06460795 |
| 1854 | Kenya | 1995 | 330.4445339 | 0.361535807 | 64.06460795 |
| 1855 | Kenya | 1997 | 333.2054294 | 0.370436465 | 64.06460795 |
| 1856 | Kenya | 1999 | 334.4644355 | 0.378630009 | 63.35324861 |
| 1857 | Kenya | 2000 | 334.5138274 | 0.382395616 | 63.35324861 |
| 1858 | Kenya | 2002 | 334.2516096 | 0.389886256 | 62.92622144 |
| 1859 | Kenya | 2004 | 333.4823321 | 0.397743651 | 62.92622144 |
| 1860 | Kenya | 2006 | 327.3078364 | 0.407745764 | 62.92622144 |
| 1861 | Kenya | 2007 | 316.4899474 | 0.413736227 | 62.92622144 |
| 1862 | Kenya | 2013 | 292.5009079 | 0.456666942 | 62.92622144 |
| 1863 | Kenya | 2014 | 293.895942 | 0.464724302 | 62.92622144 |
| 1864 | Kenya | 2016 | 295.2455115 | 0.481709072 | 53.58976548 |
| 1865 | Kenya | 2017 | 295.5341153 | 0.490296619 | 53.58976548 |
| 1866 | Kenya | 2018 | 295.8394672 | 0.499101274 | 53.58976548 |
| 1867 | Kenya | 2019 | 296.2714463 | 0.508004211 | 53.58976548 |
| 1868 | Kenya | 2020 | 296.8355388 | 0.516136572 | 53.58976548 |
| 1869 | Kenya | 2021 | 298.9655592 | 0.523768077 | 53.58976548 |
| 1870 | Kiribati | 1990 | 77.27854357 | 0.410389821 | 62.92622144 |
| 1871 | Kiribati | 1992 | 77.09632136 | 0.415577609 | 62.92622144 |
| 1872 | Kiribati | 1993 | 77.00212925 | 0.418481661 | 62.92622144 |
| 1873 | Kiribati | 1994 | 76.91174367 | 0.421716217 | 62.92622144 |
| 1874 | Kiribati | 1995 | 76.82690917 | 0.425024354 | 62.92622144 |
| 1875 | Kiribati | 2002 | 75.60122879 | 0.453462298 | 62.92622144 |
| 1876 | Kiribati | 2003 | 75.30789853 | 0.458208374 | 62.92622144 |
| 1877 | Kiribati | 2005 | 74.82352611 | 0.466722276 | 53.58976548 |
| 1878 | Kiribati | 2008 | 74.3243186 | 0.477201933 | 53.58976548 |
| 1879 | Kiribati | 2013 | 73.57130182 | 0.491880767 | 53.58976548 |
| 1880 | Kiribati | 2014 | 73.52645277 | 0.495317241 | 53.58976548 |
| 1881 | Kiribati | 2015 | 73.60080728 | 0.500059699 | 53.58976548 |
| 1882 | Kiribati | 2016 | 73.89606507 | 0.505207837 | 53.58976548 |
| 1883 | Kiribati | 2018 | 74.85796929 | 0.514810384 | 53.58976548 |
| 1884 | Kiribati | 2019 | 75.16623518 | 0.519441321 | 53.58976548 |
| 1885 | Kiribati | 2020 | 75.06773909 | 0.523454113 | 53.58976548 |
| 1886 | Kiribati | 2021 | 74.63203819 | 0.527186583 | 53.58976548 |
| 1887 | Kuwait | 1992 | 308.0536882 | 0.67015911 | 22.46369183 |
| 1888 | Kuwait | 1993 | 308.5044264 | 0.670036252 | 22.46369183 |
| 1889 | Kuwait | 1994 | 308.5737906 | 0.671393213 | 22.03195463 |
| 1890 | Kuwait | 1995 | 308.3770098 | 0.677215523 | 22.03195463 |
| 1891 | Kuwait | 1997 | 307.4278041 | 0.695789389 | 17.2540694 |
| 1892 | Kuwait | 1998 | 306.7626232 | 0.704383736 | 17.2540694 |
| 1893 | Kuwait | 1999 | 305.8974901 | 0.711841328 | 17.2540694 |
| 1894 | Kuwait | 2000 | 305.0603515 | 0.718357562 | 17.2540694 |
| 1895 | Kuwait | 2001 | 303.9362741 | 0.722840631 | 15.06516239 |
| 1896 | Kuwait | 2002 | 302.2978776 | 0.726816976 | 14.77524801 |
| 1897 | Kuwait | 2003 | 300.4780586 | 0.731778685 | 14.22935117 |
| 1898 | Kuwait | 2004 | 298.4536846 | 0.738153707 | 13.44730416 |
| 1899 | Kuwait | 2006 | 294.7302538 | 0.754928493 | 10.45018691 |
| 1900 | Kuwait | 2007 | 292.5862202 | 0.762411484 | 9.92126395 |
| 1901 | Kuwait | 2009 | 288.799583 | 0.777546551 | 9.92126395 |
| 1902 | Kuwait | 2011 | 287.5990944 | 0.791812065 | 8.445315313 |
| 1903 | Kuwait | 2012 | 288.0086966 | 0.798356988 | 8.445315313 |
| 1904 | Kuwait | 2013 | 288.5814923 | 0.804583834 | 8.445315313 |
| 1905 | Kuwait | 2015 | 290.2115822 | 0.816998413 | 8.445315313 |
| 1906 | Kuwait | 2016 | 290.5312821 | 0.823026527 | 8.445315313 |
| 1907 | Kuwait | 2017 | 290.473206 | 0.828886987 | 8.445315313 |
| 1908 | Kuwait | 2018 | 290.3468705 | 0.834371461 | 8.445315313 |
| 1909 | Kuwait | 2019 | 290.2572165 | 0.839688307 | 8.445315313 |
| 1910 | Kuwait | 2020 | 289.8514973 | 0.843459812 | 8.445315313 |
| 1911 | Kuwait | 2021 | 286.1613795 | 0.846651055 | 8.445315313 |
| 1912 | Kyrgyzstan | 1990 | 62.20237494 | 0.519407652 | 53.58976548 |
| 1913 | Kyrgyzstan | 1992 | 60.93632438 | 0.527901552 | 53.58976548 |
| 1914 | Kyrgyzstan | 1994 | 60.20474569 | 0.53623298 | 53.31984512 |
| 1915 | Kyrgyzstan | 1995 | 60.10785274 | 0.53905282 | 53.31984512 |
| 1916 | Kyrgyzstan | 1996 | 60.25418488 | 0.541628927 | 53.31984512 |
| 1917 | Kyrgyzstan | 1997 | 60.59450451 | 0.54334115 | 53.31984512 |
| 1918 | Kyrgyzstan | 1998 | 60.99336206 | 0.543258546 | 53.31984512 |
| 1919 | Kyrgyzstan | 1999 | 61.31523639 | 0.542867282 | 53.31984512 |
| 1920 | Kyrgyzstan | 2004 | 55.98311269 | 0.547262343 | 51.75814966 |
| 1921 | Kyrgyzstan | 2005 | 55.36608745 | 0.548260449 | 51.75814966 |
| 1922 | Kyrgyzstan | 2006 | 55.63237135 | 0.549383594 | 51.75814966 |
| 1923 | Kyrgyzstan | 2007 | 56.30236126 | 0.551146192 | 51.75814966 |
| 1924 | Kyrgyzstan | 2008 | 57.2513764 | 0.553206382 | 51.75814966 |
| 1925 | Kyrgyzstan | 2010 | 59.43349809 | 0.556416545 | 51.75814966 |
| 1926 | Kyrgyzstan | 2011 | 60.84030659 | 0.558923335 | 51.75814966 |
| 1927 | Kyrgyzstan | 2012 | 62.81567463 | 0.561541574 | 51.75814966 |
| 1928 | Kyrgyzstan | 2013 | 65.16984453 | 0.565776769 | 51.75814966 |
| 1929 | Kyrgyzstan | 2014 | 67.69345681 | 0.570569357 | 51.75814966 |
| 1930 | Kyrgyzstan | 2016 | 73.14835988 | 0.580388551 | 51.75814966 |
| 1931 | Kyrgyzstan | 2017 | 76.59239687 | 0.585766096 | 51.75814966 |
| 1932 | Kyrgyzstan | 2018 | 79.62903167 | 0.591237134 | 51.75814966 |
| 1933 | Kyrgyzstan | 2019 | 81.35508325 | 0.596501638 | 51.75814966 |
| 1934 | Kyrgyzstan | 2020 | 80.64175793 | 0.600499554 | 51.75814966 |
| 1935 | Kyrgyzstan | 2021 | 92.77039646 | 0.603979328 | 51.75814966 |
| 1936 | Lao People's Democratic Republic | 1991 | 164.6681973 | 0.268787618 | 100.8275782 |
| 1937 | Lao People's Democratic Republic | 1992 | 164.9434295 | 0.273435312 | 100.8275782 |
| 1938 | Lao People's Democratic Republic | 1993 | 165.1426763 | 0.278140418 | 100.8275782 |
| 1939 | Lao People's Democratic Republic | 1995 | 165.296321 | 0.288795341 | 100.8275782 |
| 1940 | Lao People's Democratic Republic | 1998 | 164.3113035 | 0.308402545 | 89.96318282 |
| 1941 | Lao People's Democratic Republic | 1999 | 163.8220864 | 0.315972615 | 67.17014806 |
| 1942 | Lao People's Democratic Republic | 2000 | 163.4323242 | 0.323947359 | 66.46502371 |
| 1943 | Lao People's Democratic Republic | 2001 | 163.1797249 | 0.332580409 | 65.82455226 |
| 1944 | Lao People's Democratic Republic | 2002 | 162.953797 | 0.341667271 | 65.82455226 |
| 1945 | Lao People's Democratic Republic | 2003 | 162.778131 | 0.350658178 | 65.82455226 |
| 1946 | Lao People's Democratic Republic | 2004 | 162.6756632 | 0.359681759 | 64.06460795 |
| 1947 | Lao People's Democratic Republic | 2007 | 163.1658459 | 0.387723649 | 62.92622144 |
| 1948 | Lao People's Democratic Republic | 2008 | 163.5476487 | 0.397011299 | 62.92622144 |
| 1949 | Lao People's Democratic Republic | 2009 | 163.9177731 | 0.406139022 | 62.92622144 |
| 1950 | Lao People's Democratic Republic | 2010 | 164.1981082 | 0.414865566 | 62.92622144 |
| 1951 | Lao People's Democratic Republic | 2011 | 164.4703473 | 0.423354523 | 62.92622144 |
| 1952 | Lao People's Democratic Republic | 2012 | 164.7584939 | 0.431149361 | 62.92622144 |
| 1953 | Lao People's Democratic Republic | 2013 | 165.0380785 | 0.438691039 | 62.92622144 |
| 1954 | Lao People's Democratic Republic | 2014 | 165.281297 | 0.445869424 | 62.92622144 |
| 1955 | Lao People's Democratic Republic | 2015 | 165.4556941 | 0.452749603 | 62.92622144 |
| 1956 | Lao People's Democratic Republic | 2016 | 165.7185766 | 0.459583996 | 62.92622144 |
| 1957 | Lao People's Democratic Republic | 2017 | 166.0770237 | 0.466168892 | 62.92622144 |
| 1958 | Lao People's Democratic Republic | 2021 | 170.144304 | 0.489136091 | 53.58976548 |
| 1959 | Latvia | 1990 | 17.2540694 | 0.680193638 | 17.2540694 |
| 1960 | Latvia | 1992 | 17.70140927 | 0.696157036 | 17.2540694 |
| 1961 | Latvia | 1993 | 18.1425017 | 0.705249855 | 17.2540694 |
| 1962 | Latvia | 1995 | 19.33321288 | 0.717639459 | 17.2540694 |
| 1963 | Latvia | 1997 | 22.51620914 | 0.72374658 | 15.06516239 |
| 1964 | Latvia | 1999 | 27.80516899 | 0.727219685 | 14.77524801 |
| 1965 | Latvia | 2001 | 33.15930217 | 0.736294196 | 13.44730416 |
| 1966 | Latvia | 2002 | 36.0375727 | 0.74187044 | 13.44730416 |
| 1967 | Latvia | 2003 | 39.14804555 | 0.748168162 | 13.44730416 |
| 1968 | Latvia | 2004 | 42.50024321 | 0.755263211 | 10.45018691 |
| 1969 | Latvia | 2005 | 46.1229076 | 0.761929433 | 9.92126395 |
| 1970 | Latvia | 2006 | 50.33151755 | 0.768459929 | 9.92126395 |
| 1971 | Latvia | 2008 | 60.42167427 | 0.786324573 | 8.445315313 |
| 1972 | Latvia | 2009 | 65.6293327 | 0.794504427 | 8.445315313 |
| 1973 | Latvia | 2011 | 75.58598376 | 0.799080776 | 8.445315313 |
| 1974 | Latvia | 2013 | 86.280498 | 0.80266049 | 8.445315313 |
| 1975 | Latvia | 2014 | 90.31735072 | 0.803721486 | 8.445315313 |
| 1976 | Latvia | 2018 | 93.81993794 | 0.821419809 | 8.445315313 |
| 1977 | Latvia | 2019 | 93.82791843 | 0.824781777 | 8.445315313 |
| 1978 | Latvia | 2020 | 91.88352979 | 0.827900448 | 8.445315313 |
| 1979 | Lebanon | 1990 | 193.6675727 | 0.536718969 | 53.31984512 |
| 1980 | Lebanon | 1991 | 193.5487654 | 0.539197492 | 53.31984512 |
| 1981 | Lebanon | 1995 | 194.0003861 | 0.55147172 | 51.75814966 |
| 1982 | Lebanon | 1996 | 194.509814 | 0.556436159 | 51.75814966 |
| 1983 | Lebanon | 1997 | 195.3636624 | 0.562120818 | 51.75814966 |
| 1984 | Lebanon | 1998 | 196.3092689 | 0.56905907 | 51.75814966 |
| 1985 | Lebanon | 1999 | 197.0909049 | 0.576078528 | 51.75814966 |
| 1986 | Lebanon | 2001 | 197.3313951 | 0.589185502 | 51.75814966 |
| 1987 | Lebanon | 2002 | 196.8998302 | 0.595978788 | 51.75814966 |
| 1988 | Lebanon | 2003 | 196.2688634 | 0.60296724 | 51.75814966 |
| 1989 | Lebanon | 2005 | 194.8584631 | 0.619391355 | 32.04043631 |
| 1990 | Lebanon | 2006 | 193.9564628 | 0.627791217 | 22.46369183 |
| 1991 | Lebanon | 2009 | 189.987943 | 0.657433186 | 22.46369183 |
| 1992 | Lebanon | 2010 | 189.0316548 | 0.668804525 | 22.46369183 |
| 1993 | Lebanon | 2011 | 188.2177645 | 0.680920617 | 17.2540694 |
| 1994 | Lebanon | 2013 | 186.296347 | 0.704811001 | 17.2540694 |
| 1995 | Lebanon | 2014 | 185.4171949 | 0.712423873 | 17.2540694 |
| 1996 | Lebanon | 2015 | 184.7553988 | 0.718697292 | 17.2540694 |
| 1997 | Lebanon | 2016 | 184.1721661 | 0.724583347 | 15.06516239 |
| 1998 | Lebanon | 2018 | 182.9484834 | 0.734746243 | 14.22935117 |
| 1999 | Lebanon | 2020 | 182.4787253 | 0.74200972 | 13.44730416 |
| 2000 | Lebanon | 2021 | 184.7865483 | 0.744746351 | 13.44730416 |
| 2001 | Lesotho | 1990 | 199.9614953 | 0.339155125 | 65.82455226 |
| 2002 | Lesotho | 1993 | 201.4280761 | 0.358780457 | 64.06460795 |
| 2003 | Lesotho | 1995 | 201.6350709 | 0.371265315 | 64.06460795 |
| 2004 | Lesotho | 1997 | 200.7630476 | 0.383096648 | 63.35324861 |
| 2005 | Lesotho | 1998 | 200.0250477 | 0.388406478 | 62.92622144 |
| 2006 | Lesotho | 2000 | 198.9177713 | 0.398635802 | 62.92622144 |
| 2007 | Lesotho | 2001 | 198.5860168 | 0.403990126 | 62.92622144 |
| 2008 | Lesotho | 2005 | 197.1709748 | 0.423211228 | 62.92622144 |
| 2009 | Lesotho | 2006 | 197.1178187 | 0.427806732 | 62.92622144 |
| 2010 | Lesotho | 2008 | 197.2069775 | 0.438340567 | 62.92622144 |
| 2011 | Lesotho | 2009 | 197.3502633 | 0.443580127 | 62.92622144 |
| 2012 | Lesotho | 2011 | 198.1407652 | 0.454530098 | 62.92622144 |
| 2013 | Lesotho | 2012 | 199.1756274 | 0.460419689 | 62.92622144 |
| 2014 | Lesotho | 2014 | 201.4497425 | 0.473188157 | 53.58976548 |
| 2015 | Lesotho | 2015 | 202.1090421 | 0.479754456 | 53.58976548 |
| 2016 | Lesotho | 2017 | 202.7596364 | 0.491978155 | 53.58976548 |
| 2017 | Lesotho | 2018 | 203.0356992 | 0.497381791 | 53.58976548 |
| 2018 | Lesotho | 2019 | 203.2778117 | 0.502457317 | 53.58976548 |
| 2019 | Lesotho | 2020 | 203.9265911 | 0.506746655 | 53.58976548 |
| 2020 | Liberia | 1991 | 268.6816955 | 0.238212218 | 101.1599627 |
| 2021 | Liberia | 1992 | 267.8684827 | 0.238283313 | 101.1599627 |
| 2022 | Liberia | 1993 | 267.2207658 | 0.235984406 | 101.1599627 |
| 2023 | Liberia | 1995 | 266.3529031 | 0.228424478 | 101.1599627 |
| 2024 | Liberia | 1998 | 265.9081706 | 0.219053855 | 101.1599627 |
| 2025 | Liberia | 1999 | 265.9210847 | 0.222212867 | 101.1599627 |
| 2026 | Liberia | 2002 | 266.5457361 | 0.248311971 | 100.8275782 |
| 2027 | Liberia | 2003 | 267.2985264 | 0.251595458 | 100.8275782 |
| 2028 | Liberia | 2004 | 267.9291557 | 0.25517961 | 100.8275782 |
| 2029 | Liberia | 2005 | 268.145739 | 0.25896799 | 100.8275782 |
| 2030 | Liberia | 2007 | 268.1792501 | 0.267239568 | 100.8275782 |
| 2031 | Liberia | 2008 | 268.4073231 | 0.271853596 | 100.8275782 |
| 2032 | Liberia | 2009 | 268.6276522 | 0.276804111 | 100.8275782 |
| 2033 | Liberia | 2010 | 268.6360646 | 0.282235752 | 100.8275782 |
| 2034 | Liberia | 2013 | 270.0799299 | 0.302985656 | 89.96318282 |
| 2035 | Liberia | 2014 | 270.8865674 | 0.31095544 | 89.96318282 |
| 2036 | Liberia | 2015 | 271.2398353 | 0.318485166 | 67.17014806 |
| 2037 | Liberia | 2019 | 273.1733298 | 0.343403262 | 65.82455226 |
| 2038 | Liberia | 2020 | 274.9975902 | 0.34813389 | 65.82455226 |
| 2039 | Libya | 1991 | 256.4169818 | 0.541432399 | 53.31984512 |
| 2040 | Libya | 1993 | 254.3957756 | 0.566798415 | 51.75814966 |
| 2041 | Libya | 1994 | 253.4683124 | 0.578651416 | 51.75814966 |
| 2042 | Libya | 1995 | 252.6559862 | 0.589578634 | 51.75814966 |
| 2043 | Libya | 1996 | 251.8617568 | 0.600350044 | 51.75814966 |
| 2044 | Libya | 1997 | 250.9424837 | 0.610620315 | 51.75814966 |
| 2045 | Libya | 1999 | 248.9895242 | 0.628600604 | 22.46369183 |
| 2046 | Libya | 2000 | 248.0560846 | 0.637047935 | 22.46369183 |
| 2047 | Libya | 2001 | 246.7920058 | 0.644685131 | 22.46369183 |
| 2048 | Libya | 2002 | 245.036829 | 0.651669147 | 22.46369183 |
| 2049 | Libya | 2003 | 243.2371028 | 0.659345125 | 22.46369183 |
| 2050 | Libya | 2004 | 241.8458225 | 0.666913926 | 22.46369183 |
| 2051 | Libya | 2005 | 241.2405851 | 0.675322268 | 22.03195463 |
| 2052 | Libya | 2006 | 242.5372269 | 0.683702 | 17.2540694 |
| 2053 | Libya | 2007 | 245.7405136 | 0.691855134 | 17.2540694 |
| 2054 | Libya | 2008 | 249.6337517 | 0.698873253 | 17.2540694 |
| 2055 | Libya | 2010 | 254.6036138 | 0.711362496 | 17.2540694 |
| 2056 | Libya | 2011 | 254.7827608 | 0.711466657 | 17.2540694 |
| 2057 | Libya | 2012 | 254.7570282 | 0.716700693 | 17.2540694 |
| 2058 | Libya | 2013 | 254.667186 | 0.71766525 | 17.2540694 |
| 2059 | Libya | 2014 | 254.6508731 | 0.716042207 | 17.2540694 |
| 2060 | Libya | 2017 | 257.0887233 | 0.710666264 | 17.2540694 |
| 2061 | Libya | 2018 | 258.6514394 | 0.712720438 | 17.2540694 |
| 2062 | Libya | 2020 | 258.3551619 | 0.720270393 | 15.06516239 |
| 2063 | Lithuania | 1991 | 22.37451201 | 0.673234121 | 22.03195463 |
| 2064 | Lithuania | 1992 | 22.66181495 | 0.68099288 | 17.2540694 |
| 2065 | Lithuania | 1994 | 23.52087785 | 0.691151653 | 17.2540694 |
| 2066 | Lithuania | 1995 | 24.0422149 | 0.69473438 | 17.2540694 |
| 2067 | Lithuania | 1996 | 24.90350506 | 0.698591301 | 17.2540694 |
| 2068 | Lithuania | 1997 | 26.26457545 | 0.70284095 | 17.2540694 |
| 2069 | Lithuania | 1999 | 29.54639347 | 0.712834171 | 17.2540694 |
| 2070 | Lithuania | 2001 | 32.25312342 | 0.727133585 | 14.77524801 |
| 2071 | Lithuania | 2002 | 33.46616897 | 0.733793398 | 14.22935117 |
| 2072 | Lithuania | 2003 | 34.71271941 | 0.741229601 | 13.44730416 |
| 2073 | Lithuania | 2005 | 37.55498205 | 0.75635236 | 10.45018691 |
| 2074 | Lithuania | 2007 | 41.44913716 | 0.770229639 | 9.92126395 |
| 2075 | Lithuania | 2009 | 46.18290332 | 0.785689739 | 8.445315313 |
| 2076 | Lithuania | 2011 | 51.4282643 | 0.800924994 | 8.445315313 |
| 2077 | Lithuania | 2012 | 54.71816599 | 0.807827026 | 8.445315313 |
| 2078 | Lithuania | 2013 | 57.96393193 | 0.813219722 | 8.445315313 |
| 2079 | Lithuania | 2016 | 63.00052202 | 0.829303695 | 8.445315313 |
| 2080 | Lithuania | 2019 | 64.15525649 | 0.847906339 | 8.445315313 |
| 2081 | Lithuania | 2020 | 63.35236537 | 0.852752388 | 8.445315313 |
| 2082 | Luxembourg | 1990 | 37.03495347 | 0.781051609 | 9.92126395 |
| 2083 | Luxembourg | 1992 | 42.62494725 | 0.789053364 | 8.445315313 |
| 2084 | Luxembourg | 1995 | 51.67290601 | 0.80268247 | 8.445315313 |
| 2085 | Luxembourg | 1996 | 55.04378878 | 0.806516138 | 8.445315313 |
| 2086 | Luxembourg | 1999 | 65.98410664 | 0.817570995 | 8.445315313 |
| 2087 | Luxembourg | 2001 | 71.41272357 | 0.825312816 | 8.445315313 |
| 2088 | Luxembourg | 2002 | 73.65927427 | 0.828223491 | 8.445315313 |
| 2089 | Luxembourg | 2003 | 75.72950445 | 0.830983946 | 8.445315313 |
| 2090 | Luxembourg | 2004 | 77.56707865 | 0.833672567 | 8.445315313 |
| 2091 | Luxembourg | 2006 | 80.65802738 | 0.840574453 | 8.445315313 |
| 2092 | Luxembourg | 2007 | 82.31535128 | 0.844707006 | 8.445315313 |
| 2093 | Luxembourg | 2010 | 85.99148728 | 0.853258277 | 8.445315313 |
| 2094 | Luxembourg | 2011 | 85.91513954 | 0.856216834 | 8.445315313 |
| 2095 | Luxembourg | 2012 | 85.24077185 | 0.859789031 | 8.445315313 |
| 2096 | Luxembourg | 2013 | 84.43614792 | 0.863368792 | 8.445315313 |
| 2097 | Luxembourg | 2014 | 83.82026547 | 0.866693556 | 8.445315313 |
| 2098 | Luxembourg | 2017 | 86.44138739 | 0.876347684 | 8.445315313 |
| 2099 | Luxembourg | 2018 | 88.09656972 | 0.878637895 | 8.445315313 |
| 2100 | Luxembourg | 2021 | 62.97354005 | 0.884428955 | 8.445315313 |
| 2101 | Madagascar | 1990 | 299.3587176 | 0.279889465 | 100.8275782 |
| 2102 | Madagascar | 1991 | 298.5434025 | 0.280429589 | 100.8275782 |
| 2103 | Madagascar | 1992 | 297.7599398 | 0.280501283 | 100.8275782 |
| 2104 | Madagascar | 1993 | 297.0333115 | 0.280204299 | 100.8275782 |
| 2105 | Madagascar | 1995 | 295.8727447 | 0.279164003 | 100.8275782 |
| 2106 | Madagascar | 1996 | 295.2515911 | 0.279066493 | 100.8275782 |
| 2107 | Madagascar | 1997 | 294.4931425 | 0.27942619 | 100.8275782 |
| 2108 | Madagascar | 2000 | 292.6435648 | 0.283190721 | 100.8275782 |
| 2109 | Madagascar | 2002 | 292.1153493 | 0.287153149 | 100.8275782 |
| 2110 | Madagascar | 2003 | 291.8530601 | 0.289675457 | 100.8275782 |
| 2111 | Madagascar | 2004 | 291.5945488 | 0.292629885 | 100.8275782 |
| 2112 | Madagascar | 2005 | 291.3851459 | 0.295919327 | 100.8275782 |
| 2113 | Madagascar | 2006 | 291.1928589 | 0.299723392 | 100.8275782 |
| 2114 | Madagascar | 2009 | 290.5498149 | 0.313450891 | 67.91401256 |
| 2115 | Madagascar | 2010 | 290.4148153 | 0.318353184 | 67.17014806 |
| 2116 | Madagascar | 2015 | 290.733649 | 0.350443595 | 65.82455226 |
| 2117 | Madagascar | 2016 | 290.864351 | 0.358026341 | 64.06460795 |
| 2118 | Madagascar | 2017 | 291.1099588 | 0.366235587 | 64.06460795 |
| 2119 | Madagascar | 2018 | 291.4441679 | 0.374969644 | 63.35324861 |
| 2120 | Madagascar | 2019 | 291.8321487 | 0.384209196 | 63.35324861 |
| 2121 | Madagascar | 2020 | 293.4482513 | 0.392778709 | 62.92622144 |
| 2122 | Madagascar | 2021 | 294.5762141 | 0.400246943 | 62.92622144 |
| 2123 | Malawi | 1990 | 268.6113317 | 0.204010245 | 101.1599627 |
| 2124 | Malawi | 1991 | 267.7237552 | 0.206043786 | 101.1599627 |
| 2125 | Malawi | 1993 | 265.8662671 | 0.208091205 | 101.1599627 |
| 2126 | Malawi | 1994 | 264.9547246 | 0.209144566 | 101.1599627 |
| 2127 | Malawi | 1995 | 264.1149344 | 0.211756232 | 101.1599627 |
| 2128 | Malawi | 1997 | 261.9916265 | 0.219416252 | 101.1599627 |
| 2129 | Malawi | 1999 | 259.6978521 | 0.227068368 | 101.1599627 |
| 2130 | Malawi | 2000 | 258.9491668 | 0.23090081 | 101.1599627 |
| 2131 | Malawi | 2001 | 258.4309282 | 0.234020507 | 101.1599627 |
| 2132 | Malawi | 2002 | 257.9309308 | 0.237602155 | 101.1599627 |
| 2133 | Malawi | 2003 | 257.4835511 | 0.241559228 | 100.8275782 |
| 2134 | Malawi | 2004 | 257.1009683 | 0.24643454 | 100.8275782 |
| 2135 | Malawi | 2005 | 256.8100088 | 0.251672318 | 100.8275782 |
| 2136 | Malawi | 2006 | 256.6206718 | 0.257595649 | 100.8275782 |
| 2137 | Malawi | 2009 | 256.5926767 | 0.282296509 | 100.8275782 |
| 2138 | Malawi | 2010 | 256.734143 | 0.29117962 | 100.8275782 |
| 2139 | Malawi | 2011 | 257.2096942 | 0.300448509 | 100.8275782 |
| 2140 | Malawi | 2016 | 261.3347951 | 0.344731542 | 65.82455226 |
| 2141 | Malawi | 2019 | 262.9709435 | 0.370114605 | 64.06460795 |
| 2142 | Malawi | 2020 | 264.1888676 | 0.37787902 | 63.35324861 |
| 2143 | Malaysia | 1991 | 65.45558961 | 0.551683548 | 51.75814966 |
| 2144 | Malaysia | 1993 | 67.69487696 | 0.565845656 | 51.75814966 |
| 2145 | Malaysia | 1994 | 68.33687575 | 0.574380446 | 51.75814966 |
| 2146 | Malaysia | 1996 | 68.4721877 | 0.593152135 | 51.75814966 |
| 2147 | Malaysia | 1997 | 68.14387929 | 0.603418105 | 51.75814966 |
| 2148 | Malaysia | 2000 | 66.63492509 | 0.630523544 | 22.46369183 |
| 2149 | Malaysia | 2001 | 66.01525899 | 0.638257477 | 22.46369183 |
| 2150 | Malaysia | 2003 | 64.19456987 | 0.650885678 | 22.46369183 |
| 2151 | Malaysia | 2004 | 63.13255954 | 0.656243326 | 22.46369183 |
| 2152 | Malaysia | 2005 | 62.06148368 | 0.661242241 | 22.46369183 |
| 2153 | Malaysia | 2006 | 60.74192477 | 0.665956287 | 22.46369183 |
| 2154 | Malaysia | 2007 | 59.11147231 | 0.670843726 | 22.46369183 |
| 2155 | Malaysia | 2008 | 57.47669442 | 0.67639991 | 22.03195463 |
| 2156 | Malaysia | 2009 | 56.15017166 | 0.682070654 | 17.2540694 |
| 2157 | Malaysia | 2012 | 54.95541461 | 0.699664734 | 17.2540694 |
| 2158 | Malaysia | 2013 | 54.79028691 | 0.704930717 | 17.2540694 |
| 2159 | Malaysia | 2015 | 54.70076173 | 0.714659871 | 17.2540694 |
| 2160 | Malaysia | 2016 | 55.12991592 | 0.719223425 | 15.06516239 |
| 2161 | Malaysia | 2017 | 56.02704912 | 0.724235412 | 15.06516239 |
| 2162 | Malaysia | 2018 | 56.95772246 | 0.729388078 | 14.22935117 |
| 2163 | Malaysia | 2019 | 57.48779456 | 0.734587751 | 14.22935117 |
| 2164 | Malaysia | 2020 | 57.40593687 | 0.738743993 | 13.44730416 |
| 2165 | Malaysia | 2021 | 58.90328966 | 0.742523828 | 13.44730416 |
| 2166 | Maldives | 1991 | 114.2049808 | 0.344398607 | 65.82455226 |
| 2167 | Maldives | 1992 | 114.0595262 | 0.35825234 | 64.06460795 |
| 2168 | Maldives | 1993 | 113.980814 | 0.372806999 | 64.06460795 |
| 2169 | Maldives | 1994 | 113.9944521 | 0.388081604 | 62.92622144 |
| 2170 | Maldives | 1996 | 114.3977448 | 0.420519128 | 62.92622144 |
| 2171 | Maldives | 1998 | 115.2039303 | 0.453973361 | 62.92622144 |
| 2172 | Maldives | 2000 | 115.889342 | 0.483495671 | 53.58976548 |
| 2173 | Maldives | 2001 | 115.9090582 | 0.495923695 | 53.58976548 |
| 2174 | Maldives | 2002 | 115.6830949 | 0.50740486 | 53.58976548 |
| 2175 | Maldives | 2003 | 115.4049047 | 0.518926864 | 53.58976548 |
| 2176 | Maldives | 2004 | 115.2773566 | 0.529910191 | 53.58976548 |
| 2177 | Maldives | 2006 | 116.1370599 | 0.547950412 | 51.75814966 |
| 2178 | Maldives | 2008 | 118.318334 | 0.566839634 | 51.75814966 |
| 2179 | Maldives | 2009 | 119.4841481 | 0.57444206 | 51.75814966 |
| 2180 | Maldives | 2010 | 120.4531223 | 0.581980986 | 51.75814966 |
| 2181 | Maldives | 2011 | 121.3420171 | 0.58968446 | 51.75814966 |
| 2182 | Maldives | 2012 | 122.329964 | 0.596948519 | 51.75814966 |
| 2183 | Maldives | 2014 | 124.2040565 | 0.611578071 | 51.75814966 |
| 2184 | Maldives | 2015 | 124.8833995 | 0.61845439 | 32.04043631 |
| 2185 | Maldives | 2017 | 126.1522424 | 0.631083288 | 22.46369183 |
| 2186 | Maldives | 2018 | 126.780385 | 0.637205659 | 22.46369183 |
| 2187 | Maldives | 2019 | 127.2698661 | 0.643082794 | 22.46369183 |
| 2188 | Maldives | 2021 | 130.7700785 | 0.650886627 | 22.46369183 |
| 2189 | Mali | 1991 | 268.5547772 | 0.12912815 | 245.5041638 |
| 2190 | Mali | 1992 | 269.5781057 | 0.131537449 | 245.5041638 |
| 2191 | Mali | 1993 | 270.4546117 | 0.134177054 | 245.5041638 |
| 2192 | Mali | 1994 | 271.1553875 | 0.136937829 | 245.5041638 |
| 2193 | Mali | 1995 | 271.6622699 | 0.139866225 | 245.5041638 |
| 2194 | Mali | 1996 | 272.0455296 | 0.143078916 | 245.5041638 |
| 2195 | Mali | 1998 | 272.7319058 | 0.14998673 | 245.5041638 |
| 2196 | Mali | 1999 | 273.0679856 | 0.153737336 | 245.5041638 |
| 2197 | Mali | 2000 | 273.4312005 | 0.157190028 | 245.5041638 |
| 2198 | Mali | 2001 | 274.0908027 | 0.161344448 | 245.5041638 |
| 2199 | Mali | 2002 | 275.1333236 | 0.165420696 | 245.5041638 |
| 2200 | Mali | 2006 | 278.4660551 | 0.182452468 | 238.2077266 |
| 2201 | Mali | 2007 | 279.0828363 | 0.186912995 | 237.6563045 |
| 2202 | Mali | 2008 | 279.6880165 | 0.191536746 | 237.6563045 |
| 2203 | Mali | 2009 | 280.1616177 | 0.196537651 | 237.6563045 |
| 2204 | Mali | 2010 | 280.3962327 | 0.201803536 | 101.1599627 |
| 2205 | Mali | 2012 | 280.3833479 | 0.21246506 | 101.1599627 |
| 2206 | Mali | 2013 | 280.2807884 | 0.217993867 | 101.1599627 |
| 2207 | Mali | 2014 | 280.1586155 | 0.22372998 | 101.1599627 |
| 2208 | Mali | 2019 | 280.0568016 | 0.256250746 | 100.8275782 |
| 2209 | Mali | 2020 | 281.6207927 | 0.262629219 | 100.8275782 |
| 2210 | Mali | 2021 | 283.1654082 | 0.268579941 | 100.8275782 |
| 2211 | Malta | 1991 | 33.3980826 | 0.661112691 | 22.46369183 |
| 2212 | Malta | 1992 | 33.9232097 | 0.666324648 | 22.46369183 |
| 2213 | Malta | 1994 | 34.87167925 | 0.677847314 | 22.03195463 |
| 2214 | Malta | 1997 | 35.78160531 | 0.691756406 | 17.2540694 |
| 2215 | Malta | 1998 | 36.04273158 | 0.698095287 | 17.2540694 |
| 2216 | Malta | 2000 | 37.05803569 | 0.711805254 | 17.2540694 |
| 2217 | Malta | 2001 | 38.72728249 | 0.717803038 | 17.2540694 |
| 2218 | Malta | 2002 | 41.73439171 | 0.722790275 | 15.06516239 |
| 2219 | Malta | 2004 | 48.90380245 | 0.731933853 | 14.22935117 |
| 2220 | Malta | 2006 | 53.62016559 | 0.740140181 | 13.44730416 |
| 2221 | Malta | 2007 | 55.28367794 | 0.744237812 | 13.44730416 |
| 2222 | Malta | 2008 | 56.82425883 | 0.748427095 | 13.44730416 |
| 2223 | Malta | 2009 | 58.43009994 | 0.752171059 | 13.44730416 |
| 2224 | Malta | 2010 | 60.310197 | 0.7562505 | 10.45018691 |
| 2225 | Malta | 2011 | 63.0162614 | 0.760310547 | 9.92126395 |
| 2226 | Malta | 2012 | 66.5667262 | 0.76453788 | 9.92126395 |
| 2227 | Malta | 2013 | 70.33384496 | 0.76861614 | 9.92126395 |
| 2228 | Malta | 2014 | 73.69824062 | 0.772728741 | 9.92126395 |
| 2229 | Malta | 2015 | 76.07572773 | 0.777276905 | 9.92126395 |
| 2230 | Malta | 2017 | 79.355847 | 0.786410184 | 8.445315313 |
| 2231 | Malta | 2020 | 66.72907862 | 0.798375166 | 8.445315313 |
| 2232 | Malta | 2021 | 61.47878958 | 0.801585034 | 8.445315313 |
| 2233 | Marshall Islands | 1990 | 130.0781116 | 0.430839288 | 62.92622144 |
| 2234 | Marshall Islands | 1992 | 129.1728798 | 0.440491018 | 62.92622144 |
| 2235 | Marshall Islands | 1994 | 128.2957336 | 0.450848221 | 62.92622144 |
| 2236 | Marshall Islands | 1995 | 127.8241043 | 0.456859674 | 62.92622144 |
| 2237 | Marshall Islands | 1996 | 127.4478183 | 0.46120943 | 62.92622144 |
| 2238 | Marshall Islands | 1998 | 127.0095399 | 0.468133032 | 53.58976548 |
| 2239 | Marshall Islands | 1999 | 126.8054298 | 0.471292727 | 53.58976548 |
| 2240 | Marshall Islands | 2000 | 126.4677598 | 0.474562536 | 53.58976548 |
| 2241 | Marshall Islands | 2001 | 126.0062145 | 0.478588316 | 53.58976548 |
| 2242 | Marshall Islands | 2005 | 124.0956481 | 0.493271391 | 53.58976548 |
| 2243 | Marshall Islands | 2006 | 123.5825287 | 0.497456175 | 53.58976548 |
| 2244 | Marshall Islands | 2007 | 122.939289 | 0.502250042 | 53.58976548 |
| 2245 | Marshall Islands | 2009 | 121.7265842 | 0.510794536 | 53.58976548 |
| 2246 | Marshall Islands | 2010 | 121.3532188 | 0.516085138 | 53.58976548 |
| 2247 | Marshall Islands | 2013 | 120.669823 | 0.5314463 | 53.58976548 |
| 2248 | Marshall Islands | 2014 | 120.5092279 | 0.536640131 | 53.31984512 |
| 2249 | Marshall Islands | 2016 | 120.5906069 | 0.546782936 | 51.75814966 |
| 2250 | Marshall Islands | 2017 | 120.8764066 | 0.552065152 | 51.75814966 |
| 2251 | Marshall Islands | 2019 | 121.3460795 | 0.56342875 | 51.75814966 |
| 2252 | Marshall Islands | 2021 | 121.3470593 | 0.574091128 | 51.75814966 |
| 2253 | Mauritania | 1990 | 266.0099457 | 0.335780942 | 65.82455226 |
| 2254 | Mauritania | 1992 | 267.1652515 | 0.346531288 | 65.82455226 |
| 2255 | Mauritania | 1993 | 267.8163166 | 0.352176572 | 65.82455226 |
| 2256 | Mauritania | 1994 | 268.3554061 | 0.357585912 | 64.06460795 |
| 2257 | Mauritania | 1996 | 269.4247183 | 0.368508328 | 64.06460795 |
| 2258 | Mauritania | 1998 | 270.9435235 | 0.37708025 | 63.35324861 |
| 2259 | Mauritania | 2001 | 273.3384376 | 0.386487416 | 63.35324861 |
| 2260 | Mauritania | 2002 | 274.6148389 | 0.388564478 | 62.92622144 |
| 2261 | Mauritania | 2003 | 275.9894444 | 0.391104879 | 62.92622144 |
| 2262 | Mauritania | 2005 | 278.1609631 | 0.397499105 | 62.92622144 |
| 2263 | Mauritania | 2006 | 279.0563043 | 0.403159467 | 62.92622144 |
| 2264 | Mauritania | 2008 | 281.4926391 | 0.413233621 | 62.92622144 |
| 2265 | Mauritania | 2009 | 282.4993749 | 0.417910609 | 62.92622144 |
| 2266 | Mauritania | 2010 | 283.1427661 | 0.423202436 | 62.92622144 |
| 2267 | Mauritania | 2011 | 283.544927 | 0.42886729 | 62.92622144 |
| 2268 | Mauritania | 2012 | 283.9220636 | 0.434724376 | 62.92622144 |
| 2269 | Mauritania | 2013 | 284.3557242 | 0.44106891 | 62.92622144 |
| 2270 | Mauritania | 2014 | 284.5819821 | 0.447653881 | 62.92622144 |
| 2271 | Mauritania | 2016 | 284.8479128 | 0.461269545 | 62.92622144 |
| 2272 | Mauritania | 2020 | 287.0187186 | 0.491365603 | 53.58976548 |
| 2273 | Mauritania | 2021 | 288.2646519 | 0.4989451 | 53.58976548 |
| 2274 | Mauritius | 1990 | 169.7743607 | 0.544586533 | 51.75814966 |
| 2275 | Mauritius | 1991 | 171.288341 | 0.548209121 | 51.75814966 |
| 2276 | Mauritius | 1992 | 172.2448358 | 0.55345463 | 51.75814966 |
| 2277 | Mauritius | 1993 | 172.7648634 | 0.56068864 | 51.75814966 |
| 2278 | Mauritius | 1996 | 171.2925377 | 0.584797061 | 51.75814966 |
| 2279 | Mauritius | 1997 | 167.7100579 | 0.591497735 | 51.75814966 |
| 2280 | Mauritius | 1998 | 163.4749362 | 0.597363039 | 51.75814966 |
| 2281 | Mauritius | 2000 | 158.426747 | 0.607087901 | 51.75814966 |
| 2282 | Mauritius | 2001 | 159.3647176 | 0.612240817 | 51.75814966 |
| 2283 | Mauritius | 2002 | 161.7770998 | 0.617190007 | 32.04043631 |
| 2284 | Mauritius | 2003 | 164.9172413 | 0.622764533 | 32.04043631 |
| 2285 | Mauritius | 2004 | 168.01698 | 0.629030193 | 22.46369183 |
| 2286 | Mauritius | 2005 | 170.3392557 | 0.63476658 | 22.46369183 |
| 2287 | Mauritius | 2006 | 171.7706152 | 0.639545183 | 22.46369183 |
| 2288 | Mauritius | 2007 | 172.8802145 | 0.644358656 | 22.46369183 |
| 2289 | Mauritius | 2009 | 175.2869797 | 0.655000295 | 22.46369183 |
| 2290 | Mauritius | 2010 | 177.1594841 | 0.6604698 | 22.46369183 |
| 2291 | Mauritius | 2013 | 191.2276146 | 0.680114023 | 22.03195463 |
| 2292 | Mauritius | 2014 | 196.4782422 | 0.686616503 | 17.2540694 |
| 2293 | Mauritius | 2017 | 205.9527314 | 0.701618136 | 17.2540694 |
| 2294 | Mauritius | 2018 | 208.1162506 | 0.706266651 | 17.2540694 |
| 2295 | Mauritius | 2019 | 209.3358019 | 0.710935045 | 17.2540694 |
| 2296 | Mauritius | 2020 | 209.1693104 | 0.714629804 | 17.2540694 |
| 2297 | Mexico | 1990 | 110.4719942 | 0.504996083 | 53.58976548 |
| 2298 | Mexico | 1991 | 110.4496758 | 0.50951067 | 53.58976548 |
| 2299 | Mexico | 1992 | 110.3064375 | 0.514638057 | 53.58976548 |
| 2300 | Mexico | 1993 | 110.0636163 | 0.520493167 | 53.58976548 |
| 2301 | Mexico | 1998 | 106.7434324 | 0.547842121 | 51.75814966 |
| 2302 | Mexico | 1999 | 105.6048552 | 0.55381337 | 51.75814966 |
| 2303 | Mexico | 2000 | 104.5675932 | 0.559532547 | 51.75814966 |
| 2304 | Mexico | 2001 | 103.410492 | 0.564774176 | 51.75814966 |
| 2305 | Mexico | 2002 | 101.9892472 | 0.570113531 | 51.75814966 |
| 2306 | Mexico | 2003 | 100.5721204 | 0.575698215 | 51.75814966 |
| 2307 | Mexico | 2004 | 99.42221324 | 0.580984982 | 51.75814966 |
| 2308 | Mexico | 2007 | 98.69485877 | 0.591447408 | 51.75814966 |
| 2309 | Mexico | 2008 | 98.79549377 | 0.594454558 | 51.75814966 |
| 2310 | Mexico | 2009 | 98.87254777 | 0.596684358 | 51.75814966 |
| 2311 | Mexico | 2010 | 98.82707811 | 0.599527028 | 51.75814966 |
| 2312 | Mexico | 2011 | 98.4746067 | 0.603646095 | 51.75814966 |
| 2313 | Mexico | 2012 | 97.83490628 | 0.608914289 | 51.75814966 |
| 2314 | Mexico | 2013 | 97.12619892 | 0.615089993 | 32.04043631 |
| 2315 | Mexico | 2014 | 96.56240923 | 0.621857405 | 32.04043631 |
| 2316 | Mexico | 2015 | 96.359262 | 0.628896724 | 22.46369183 |
| 2317 | Mexico | 2017 | 97.58019817 | 0.642667198 | 22.46369183 |
| 2318 | Mexico | 2019 | 98.96557385 | 0.655095338 | 22.46369183 |
| 2319 | Mexico | 2020 | 99.0156557 | 0.660119062 | 22.46369183 |
| 2320 | Mexico | 2021 | 100.0390578 | 0.664575304 | 22.46369183 |
| 2321 | Micronesia (Federated States of) | 1991 | 132.3148354 | 0.468144831 | 53.58976548 |
| 2322 | Micronesia (Federated States of) | 1992 | 132.416977 | 0.473792936 | 53.58976548 |
| 2323 | Micronesia (Federated States of) | 1993 | 132.4743632 | 0.479939595 | 53.58976548 |
| 2324 | Micronesia (Federated States of) | 1995 | 132.4539713 | 0.491015185 | 53.58976548 |
| 2325 | Micronesia (Federated States of) | 1996 | 132.3274689 | 0.495570308 | 53.58976548 |
| 2326 | Micronesia (Federated States of) | 1998 | 131.7770309 | 0.502861564 | 53.58976548 |
| 2327 | Micronesia (Federated States of) | 2001 | 130.8212935 | 0.515316814 | 53.58976548 |
| 2328 | Micronesia (Federated States of) | 2003 | 130.0331108 | 0.523829599 | 53.58976548 |
| 2329 | Micronesia (Federated States of) | 2005 | 129.2968186 | 0.531703321 | 53.58976548 |
| 2330 | Micronesia (Federated States of) | 2007 | 128.3644551 | 0.539431464 | 53.31984512 |
| 2331 | Micronesia (Federated States of) | 2009 | 127.3269086 | 0.546275496 | 51.75814966 |
| 2332 | Micronesia (Federated States of) | 2010 | 127.030378 | 0.550122546 | 51.75814966 |
| 2333 | Micronesia (Federated States of) | 2011 | 126.9259773 | 0.554240411 | 51.75814966 |
| 2334 | Micronesia (Federated States of) | 2012 | 126.9396339 | 0.557965853 | 51.75814966 |
| 2335 | Micronesia (Federated States of) | 2013 | 127.0283579 | 0.561082821 | 51.75814966 |
| 2336 | Micronesia (Federated States of) | 2016 | 127.3750525 | 0.570458101 | 51.75814966 |
| 2337 | Micronesia (Federated States of) | 2017 | 127.534661 | 0.574039833 | 51.75814966 |
| 2338 | Micronesia (Federated States of) | 2019 | 127.8402344 | 0.581195255 | 51.75814966 |
| 2339 | Micronesia (Federated States of) | 2020 | 128.1677513 | 0.584527229 | 51.75814966 |
| 2340 | Micronesia (Federated States of) | 2021 | 128.1711406 | 0.587534967 | 51.75814966 |
| 2341 | Monaco | 1990 | 50.01962116 | 0.845495153 | 8.445315313 |
| 2342 | Monaco | 1992 | 51.55599284 | 0.85086394 | 8.445315313 |
| 2343 | Monaco | 1993 | 52.67455286 | 0.853264992 | 8.445315313 |
| 2344 | Monaco | 1994 | 53.95946263 | 0.855626927 | 8.445315313 |
| 2345 | Monaco | 1995 | 55.35578056 | 0.857986147 | 8.445315313 |
| 2346 | Monaco | 1996 | 57.08393083 | 0.860245432 | 8.445315313 |
| 2347 | Monaco | 1997 | 59.27214351 | 0.86244672 | 8.445315313 |
| 2348 | Monaco | 1998 | 61.72399456 | 0.864716513 | 8.445315313 |
| 2349 | Monaco | 2001 | 68.9672512 | 0.871094755 | 8.445315313 |
| 2350 | Monaco | 2002 | 71.46958708 | 0.873182769 | 8.445315313 |
| 2351 | Monaco | 2003 | 73.91251776 | 0.875197652 | 8.445315313 |
| 2352 | Monaco | 2004 | 76.10367195 | 0.877231549 | 8.445315313 |
| 2353 | Monaco | 2005 | 77.83551775 | 0.879288089 | 8.445315313 |
| 2354 | Monaco | 2007 | 80.69291083 | 0.883262291 | 8.445315313 |
| 2355 | Monaco | 2008 | 81.92157768 | 0.885331394 | 8.445315313 |
| 2356 | Monaco | 2009 | 82.84752541 | 0.887328279 | 8.445315313 |
| 2357 | Monaco | 2010 | 83.32022064 | 0.889262841 | 8.445315313 |
| 2358 | Monaco | 2011 | 83.47704215 | 0.891237592 | 8.445315313 |
| 2359 | Monaco | 2012 | 83.56191997 | 0.893073822 | 8.445315313 |
| 2360 | Monaco | 2013 | 83.61440457 | 0.894864472 | 8.445315313 |
| 2361 | Monaco | 2014 | 83.65688489 | 0.896547874 | 8.445315313 |
| 2362 | Monaco | 2015 | 83.70350388 | 0.898356337 | 8.445315313 |
| 2363 | Monaco | 2017 | 84.00268951 | 0.901752712 | 8.445315313 |
| 2364 | Monaco | 2018 | 84.17615253 | 0.903388687 | 8.445315313 |
| 2365 | Monaco | 2019 | 84.25811829 | 0.905030991 | 8.445315313 |
| 2366 | Monaco | 2021 | 79.76611316 | 0.908262831 | 8.445315313 |
| 2367 | Mongolia | 1990 | 53.58976548 | 0.466550118 | 53.58976548 |
| 2368 | Mongolia | 1991 | 53.61317175 | 0.472199014 | 53.58976548 |
| 2369 | Mongolia | 1992 | 53.61847663 | 0.477586554 | 53.58976548 |
| 2370 | Mongolia | 1994 | 53.63002312 | 0.488276683 | 53.58976548 |
| 2371 | Mongolia | 1995 | 53.61779063 | 0.495006781 | 53.58976548 |
| 2372 | Mongolia | 1996 | 53.62251326 | 0.501703718 | 53.58976548 |
| 2373 | Mongolia | 1999 | 53.70458035 | 0.520781189 | 53.58976548 |
| 2374 | Mongolia | 2000 | 53.66881611 | 0.526650345 | 53.58976548 |
| 2375 | Mongolia | 2001 | 53.31984512 | 0.532620201 | 53.31984512 |
| 2376 | Mongolia | 2003 | 51.75814966 | 0.543971651 | 51.75814966 |
| 2377 | Mongolia | 2009 | 52.57852257 | 0.571341686 | 51.75814966 |
| 2378 | Mongolia | 2010 | 52.95762681 | 0.574406669 | 51.75814966 |
| 2379 | Mongolia | 2011 | 53.11782611 | 0.578600663 | 51.75814966 |
| 2380 | Mongolia | 2013 | 53.02878925 | 0.588080811 | 51.75814966 |
| 2381 | Mongolia | 2015 | 53.02913841 | 0.596859648 | 51.75814966 |
| 2382 | Mongolia | 2016 | 53.22314666 | 0.600260767 | 51.75814966 |
| 2383 | Mongolia | 2018 | 53.70447399 | 0.607501833 | 51.75814966 |
| 2384 | Mongolia | 2019 | 53.87607966 | 0.611250681 | 51.75814966 |
| 2385 | Mongolia | 2020 | 53.00423164 | 0.614629637 | 32.04043631 |
| 2386 | Montenegro | 1994 | 58.3125729 | 0.665011931 | 22.46369183 |
| 2387 | Montenegro | 1997 | 58.49002138 | 0.665542032 | 22.46369183 |
| 2388 | Montenegro | 1998 | 58.68075947 | 0.669024771 | 22.46369183 |
| 2389 | Montenegro | 1999 | 58.82587102 | 0.672096103 | 22.03195463 |
| 2390 | Montenegro | 2000 | 58.8627665 | 0.67730685 | 22.03195463 |
| 2391 | Montenegro | 2001 | 58.72189491 | 0.683433454 | 17.2540694 |
| 2392 | Montenegro | 2003 | 58.12563719 | 0.696517506 | 17.2540694 |
| 2393 | Montenegro | 2004 | 57.86299881 | 0.702998276 | 17.2540694 |
| 2394 | Montenegro | 2005 | 57.74350731 | 0.709065465 | 17.2540694 |
| 2395 | Montenegro | 2006 | 57.82765503 | 0.715459074 | 17.2540694 |
| 2396 | Montenegro | 2007 | 58.06712163 | 0.72250394 | 15.06516239 |
| 2397 | Montenegro | 2008 | 58.39565501 | 0.729981756 | 14.22935117 |
| 2398 | Montenegro | 2010 | 59.08357666 | 0.743347873 | 13.44730416 |
| 2399 | Montenegro | 2012 | 60.17699784 | 0.75557838 | 10.45018691 |
| 2400 | Montenegro | 2014 | 61.383843 | 0.765776527 | 9.92126395 |
| 2401 | Montenegro | 2016 | 61.52631936 | 0.77520077 | 9.92126395 |
| 2402 | Montenegro | 2017 | 61.26915941 | 0.779764632 | 9.92126395 |
| 2403 | Montenegro | 2019 | 60.80495453 | 0.789026903 | 8.445315313 |
| 2404 | Montenegro | 2020 | 60.89487226 | 0.792554393 | 8.445315313 |
| 2405 | Morocco | 1990 | 230.6322728 | 0.35807287 | 64.06460795 |
| 2406 | Morocco | 1991 | 229.6667805 | 0.364984629 | 64.06460795 |
| 2407 | Morocco | 1992 | 228.7121294 | 0.371187194 | 64.06460795 |
| 2408 | Morocco | 1994 | 226.9639566 | 0.382849451 | 63.35324861 |
| 2409 | Morocco | 1995 | 226.234072 | 0.38816411 | 62.92622144 |
| 2410 | Morocco | 1996 | 225.4276901 | 0.394150096 | 62.92622144 |
| 2411 | Morocco | 1997 | 224.4697251 | 0.399504009 | 62.92622144 |
| 2412 | Morocco | 1999 | 222.8213915 | 0.410303191 | 62.92622144 |
| 2413 | Morocco | 2000 | 222.5132197 | 0.415344026 | 62.92622144 |
| 2414 | Morocco | 2001 | 222.7312793 | 0.420595133 | 62.92622144 |
| 2415 | Morocco | 2002 | 223.3150567 | 0.42577575 | 62.92622144 |
| 2416 | Morocco | 2003 | 224.1191187 | 0.43121781 | 62.92622144 |
| 2417 | Morocco | 2004 | 224.9722283 | 0.436822352 | 62.92622144 |
| 2418 | Morocco | 2005 | 225.7190934 | 0.442531339 | 62.92622144 |
| 2419 | Morocco | 2006 | 226.5495261 | 0.44867616 | 62.92622144 |
| 2420 | Morocco | 2011 | 231.818954 | 0.483922152 | 53.58976548 |
| 2421 | Morocco | 2013 | 234.2271037 | 0.499528607 | 53.58976548 |
| 2422 | Morocco | 2014 | 235.1759359 | 0.507443265 | 53.58976548 |
| 2423 | Morocco | 2016 | 235.3829147 | 0.523518592 | 53.58976548 |
| 2424 | Morocco | 2017 | 234.6435575 | 0.531610862 | 53.58976548 |
| 2425 | Morocco | 2018 | 233.8335958 | 0.539729671 | 53.31984512 |
| 2426 | Morocco | 2020 | 232.6318905 | 0.555246069 | 51.75814966 |
| 2427 | Morocco | 2021 | 232.2069471 | 0.562698301 | 51.75814966 |
| 2428 | Mozambique | 1991 | 239.0367772 | 0.175550435 | 239.0367772 |
| 2429 | Mozambique | 1992 | 238.7663549 | 0.177285188 | 238.7663549 |
| 2430 | Mozambique | 1994 | 238.2077266 | 0.182236861 | 238.2077266 |
| 2431 | Mozambique | 1996 | 237.6563045 | 0.185705869 | 237.6563045 |
| 2432 | Mozambique | 2000 | 236.3318337 | 0.198534397 | 236.3318337 |
| 2433 | Mozambique | 2001 | 236.4154985 | 0.202241924 | 101.1599627 |
| 2434 | Mozambique | 2002 | 236.8963701 | 0.20639006 | 101.1599627 |
| 2435 | Mozambique | 2004 | 238.0772059 | 0.214972457 | 101.1599627 |
| 2436 | Mozambique | 2006 | 238.1052236 | 0.224758087 | 101.1599627 |
| 2437 | Mozambique | 2007 | 237.8408046 | 0.230158227 | 101.1599627 |
| 2438 | Mozambique | 2008 | 237.5478303 | 0.23579017 | 101.1599627 |
| 2439 | Mozambique | 2009 | 237.3073335 | 0.241360656 | 100.8275782 |
| 2440 | Mozambique | 2010 | 237.202685 | 0.2467515 | 100.8275782 |
| 2441 | Mozambique | 2011 | 237.3771593 | 0.252232471 | 100.8275782 |
| 2442 | Mozambique | 2012 | 237.8049018 | 0.258315562 | 100.8275782 |
| 2443 | Mozambique | 2013 | 238.3177097 | 0.265280462 | 100.8275782 |
| 2444 | Mozambique | 2014 | 238.736571 | 0.272892814 | 100.8275782 |
| 2445 | Mozambique | 2018 | 236.9341524 | 0.305226772 | 89.96318282 |
| 2446 | Mozambique | 2019 | 236.5043217 | 0.313174508 | 67.91401256 |
| 2447 | Mozambique | 2020 | 237.6422929 | 0.320291182 | 66.46502371 |
| 2448 | Mozambique | 2021 | 239.2011998 | 0.326462614 | 66.46502371 |
| 2449 | Myanmar | 1990 | 162.7734589 | 0.319219724 | 66.46502371 |
| 2450 | Myanmar | 1994 | 162.3071298 | 0.33429012 | 65.82455226 |
| 2451 | Myanmar | 1995 | 162.0717055 | 0.339590976 | 65.82455226 |
| 2452 | Myanmar | 1998 | 160.7429477 | 0.356696902 | 64.06460795 |
| 2453 | Myanmar | 1999 | 160.2115942 | 0.363037732 | 64.06460795 |
| 2454 | Myanmar | 2000 | 159.7184255 | 0.370360017 | 64.06460795 |
| 2455 | Myanmar | 2001 | 159.1213195 | 0.37817198 | 63.35324861 |
| 2456 | Myanmar | 2003 | 157.5012829 | 0.395218766 | 62.92622144 |
| 2457 | Myanmar | 2004 | 156.7516606 | 0.404692358 | 62.92622144 |
| 2458 | Myanmar | 2006 | 155.8389299 | 0.424705071 | 62.92622144 |
| 2459 | Myanmar | 2008 | 155.1248306 | 0.443595531 | 62.92622144 |
| 2460 | Myanmar | 2009 | 154.8881613 | 0.451954884 | 62.92622144 |
| 2461 | Myanmar | 2010 | 154.8225004 | 0.460180429 | 62.92622144 |
| 2462 | Myanmar | 2011 | 154.9975056 | 0.467850872 | 53.58976548 |
| 2463 | Myanmar | 2013 | 156.028542 | 0.482821855 | 53.58976548 |
| 2464 | Myanmar | 2016 | 158.3281348 | 0.504115978 | 53.58976548 |
| 2465 | Myanmar | 2018 | 161.2414934 | 0.517557469 | 53.58976548 |
| 2466 | Myanmar | 2019 | 162.134186 | 0.52410897 | 53.58976548 |
| 2467 | Myanmar | 2021 | 162.6736418 | 0.53390084 | 53.31984512 |
| 2468 | Namibia | 1990 | 205.2887142 | 0.450040233 | 62.92622144 |
| 2469 | Namibia | 1991 | 205.4924763 | 0.45453444 | 62.92622144 |
| 2470 | Namibia | 1992 | 205.7154356 | 0.460021314 | 62.92622144 |
| 2471 | Namibia | 1993 | 205.9371624 | 0.465734135 | 62.92622144 |
| 2472 | Namibia | 1995 | 206.28784 | 0.477794329 | 53.58976548 |
| 2473 | Namibia | 1997 | 206.8547584 | 0.488012938 | 53.58976548 |
| 2474 | Namibia | 2000 | 207.9289001 | 0.502406958 | 53.58976548 |
| 2475 | Namibia | 2002 | 208.7076164 | 0.510818275 | 53.58976548 |
| 2476 | Namibia | 2003 | 209.2692265 | 0.514574645 | 53.58976548 |
| 2477 | Namibia | 2004 | 209.852671 | 0.519103605 | 53.58976548 |
| 2478 | Namibia | 2007 | 212.2043117 | 0.535062126 | 53.31984512 |
| 2479 | Namibia | 2008 | 213.4505712 | 0.540896995 | 53.31984512 |
| 2480 | Namibia | 2009 | 214.682582 | 0.546516477 | 51.75814966 |
| 2481 | Namibia | 2010 | 215.7151132 | 0.552741565 | 51.75814966 |
| 2482 | Namibia | 2014 | 220.548026 | 0.582321425 | 51.75814966 |
| 2483 | Namibia | 2015 | 221.4654416 | 0.58982104 | 51.75814966 |
| 2484 | Namibia | 2016 | 222.3374336 | 0.596283059 | 51.75814966 |
| 2485 | Namibia | 2018 | 224.3239417 | 0.606618403 | 51.75814966 |
| 2486 | Namibia | 2019 | 224.9578358 | 0.610921091 | 51.75814966 |
| 2487 | Namibia | 2021 | 227.128331 | 0.617564872 | 32.04043631 |
| 2488 | Nauru | 1990 | 106.8726755 | 0.539145981 | 53.31984512 |
| 2489 | Nauru | 1991 | 106.331524 | 0.538433557 | 53.31984512 |
| 2490 | Nauru | 1993 | 105.1641505 | 0.535059742 | 53.31984512 |
| 2491 | Nauru | 1994 | 104.560973 | 0.533001097 | 53.31984512 |
| 2492 | Nauru | 1996 | 103.2530178 | 0.528544474 | 53.58976548 |
| 2493 | Nauru | 1998 | 101.5194299 | 0.523885302 | 53.58976548 |
| 2494 | Nauru | 1999 | 100.7119659 | 0.521894324 | 53.58976548 |
| 2495 | Nauru | 2000 | 100.1001234 | 0.520254888 | 53.58976548 |
| 2496 | Nauru | 2001 | 99.58791502 | 0.519235425 | 53.58976548 |
| 2497 | Nauru | 2002 | 99.02638354 | 0.518438705 | 53.58976548 |
| 2498 | Nauru | 2004 | 97.91656564 | 0.51880954 | 53.58976548 |
| 2499 | Nauru | 2005 | 97.43791385 | 0.519892863 | 53.58976548 |
| 2500 | Nauru | 2006 | 96.91926022 | 0.522673179 | 53.58976548 |
| 2501 | Nauru | 2007 | 96.30066945 | 0.52320135 | 53.58976548 |
| 2502 | Nauru | 2008 | 95.69190618 | 0.526084096 | 53.58976548 |
| 2503 | Nauru | 2009 | 95.20140114 | 0.530221092 | 53.58976548 |
| 2504 | Nauru | 2010 | 94.9374746 | 0.535689995 | 53.31984512 |
| 2505 | Nauru | 2011 | 94.89503192 | 0.542342743 | 53.31984512 |
| 2506 | Nauru | 2013 | 95.05638524 | 0.560112906 | 51.75814966 |
| 2507 | Nauru | 2014 | 95.14911601 | 0.571424364 | 51.75814966 |
| 2508 | Nauru | 2015 | 95.17205871 | 0.581283234 | 51.75814966 |
| 2509 | Nauru | 2016 | 95.02862663 | 0.590102988 | 51.75814966 |
| 2510 | Nauru | 2020 | 94.79477642 | 0.619330076 | 32.04043631 |
| 2511 | Nauru | 2021 | 93.21662449 | 0.625177834 | 32.04043631 |
| 2512 | Nepal | 1990 | 101.1599627 | 0.199560654 | 101.1599627 |
| 2513 | Nepal | 1996 | 100.8275782 | 0.240700616 | 100.8275782 |
| 2514 | Nepal | 1997 | 100.9087802 | 0.248382797 | 100.8275782 |
| 2515 | Nepal | 1999 | 101.1284634 | 0.26435918 | 100.8275782 |
| 2516 | Nepal | 2000 | 101.2099327 | 0.272719115 | 100.8275782 |
| 2517 | Nepal | 2002 | 101.351612 | 0.289513608 | 100.8275782 |
| 2518 | Nepal | 2003 | 101.4375861 | 0.297772595 | 100.8275782 |
| 2519 | Nepal | 2004 | 101.54169 | 0.30619653 | 89.96318282 |
| 2520 | Nepal | 2005 | 101.6674231 | 0.314661796 | 67.17014806 |
| 2521 | Nepal | 2007 | 102.2115148 | 0.33144561 | 65.82455226 |
| 2522 | Nepal | 2008 | 102.6555941 | 0.340150706 | 65.82455226 |
| 2523 | Nepal | 2009 | 103.1924439 | 0.348701893 | 65.82455226 |
| 2524 | Nepal | 2010 | 103.8098341 | 0.3568681 | 64.06460795 |
| 2525 | Nepal | 2011 | 104.8660981 | 0.364642605 | 64.06460795 |
| 2526 | Nepal | 2012 | 106.5408032 | 0.372437588 | 64.06460795 |
| 2527 | Nepal | 2015 | 112.265364 | 0.395021154 | 62.92622144 |
| 2528 | Nepal | 2016 | 113.7487326 | 0.401628776 | 62.92622144 |
| 2529 | Nepal | 2017 | 115.2222662 | 0.408463874 | 62.92622144 |
| 2530 | Nepal | 2018 | 116.6199674 | 0.415236684 | 62.92622144 |
| 2531 | Nepal | 2020 | 119.281762 | 0.427417089 | 62.92622144 |
| 2532 | Nepal | 2021 | 121.8412459 | 0.433174635 | 62.92622144 |
| 2533 | Netherlands | 1990 | 18.42257256 | 0.794612123 | 8.445315313 |
| 2534 | Netherlands | 1991 | 18.45772839 | 0.799285437 | 8.445315313 |
| 2535 | Netherlands | 1992 | 18.57330076 | 0.803965978 | 8.445315313 |
| 2536 | Netherlands | 1993 | 18.75115412 | 0.808278967 | 8.445315313 |
| 2537 | Netherlands | 1994 | 18.97786353 | 0.812530216 | 8.445315313 |
| 2538 | Netherlands | 1995 | 19.23484026 | 0.816662769 | 8.445315313 |
| 2539 | Netherlands | 1998 | 21.85709196 | 0.825882234 | 8.445315313 |
| 2540 | Netherlands | 1999 | 23.02827694 | 0.828693827 | 8.445315313 |
| 2541 | Netherlands | 2001 | 24.79654204 | 0.835518148 | 8.445315313 |
| 2542 | Netherlands | 2002 | 25.48309464 | 0.838676077 | 8.445315313 |
| 2543 | Netherlands | 2003 | 26.18344426 | 0.841639477 | 8.445315313 |
| 2544 | Netherlands | 2006 | 29.89345846 | 0.850798808 | 8.445315313 |
| 2545 | Netherlands | 2011 | 39.33372407 | 0.864246678 | 8.445315313 |
| 2546 | Netherlands | 2012 | 39.20827341 | 0.866950626 | 8.445315313 |
| 2547 | Netherlands | 2013 | 39.00946353 | 0.869402979 | 8.445315313 |
| 2548 | Netherlands | 2014 | 38.85906302 | 0.871848914 | 8.445315313 |
| 2549 | Netherlands | 2018 | 41.66211247 | 0.881900218 | 8.445315313 |
| 2550 | Netherlands | 2019 | 42.41861739 | 0.88464384 | 8.445315313 |
| 2551 | Netherlands | 2020 | 43.02868607 | 0.886558566 | 8.445315313 |
| 2552 | New Zealand | 1990 | 33.65249854 | 0.752321655 | 13.44730416 |
| 2553 | New Zealand | 1992 | 33.6090727 | 0.759461372 | 9.92126395 |
| 2554 | New Zealand | 1995 | 33.27118501 | 0.769998036 | 9.92126395 |
| 2555 | New Zealand | 1998 | 31.98926505 | 0.781730757 | 9.92126395 |
| 2556 | New Zealand | 2002 | 30.58747304 | 0.794519591 | 8.445315313 |
| 2557 | New Zealand | 2006 | 30.25740394 | 0.798456866 | 8.445315313 |
| 2558 | New Zealand | 2008 | 30.64846852 | 0.799669514 | 8.445315313 |
| 2559 | New Zealand | 2010 | 31.11440545 | 0.805133185 | 8.445315313 |
| 2560 | New Zealand | 2011 | 31.43492277 | 0.809760352 | 8.445315313 |
| 2561 | New Zealand | 2012 | 31.91773644 | 0.814957564 | 8.445315313 |
| 2562 | New Zealand | 2014 | 33.05244299 | 0.823908944 | 8.445315313 |
| 2563 | New Zealand | 2016 | 33.89743374 | 0.831843967 | 8.445315313 |
| 2564 | New Zealand | 2017 | 34.28911448 | 0.836301795 | 8.445315313 |
| 2565 | New Zealand | 2018 | 34.72896161 | 0.840597863 | 8.445315313 |
| 2566 | New Zealand | 2020 | 36.2187889 | 0.847398737 | 8.445315313 |
| 2567 | New Zealand | 2021 | 32.22889515 | 0.849442499 | 8.445315313 |
| 2568 | Nicaragua | 1990 | 164.4462753 | 0.346035235 | 65.82455226 |
| 2569 | Nicaragua | 1991 | 164.3935129 | 0.351740622 | 65.82455226 |
| 2570 | Nicaragua | 1992 | 164.2855857 | 0.357712704 | 64.06460795 |
| 2571 | Nicaragua | 1994 | 163.978072 | 0.369727968 | 64.06460795 |
| 2572 | Nicaragua | 1995 | 163.8227515 | 0.376625345 | 63.35324861 |
| 2573 | Nicaragua | 1997 | 163.1669419 | 0.391775893 | 62.92622144 |
| 2574 | Nicaragua | 1999 | 162.0131931 | 0.408085965 | 62.92622144 |
| 2575 | Nicaragua | 2000 | 161.3543397 | 0.416108363 | 62.92622144 |
| 2576 | Nicaragua | 2004 | 154.6796074 | 0.443274511 | 62.92622144 |
| 2577 | Nicaragua | 2005 | 153.2377761 | 0.448844739 | 62.92622144 |
| 2578 | Nicaragua | 2009 | 149.1168323 | 0.467961839 | 53.58976548 |
| 2579 | Nicaragua | 2010 | 148.4123564 | 0.472172 | 53.58976548 |
| 2580 | Nicaragua | 2012 | 147.0942618 | 0.4812969 | 53.58976548 |
| 2581 | Nicaragua | 2015 | 145.6355466 | 0.496391663 | 53.58976548 |
| 2582 | Nicaragua | 2019 | 145.3270833 | 0.516597046 | 53.58976548 |
| 2583 | Niger | 1990 | 264.4736059 | 0.08086848 | 245.5041638 |
| 2584 | Niger | 1991 | 265.0336154 | 0.082848208 | 245.5041638 |
| 2585 | Niger | 1992 | 265.5332296 | 0.084771578 | 245.5041638 |
| 2586 | Niger | 1994 | 266.3857745 | 0.088608733 | 245.5041638 |
| 2587 | Niger | 1996 | 266.9362465 | 0.092346359 | 245.5041638 |
| 2588 | Niger | 1997 | 267.0505915 | 0.094219115 | 245.5041638 |
| 2589 | Niger | 1999 | 267.114663 | 0.098813686 | 245.5041638 |
| 2590 | Niger | 2000 | 267.2638455 | 0.100886948 | 245.5041638 |
| 2591 | Niger | 2001 | 267.8016284 | 0.103271777 | 245.5041638 |
| 2592 | Niger | 2003 | 269.8089605 | 0.108322891 | 245.5041638 |
| 2593 | Niger | 2005 | 271.3011399 | 0.113452023 | 245.5041638 |
| 2594 | Niger | 2007 | 272.0398593 | 0.118971626 | 245.5041638 |
| 2595 | Niger | 2008 | 272.4257309 | 0.121957786 | 245.5041638 |
| 2596 | Niger | 2012 | 272.2311179 | 0.134469718 | 245.5041638 |
| 2597 | Niger | 2013 | 271.9401553 | 0.13797817 | 245.5041638 |
| 2598 | Niger | 2014 | 271.5789967 | 0.141731738 | 245.5041638 |
| 2599 | Niger | 2015 | 271.1378634 | 0.145440503 | 245.5041638 |
| 2600 | Niger | 2016 | 270.4449776 | 0.149200109 | 245.5041638 |
| 2601 | Niger | 2018 | 268.3220954 | 0.156875196 | 245.5041638 |
| 2602 | Niger | 2019 | 267.6953858 | 0.160889484 | 245.5041638 |
| 2603 | Niger | 2020 | 269.0469558 | 0.164539265 | 245.5041638 |
| 2604 | Niger | 2021 | 269.8671052 | 0.168072774 | 245.5041638 |
| 2605 | Nigeria | 1990 | 250.073355 | 0.305868047 | 89.96318282 |
| 2606 | Nigeria | 1992 | 251.2389938 | 0.311234052 | 89.96318282 |
| 2607 | Nigeria | 1994 | 252.137366 | 0.316747447 | 67.17014806 |
| 2608 | Nigeria | 1996 | 252.5407971 | 0.323137685 | 66.46502371 |
| 2609 | Nigeria | 1997 | 252.4694336 | 0.326690553 | 66.46502371 |
| 2610 | Nigeria | 2001 | 254.6402319 | 0.343029751 | 65.82455226 |
| 2611 | Nigeria | 2002 | 258.8813138 | 0.349092528 | 65.82455226 |
| 2612 | Nigeria | 2003 | 263.8691537 | 0.355911392 | 65.82455226 |
| 2613 | Nigeria | 2004 | 268.1398022 | 0.363780814 | 64.06460795 |
| 2614 | Nigeria | 2005 | 270.2150291 | 0.372583553 | 64.06460795 |
| 2615 | Nigeria | 2006 | 270.5401754 | 0.38102604 | 63.35324861 |
| 2616 | Nigeria | 2007 | 270.5965753 | 0.389779649 | 62.92622144 |
| 2617 | Nigeria | 2008 | 270.4611818 | 0.398037706 | 62.92622144 |
| 2618 | Nigeria | 2009 | 270.2454839 | 0.406353307 | 62.92622144 |
| 2619 | Nigeria | 2013 | 269.4584886 | 0.440124664 | 62.92622144 |
| 2620 | Nigeria | 2015 | 267.8898345 | 0.457240991 | 62.92622144 |
| 2621 | Nigeria | 2017 | 261.1123445 | 0.472972869 | 53.58976548 |
| 2622 | Nigeria | 2018 | 256.9860265 | 0.480844413 | 53.58976548 |
| 2623 | Nigeria | 2019 | 254.6756798 | 0.488787361 | 53.58976548 |
| 2624 | Nigeria | 2020 | 255.5626746 | 0.496204741 | 53.58976548 |
| 2625 | Niue | 1992 | 127.9088479 | 0.599881243 | 51.75814966 |
| 2626 | Niue | 1993 | 127.3136495 | 0.605514779 | 51.75814966 |
| 2627 | Niue | 1994 | 126.7277134 | 0.610650175 | 51.75814966 |
| 2628 | Niue | 1998 | 124.3258353 | 0.627578934 | 22.46369183 |
| 2629 | Niue | 1999 | 123.7557792 | 0.630857172 | 22.46369183 |
| 2630 | Niue | 2001 | 122.7408517 | 0.637366627 | 22.46369183 |
| 2631 | Niue | 2002 | 122.2349108 | 0.641122021 | 22.46369183 |
| 2632 | Niue | 2003 | 121.7311259 | 0.64577954 | 22.46369183 |
| 2633 | Niue | 2004 | 121.2599015 | 0.650614832 | 22.46369183 |
| 2634 | Niue | 2006 | 120.5062246 | 0.664162983 | 22.46369183 |
| 2635 | Niue | 2008 | 119.8591804 | 0.676396859 | 22.03195463 |
| 2636 | Niue | 2011 | 118.8976358 | 0.690315578 | 17.2540694 |
| 2637 | Niue | 2014 | 117.7176509 | 0.700545924 | 17.2540694 |
| 2638 | Niue | 2015 | 117.5682115 | 0.704232979 | 17.2540694 |
| 2639 | Niue | 2016 | 117.6730897 | 0.707476099 | 17.2540694 |
| 2640 | Niue | 2017 | 117.9618778 | 0.711220996 | 17.2540694 |
| 2641 | Niue | 2019 | 118.5380901 | 0.719078885 | 17.2540694 |
| 2642 | Niue | 2021 | 119.2392555 | 0.72622205 | 14.77524801 |
| 2643 | North Macedonia | 1990 | 134.6581464 | 0.609026094 | 51.75814966 |
| 2644 | North Macedonia | 1991 | 134.459416 | 0.611337453 | 51.75814966 |
| 2645 | North Macedonia | 1992 | 134.3827591 | 0.612708856 | 51.75814966 |
| 2646 | North Macedonia | 1994 | 134.4758272 | 0.614362905 | 32.04043631 |
| 2647 | North Macedonia | 1995 | 134.6393315 | 0.616994878 | 32.04043631 |
| 2648 | North Macedonia | 1996 | 135.1780404 | 0.620944017 | 32.04043631 |
| 2649 | North Macedonia | 1997 | 136.2451544 | 0.626223811 | 32.04043631 |
| 2650 | North Macedonia | 1999 | 139.0883357 | 0.636687259 | 22.46369183 |
| 2651 | North Macedonia | 2002 | 143.2508612 | 0.65539879 | 22.46369183 |
| 2652 | North Macedonia | 2005 | 147.779722 | 0.678047273 | 22.03195463 |
| 2653 | North Macedonia | 2007 | 151.2186291 | 0.689729481 | 17.2540694 |
| 2654 | North Macedonia | 2008 | 153.0905568 | 0.695376955 | 17.2540694 |
| 2655 | North Macedonia | 2010 | 155.4462382 | 0.705589324 | 17.2540694 |
| 2656 | North Macedonia | 2011 | 155.8020891 | 0.710592521 | 17.2540694 |
| 2657 | North Macedonia | 2012 | 156.0963055 | 0.715173148 | 17.2540694 |
| 2658 | North Macedonia | 2013 | 156.3050979 | 0.719671149 | 15.06516239 |
| 2659 | North Macedonia | 2016 | 156.0822417 | 0.733173624 | 14.22935117 |
| 2660 | North Macedonia | 2017 | 155.5402225 | 0.737339955 | 13.44730416 |
| 2661 | North Macedonia | 2019 | 154.501108 | 0.745193569 | 13.44730416 |
| 2662 | North Macedonia | 2020 | 153.8726506 | 0.7478499 | 13.44730416 |
| 2663 | North Macedonia | 2021 | 145.1120101 | 0.750629703 | 13.44730416 |
| 2664 | Northern Mariana Islands | 1990 | 72.11111447 | 0.708593838 | 17.2540694 |
| 2665 | Northern Mariana Islands | 1991 | 71.5118414 | 0.712223642 | 17.2540694 |
| 2666 | Northern Mariana Islands | 1993 | 70.69381989 | 0.719179396 | 15.06516239 |
| 2667 | Northern Mariana Islands | 1994 | 70.36753267 | 0.722510494 | 15.06516239 |
| 2668 | Northern Mariana Islands | 1996 | 69.97879004 | 0.730074086 | 14.22935117 |
| 2669 | Northern Mariana Islands | 1997 | 69.87992997 | 0.733795366 | 14.22935117 |
| 2670 | Northern Mariana Islands | 1998 | 69.75946387 | 0.737423127 | 13.44730416 |
| 2671 | Northern Mariana Islands | 1999 | 69.58891354 | 0.740934815 | 13.44730416 |
| 2672 | Northern Mariana Islands | 2003 | 68.3624755 | 0.750171533 | 13.44730416 |
| 2673 | Northern Mariana Islands | 2004 | 67.77762739 | 0.751528841 | 13.44730416 |
| 2674 | Northern Mariana Islands | 2006 | 66.93498278 | 0.751797809 | 13.44730416 |
| 2675 | Northern Mariana Islands | 2007 | 66.64495848 | 0.751608416 | 13.44730416 |
| 2676 | Northern Mariana Islands | 2010 | 65.19623458 | 0.746617262 | 13.44730416 |
| 2677 | Northern Mariana Islands | 2011 | 64.7287458 | 0.744411908 | 13.44730416 |
| 2678 | Northern Mariana Islands | 2013 | 63.56836592 | 0.74228436 | 13.44730416 |
| 2679 | Northern Mariana Islands | 2014 | 62.9382457 | 0.742605449 | 13.44730416 |
| 2680 | Northern Mariana Islands | 2016 | 62.5362818 | 0.748455466 | 13.44730416 |
| 2681 | Northern Mariana Islands | 2017 | 62.62512506 | 0.756495479 | 10.45018691 |
| 2682 | Northern Mariana Islands | 2018 | 62.65757805 | 0.7612554 | 9.92126395 |
| 2683 | Northern Mariana Islands | 2019 | 62.58890666 | 0.765770647 | 9.92126395 |
| 2684 | Northern Mariana Islands | 2021 | 63.24815637 | 0.771535213 | 9.92126395 |
| 2685 | Norway | 1990 | 16.19583344 | 0.795887277 | 8.445315313 |
| 2686 | Norway | 1991 | 16.27513102 | 0.800468886 | 8.445315313 |
| 2687 | Norway | 1993 | 16.4476331 | 0.810565689 | 8.445315313 |
| 2688 | Norway | 1995 | 16.62483306 | 0.820223522 | 8.445315313 |
| 2689 | Norway | 1997 | 16.85772238 | 0.83146877 | 8.445315313 |
| 2690 | Norway | 2001 | 17.33649067 | 0.852111936 | 8.445315313 |
| 2691 | Norway | 2002 | 17.53521532 | 0.856757888 | 8.445315313 |
| 2692 | Norway | 2004 | 18.09027049 | 0.863468908 | 8.445315313 |
| 2693 | Norway | 2005 | 18.42331424 | 0.866217488 | 8.445315313 |
| 2694 | Norway | 2006 | 18.91575555 | 0.868588435 | 8.445315313 |
| 2695 | Norway | 2007 | 19.639118 | 0.87092967 | 8.445315313 |
| 2696 | Norway | 2009 | 21.36169905 | 0.876582817 | 8.445315313 |
| 2697 | Norway | 2011 | 22.93274554 | 0.884351667 | 8.445315313 |
| 2698 | Norway | 2013 | 24.57676563 | 0.891989618 | 8.445315313 |
| 2699 | Norway | 2014 | 25.32202391 | 0.895499546 | 8.445315313 |
| 2700 | Norway | 2018 | 27.39942718 | 0.909261782 | 8.445315313 |
| 2701 | Norway | 2019 | 27.70798531 | 0.912278761 | 8.445315313 |
| 2702 | Norway | 2021 | 26.80122331 | 0.91613281 | 8.445315313 |
| 2703 | Oman | 1990 | 194.47644 | 0.429270949 | 62.92622144 |
| 2704 | Oman | 1991 | 194.2287905 | 0.442268122 | 62.92622144 |
| 2705 | Oman | 1994 | 194.4349955 | 0.493513107 | 53.58976548 |
| 2706 | Oman | 1995 | 194.6866753 | 0.513194359 | 53.58976548 |
| 2707 | Oman | 1996 | 195.4160948 | 0.533246394 | 53.31984512 |
| 2708 | Oman | 1998 | 198.4878725 | 0.574474902 | 51.75814966 |
| 2709 | Oman | 2000 | 201.7353752 | 0.608421541 | 51.75814966 |
| 2710 | Oman | 2001 | 203.0261996 | 0.622160627 | 32.04043631 |
| 2711 | Oman | 2002 | 204.3256032 | 0.635162126 | 22.46369183 |
| 2712 | Oman | 2003 | 205.7052077 | 0.647249093 | 22.46369183 |
| 2713 | Oman | 2004 | 207.1750176 | 0.657626118 | 22.46369183 |
| 2714 | Oman | 2005 | 208.7617486 | 0.666401231 | 22.46369183 |
| 2715 | Oman | 2006 | 210.5456037 | 0.675353885 | 22.03195463 |
| 2716 | Oman | 2010 | 220.3960228 | 0.710688695 | 17.2540694 |
| 2717 | Oman | 2011 | 226.1992138 | 0.718264528 | 17.2540694 |
| 2718 | Oman | 2012 | 235.105948 | 0.726450074 | 14.77524801 |
| 2719 | Oman | 2014 | 253.338707 | 0.738743772 | 13.44730416 |
| 2720 | Oman | 2015 | 257.9925867 | 0.743597182 | 13.44730416 |
| 2721 | Oman | 2017 | 261.3793866 | 0.753786058 | 10.45018691 |
| 2722 | Oman | 2018 | 262.5199411 | 0.759179514 | 9.92126395 |
| 2723 | Oman | 2020 | 262.4458839 | 0.768854216 | 9.92126395 |
| 2724 | Pakistan | 1990 | 119.7023822 | 0.310467621 | 89.96318282 |
| 2725 | Pakistan | 1991 | 119.7692091 | 0.316265667 | 67.17014806 |
| 2726 | Pakistan | 1993 | 120.0230031 | 0.328219252 | 65.82455226 |
| 2727 | Pakistan | 1995 | 120.4119743 | 0.340926296 | 65.82455226 |
| 2728 | Pakistan | 1996 | 120.7337863 | 0.347656203 | 65.82455226 |
| 2729 | Pakistan | 1999 | 122.2324356 | 0.367641397 | 64.06460795 |
| 2730 | Pakistan | 2000 | 122.5968019 | 0.374319132 | 64.06460795 |
| 2731 | Pakistan | 2002 | 123.337541 | 0.386629992 | 63.35324861 |
| 2732 | Pakistan | 2003 | 123.7611998 | 0.392719263 | 62.92622144 |
| 2733 | Pakistan | 2004 | 124.1095499 | 0.399084167 | 62.92622144 |
| 2734 | Pakistan | 2005 | 124.29466 | 0.405667437 | 62.92622144 |
| 2735 | Pakistan | 2006 | 124.3075965 | 0.41223483 | 62.92622144 |
| 2736 | Pakistan | 2007 | 124.2147536 | 0.418519756 | 62.92622144 |
| 2737 | Pakistan | 2008 | 124.0545587 | 0.4243482 | 62.92622144 |
| 2738 | Pakistan | 2010 | 123.6743299 | 0.435640657 | 62.92622144 |
| 2739 | Pakistan | 2012 | 122.5059273 | 0.447096599 | 62.92622144 |
| 2740 | Pakistan | 2013 | 121.6691967 | 0.452823095 | 62.92622144 |
| 2741 | Pakistan | 2016 | 120.7770408 | 0.471022168 | 53.58976548 |
| 2742 | Pakistan | 2017 | 121.1037456 | 0.477544137 | 53.58976548 |
| 2743 | Pakistan | 2018 | 121.4970637 | 0.484326128 | 53.58976548 |
| 2744 | Palau | 1991 | 56.87144341 | 0.668230731 | 22.46369183 |
| 2745 | Palau | 1992 | 56.38604619 | 0.673302462 | 22.03195463 |
| 2746 | Palau | 1993 | 55.93988108 | 0.676648715 | 22.03195463 |
| 2747 | Palau | 1995 | 55.07227178 | 0.68483214 | 17.2540694 |
| 2748 | Palau | 1996 | 54.76339046 | 0.690095673 | 17.2540694 |
| 2749 | Palau | 1998 | 54.43507768 | 0.69965291 | 17.2540694 |
| 2750 | Palau | 2001 | 53.81584414 | 0.707402386 | 17.2540694 |
| 2751 | Palau | 2003 | 53.32824799 | 0.71100022 | 17.2540694 |
| 2752 | Palau | 2004 | 53.09297691 | 0.712543811 | 17.2540694 |
| 2753 | Palau | 2006 | 52.74905022 | 0.716353431 | 17.2540694 |
| 2754 | Palau | 2007 | 52.57737029 | 0.718809542 | 17.2540694 |
| 2755 | Palau | 2008 | 52.38276612 | 0.720806623 | 15.06516239 |
| 2756 | Palau | 2010 | 51.98858869 | 0.724065956 | 15.06516239 |
| 2757 | Palau | 2011 | 51.76835551 | 0.726733446 | 14.77524801 |
| 2758 | Palau | 2012 | 51.46785561 | 0.729612899 | 14.22935117 |
| 2759 | Palau | 2013 | 51.14049596 | 0.731953475 | 14.22935117 |
| 2760 | Palau | 2014 | 50.86610053 | 0.734768297 | 14.22935117 |
| 2761 | Palau | 2015 | 50.73797121 | 0.738166425 | 13.44730416 |
| 2762 | Palau | 2016 | 50.87156605 | 0.741361911 | 13.44730416 |
| 2763 | Palau | 2020 | 51.61082422 | 0.752780929 | 13.44730416 |
| 2764 | Palestine | 1990 | 232.9715871 | 0.40179221 | 62.92622144 |
| 2765 | Palestine | 1991 | 232.1837446 | 0.40572715 | 62.92622144 |
| 2766 | Palestine | 1992 | 231.4062928 | 0.411146869 | 62.92622144 |
| 2767 | Palestine | 1995 | 229.3571296 | 0.429307027 | 62.92622144 |
| 2768 | Palestine | 1996 | 228.7061892 | 0.434731371 | 62.92622144 |
| 2769 | Palestine | 1997 | 227.9598635 | 0.440901776 | 62.92622144 |
| 2770 | Palestine | 2000 | 226.0973927 | 0.460681496 | 62.92622144 |
| 2771 | Palestine | 2001 | 225.7278633 | 0.465299102 | 62.92622144 |
| 2772 | Palestine | 2002 | 225.3441065 | 0.468600257 | 53.58976548 |
| 2773 | Palestine | 2003 | 225.0123748 | 0.472881732 | 53.58976548 |
| 2774 | Palestine | 2004 | 224.7662424 | 0.478527322 | 53.58976548 |
| 2775 | Palestine | 2006 | 224.7776187 | 0.491890407 | 53.58976548 |
| 2776 | Palestine | 2008 | 225.7017912 | 0.506849479 | 53.58976548 |
| 2777 | Palestine | 2009 | 226.2873139 | 0.515815836 | 53.58976548 |
| 2778 | Palestine | 2010 | 226.7957654 | 0.525711905 | 53.58976548 |
| 2779 | Palestine | 2011 | 227.3365916 | 0.53677763 | 53.31984512 |
| 2780 | Palestine | 2013 | 228.8357069 | 0.559163481 | 51.75814966 |
| 2781 | Palestine | 2015 | 230.2617047 | 0.579204261 | 51.75814966 |
| 2782 | Palestine | 2019 | 233.573358 | 0.616466839 | 32.04043631 |
| 2783 | Panama | 1991 | 91.0446768 | 0.550484218 | 51.75814966 |
| 2784 | Panama | 1992 | 92.09343376 | 0.554972755 | 51.75814966 |
| 2785 | Panama | 1993 | 93.1817759 | 0.559168666 | 51.75814966 |
| 2786 | Panama | 1994 | 94.28998112 | 0.561714534 | 51.75814966 |
| 2787 | Panama | 1996 | 96.77608562 | 0.56531007 | 51.75814966 |
| 2788 | Panama | 1997 | 98.47591377 | 0.569395939 | 51.75814966 |
| 2789 | Panama | 2001 | 101.8651395 | 0.594535701 | 51.75814966 |
| 2790 | Panama | 2002 | 101.3340557 | 0.600138677 | 51.75814966 |
| 2791 | Panama | 2003 | 100.686857 | 0.604043365 | 51.75814966 |
| 2792 | Panama | 2005 | 100.0448267 | 0.609494125 | 51.75814966 |
| 2793 | Panama | 2007 | 102.5343438 | 0.615285417 | 32.04043631 |
| 2794 | Panama | 2008 | 104.8409615 | 0.618958907 | 32.04043631 |
| 2795 | Panama | 2009 | 107.3341909 | 0.622402806 | 32.04043631 |
| 2796 | Panama | 2010 | 109.6222943 | 0.625116323 | 32.04043631 |
| 2797 | Panama | 2011 | 111.7371405 | 0.628633942 | 22.46369183 |
| 2798 | Panama | 2014 | 119.0304645 | 0.649108174 | 22.46369183 |
| 2799 | Panama | 2015 | 121.8670122 | 0.657962047 | 22.46369183 |
| 2800 | Panama | 2016 | 125.8309135 | 0.667276758 | 22.46369183 |
| 2801 | Panama | 2017 | 130.8121354 | 0.676783503 | 22.03195463 |
| 2802 | Panama | 2018 | 135.3239527 | 0.686719925 | 17.2540694 |
| 2803 | Panama | 2019 | 137.8819267 | 0.69667068 | 17.2540694 |
| 2804 | Panama | 2021 | 135.9126207 | 0.708864828 | 17.2540694 |
| 2805 | Papua New Guinea | 1990 | 110.1984559 | 0.310668629 | 89.96318282 |
| 2806 | Papua New Guinea | 1991 | 109.7652508 | 0.314967305 | 67.17014806 |
| 2807 | Papua New Guinea | 1993 | 109.0756966 | 0.325005984 | 66.46502371 |
| 2808 | Papua New Guinea | 1994 | 108.7652068 | 0.330664468 | 65.82455226 |
| 2809 | Papua New Guinea | 1996 | 108.2314237 | 0.340348698 | 65.82455226 |
| 2810 | Papua New Guinea | 1997 | 107.9981852 | 0.344258325 | 65.82455226 |
| 2811 | Papua New Guinea | 1999 | 107.4756891 | 0.351250621 | 65.82455226 |
| 2812 | Papua New Guinea | 2005 | 103.4074786 | 0.365294965 | 64.06460795 |
| 2813 | Papua New Guinea | 2006 | 102.9073684 | 0.367826759 | 64.06460795 |
| 2814 | Papua New Guinea | 2008 | 102.0562183 | 0.373426209 | 64.06460795 |
| 2815 | Papua New Guinea | 2009 | 101.7282799 | 0.376528285 | 63.35324861 |
| 2816 | Papua New Guinea | 2010 | 101.5269619 | 0.380286778 | 63.35324861 |
| 2817 | Papua New Guinea | 2011 | 101.3503331 | 0.383587539 | 63.35324861 |
| 2818 | Papua New Guinea | 2013 | 101.1015218 | 0.389926437 | 62.92622144 |
| 2819 | Papua New Guinea | 2014 | 101.0365703 | 0.394004571 | 62.92622144 |
| 2820 | Papua New Guinea | 2015 | 101.0670858 | 0.398285864 | 62.92622144 |
| 2821 | Papua New Guinea | 2016 | 101.4434617 | 0.402407267 | 62.92622144 |
| 2822 | Papua New Guinea | 2017 | 102.1971982 | 0.406153435 | 62.92622144 |
| 2823 | Papua New Guinea | 2018 | 103.0057347 | 0.409165083 | 62.92622144 |
| 2824 | Papua New Guinea | 2020 | 103.8577147 | 0.415186879 | 62.92622144 |
| 2825 | Paraguay | 1990 | 170.692171 | 0.469527785 | 53.58976548 |
| 2826 | Paraguay | 1991 | 171.6232633 | 0.474352286 | 53.58976548 |
| 2827 | Paraguay | 1993 | 173.9308748 | 0.4846142 | 53.58976548 |
| 2828 | Paraguay | 1994 | 175.2386846 | 0.490226935 | 53.58976548 |
| 2829 | Paraguay | 1995 | 176.5996976 | 0.496336876 | 53.58976548 |
| 2830 | Paraguay | 1997 | 180.5974293 | 0.508286948 | 53.58976548 |
| 2831 | Paraguay | 1998 | 183.0202376 | 0.513819896 | 53.58976548 |
| 2832 | Paraguay | 1999 | 185.2737242 | 0.518720583 | 53.58976548 |
| 2833 | Paraguay | 2000 | 187.022174 | 0.523007793 | 53.58976548 |
| 2834 | Paraguay | 2002 | 189.9666877 | 0.531177358 | 53.58976548 |
| 2835 | Paraguay | 2003 | 191.3517228 | 0.535360126 | 53.31984512 |
| 2836 | Paraguay | 2004 | 192.4770947 | 0.539556655 | 53.31984512 |
| 2837 | Paraguay | 2005 | 193.1947869 | 0.543622697 | 53.31984512 |
| 2838 | Paraguay | 2008 | 194.0996326 | 0.558016903 | 51.75814966 |
| 2839 | Paraguay | 2009 | 194.3194731 | 0.562900059 | 51.75814966 |
| 2840 | Paraguay | 2010 | 194.5821635 | 0.568846562 | 51.75814966 |
| 2841 | Paraguay | 2011 | 195.1573441 | 0.574954463 | 51.75814966 |
| 2842 | Paraguay | 2012 | 196.0934402 | 0.580650186 | 51.75814966 |
| 2843 | Paraguay | 2013 | 197.104209 | 0.587247042 | 51.75814966 |
| 2844 | Paraguay | 2014 | 197.917538 | 0.593953153 | 51.75814966 |
| 2845 | Paraguay | 2016 | 197.6420169 | 0.607018145 | 51.75814966 |
| 2846 | Paraguay | 2019 | 194.1321978 | 0.626070139 | 32.04043631 |
| 2847 | Paraguay | 2020 | 195.0891407 | 0.631057689 | 22.46369183 |
| 2848 | Peru | 1990 | 127.3891646 | 0.510419852 | 53.58976548 |
| 2849 | Peru | 1992 | 127.8467634 | 0.513821552 | 53.58976548 |
| 2850 | Peru | 1994 | 128.4580764 | 0.51930608 | 53.58976548 |
| 2851 | Peru | 1995 | 128.7743821 | 0.523640527 | 53.58976548 |
| 2852 | Peru | 1996 | 129.2603801 | 0.528312042 | 53.58976548 |
| 2853 | Peru | 1997 | 129.9885865 | 0.533576491 | 53.31984512 |
| 2854 | Peru | 1998 | 130.8033161 | 0.538721717 | 53.31984512 |
| 2855 | Peru | 1999 | 131.5461063 | 0.543853377 | 53.31984512 |
| 2856 | Peru | 2000 | 132.0564469 | 0.548952201 | 51.75814966 |
| 2857 | Peru | 2003 | 132.9511109 | 0.563871072 | 51.75814966 |
| 2858 | Peru | 2004 | 133.1810389 | 0.569063024 | 51.75814966 |
| 2859 | Peru | 2005 | 133.3648975 | 0.574517001 | 51.75814966 |
| 2860 | Peru | 2007 | 133.7583974 | 0.586958489 | 51.75814966 |
| 2861 | Peru | 2009 | 134.0376857 | 0.599954993 | 51.75814966 |
| 2862 | Peru | 2011 | 133.6664514 | 0.613051221 | 51.75814966 |
| 2863 | Peru | 2012 | 133.0134898 | 0.619355264 | 32.04043631 |
| 2864 | Peru | 2013 | 132.2659251 | 0.625519024 | 32.04043631 |
| 2865 | Peru | 2016 | 131.4349353 | 0.641285279 | 22.46369183 |
| 2866 | Peru | 2017 | 131.7099523 | 0.646180855 | 22.46369183 |
| 2867 | Peru | 2018 | 132.0131403 | 0.650977317 | 22.46369183 |
| 2868 | Peru | 2021 | 126.8482686 | 0.662054037 | 22.46369183 |
| 2869 | Philippines | 1990 | 191.7419842 | 0.510011796 | 53.58976548 |
| 2870 | Philippines | 1991 | 191.364209 | 0.513707183 | 53.58976548 |
| 2871 | Philippines | 1992 | 191.0265018 | 0.517109502 | 53.58976548 |
| 2872 | Philippines | 1994 | 190.5672637 | 0.524613607 | 53.58976548 |
| 2873 | Philippines | 1999 | 190.810182 | 0.542257968 | 53.31984512 |
| 2874 | Philippines | 2000 | 190.9233188 | 0.544997723 | 51.75814966 |
| 2875 | Philippines | 2001 | 191.0534349 | 0.547409432 | 51.75814966 |
| 2876 | Philippines | 2003 | 191.5640471 | 0.551567968 | 51.75814966 |
| 2877 | Philippines | 2005 | 192.320246 | 0.555664103 | 51.75814966 |
| 2878 | Philippines | 2006 | 193.0634459 | 0.557976265 | 51.75814966 |
| 2879 | Philippines | 2008 | 195.547781 | 0.563974721 | 51.75814966 |
| 2880 | Philippines | 2009 | 196.7383636 | 0.56735582 | 51.75814966 |
| 2881 | Philippines | 2010 | 197.5389084 | 0.571765888 | 51.75814966 |
| 2882 | Philippines | 2011 | 197.9781931 | 0.577240283 | 51.75814966 |
| 2883 | Philippines | 2012 | 198.3252514 | 0.583767625 | 51.75814966 |
| 2884 | Philippines | 2015 | 199.2694652 | 0.606215624 | 51.75814966 |
| 2885 | Philippines | 2016 | 199.9459034 | 0.614224961 | 32.04043631 |
| 2886 | Philippines | 2017 | 200.952409 | 0.622315029 | 32.04043631 |
| 2887 | Philippines | 2018 | 201.9260811 | 0.630464276 | 22.46369183 |
| 2888 | Philippines | 2019 | 202.5255491 | 0.638547384 | 22.46369183 |
| 2889 | Philippines | 2020 | 202.5313659 | 0.644867233 | 22.46369183 |
| 2890 | Poland | 1990 | 119.1290351 | 0.627227888 | 22.46369183 |
| 2891 | Poland | 1991 | 117.8295443 | 0.632671471 | 22.46369183 |
| 2892 | Poland | 1995 | 116.8683125 | 0.666678867 | 22.46369183 |
| 2893 | Poland | 1998 | 126.3980293 | 0.690805671 | 17.2540694 |
| 2894 | Poland | 1999 | 131.2416017 | 0.698536056 | 17.2540694 |
| 2895 | Poland | 2000 | 135.5800436 | 0.706662052 | 17.2540694 |
| 2896 | Poland | 2001 | 140.2040238 | 0.714831304 | 17.2540694 |
| 2897 | Poland | 2003 | 151.4061769 | 0.728155254 | 14.22935117 |
| 2898 | Poland | 2004 | 156.0308031 | 0.733673192 | 14.22935117 |
| 2899 | Poland | 2007 | 157.9624975 | 0.746008817 | 13.44730416 |
| 2900 | Poland | 2009 | 155.4037058 | 0.756427378 | 10.45018691 |
| 2901 | Poland | 2016 | 180.3725347 | 0.790651015 | 8.445315313 |
| 2902 | Poland | 2017 | 180.5018735 | 0.794530345 | 8.445315313 |
| 2903 | Poland | 2019 | 180.2679985 | 0.804791332 | 8.445315313 |
| 2904 | Poland | 2021 | 183.2109486 | 0.812042809 | 8.445315313 |
| 2905 | Portugal | 1991 | 55.20530893 | 0.607269449 | 51.75814966 |
| 2906 | Portugal | 1992 | 56.20868234 | 0.61480808 | 32.04043631 |
| 2907 | Portugal | 1993 | 57.25019236 | 0.622101177 | 32.04043631 |
| 2908 | Portugal | 1994 | 58.30886272 | 0.629306387 | 22.46369183 |
| 2909 | Portugal | 1996 | 60.50465965 | 0.640864526 | 22.46369183 |
| 2910 | Portugal | 1997 | 61.79975544 | 0.646192904 | 22.46369183 |
| 2911 | Portugal | 1998 | 63.19136628 | 0.651335994 | 22.46369183 |
| 2912 | Portugal | 1999 | 64.6254309 | 0.656120667 | 22.46369183 |
| 2913 | Portugal | 2000 | 66.0455373 | 0.661610852 | 22.46369183 |
| 2914 | Portugal | 2001 | 67.56985878 | 0.667247151 | 22.46369183 |
| 2915 | Portugal | 2002 | 69.2546088 | 0.67258394 | 22.03195463 |
| 2916 | Portugal | 2003 | 70.94874862 | 0.677663412 | 22.03195463 |
| 2917 | Portugal | 2006 | 74.88088168 | 0.691158795 | 17.2540694 |
| 2918 | Portugal | 2008 | 77.0544493 | 0.698604599 | 17.2540694 |
| 2919 | Portugal | 2009 | 78.00911922 | 0.702223399 | 17.2540694 |
| 2920 | Portugal | 2013 | 81.31697756 | 0.719836991 | 15.06516239 |
| 2921 | Portugal | 2014 | 82.05420714 | 0.723021282 | 15.06516239 |
| 2922 | Portugal | 2015 | 82.54761523 | 0.72575622 | 14.77524801 |
| 2923 | Portugal | 2017 | 83.22487148 | 0.731225535 | 14.22935117 |
| 2924 | Portugal | 2020 | 84.42497961 | 0.741037384 | 13.44730416 |
| 2925 | Portugal | 2021 | 74.26667853 | 0.744151851 | 13.44730416 |
| 2926 | Puerto Rico | 1991 | 144.7936693 | 0.66326172 | 22.46369183 |
| 2927 | Puerto Rico | 1994 | 146.2098946 | 0.67545081 | 22.03195463 |
| 2928 | Puerto Rico | 1995 | 146.8617024 | 0.68067395 | 17.2540694 |
| 2929 | Puerto Rico | 1997 | 148.9670226 | 0.691274382 | 17.2540694 |
| 2930 | Puerto Rico | 1999 | 152.1455875 | 0.702850671 | 17.2540694 |
| 2931 | Puerto Rico | 2000 | 153.9551647 | 0.710035652 | 17.2540694 |
| 2932 | Puerto Rico | 2002 | 159.3576967 | 0.725956856 | 14.77524801 |
| 2933 | Puerto Rico | 2003 | 162.8552321 | 0.730475138 | 14.22935117 |
| 2934 | Puerto Rico | 2008 | 179.7321159 | 0.750922167 | 13.44730416 |
| 2935 | Puerto Rico | 2010 | 186.7940784 | 0.761763253 | 9.92126395 |
| 2936 | Puerto Rico | 2011 | 191.0879843 | 0.766784882 | 9.92126395 |
| 2937 | Puerto Rico | 2013 | 201.2299291 | 0.778461162 | 9.92126395 |
| 2938 | Puerto Rico | 2017 | 210.0425406 | 0.806648961 | 8.445315313 |
| 2939 | Puerto Rico | 2018 | 210.6162203 | 0.812906066 | 8.445315313 |
| 2940 | Puerto Rico | 2020 | 211.182995 | 0.822918436 | 8.445315313 |
| 2941 | Puerto Rico | 2021 | 214.26032 | 0.825525847 | 8.445315313 |
| 2942 | Qatar | 1992 | 137.5262348 | 0.660673763 | 22.46369183 |
| 2943 | Qatar | 1993 | 135.2095033 | 0.666194399 | 22.46369183 |
| 2944 | Qatar | 1996 | 133.0892155 | 0.684835919 | 17.2540694 |
| 2945 | Qatar | 1997 | 133.4848287 | 0.693109654 | 17.2540694 |
| 2946 | Qatar | 1998 | 134.0966183 | 0.701409064 | 17.2540694 |
| 2947 | Qatar | 1999 | 134.7911739 | 0.708880981 | 17.2540694 |
| 2948 | Qatar | 2000 | 135.3892586 | 0.714831609 | 17.2540694 |
| 2949 | Qatar | 2001 | 136.0410118 | 0.720831064 | 15.06516239 |
| 2950 | Qatar | 2002 | 136.8851549 | 0.727112174 | 14.77524801 |
| 2951 | Qatar | 2003 | 137.9293469 | 0.733536599 | 14.22935117 |
| 2952 | Qatar | 2006 | 142.2205523 | 0.754123028 | 10.45018691 |
| 2953 | Qatar | 2011 | 157.9195886 | 0.78721245 | 8.445315313 |
| 2954 | Qatar | 2013 | 165.6913521 | 0.799611555 | 8.445315313 |
| 2955 | Qatar | 2014 | 168.8811682 | 0.80572571 | 8.445315313 |
| 2956 | Qatar | 2016 | 171.2663138 | 0.817979841 | 8.445315313 |
| 2957 | Qatar | 2018 | 171.8990053 | 0.829962127 | 8.445315313 |
| 2958 | Qatar | 2019 | 171.9405333 | 0.835805284 | 8.445315313 |
| 2959 | Qatar | 2021 | 174.51553 | 0.846860584 | 8.445315313 |
| 2960 | Republic of Korea | 1990 | 107.7096685 | 0.692329307 | 17.2540694 |
| 2961 | Republic of Korea | 1993 | 106.0199996 | 0.72228674 | 15.06516239 |
| 2962 | Republic of Korea | 1995 | 104.3434249 | 0.74194003 | 13.44730416 |
| 2963 | Republic of Korea | 1996 | 102.9631833 | 0.75155324 | 13.44730416 |
| 2964 | Republic of Korea | 1997 | 101.0366227 | 0.760591683 | 9.92126395 |
| 2965 | Republic of Korea | 2000 | 96.70752299 | 0.785428821 | 8.445315313 |
| 2966 | Republic of Korea | 2004 | 113.8657535 | 0.813720927 | 8.445315313 |
| 2967 | Republic of Korea | 2005 | 115.8813357 | 0.8190934 | 8.445315313 |
| 2968 | Republic of Korea | 2006 | 114.9002142 | 0.82417982 | 8.445315313 |
| 2969 | Republic of Korea | 2007 | 112.5108656 | 0.829127953 | 8.445315313 |
| 2970 | Republic of Korea | 2008 | 109.596102 | 0.833894627 | 8.445315313 |
| 2971 | Republic of Korea | 2010 | 105.6837166 | 0.842511429 | 8.445315313 |
| 2972 | Republic of Korea | 2011 | 105.2633757 | 0.846913229 | 8.445315313 |
| 2973 | Republic of Korea | 2012 | 104.8536887 | 0.851140263 | 8.445315313 |
| 2974 | Republic of Korea | 2015 | 103.8364762 | 0.863907408 | 8.445315313 |
| 2975 | Republic of Korea | 2016 | 103.4729225 | 0.868317824 | 8.445315313 |
| 2976 | Republic of Korea | 2018 | 102.3535283 | 0.876823317 | 8.445315313 |
| 2977 | Republic of Korea | 2021 | 103.0136374 | 0.886675267 | 8.445315313 |
| 2978 | Republic of Moldova | 1993 | 32.04043631 | 0.61415292 | 32.04043631 |
| 2979 | Republic of Moldova | 1996 | 35.27738986 | 0.623113984 | 32.04043631 |
| 2980 | Republic of Moldova | 1997 | 37.35720712 | 0.624370105 | 32.04043631 |
| 2981 | Republic of Moldova | 1998 | 39.82545945 | 0.624116761 | 32.04043631 |
| 2982 | Republic of Moldova | 2000 | 44.79842583 | 0.622004934 | 32.04043631 |
| 2983 | Republic of Moldova | 2001 | 47.06163586 | 0.62293641 | 32.04043631 |
| 2984 | Republic of Moldova | 2002 | 49.4658946 | 0.625874845 | 32.04043631 |
| 2985 | Republic of Moldova | 2003 | 52.06954773 | 0.629985045 | 22.46369183 |
| 2986 | Republic of Moldova | 2005 | 58.11341667 | 0.641530965 | 22.46369183 |
| 2987 | Republic of Moldova | 2006 | 62.00657373 | 0.647996264 | 22.46369183 |
| 2988 | Republic of Moldova | 2007 | 66.7621708 | 0.654215851 | 22.46369183 |
| 2989 | Republic of Moldova | 2011 | 88.71692276 | 0.678004137 | 22.03195463 |
| 2990 | Republic of Moldova | 2012 | 94.79030102 | 0.682979796 | 17.2540694 |
| 2991 | Republic of Moldova | 2013 | 100.9325779 | 0.688519175 | 17.2540694 |
| 2992 | Republic of Moldova | 2014 | 106.7994053 | 0.694104557 | 17.2540694 |
| 2993 | Republic of Moldova | 2015 | 112.0414453 | 0.699355166 | 17.2540694 |
| 2994 | Republic of Moldova | 2016 | 117.266473 | 0.705144542 | 17.2540694 |
| 2995 | Republic of Moldova | 2017 | 122.5645805 | 0.711206637 | 17.2540694 |
| 2996 | Republic of Moldova | 2018 | 126.7933781 | 0.717244427 | 17.2540694 |
| 2997 | Republic of Moldova | 2019 | 128.8314574 | 0.723011648 | 15.06516239 |
| 2998 | Republic of Moldova | 2020 | 127.2338717 | 0.72771054 | 14.22935117 |
| 2999 | Republic of Moldova | 2021 | 97.82376641 | 0.732214875 | 14.22935117 |
| 3000 | Romania | 1991 | 113.5568164 | 0.626795627 | 32.04043631 |
| 3001 | Romania | 1998 | 116.6263524 | 0.654927769 | 22.46369183 |
| 3002 | Romania | 2000 | 121.3353099 | 0.665294346 | 22.46369183 |
| 3003 | Romania | 2001 | 124.863274 | 0.672020167 | 22.03195463 |
| 3004 | Romania | 2002 | 130.3608003 | 0.678297417 | 22.03195463 |
| 3005 | Romania | 2006 | 150.180608 | 0.699490766 | 17.2540694 |
| 3006 | Romania | 2008 | 156.5324583 | 0.710757128 | 17.2540694 |
| 3007 | Romania | 2009 | 159.0872502 | 0.717445122 | 17.2540694 |
| 3008 | Romania | 2010 | 160.9129381 | 0.723891349 | 15.06516239 |
| 3009 | Romania | 2012 | 163.4015108 | 0.733892482 | 14.22935117 |
| 3010 | Romania | 2014 | 165.5062965 | 0.740395467 | 13.44730416 |
| 3011 | Romania | 2015 | 166.507215 | 0.74347353 | 13.44730416 |
| 3012 | Romania | 2017 | 169.6689935 | 0.751117367 | 13.44730416 |
| 3013 | Romania | 2018 | 171.3261681 | 0.755533333 | 10.45018691 |
| 3014 | Romania | 2020 | 151.1811139 | 0.764276444 | 9.92126395 |
| 3015 | Russian Federation | 1990 | 27.63768735 | 0.671600578 | 22.03195463 |
| 3016 | Russian Federation | 1992 | 27.67917099 | 0.688068022 | 17.2540694 |
| 3017 | Russian Federation | 1994 | 28.19840797 | 0.694144966 | 17.2540694 |
| 3018 | Russian Federation | 1997 | 30.78339739 | 0.703752867 | 17.2540694 |
| 3019 | Russian Federation | 1998 | 32.51859391 | 0.705198518 | 17.2540694 |
| 3020 | Russian Federation | 2000 | 35.68379724 | 0.708643648 | 17.2540694 |
| 3021 | Russian Federation | 2001 | 36.82904973 | 0.710570359 | 17.2540694 |
| 3022 | Russian Federation | 2002 | 37.94182396 | 0.713460957 | 17.2540694 |
| 3023 | Russian Federation | 2004 | 39.93705602 | 0.726305319 | 14.77524801 |
| 3024 | Russian Federation | 2005 | 40.7355316 | 0.733592777 | 14.22935117 |
| 3025 | Russian Federation | 2006 | 41.48063945 | 0.740064664 | 13.44730416 |
| 3026 | Russian Federation | 2007 | 42.26184225 | 0.746191512 | 13.44730416 |
| 3027 | Russian Federation | 2008 | 43.01559319 | 0.752623243 | 13.44730416 |
| 3028 | Russian Federation | 2010 | 44.11152142 | 0.763320116 | 9.92126395 |
| 3029 | Russian Federation | 2011 | 44.29600373 | 0.767633588 | 9.92126395 |
| 3030 | Russian Federation | 2013 | 44.33549804 | 0.776526979 | 9.92126395 |
| 3031 | Russian Federation | 2016 | 44.03099506 | 0.790330149 | 8.445315313 |
| 3032 | Russian Federation | 2017 | 43.79813791 | 0.795669484 | 8.445315313 |
| 3033 | Russian Federation | 2019 | 43.36110738 | 0.803658201 | 8.445315313 |
| 3034 | Russian Federation | 2020 | 42.75615494 | 0.806011474 | 8.445315313 |
| 3035 | Russian Federation | 2021 | 42.6503026 | 0.808536005 | 8.445315313 |
| 3036 | Rwanda | 1990 | 280.6847887 | 0.27509719 | 100.8275782 |
| 3037 | Rwanda | 1991 | 280.1405282 | 0.276194698 | 100.8275782 |
| 3038 | Rwanda | 1993 | 279.3479361 | 0.276980133 | 100.8275782 |
| 3039 | Rwanda | 1997 | 278.7133475 | 0.272455861 | 100.8275782 |
| 3040 | Rwanda | 1998 | 278.9118207 | 0.274582551 | 100.8275782 |
| 3041 | Rwanda | 1999 | 279.1172296 | 0.277014387 | 100.8275782 |
| 3042 | Rwanda | 2003 | 282.013209 | 0.299676918 | 100.8275782 |
| 3043 | Rwanda | 2004 | 283.1878566 | 0.306943916 | 89.96318282 |
| 3044 | Rwanda | 2005 | 284.0236873 | 0.314219578 | 67.91401256 |
| 3045 | Rwanda | 2006 | 284.7911908 | 0.321998519 | 66.46502371 |
| 3046 | Rwanda | 2007 | 285.765398 | 0.329616591 | 65.82455226 |
| 3047 | Rwanda | 2010 | 288.3507628 | 0.351659416 | 65.82455226 |
| 3048 | Rwanda | 2011 | 289.1016533 | 0.35894034 | 64.06460795 |
| 3049 | Rwanda | 2012 | 290.1900732 | 0.366411821 | 64.06460795 |
| 3050 | Rwanda | 2013 | 291.3672195 | 0.373769682 | 64.06460795 |
| 3051 | Rwanda | 2014 | 292.3358269 | 0.381533179 | 63.35324861 |
| 3052 | Rwanda | 2016 | 292.8788499 | 0.398287959 | 62.92622144 |
| 3053 | Rwanda | 2019 | 292.7818883 | 0.423086727 | 62.92622144 |
| 3054 | Rwanda | 2020 | 294.368268 | 0.429896596 | 62.92622144 |
| 3055 | Saint Kitts and Nevis | 1990 | 144.0343641 | 0.580685877 | 51.75814966 |
| 3056 | Saint Kitts and Nevis | 1994 | 142.6021115 | 0.614514658 | 32.04043631 |
| 3057 | Saint Kitts and Nevis | 1996 | 143.1429895 | 0.626207411 | 32.04043631 |
| 3058 | Saint Kitts and Nevis | 2001 | 151.5390972 | 0.649226653 | 22.46369183 |
| 3059 | Saint Kitts and Nevis | 2002 | 154.4326591 | 0.654593409 | 22.46369183 |
| 3060 | Saint Kitts and Nevis | 2004 | 161.4656018 | 0.66756813 | 22.46369183 |
| 3061 | Saint Kitts and Nevis | 2005 | 165.1993855 | 0.679145457 | 22.03195463 |
| 3062 | Saint Kitts and Nevis | 2006 | 169.4396538 | 0.690078667 | 17.2540694 |
| 3063 | Saint Kitts and Nevis | 2007 | 174.4554376 | 0.697727628 | 17.2540694 |
| 3064 | Saint Kitts and Nevis | 2008 | 179.7929162 | 0.704141494 | 17.2540694 |
| 3065 | Saint Kitts and Nevis | 2011 | 194.1646839 | 0.719324381 | 15.06516239 |
| 3066 | Saint Kitts and Nevis | 2012 | 198.8604384 | 0.723436152 | 15.06516239 |
| 3067 | Saint Kitts and Nevis | 2013 | 203.2484944 | 0.726706593 | 14.77524801 |
| 3068 | Saint Kitts and Nevis | 2015 | 209.0360326 | 0.732220672 | 14.22935117 |
| 3069 | Saint Kitts and Nevis | 2016 | 210.096648 | 0.735566138 | 13.44730416 |
| 3070 | Saint Kitts and Nevis | 2017 | 210.7364134 | 0.739470681 | 13.44730416 |
| 3071 | Saint Kitts and Nevis | 2018 | 211.1614497 | 0.743736461 | 13.44730416 |
| 3072 | Saint Kitts and Nevis | 2019 | 211.5606787 | 0.748152066 | 13.44730416 |
| 3073 | Saint Kitts and Nevis | 2020 | 212.6811324 | 0.751568409 | 13.44730416 |
| 3074 | Saint Kitts and Nevis | 2021 | 206.6157416 | 0.754987055 | 10.45018691 |
| 3075 | Saint Lucia | 1990 | 224.6618008 | 0.49629657 | 53.58976548 |
| 3076 | Saint Lucia | 1991 | 225.7186225 | 0.505975555 | 53.58976548 |
| 3077 | Saint Lucia | 1992 | 226.6190113 | 0.515368261 | 53.58976548 |
| 3078 | Saint Lucia | 1993 | 227.3488804 | 0.524234949 | 53.58976548 |
| 3079 | Saint Lucia | 1994 | 227.8702391 | 0.532731083 | 53.31984512 |
| 3080 | Saint Lucia | 1995 | 228.1401847 | 0.541479794 | 53.31984512 |
| 3081 | Saint Lucia | 1999 | 227.3105295 | 0.573428555 | 51.75814966 |
| 3082 | Saint Lucia | 2001 | 228.5893025 | 0.585087558 | 51.75814966 |
| 3083 | Saint Lucia | 2002 | 231.0605085 | 0.590167578 | 51.75814966 |
| 3084 | Saint Lucia | 2006 | 242.8718242 | 0.6127553 | 51.75814966 |
| 3085 | Saint Lucia | 2007 | 245.6480872 | 0.617994714 | 32.04043631 |
| 3086 | Saint Lucia | 2008 | 248.6012696 | 0.623273001 | 32.04043631 |
| 3087 | Saint Lucia | 2009 | 251.6479171 | 0.628169849 | 22.46369183 |
| 3088 | Saint Lucia | 2010 | 254.7024338 | 0.632782905 | 22.46369183 |
| 3089 | Saint Lucia | 2011 | 258.2911272 | 0.637439442 | 22.46369183 |
| 3090 | Saint Lucia | 2012 | 262.5242049 | 0.641567127 | 22.46369183 |
| 3091 | Saint Lucia | 2013 | 266.7486061 | 0.645153079 | 22.46369183 |
| 3092 | Saint Lucia | 2014 | 270.3110301 | 0.648743507 | 22.46369183 |
| 3093 | Saint Lucia | 2015 | 272.544747 | 0.65215748 | 22.46369183 |
| 3094 | Saint Lucia | 2016 | 273.8005927 | 0.65565334 | 22.46369183 |
| 3095 | Saint Lucia | 2017 | 274.8259133 | 0.659341346 | 22.46369183 |
| 3096 | Saint Lucia | 2019 | 276.4752236 | 0.666840359 | 22.46369183 |
| 3097 | Saint Lucia | 2020 | 277.8786272 | 0.66972049 | 22.46369183 |
| 3098 | Saint Vincent and the Grenadines | 1990 | 248.603117 | 0.475930186 | 53.58976548 |
| 3099 | Saint Vincent and the Grenadines | 1991 | 247.8279597 | 0.481075658 | 53.58976548 |
| 3100 | Saint Vincent and the Grenadines | 1992 | 247.3218801 | 0.485977871 | 53.58976548 |
| 3101 | Saint Vincent and the Grenadines | 1993 | 247.0514984 | 0.490998791 | 53.58976548 |
| 3102 | Saint Vincent and the Grenadines | 1994 | 246.9925758 | 0.496379132 | 53.58976548 |
| 3103 | Saint Vincent and the Grenadines | 1997 | 249.0488063 | 0.515482619 | 53.58976548 |
| 3104 | Saint Vincent and the Grenadines | 1998 | 250.8062104 | 0.521209679 | 53.58976548 |
| 3105 | Saint Vincent and the Grenadines | 1999 | 252.5550579 | 0.527099552 | 53.58976548 |
| 3106 | Saint Vincent and the Grenadines | 2000 | 253.8878432 | 0.53317329 | 53.31984512 |
| 3107 | Saint Vincent and the Grenadines | 2001 | 254.9390859 | 0.539455712 | 53.31984512 |
| 3108 | Saint Vincent and the Grenadines | 2002 | 256.1361484 | 0.546063616 | 51.75814966 |
| 3109 | Saint Vincent and the Grenadines | 2003 | 257.4877427 | 0.552693724 | 51.75814966 |
| 3110 | Saint Vincent and the Grenadines | 2006 | 263.5016129 | 0.568221748 | 51.75814966 |
| 3111 | Saint Vincent and the Grenadines | 2008 | 272.5144989 | 0.577611145 | 51.75814966 |
| 3112 | Saint Vincent and the Grenadines | 2009 | 277.5908052 | 0.582549107 | 51.75814966 |
| 3113 | Saint Vincent and the Grenadines | 2010 | 282.1971395 | 0.587190384 | 51.75814966 |
| 3114 | Saint Vincent and the Grenadines | 2011 | 286.4859263 | 0.590259096 | 51.75814966 |
| 3115 | Saint Vincent and the Grenadines | 2015 | 303.222858 | 0.603754135 | 51.75814966 |
| 3116 | Saint Vincent and the Grenadines | 2016 | 307.4015491 | 0.61005921 | 51.75814966 |
| 3117 | Saint Vincent and the Grenadines | 2017 | 311.8966729 | 0.616438473 | 32.04043631 |
| 3118 | Saint Vincent and the Grenadines | 2018 | 315.8348948 | 0.622634944 | 32.04043631 |
| 3119 | Saint Vincent and the Grenadines | 2020 | 318.3272053 | 0.632860904 | 22.46369183 |
| 3120 | Saint Vincent and the Grenadines | 2021 | 310.1537629 | 0.637195963 | 22.46369183 |
| 3121 | Samoa | 1991 | 148.6401755 | 0.490987146 | 53.58976548 |
| 3122 | Samoa | 1992 | 147.6466483 | 0.494027448 | 53.58976548 |
| 3123 | Samoa | 1995 | 144.7214536 | 0.502369735 | 53.58976548 |
| 3124 | Samoa | 1996 | 143.7126231 | 0.505778026 | 53.58976548 |
| 3125 | Samoa | 1998 | 141.4227015 | 0.512367566 | 53.58976548 |
| 3126 | Samoa | 1999 | 140.3095015 | 0.51540905 | 53.58976548 |
| 3127 | Samoa | 2000 | 139.3295166 | 0.518902327 | 53.58976548 |
| 3128 | Samoa | 2002 | 137.4892124 | 0.52724267 | 53.58976548 |
| 3129 | Samoa | 2003 | 136.5663023 | 0.531603736 | 53.58976548 |
| 3130 | Samoa | 2005 | 134.8698309 | 0.540557908 | 53.31984512 |
| 3131 | Samoa | 2006 | 134.0272245 | 0.544812068 | 51.75814966 |
| 3132 | Samoa | 2007 | 133.0985316 | 0.549110752 | 51.75814966 |
| 3133 | Samoa | 2008 | 132.1796801 | 0.553164596 | 51.75814966 |
| 3134 | Samoa | 2009 | 131.3672254 | 0.556474015 | 51.75814966 |
| 3135 | Samoa | 2010 | 130.7530307 | 0.559622905 | 51.75814966 |
| 3136 | Samoa | 2011 | 130.2210398 | 0.562891574 | 51.75814966 |
| 3137 | Samoa | 2015 | 128.6315914 | 0.571998983 | 51.75814966 |
| 3138 | Samoa | 2017 | 129.5447133 | 0.578925435 | 51.75814966 |
| 3139 | Samoa | 2019 | 130.6953553 | 0.586256695 | 51.75814966 |
| 3140 | Samoa | 2021 | 132.5659376 | 0.593392769 | 51.75814966 |
| 3141 | San Marino | 1994 | 74.34907699 | 0.831970983 | 8.445315313 |
| 3142 | San Marino | 1996 | 75.55794321 | 0.841389495 | 8.445315313 |
| 3143 | San Marino | 1997 | 76.56057198 | 0.845864865 | 8.445315313 |
| 3144 | San Marino | 1999 | 79.13179051 | 0.855338613 | 8.445315313 |
| 3145 | San Marino | 2000 | 80.53440092 | 0.859695363 | 8.445315313 |
| 3146 | San Marino | 2002 | 83.99511441 | 0.865680592 | 8.445315313 |
| 3147 | San Marino | 2003 | 86.04000739 | 0.867522009 | 8.445315313 |
| 3148 | San Marino | 2004 | 88.13437676 | 0.869287134 | 8.445315313 |
| 3149 | San Marino | 2006 | 92.30826822 | 0.87265215 | 8.445315313 |
| 3150 | San Marino | 2007 | 94.66547489 | 0.874307253 | 8.445315313 |
| 3151 | San Marino | 2008 | 96.98823396 | 0.875991881 | 8.445315313 |
| 3152 | San Marino | 2009 | 99.02883998 | 0.877705228 | 8.445315313 |
| 3153 | San Marino | 2010 | 100.5337861 | 0.879440814 | 8.445315313 |
| 3154 | San Marino | 2013 | 103.545396 | 0.884592627 | 8.445315313 |
| 3155 | San Marino | 2016 | 105.0431867 | 0.884477665 | 8.445315313 |
| 3156 | San Marino | 2017 | 105.3465845 | 0.884557607 | 8.445315313 |
| 3157 | San Marino | 2018 | 105.6140456 | 0.885133516 | 8.445315313 |
| 3158 | San Marino | 2021 | 96.90301727 | 0.888005474 | 8.445315313 |
| 3159 | Sao Tome and Principe | 1990 | 277.244481 | 0.309542852 | 89.96318282 |
| 3160 | Sao Tome and Principe | 1991 | 276.9496135 | 0.310601028 | 89.96318282 |
| 3161 | Sao Tome and Principe | 1994 | 276.8061112 | 0.314261087 | 67.91401256 |
| 3162 | Sao Tome and Principe | 1995 | 276.9754396 | 0.31584392 | 67.17014806 |
| 3163 | Sao Tome and Principe | 1998 | 275.8740512 | 0.322519219 | 66.46502371 |
| 3164 | Sao Tome and Principe | 1999 | 275.4234221 | 0.325651916 | 66.46502371 |
| 3165 | Sao Tome and Principe | 2000 | 275.3240222 | 0.329460109 | 65.82455226 |
| 3166 | Sao Tome and Principe | 2002 | 275.8111558 | 0.340302837 | 65.82455226 |
| 3167 | Sao Tome and Principe | 2003 | 276.159705 | 0.346851981 | 65.82455226 |
| 3168 | Sao Tome and Principe | 2004 | 276.5263473 | 0.353722384 | 65.82455226 |
| 3169 | Sao Tome and Principe | 2005 | 276.8716692 | 0.361105881 | 64.06460795 |
| 3170 | Sao Tome and Principe | 2006 | 277.4469646 | 0.369413007 | 64.06460795 |
| 3171 | Sao Tome and Principe | 2007 | 278.4100249 | 0.377813517 | 63.35324861 |
| 3172 | Sao Tome and Principe | 2008 | 279.519544 | 0.386688944 | 63.35324861 |
| 3173 | Sao Tome and Principe | 2010 | 281.2401802 | 0.405530666 | 62.92622144 |
| 3174 | Sao Tome and Principe | 2011 | 281.7164288 | 0.415783728 | 62.92622144 |
| 3175 | Sao Tome and Principe | 2015 | 283.8570594 | 0.454051473 | 62.92622144 |
| 3176 | Sao Tome and Principe | 2016 | 284.6625226 | 0.463230082 | 62.92622144 |
| 3177 | Sao Tome and Principe | 2017 | 285.858565 | 0.47217093 | 53.58976548 |
| 3178 | Sao Tome and Principe | 2019 | 288.0711029 | 0.48983823 | 53.58976548 |
| 3179 | Sao Tome and Principe | 2020 | 288.8225589 | 0.497861409 | 53.58976548 |
| 3180 | Sao Tome and Principe | 2021 | 290.0823811 | 0.505413747 | 53.58976548 |
| 3181 | Saudi Arabia | 1991 | 279.0438265 | 0.549907717 | 51.75814966 |
| 3182 | Saudi Arabia | 1993 | 277.6835386 | 0.572186913 | 51.75814966 |
| 3183 | Saudi Arabia | 1994 | 277.2303071 | 0.582878871 | 51.75814966 |
| 3184 | Saudi Arabia | 1996 | 276.9998725 | 0.603648418 | 51.75814966 |
| 3185 | Saudi Arabia | 1997 | 277.4430669 | 0.613749116 | 51.75814966 |
| 3186 | Saudi Arabia | 2000 | 278.7346953 | 0.642311774 | 22.46369183 |
| 3187 | Saudi Arabia | 2001 | 278.3388944 | 0.651502092 | 22.46369183 |
| 3188 | Saudi Arabia | 2002 | 277.668723 | 0.660356856 | 22.46369183 |
| 3189 | Saudi Arabia | 2003 | 276.9271963 | 0.669839577 | 22.46369183 |
| 3190 | Saudi Arabia | 2004 | 276.327149 | 0.679917856 | 22.03195463 |
| 3191 | Saudi Arabia | 2005 | 276.1296787 | 0.690520852 | 17.2540694 |
| 3192 | Saudi Arabia | 2006 | 276.2076637 | 0.701152613 | 17.2540694 |
| 3193 | Saudi Arabia | 2007 | 276.4821717 | 0.711442141 | 17.2540694 |
| 3194 | Saudi Arabia | 2008 | 276.9211625 | 0.721960205 | 15.06516239 |
| 3195 | Saudi Arabia | 2010 | 278.1652902 | 0.740300669 | 13.44730416 |
| 3196 | Saudi Arabia | 2011 | 279.2743087 | 0.749959451 | 13.44730416 |
| 3197 | Saudi Arabia | 2014 | 285.2355651 | 0.775178133 | 9.92126395 |
| 3198 | Saudi Arabia | 2015 | 286.4409867 | 0.782300802 | 9.92126395 |
| 3199 | Saudi Arabia | 2017 | 286.751558 | 0.794886288 | 8.445315313 |
| 3200 | Saudi Arabia | 2018 | 286.7566564 | 0.800656985 | 8.445315313 |
| 3201 | Saudi Arabia | 2019 | 286.9143018 | 0.806087841 | 8.445315313 |
| 3202 | Saudi Arabia | 2021 | 289.0682546 | 0.815143493 | 8.445315313 |
| 3203 | Senegal | 1990 | 253.1856792 | 0.238047613 | 101.1599627 |
| 3204 | Senegal | 1992 | 253.3272556 | 0.250416532 | 100.8275782 |
| 3205 | Senegal | 1993 | 253.5240767 | 0.255767964 | 100.8275782 |
| 3206 | Senegal | 1995 | 253.9922143 | 0.264438773 | 100.8275782 |
| 3207 | Senegal | 1996 | 254.9451144 | 0.268318965 | 100.8275782 |
| 3208 | Senegal | 1997 | 256.831958 | 0.272072875 | 100.8275782 |
| 3209 | Senegal | 1998 | 259.0330021 | 0.275995656 | 100.8275782 |
| 3210 | Senegal | 1999 | 260.8990447 | 0.280111918 | 100.8275782 |
| 3211 | Senegal | 2001 | 261.3606573 | 0.287451899 | 100.8275782 |
| 3212 | Senegal | 2002 | 260.5424459 | 0.291046288 | 100.8275782 |
| 3213 | Senegal | 2003 | 259.5093486 | 0.294887172 | 100.8275782 |
| 3214 | Senegal | 2007 | 254.6501079 | 0.312570298 | 67.91401256 |
| 3215 | Senegal | 2009 | 251.0433864 | 0.323657726 | 66.46502371 |
| 3216 | Senegal | 2013 | 254.2813653 | 0.350134004 | 65.82455226 |
| 3217 | Senegal | 2019 | 260.6826059 | 0.394012316 | 62.92622144 |
| 3218 | Senegal | 2021 | 263.3819169 | 0.408054193 | 62.92622144 |
| 3219 | Serbia | 1990 | 80.62190758 | 0.63051102 | 22.46369183 |
| 3220 | Serbia | 1991 | 80.43926562 | 0.638016934 | 22.46369183 |
| 3221 | Serbia | 1992 | 80.27662655 | 0.641971994 | 22.46369183 |
| 3222 | Serbia | 1993 | 80.1532465 | 0.642096034 | 22.46369183 |
| 3223 | Serbia | 1994 | 80.10043261 | 0.642223221 | 22.46369183 |
| 3224 | Serbia | 1997 | 80.4946102 | 0.647162094 | 22.46369183 |
| 3225 | Serbia | 1998 | 80.73639156 | 0.650245989 | 22.46369183 |
| 3226 | Serbia | 1999 | 80.90425136 | 0.655534174 | 22.46369183 |
| 3227 | Serbia | 2000 | 80.9004447 | 0.6607528 | 22.46369183 |
| 3228 | Serbia | 2002 | 73.54832897 | 0.671511072 | 22.03195463 |
| 3229 | Serbia | 2004 | 62.33283274 | 0.686819842 | 17.2540694 |
| 3230 | Serbia | 2008 | 61.19934281 | 0.721505306 | 15.06516239 |
| 3231 | Serbia | 2010 | 62.40624521 | 0.740091311 | 13.44730416 |
| 3232 | Serbia | 2011 | 63.07774508 | 0.750072762 | 13.44730416 |
| 3233 | Serbia | 2013 | 65.02774754 | 0.762170908 | 9.92126395 |
| 3234 | Serbia | 2014 | 66.10703877 | 0.766043091 | 9.92126395 |
| 3235 | Serbia | 2019 | 71.66080266 | 0.7857683 | 8.445315313 |
| 3236 | Serbia | 2020 | 92.47963556 | 0.789183333 | 8.445315313 |
| 3237 | Serbia | 2021 | 89.38555808 | 0.792416294 | 8.445315313 |
| 3238 | Seychelles | 1993 | 284.8020087 | 0.598044194 | 51.75814966 |
| 3239 | Seychelles | 1994 | 285.4310651 | 0.606041268 | 51.75814966 |
| 3240 | Seychelles | 1995 | 285.8512275 | 0.613853281 | 51.75814966 |
| 3241 | Seychelles | 1996 | 286.0916225 | 0.62213517 | 32.04043631 |
| 3242 | Seychelles | 1998 | 286.2388102 | 0.638438882 | 22.46369183 |
| 3243 | Seychelles | 2000 | 286.1878418 | 0.651551988 | 22.46369183 |
| 3244 | Seychelles | 2001 | 286.1974131 | 0.656490668 | 22.46369183 |
| 3245 | Seychelles | 2002 | 286.2792392 | 0.66090779 | 22.46369183 |
| 3246 | Seychelles | 2008 | 287.226587 | 0.676590946 | 22.03195463 |
| 3247 | Seychelles | 2009 | 287.4777561 | 0.677847028 | 22.03195463 |
| 3248 | Seychelles | 2010 | 287.6476488 | 0.68016364 | 22.03195463 |
| 3249 | Seychelles | 2011 | 287.9458307 | 0.683681491 | 17.2540694 |
| 3250 | Seychelles | 2012 | 288.5858509 | 0.687629394 | 17.2540694 |
| 3251 | Seychelles | 2013 | 289.4169294 | 0.692525734 | 17.2540694 |
| 3252 | Seychelles | 2015 | 290.6655198 | 0.70172282 | 17.2540694 |
| 3253 | Seychelles | 2016 | 291.2211097 | 0.706183623 | 17.2540694 |
| 3254 | Seychelles | 2018 | 293.0336168 | 0.716407819 | 17.2540694 |
| 3255 | Seychelles | 2019 | 293.5656509 | 0.722073685 | 15.06516239 |
| 3256 | Seychelles | 2021 | 282.9494094 | 0.730150775 | 14.22935117 |
| 3257 | Sierra Leone | 1995 | 248.090277 | 0.219822604 | 101.1599627 |
| 3258 | Sierra Leone | 1997 | 248.2065268 | 0.219089791 | 101.1599627 |
| 3259 | Sierra Leone | 1999 | 248.171951 | 0.217943042 | 101.1599627 |
| 3260 | Sierra Leone | 2005 | 252.1468211 | 0.23954994 | 101.1599627 |
| 3261 | Sierra Leone | 2006 | 252.7451123 | 0.245647886 | 100.8275782 |
| 3262 | Sierra Leone | 2007 | 253.4961968 | 0.252523077 | 100.8275782 |
| 3263 | Sierra Leone | 2008 | 254.3098069 | 0.259781562 | 100.8275782 |
| 3264 | Sierra Leone | 2010 | 255.736069 | 0.274270275 | 100.8275782 |
| 3265 | Sierra Leone | 2011 | 256.5775479 | 0.281673575 | 100.8275782 |
| 3266 | Sierra Leone | 2012 | 257.6816807 | 0.290338512 | 100.8275782 |
| 3267 | Sierra Leone | 2015 | 260.5107597 | 0.31699865 | 67.17014806 |
| 3268 | Sierra Leone | 2016 | 260.9327258 | 0.324091565 | 66.46502371 |
| 3269 | Sierra Leone | 2019 | 261.9550218 | 0.345770019 | 65.82455226 |
| 3270 | Singapore | 1990 | 78.36906592 | 0.686404444 | 17.2540694 |
| 3271 | Singapore | 1991 | 78.59174534 | 0.695055558 | 17.2540694 |
| 3272 | Singapore | 1992 | 78.84092664 | 0.703427834 | 17.2540694 |
| 3273 | Singapore | 1993 | 79.14090192 | 0.712315204 | 17.2540694 |
| 3274 | Singapore | 1995 | 79.83835329 | 0.729982231 | 14.22935117 |
| 3275 | Singapore | 1997 | 80.61603773 | 0.747571898 | 13.44730416 |
| 3276 | Singapore | 1999 | 81.57366669 | 0.760831136 | 9.92126395 |
| 3277 | Singapore | 2000 | 82.24842168 | 0.76771737 | 9.92126395 |
| 3278 | Singapore | 2002 | 84.17692951 | 0.77973577 | 9.92126395 |
| 3279 | Singapore | 2003 | 85.44434809 | 0.784165555 | 8.445315313 |
| 3280 | Singapore | 2005 | 88.7266558 | 0.796455751 | 8.445315313 |
| 3281 | Singapore | 2007 | 95.01265281 | 0.810625304 | 8.445315313 |
| 3282 | Singapore | 2008 | 99.11134637 | 0.818124162 | 8.445315313 |
| 3283 | Singapore | 2010 | 105.4871206 | 0.83022216 | 8.445315313 |
| 3284 | Singapore | 2012 | 108.9568521 | 0.837391807 | 8.445315313 |
| 3285 | Singapore | 2013 | 110.5083081 | 0.840420178 | 8.445315313 |
| 3286 | Singapore | 2014 | 111.9994322 | 0.842894538 | 8.445315313 |
| 3287 | Singapore | 2016 | 115.6192379 | 0.847596999 | 8.445315313 |
| 3288 | Singapore | 2017 | 118.089129 | 0.849051518 | 8.445315313 |
| 3289 | Singapore | 2018 | 120.2496881 | 0.850762729 | 8.445315313 |
| 3290 | Singapore | 2019 | 121.3343168 | 0.852497338 | 8.445315313 |
| 3291 | Singapore | 2020 | 120.422376 | 0.85429601 | 8.445315313 |
| 3292 | Slovakia | 1990 | 61.48989132 | 0.653853505 | 22.46369183 |
| 3293 | Slovakia | 1991 | 61.4720001 | 0.658731402 | 22.46369183 |
| 3294 | Slovakia | 1992 | 61.80063168 | 0.665830111 | 22.46369183 |
| 3295 | Slovakia | 1994 | 63.36936331 | 0.688136477 | 17.2540694 |
| 3296 | Slovakia | 1995 | 64.55505708 | 0.697476536 | 17.2540694 |
| 3297 | Slovakia | 1996 | 66.28878064 | 0.704405003 | 17.2540694 |
| 3298 | Slovakia | 1997 | 68.6910715 | 0.71151321 | 17.2540694 |
| 3299 | Slovakia | 2000 | 77.35391271 | 0.73404624 | 14.22935117 |
| 3300 | Slovakia | 2002 | 83.55773673 | 0.746791142 | 13.44730416 |
| 3301 | Slovakia | 2003 | 86.94471968 | 0.75116727 | 13.44730416 |
| 3302 | Slovakia | 2004 | 90.29289943 | 0.755836694 | 10.45018691 |
| 3303 | Slovakia | 2007 | 100.4531625 | 0.772914153 | 9.92126395 |
| 3304 | Slovakia | 2008 | 103.9477692 | 0.7771974 | 9.92126395 |
| 3305 | Slovakia | 2010 | 108.3591321 | 0.786254422 | 8.445315313 |
| 3306 | Slovakia | 2013 | 109.7878085 | 0.797903723 | 8.445315313 |
| 3307 | Slovakia | 2015 | 110.2501811 | 0.80039418 | 8.445315313 |
| 3308 | Slovakia | 2016 | 110.8614448 | 0.80087821 | 8.445315313 |
| 3309 | Slovakia | 2018 | 112.817207 | 0.804357302 | 8.445315313 |
| 3310 | Slovakia | 2020 | 96.18867922 | 0.808305966 | 8.445315313 |
| 3311 | Slovakia | 2021 | 96.90009228 | 0.81061053 | 8.445315313 |
| 3312 | Slovenia | 1991 | 85.1541728 | 0.732666986 | 14.22935117 |
| 3313 | Slovenia | 1992 | 86.73694252 | 0.737391552 | 13.44730416 |
| 3314 | Slovenia | 1994 | 89.95587064 | 0.746234861 | 13.44730416 |
| 3315 | Slovenia | 1995 | 91.52035897 | 0.750994975 | 13.44730416 |
| 3316 | Slovenia | 1996 | 93.03297428 | 0.756348987 | 10.45018691 |
| 3317 | Slovenia | 1997 | 94.62804866 | 0.762275292 | 9.92126395 |
| 3318 | Slovenia | 1998 | 96.39390388 | 0.767728526 | 9.92126395 |
| 3319 | Slovenia | 1999 | 98.45516188 | 0.773236192 | 9.92126395 |
| 3320 | Slovenia | 2000 | 100.94543 | 0.77957801 | 9.92126395 |
| 3321 | Slovenia | 2001 | 104.3583172 | 0.786240776 | 8.445315313 |
| 3322 | Slovenia | 2003 | 113.9507336 | 0.796371531 | 8.445315313 |
| 3323 | Slovenia | 2004 | 119.1458468 | 0.80096224 | 8.445315313 |
| 3324 | Slovenia | 2005 | 123.9835426 | 0.805601248 | 8.445315313 |
| 3325 | Slovenia | 2006 | 128.7781707 | 0.809708595 | 8.445315313 |
| 3326 | Slovenia | 2007 | 133.9564785 | 0.813006319 | 8.445315313 |
| 3327 | Slovenia | 2010 | 147.3209923 | 0.821178486 | 8.445315313 |
| 3328 | Slovenia | 2012 | 154.0832506 | 0.824336563 | 8.445315313 |
| 3329 | Slovenia | 2013 | 157.4099338 | 0.825826275 | 8.445315313 |
| 3330 | Slovenia | 2014 | 160.2820282 | 0.827799246 | 8.445315313 |
| 3331 | Slovenia | 2015 | 162.3908894 | 0.829837071 | 8.445315313 |
| 3332 | Slovenia | 2016 | 164.0933317 | 0.831367915 | 8.445315313 |
| 3333 | Slovenia | 2017 | 165.8004084 | 0.833038139 | 8.445315313 |
| 3334 | Slovenia | 2018 | 167.1456516 | 0.835472481 | 8.445315313 |
| 3335 | Slovenia | 2019 | 167.8557181 | 0.838266782 | 8.445315313 |
| 3336 | Slovenia | 2021 | 148.2529029 | 0.842430731 | 8.445315313 |
| 3337 | Solomon Islands | 1990 | 89.96318282 | 0.301217167 | 89.96318282 |
| 3338 | Solomon Islands | 1991 | 90.14424898 | 0.305830668 | 89.96318282 |
| 3339 | Solomon Islands | 1994 | 90.79138814 | 0.323300247 | 66.46502371 |
| 3340 | Solomon Islands | 1997 | 91.73220058 | 0.341835627 | 65.82455226 |
| 3341 | Solomon Islands | 1998 | 92.14527668 | 0.34713506 | 65.82455226 |
| 3342 | Solomon Islands | 2000 | 92.6504236 | 0.354799143 | 65.82455226 |
| 3343 | Solomon Islands | 2002 | 92.69596401 | 0.357910116 | 64.06460795 |
| 3344 | Solomon Islands | 2003 | 92.69270047 | 0.359424359 | 64.06460795 |
| 3345 | Solomon Islands | 2004 | 92.68221044 | 0.361290558 | 64.06460795 |
| 3346 | Solomon Islands | 2005 | 92.67802418 | 0.363580019 | 64.06460795 |
| 3347 | Solomon Islands | 2006 | 92.64411218 | 0.366443698 | 64.06460795 |
| 3348 | Solomon Islands | 2009 | 92.29047144 | 0.377821028 | 63.35324861 |
| 3349 | Solomon Islands | 2010 | 92.12262662 | 0.38271759 | 63.35324861 |
| 3350 | Solomon Islands | 2011 | 91.81198285 | 0.388055337 | 62.92622144 |
| 3351 | Solomon Islands | 2016 | 89.66901862 | 0.410406962 | 62.92622144 |
| 3352 | Solomon Islands | 2019 | 88.9388227 | 0.422736386 | 62.92622144 |
| 3353 | Solomon Islands | 2021 | 86.84421477 | 0.429360316 | 62.92622144 |
| 3354 | Somalia | 1990 | 258.2963994 | 0.048848564 | 258.2963994 |
| 3355 | Somalia | 1994 | 257.3180272 | 0.051471696 | 257.3180272 |
| 3356 | Somalia | 1998 | 253.4076988 | 0.054321527 | 253.4076988 |
| 3357 | Somalia | 1999 | 252.1104893 | 0.055071838 | 252.1104893 |
| 3358 | Somalia | 2000 | 251.1925903 | 0.055842063 | 251.1925903 |
| 3359 | Somalia | 2001 | 250.5053711 | 0.056643165 | 250.5053711 |
| 3360 | Somalia | 2006 | 247.4289797 | 0.060741484 | 247.4289797 |
| 3361 | Somalia | 2007 | 246.9211757 | 0.061598151 | 246.9211757 |
| 3362 | Somalia | 2013 | 245.5041638 | 0.067459486 | 245.5041638 |
| 3363 | Somalia | 2014 | 245.8188092 | 0.068609299 | 245.5041638 |
| 3364 | Somalia | 2016 | 245.9925692 | 0.071064047 | 245.5041638 |
| 3365 | Somalia | 2017 | 245.9787437 | 0.072334776 | 245.5041638 |
| 3366 | Somalia | 2018 | 245.9637603 | 0.073642248 | 245.5041638 |
| 3367 | Somalia | 2019 | 245.9744588 | 0.074979811 | 245.5041638 |
| 3368 | Somalia | 2020 | 245.8903477 | 0.07636337 | 245.5041638 |
| 3369 | Somalia | 2021 | 246.2731243 | 0.077688109 | 245.5041638 |
| 3370 | South Africa | 1991 | 195.1553137 | 0.546921136 | 51.75814966 |
| 3371 | South Africa | 1992 | 194.7712425 | 0.552066899 | 51.75814966 |
| 3372 | South Africa | 1994 | 194.1376307 | 0.562448636 | 51.75814966 |
| 3373 | South Africa | 1995 | 193.8874248 | 0.567905304 | 51.75814966 |
| 3374 | South Africa | 1996 | 193.5920922 | 0.573453988 | 51.75814966 |
| 3375 | South Africa | 1998 | 193.0005636 | 0.584437313 | 51.75814966 |
| 3376 | South Africa | 1999 | 192.7991973 | 0.589407349 | 51.75814966 |
| 3377 | South Africa | 2001 | 193.0320756 | 0.597884168 | 51.75814966 |
| 3378 | South Africa | 2002 | 193.6937448 | 0.600921676 | 51.75814966 |
| 3379 | South Africa | 2003 | 194.5361081 | 0.603515602 | 51.75814966 |
| 3380 | South Africa | 2004 | 195.3851673 | 0.606262514 | 51.75814966 |
| 3381 | South Africa | 2007 | 197.1844295 | 0.62060815 | 32.04043631 |
| 3382 | South Africa | 2010 | 199.4560921 | 0.635268538 | 22.46369183 |
| 3383 | South Africa | 2011 | 200.4814202 | 0.639991722 | 22.46369183 |
| 3384 | South Africa | 2012 | 201.7291296 | 0.644715661 | 22.46369183 |
| 3385 | South Africa | 2014 | 204.2998503 | 0.653964495 | 22.46369183 |
| 3386 | South Africa | 2017 | 209.1525128 | 0.666622608 | 22.46369183 |
| 3387 | South Africa | 2018 | 211.4167957 | 0.670335365 | 22.46369183 |
| 3388 | South Africa | 2019 | 212.8407957 | 0.674041037 | 22.03195463 |
| 3389 | South Africa | 2020 | 212.9816485 | 0.677166096 | 22.03195463 |
| 3390 | South Africa | 2021 | 212.4220655 | 0.679626598 | 22.03195463 |
| 3391 | South Sudan | 1990 | 304.0671402 | 0.2066565 | 101.1599627 |
| 3392 | South Sudan | 1991 | 304.442926 | 0.208932716 | 101.1599627 |
| 3393 | South Sudan | 1993 | 304.8418927 | 0.213416471 | 101.1599627 |
| 3394 | South Sudan | 1997 | 304.3180902 | 0.223037171 | 101.1599627 |
| 3395 | South Sudan | 2002 | 301.7999163 | 0.238341186 | 101.1599627 |
| 3396 | South Sudan | 2003 | 301.038764 | 0.241797191 | 100.8275782 |
| 3397 | South Sudan | 2005 | 299.9488944 | 0.249280904 | 100.8275782 |
| 3398 | South Sudan | 2007 | 299.4629999 | 0.257491764 | 100.8275782 |
| 3399 | South Sudan | 2008 | 299.1782583 | 0.261740458 | 100.8275782 |
| 3400 | South Sudan | 2009 | 298.918886 | 0.266179235 | 100.8275782 |
| 3401 | South Sudan | 2010 | 298.7257126 | 0.270572622 | 100.8275782 |
| 3402 | South Sudan | 2011 | 298.7577368 | 0.274719143 | 100.8275782 |
| 3403 | South Sudan | 2013 | 299.274162 | 0.275813942 | 100.8275782 |
| 3404 | South Sudan | 2014 | 299.4577594 | 0.276311461 | 100.8275782 |
| 3405 | South Sudan | 2015 | 299.3799711 | 0.275833634 | 100.8275782 |
| 3406 | South Sudan | 2017 | 298.5800539 | 0.274387859 | 100.8275782 |
| 3407 | South Sudan | 2018 | 298.1122359 | 0.274987848 | 100.8275782 |
| 3408 | South Sudan | 2020 | 299.2287153 | 0.276787924 | 100.8275782 |
| 3409 | South Sudan | 2021 | 300.0306408 | 0.278371125 | 100.8275782 |
| 3410 | Spain | 1990 | 44.45544001 | 0.636673166 | 22.46369183 |
| 3411 | Spain | 1991 | 44.85402772 | 0.644130745 | 22.46369183 |
| 3412 | Spain | 1993 | 46.01175557 | 0.658637569 | 22.46369183 |
| 3413 | Spain | 1997 | 50.34934835 | 0.682429497 | 17.2540694 |
| 3414 | Spain | 2000 | 54.71624643 | 0.697056031 | 17.2540694 |
| 3415 | Spain | 2001 | 55.20831909 | 0.70172607 | 17.2540694 |
| 3416 | Spain | 2002 | 55.55450235 | 0.706309655 | 17.2540694 |
| 3417 | Spain | 2004 | 56.28297001 | 0.71488623 | 17.2540694 |
| 3418 | Spain | 2005 | 56.86230529 | 0.718717289 | 17.2540694 |
| 3419 | Spain | 2006 | 58.4482864 | 0.721970128 | 15.06516239 |
| 3420 | Spain | 2007 | 61.34123413 | 0.725211443 | 14.77524801 |
| 3421 | Spain | 2009 | 68.34850682 | 0.733956055 | 14.22935117 |
| 3422 | Spain | 2010 | 71.09528494 | 0.738352055 | 13.44730416 |
| 3423 | Spain | 2011 | 73.22240941 | 0.74209645 | 13.44730416 |
| 3424 | Spain | 2012 | 75.28453044 | 0.745181867 | 13.44730416 |
| 3425 | Spain | 2013 | 77.23983884 | 0.747651982 | 13.44730416 |
| 3426 | Spain | 2014 | 79.03369183 | 0.749800929 | 13.44730416 |
| 3427 | Spain | 2015 | 80.62593115 | 0.752269823 | 13.44730416 |
| 3428 | Spain | 2016 | 82.16540875 | 0.754757939 | 10.45018691 |
| 3429 | Spain | 2017 | 83.69971713 | 0.757663276 | 10.45018691 |
| 3430 | Spain | 2018 | 84.95684734 | 0.760659575 | 9.92126395 |
| 3431 | Spain | 2019 | 85.66386793 | 0.763942918 | 9.92126395 |
| 3432 | Sri Lanka | 1992 | 172.8546619 | 0.533345535 | 53.31984512 |
| 3433 | Sri Lanka | 1994 | 172.7116356 | 0.545124551 | 51.75814966 |
| 3434 | Sri Lanka | 1997 | 171.4856883 | 0.566128422 | 51.75814966 |
| 3435 | Sri Lanka | 1998 | 170.5490451 | 0.572326187 | 51.75814966 |
| 3436 | Sri Lanka | 1999 | 169.5162941 | 0.577630048 | 51.75814966 |
| 3437 | Sri Lanka | 2000 | 168.503086 | 0.582942638 | 51.75814966 |
| 3438 | Sri Lanka | 2003 | 163.6455886 | 0.597410284 | 51.75814966 |
| 3439 | Sri Lanka | 2004 | 161.8387689 | 0.602250062 | 51.75814966 |
| 3440 | Sri Lanka | 2005 | 160.3515428 | 0.606906656 | 51.75814966 |
| 3441 | Sri Lanka | 2006 | 158.9061303 | 0.611807083 | 51.75814966 |
| 3442 | Sri Lanka | 2009 | 154.6213854 | 0.62814963 | 22.46369183 |
| 3443 | Sri Lanka | 2012 | 155.8730247 | 0.648049816 | 22.46369183 |
| 3444 | Sri Lanka | 2013 | 157.3569013 | 0.655435838 | 22.46369183 |
| 3445 | Sri Lanka | 2018 | 161.9402467 | 0.688125858 | 17.2540694 |
| 3446 | Sri Lanka | 2019 | 162.4450372 | 0.693516063 | 17.2540694 |
| 3447 | Sri Lanka | 2020 | 162.5875982 | 0.697648535 | 17.2540694 |
| 3448 | Sri Lanka | 2021 | 164.756616 | 0.701534935 | 17.2540694 |
| 3449 | Sudan | 1990 | 234.4863488 | 0.292178643 | 100.8275782 |
| 3450 | Sudan | 1991 | 232.8272499 | 0.296473446 | 100.8275782 |
| 3451 | Sudan | 1992 | 231.3062047 | 0.300863677 | 100.8275782 |
| 3452 | Sudan | 1994 | 229.0423129 | 0.31009643 | 89.96318282 |
| 3453 | Sudan | 1995 | 228.4988376 | 0.315208259 | 67.17014806 |
| 3454 | Sudan | 1997 | 227.8584368 | 0.326761462 | 66.46502371 |
| 3455 | Sudan | 1999 | 227.4354167 | 0.339754884 | 65.82455226 |
| 3456 | Sudan | 2000 | 227.3991716 | 0.346875809 | 65.82455226 |
| 3457 | Sudan | 2001 | 227.4691474 | 0.354215515 | 65.82455226 |
| 3458 | Sudan | 2003 | 227.9434234 | 0.369693653 | 64.06460795 |
| 3459 | Sudan | 2005 | 228.8209729 | 0.386685712 | 63.35324861 |
| 3460 | Sudan | 2008 | 232.7945307 | 0.4160876 | 62.92622144 |
| 3461 | Sudan | 2009 | 234.373931 | 0.425975917 | 62.92622144 |
| 3462 | Sudan | 2011 | 236.4596711 | 0.447239442 | 62.92622144 |
| 3463 | Sudan | 2012 | 237.2886175 | 0.456790265 | 62.92622144 |
| 3464 | Sudan | 2014 | 238.9029395 | 0.476362135 | 53.58976548 |
| 3465 | Sudan | 2016 | 240.9674305 | 0.496848219 | 53.58976548 |
| 3466 | Sudan | 2018 | 244.2585545 | 0.516214086 | 53.58976548 |
| 3467 | Sudan | 2019 | 245.195637 | 0.525009122 | 53.58976548 |
| 3468 | Sudan | 2020 | 244.8871097 | 0.533455422 | 53.31984512 |
| 3469 | Sudan | 2021 | 239.0388002 | 0.541949735 | 53.31984512 |
| 3470 | Suriname | 1990 | 189.5231018 | 0.502054305 | 53.58976548 |
| 3471 | Suriname | 1992 | 189.4111781 | 0.510959772 | 53.58976548 |
| 3472 | Suriname | 1993 | 189.5139826 | 0.514419437 | 53.58976548 |
| 3473 | Suriname | 1996 | 190.341022 | 0.525059318 | 53.58976548 |
| 3474 | Suriname | 1998 | 192.5405798 | 0.534599696 | 53.31984512 |
| 3475 | Suriname | 1999 | 193.8336326 | 0.538932791 | 53.31984512 |
| 3476 | Suriname | 2001 | 196.324123 | 0.548683524 | 51.75814966 |
| 3477 | Suriname | 2002 | 198.1722948 | 0.553879432 | 51.75814966 |
| 3478 | Suriname | 2003 | 200.2081438 | 0.559418373 | 51.75814966 |
| 3479 | Suriname | 2004 | 202.1098579 | 0.565109708 | 51.75814966 |
| 3480 | Suriname | 2006 | 204.7138014 | 0.575647048 | 51.75814966 |
| 3481 | Suriname | 2008 | 207.0732231 | 0.585644825 | 51.75814966 |
| 3482 | Suriname | 2010 | 208.8397153 | 0.595469745 | 51.75814966 |
| 3483 | Suriname | 2013 | 210.3439919 | 0.608910612 | 51.75814966 |
| 3484 | Suriname | 2015 | 211.257629 | 0.616097315 | 32.04043631 |
| 3485 | Suriname | 2020 | 214.8098725 | 0.630683705 | 22.46369183 |
| 3486 | Sweden | 1990 | 43.90491097 | 0.785535792 | 8.445315313 |
| 3487 | Sweden | 1991 | 42.89573246 | 0.790096441 | 8.445315313 |
| 3488 | Sweden | 1993 | 42.11113503 | 0.801009522 | 8.445315313 |
| 3489 | Sweden | 1994 | 42.19821347 | 0.806700264 | 8.445315313 |
| 3490 | Sweden | 1995 | 42.51378638 | 0.812244654 | 8.445315313 |
| 3491 | Sweden | 1996 | 43.84300841 | 0.817227025 | 8.445315313 |
| 3492 | Sweden | 1999 | 54.50461057 | 0.829487099 | 8.445315313 |
| 3493 | Sweden | 2000 | 58.47086444 | 0.833028112 | 8.445315313 |
| 3494 | Sweden | 2002 | 67.42327009 | 0.838741527 | 8.445315313 |
| 3495 | Sweden | 2004 | 77.50079095 | 0.844506882 | 8.445315313 |
| 3496 | Sweden | 2005 | 82.0422525 | 0.846876344 | 8.445315313 |
| 3497 | Sweden | 2007 | 90.30254233 | 0.851420847 | 8.445315313 |
| 3498 | Sweden | 2008 | 94.30587882 | 0.853705851 | 8.445315313 |
| 3499 | Sweden | 2009 | 98.22225464 | 0.855702748 | 8.445315313 |
| 3500 | Sweden | 2010 | 102.0276117 | 0.858727941 | 8.445315313 |
| 3501 | Sweden | 2011 | 105.7586452 | 0.861892379 | 8.445315313 |
| 3502 | Sweden | 2014 | 116.6730482 | 0.869923325 | 8.445315313 |
| 3503 | Sweden | 2015 | 120.1712323 | 0.872388515 | 8.445315313 |
| 3504 | Sweden | 2016 | 124.3443235 | 0.874661655 | 8.445315313 |
| 3505 | Sweden | 2018 | 133.1466549 | 0.879888912 | 8.445315313 |
| 3506 | Sweden | 2019 | 135.2557764 | 0.882984116 | 8.445315313 |
| 3507 | Sweden | 2020 | 131.8863545 | 0.88506201 | 8.445315313 |
| 3508 | Sweden | 2021 | 118.7018173 | 0.886880299 | 8.445315313 |
| 3509 | Switzerland | 1990 | 45.81720439 | 0.862766844 | 8.445315313 |
| 3510 | Switzerland | 1992 | 41.73012314 | 0.867736079 | 8.445315313 |
| 3511 | Switzerland | 1993 | 40.17370824 | 0.870863558 | 8.445315313 |
| 3512 | Switzerland | 1994 | 39.0838643 | 0.873140871 | 8.445315313 |
| 3513 | Switzerland | 1995 | 38.56469711 | 0.874835331 | 8.445315313 |
| 3514 | Switzerland | 1996 | 38.44641954 | 0.876640557 | 8.445315313 |
| 3515 | Switzerland | 2000 | 39.47307956 | 0.885243531 | 8.445315313 |
| 3516 | Switzerland | 2001 | 40.69872152 | 0.888445951 | 8.445315313 |
| 3517 | Switzerland | 2003 | 46.03648284 | 0.892780807 | 8.445315313 |
| 3518 | Switzerland | 2005 | 52.2889781 | 0.89727263 | 8.445315313 |
| 3519 | Switzerland | 2006 | 55.16144971 | 0.899962305 | 8.445315313 |
| 3520 | Switzerland | 2007 | 58.20223382 | 0.902913413 | 8.445315313 |
| 3521 | Switzerland | 2008 | 61.3776008 | 0.905778454 | 8.445315313 |
| 3522 | Switzerland | 2013 | 80.66196487 | 0.917955006 | 8.445315313 |
| 3523 | Switzerland | 2015 | 86.2574797 | 0.921832991 | 8.445315313 |
| 3524 | Switzerland | 2016 | 87.07216034 | 0.923930598 | 8.445315313 |
| 3525 | Switzerland | 2017 | 87.56224392 | 0.926202291 | 8.445315313 |
| 3526 | Switzerland | 2018 | 88.06631727 | 0.92855059 | 8.445315313 |
| 3527 | Switzerland | 2019 | 88.90684004 | 0.930682102 | 8.445315313 |
| 3528 | Switzerland | 2020 | 95.03987986 | 0.932027645 | 8.445315313 |
| 3529 | Syrian Arab Republic | 1992 | 188.6239702 | 0.445904236 | 62.92622144 |
| 3530 | Syrian Arab Republic | 1993 | 188.3898709 | 0.45414786 | 62.92622144 |
| 3531 | Syrian Arab Republic | 1994 | 188.0928033 | 0.462437298 | 62.92622144 |
| 3532 | Syrian Arab Republic | 1996 | 187.1431801 | 0.478743085 | 53.58976548 |
| 3533 | Syrian Arab Republic | 1998 | 184.9377987 | 0.493707992 | 53.58976548 |
| 3534 | Syrian Arab Republic | 2000 | 182.2881609 | 0.507143686 | 53.58976548 |
| 3535 | Syrian Arab Republic | 2002 | 178.4738504 | 0.521309744 | 53.58976548 |
| 3536 | Syrian Arab Republic | 2005 | 173.4301908 | 0.549750449 | 51.75814966 |
| 3537 | Syrian Arab Republic | 2006 | 173.2293675 | 0.560484761 | 51.75814966 |
| 3538 | Syrian Arab Republic | 2007 | 173.1617749 | 0.57002812 | 51.75814966 |
| 3539 | Syrian Arab Republic | 2009 | 173.0394668 | 0.587789118 | 51.75814966 |
| 3540 | Syrian Arab Republic | 2010 | 172.824652 | 0.595967764 | 51.75814966 |
| 3541 | Syrian Arab Republic | 2012 | 171.3648948 | 0.602872601 | 51.75814966 |
| 3542 | Syrian Arab Republic | 2014 | 169.670149 | 0.60223576 | 51.75814966 |
| 3543 | Syrian Arab Republic | 2016 | 170.1155576 | 0.602911181 | 51.75814966 |
| 3544 | Syrian Arab Republic | 2018 | 173.8280175 | 0.608507164 | 51.75814966 |
| 3545 | Syrian Arab Republic | 2019 | 174.862731 | 0.613336836 | 51.75814966 |
| 3546 | Syrian Arab Republic | 2020 | 174.1866881 | 0.617727593 | 32.04043631 |
| 3547 | Taiwan (Province of China) | 1990 | 196.5659315 | 0.667633854 | 22.46369183 |
| 3548 | Taiwan (Province of China) | 1991 | 195.7469753 | 0.676537237 | 22.03195463 |
| 3549 | Taiwan (Province of China) | 1993 | 191.94297 | 0.69292413 | 17.2540694 |
| 3550 | Taiwan (Province of China) | 1994 | 189.3312205 | 0.70107875 | 17.2540694 |
| 3551 | Taiwan (Province of China) | 1995 | 186.4133018 | 0.709626821 | 17.2540694 |
| 3552 | Taiwan (Province of China) | 1996 | 181.0343769 | 0.719128415 | 15.06516239 |
| 3553 | Taiwan (Province of China) | 1998 | 163.3693359 | 0.738125787 | 13.44730416 |
| 3554 | Taiwan (Province of China) | 1999 | 156.0813467 | 0.745338399 | 13.44730416 |
| 3555 | Taiwan (Province of China) | 2000 | 153.0319549 | 0.752926841 | 13.44730416 |
| 3556 | Taiwan (Province of China) | 2002 | 154.9200625 | 0.769008984 | 9.92126395 |
| 3557 | Taiwan (Province of China) | 2003 | 157.0041702 | 0.776955803 | 9.92126395 |
| 3558 | Taiwan (Province of China) | 2004 | 159.480318 | 0.784677978 | 8.445315313 |
| 3559 | Taiwan (Province of China) | 2005 | 162.0236375 | 0.792445129 | 8.445315313 |
| 3560 | Taiwan (Province of China) | 2006 | 165.0750797 | 0.800174675 | 8.445315313 |
| 3561 | Taiwan (Province of China) | 2007 | 169.0708784 | 0.808137694 | 8.445315313 |
| 3562 | Taiwan (Province of China) | 2008 | 173.7221264 | 0.815400691 | 8.445315313 |
| 3563 | Taiwan (Province of China) | 2009 | 178.7830313 | 0.821432279 | 8.445315313 |
| 3564 | Taiwan (Province of China) | 2011 | 190.1983766 | 0.832064074 | 8.445315313 |
| 3565 | Taiwan (Province of China) | 2012 | 197.8277712 | 0.836677665 | 8.445315313 |
| 3566 | Taiwan (Province of China) | 2013 | 205.6843273 | 0.841280463 | 8.445315313 |
| 3567 | Taiwan (Province of China) | 2016 | 220.9183164 | 0.854466038 | 8.445315313 |
| 3568 | Taiwan (Province of China) | 2018 | 226.3578835 | 0.863158361 | 8.445315313 |
| 3569 | Taiwan (Province of China) | 2019 | 227.9293121 | 0.86741185 | 8.445315313 |
| 3570 | Tajikistan | 1991 | 169.8887465 | 0.472342173 | 53.58976548 |
| 3571 | Tajikistan | 1992 | 169.7887995 | 0.475745195 | 53.58976548 |
| 3572 | Tajikistan | 1994 | 170.4016084 | 0.478726011 | 53.58976548 |
| 3573 | Tajikistan | 1995 | 171.2157203 | 0.477586868 | 53.58976548 |
| 3574 | Tajikistan | 1996 | 172.6977815 | 0.474346027 | 53.58976548 |
| 3575 | Tajikistan | 1997 | 174.8186424 | 0.470274796 | 53.58976548 |
| 3576 | Tajikistan | 1999 | 178.8720168 | 0.460505616 | 62.92622144 |
| 3577 | Tajikistan | 2002 | 176.3403262 | 0.45880778 | 62.92622144 |
| 3578 | Tajikistan | 2004 | 170.693481 | 0.469154214 | 53.58976548 |
| 3579 | Tajikistan | 2005 | 169.5214197 | 0.474637786 | 53.58976548 |
| 3580 | Tajikistan | 2006 | 170.2028152 | 0.479651831 | 53.58976548 |
| 3581 | Tajikistan | 2008 | 174.020696 | 0.488441136 | 53.58976548 |
| 3582 | Tajikistan | 2009 | 175.8751654 | 0.492064041 | 53.58976548 |
| 3583 | Tajikistan | 2010 | 176.7312398 | 0.495893964 | 53.58976548 |
| 3584 | Tajikistan | 2012 | 175.0594648 | 0.503953885 | 53.58976548 |
| 3585 | Tajikistan | 2013 | 173.550569 | 0.507970676 | 53.58976548 |
| 3586 | Tajikistan | 2014 | 172.3130958 | 0.511989518 | 53.58976548 |
| 3587 | Tajikistan | 2016 | 172.0840942 | 0.519897002 | 53.58976548 |
| 3588 | Tajikistan | 2017 | 172.4940782 | 0.524492607 | 53.58976548 |
| 3589 | Tajikistan | 2020 | 171.9569212 | 0.537690531 | 53.31984512 |
| 3590 | Tajikistan | 2021 | 163.2467665 | 0.541511187 | 53.31984512 |
| 3591 | Thailand | 1991 | 63.82653699 | 0.515911466 | 53.58976548 |
| 3592 | Thailand | 1993 | 64.33413733 | 0.534303709 | 53.31984512 |
| 3593 | Thailand | 1995 | 64.57411224 | 0.552452209 | 51.75814966 |
| 3594 | Thailand | 1997 | 64.53837009 | 0.568449492 | 51.75814966 |
| 3595 | Thailand | 1999 | 64.48231873 | 0.578854168 | 51.75814966 |
| 3596 | Thailand | 2000 | 64.5153477 | 0.583500917 | 51.75814966 |
| 3597 | Thailand | 2001 | 64.63646202 | 0.587661561 | 51.75814966 |
| 3598 | Thailand | 2003 | 65.14376294 | 0.59604892 | 51.75814966 |
| 3599 | Thailand | 2004 | 65.49493774 | 0.600520665 | 51.75814966 |
| 3600 | Thailand | 2007 | 67.48997783 | 0.616368591 | 32.04043631 |
| 3601 | Thailand | 2008 | 68.66693068 | 0.621067543 | 32.04043631 |
| 3602 | Thailand | 2011 | 72.15181923 | 0.633226728 | 22.46369183 |
| 3603 | Thailand | 2016 | 77.8053812 | 0.659369303 | 22.46369183 |
| 3604 | Thailand | 2018 | 79.73536325 | 0.670225587 | 22.46369183 |
| 3605 | Thailand | 2019 | 80.21200471 | 0.675273901 | 22.03195463 |
| 3606 | Thailand | 2021 | 82.71091541 | 0.682547933 | 17.2540694 |
| 3607 | Timor-Leste | 1991 | 191.4178656 | 0.270943349 | 100.8275782 |
| 3608 | Timor-Leste | 1992 | 191.1347459 | 0.27991676 | 100.8275782 |
| 3609 | Timor-Leste | 1994 | 190.3930225 | 0.298763189 | 100.8275782 |
| 3610 | Timor-Leste | 1996 | 189.4090089 | 0.31758875 | 67.17014806 |
| 3611 | Timor-Leste | 1997 | 188.6356769 | 0.326705374 | 66.46502371 |
| 3612 | Timor-Leste | 1998 | 187.7073297 | 0.334894497 | 65.82455226 |
| 3613 | Timor-Leste | 1999 | 186.6886681 | 0.33925214 | 65.82455226 |
| 3614 | Timor-Leste | 2000 | 185.6352386 | 0.343793649 | 65.82455226 |
| 3615 | Timor-Leste | 2004 | 179.1714746 | 0.362687998 | 64.06460795 |
| 3616 | Timor-Leste | 2005 | 178.1176571 | 0.366913584 | 64.06460795 |
| 3617 | Timor-Leste | 2006 | 177.658407 | 0.370654092 | 64.06460795 |
| 3618 | Timor-Leste | 2008 | 177.4790269 | 0.381227528 | 63.35324861 |
| 3619 | Timor-Leste | 2011 | 176.5631615 | 0.401872356 | 62.92622144 |
| 3620 | Timor-Leste | 2012 | 175.7143053 | 0.4089103 | 62.92622144 |
| 3621 | Timor-Leste | 2013 | 174.8323639 | 0.415217878 | 62.92622144 |
| 3622 | Timor-Leste | 2014 | 174.1293878 | 0.421155538 | 62.92622144 |
| 3623 | Timor-Leste | 2016 | 173.9125278 | 0.431207272 | 62.92622144 |
| 3624 | Timor-Leste | 2017 | 174.0900473 | 0.434519734 | 62.92622144 |
| 3625 | Timor-Leste | 2018 | 174.2848458 | 0.437018663 | 62.92622144 |
| 3626 | Timor-Leste | 2020 | 174.0968501 | 0.442349336 | 62.92622144 |
| 3627 | Timor-Leste | 2021 | 178.2206535 | 0.444667619 | 62.92622144 |
| 3628 | Togo | 1991 | 256.9001424 | 0.274530568 | 100.8275782 |
| 3629 | Togo | 1994 | 256.0363016 | 0.284653387 | 100.8275782 |
| 3630 | Togo | 1995 | 255.7643255 | 0.288536692 | 100.8275782 |
| 3631 | Togo | 1996 | 255.3780382 | 0.292606677 | 100.8275782 |
| 3632 | Togo | 1997 | 254.7449175 | 0.296947835 | 100.8275782 |
| 3633 | Togo | 1998 | 254.069227 | 0.300022333 | 100.8275782 |
| 3634 | Togo | 2000 | 253.2522154 | 0.305472963 | 89.96318282 |
| 3635 | Togo | 2003 | 254.3051236 | 0.313792064 | 67.91401256 |
| 3636 | Togo | 2007 | 255.5070773 | 0.324382497 | 66.46502371 |
| 3637 | Togo | 2008 | 255.7776248 | 0.327743136 | 65.82455226 |
| 3638 | Togo | 2010 | 256.4543684 | 0.335899583 | 65.82455226 |
| 3639 | Togo | 2012 | 258.2992074 | 0.346320495 | 65.82455226 |
| 3640 | Togo | 2013 | 259.5806307 | 0.352278605 | 65.82455226 |
| 3641 | Togo | 2014 | 260.9621576 | 0.358661442 | 64.06460795 |
| 3642 | Togo | 2016 | 262.6011597 | 0.372910028 | 64.06460795 |
| 3643 | Togo | 2017 | 263.1019284 | 0.380336231 | 63.35324861 |
| 3644 | Togo | 2018 | 263.6044811 | 0.387921229 | 62.92622144 |
| 3645 | Togo | 2020 | 266.7229216 | 0.402356922 | 62.92622144 |
| 3646 | Togo | 2021 | 267.9620502 | 0.408533695 | 62.92622144 |
| 3647 | Tokelau | 1991 | 137.1578226 | 0.526628473 | 53.58976548 |
| 3648 | Tokelau | 1992 | 136.1237392 | 0.53130785 | 53.58976548 |
| 3649 | Tokelau | 1996 | 132.6924919 | 0.550282652 | 51.75814966 |
| 3650 | Tokelau | 1997 | 131.8773413 | 0.555631326 | 51.75814966 |
| 3651 | Tokelau | 1998 | 131.1425509 | 0.561047611 | 51.75814966 |
| 3652 | Tokelau | 2000 | 129.7038669 | 0.57181437 | 51.75814966 |
| 3653 | Tokelau | 2001 | 128.8912066 | 0.576915597 | 51.75814966 |
| 3654 | Tokelau | 2002 | 127.9817215 | 0.581858317 | 51.75814966 |
| 3655 | Tokelau | 2004 | 126.1960807 | 0.592342442 | 51.75814966 |
| 3656 | Tokelau | 2005 | 125.3483684 | 0.598176117 | 51.75814966 |
| 3657 | Tokelau | 2007 | 123.6532458 | 0.611163237 | 51.75814966 |
| 3658 | Tokelau | 2010 | 121.5172272 | 0.63017554 | 22.46369183 |
| 3659 | Tokelau | 2012 | 120.2489724 | 0.641611991 | 22.46369183 |
| 3660 | Tokelau | 2013 | 119.6636092 | 0.647966148 | 22.46369183 |
| 3661 | Tokelau | 2014 | 119.1995132 | 0.653231999 | 22.46369183 |
| 3662 | Tokelau | 2015 | 118.9382585 | 0.658798995 | 22.46369183 |
| 3663 | Tokelau | 2016 | 118.9191908 | 0.663974319 | 22.46369183 |
| 3664 | Tokelau | 2017 | 119.0639948 | 0.669054134 | 22.46369183 |
| 3665 | Tokelau | 2019 | 119.4509335 | 0.679247839 | 22.03195463 |
| 3666 | Tokelau | 2020 | 119.5136675 | 0.682937378 | 17.2540694 |
| 3667 | Tokelau | 2021 | 120.181388 | 0.686425621 | 17.2540694 |
| 3668 | Tonga | 1991 | 78.27151513 | 0.498166205 | 53.58976548 |
| 3669 | Tonga | 1993 | 77.78303982 | 0.50890445 | 53.58976548 |
| 3670 | Tonga | 1995 | 77.54520954 | 0.52021502 | 53.58976548 |
| 3671 | Tonga | 1996 | 77.74950351 | 0.525808135 | 53.58976548 |
| 3672 | Tonga | 1998 | 78.98718412 | 0.536172314 | 53.31984512 |
| 3673 | Tonga | 2000 | 79.61452515 | 0.546303195 | 51.75814966 |
| 3674 | Tonga | 2001 | 79.29219237 | 0.551054251 | 51.75814966 |
| 3675 | Tonga | 2002 | 78.77597282 | 0.556008345 | 51.75814966 |
| 3676 | Tonga | 2003 | 78.17850258 | 0.560736066 | 51.75814966 |
| 3677 | Tonga | 2007 | 76.39535968 | 0.57435151 | 51.75814966 |
| 3678 | Tonga | 2008 | 76.02976253 | 0.577305415 | 51.75814966 |
| 3679 | Tonga | 2010 | 75.35776073 | 0.580657299 | 51.75814966 |
| 3680 | Tonga | 2011 | 74.9424852 | 0.582656723 | 51.75814966 |
| 3681 | Tonga | 2013 | 73.83953811 | 0.587351141 | 51.75814966 |
| 3682 | Tonga | 2014 | 73.39817301 | 0.590826853 | 51.75814966 |
| 3683 | Tonga | 2016 | 73.2831062 | 0.600343669 | 51.75814966 |
| 3684 | Tonga | 2019 | 74.12620297 | 0.617324063 | 32.04043631 |
| 3685 | Tonga | 2020 | 74.37342862 | 0.622104456 | 32.04043631 |
| 3686 | Trinidad and Tobago | 1990 | 203.1124792 | 0.62397015 | 32.04043631 |
| 3687 | Trinidad and Tobago | 1991 | 200.9826771 | 0.628758918 | 22.46369183 |
| 3688 | Trinidad and Tobago | 1992 | 199.0197702 | 0.634039552 | 22.46369183 |
| 3689 | Trinidad and Tobago | 1993 | 197.2770725 | 0.639191914 | 22.46369183 |
| 3690 | Trinidad and Tobago | 1994 | 195.817837 | 0.64424291 | 22.46369183 |
| 3691 | Trinidad and Tobago | 1996 | 193.8122599 | 0.654169455 | 22.46369183 |
| 3692 | Trinidad and Tobago | 1997 | 192.9738305 | 0.659293633 | 22.46369183 |
| 3693 | Trinidad and Tobago | 1998 | 192.2828333 | 0.664507714 | 22.46369183 |
| 3694 | Trinidad and Tobago | 1999 | 191.7792644 | 0.670244574 | 22.46369183 |
| 3695 | Trinidad and Tobago | 2002 | 191.5817403 | 0.688872271 | 17.2540694 |
| 3696 | Trinidad and Tobago | 2004 | 192.0753998 | 0.702610809 | 17.2540694 |
| 3697 | Trinidad and Tobago | 2007 | 193.077503 | 0.720196417 | 15.06516239 |
| 3698 | Trinidad and Tobago | 2008 | 193.4490628 | 0.725720702 | 14.77524801 |
| 3699 | Trinidad and Tobago | 2010 | 194.0966062 | 0.733237077 | 14.22935117 |
| 3700 | Trinidad and Tobago | 2011 | 194.3830476 | 0.736802405 | 13.44730416 |
| 3701 | Trinidad and Tobago | 2012 | 194.7055722 | 0.739999073 | 13.44730416 |
| 3702 | Trinidad and Tobago | 2013 | 195.027628 | 0.743593955 | 13.44730416 |
| 3703 | Trinidad and Tobago | 2014 | 195.3293477 | 0.747338963 | 13.44730416 |
| 3704 | Trinidad and Tobago | 2015 | 195.603236 | 0.751510682 | 13.44730416 |
| 3705 | Trinidad and Tobago | 2016 | 195.9312893 | 0.754953348 | 10.45018691 |
| 3706 | Trinidad and Tobago | 2017 | 196.3441287 | 0.758226988 | 9.92126395 |
| 3707 | Trinidad and Tobago | 2019 | 197.1749596 | 0.764169599 | 9.92126395 |
| 3708 | Trinidad and Tobago | 2020 | 198.2886134 | 0.766422081 | 9.92126395 |
| 3709 | Trinidad and Tobago | 2021 | 197.6856107 | 0.768763254 | 9.92126395 |
| 3710 | Tunisia | 1990 | 264.2297339 | 0.471138521 | 53.58976548 |
| 3711 | Tunisia | 1991 | 262.2257306 | 0.479543057 | 53.58976548 |
| 3712 | Tunisia | 1992 | 260.3917228 | 0.488285281 | 53.58976548 |
| 3713 | Tunisia | 1993 | 258.8416043 | 0.496852039 | 53.58976548 |
| 3714 | Tunisia | 1994 | 257.6784628 | 0.505564882 | 53.58976548 |
| 3715 | Tunisia | 1996 | 256.611621 | 0.523294274 | 53.58976548 |
| 3716 | Tunisia | 1999 | 255.9802983 | 0.548724563 | 51.75814966 |
| 3717 | Tunisia | 2000 | 256.0973239 | 0.55667265 | 51.75814966 |
| 3718 | Tunisia | 2001 | 256.7301148 | 0.56442101 | 51.75814966 |
| 3719 | Tunisia | 2002 | 257.9937169 | 0.571719534 | 51.75814966 |
| 3720 | Tunisia | 2004 | 261.3115278 | 0.586032594 | 51.75814966 |
| 3721 | Tunisia | 2006 | 264.3867118 | 0.599758513 | 51.75814966 |
| 3722 | Tunisia | 2007 | 266.2517616 | 0.606696784 | 51.75814966 |
| 3723 | Tunisia | 2008 | 268.2147114 | 0.613529322 | 51.75814966 |
| 3724 | Tunisia | 2010 | 271.5759382 | 0.626432774 | 32.04043631 |
| 3725 | Tunisia | 2012 | 274.3772735 | 0.637578345 | 22.46369183 |
| 3726 | Tunisia | 2014 | 277.0997475 | 0.648297395 | 22.46369183 |
| 3727 | Tunisia | 2015 | 278.1764023 | 0.653413538 | 22.46369183 |
| 3728 | Tunisia | 2019 | 282.7680579 | 0.673170552 | 22.03195463 |
| 3729 | Turkmenistan | 1990 | 62.62333694 | 0.563126887 | 51.75814966 |
| 3730 | Turkmenistan | 1991 | 62.56255222 | 0.564939386 | 51.75814966 |
| 3731 | Turkmenistan | 1992 | 62.61428422 | 0.565587505 | 51.75814966 |
| 3732 | Turkmenistan | 1994 | 62.95720822 | 0.567288976 | 51.75814966 |
| 3733 | Turkmenistan | 1995 | 63.20130348 | 0.567198635 | 51.75814966 |
| 3734 | Turkmenistan | 1998 | 65.63770978 | 0.564405264 | 51.75814966 |
| 3735 | Turkmenistan | 1999 | 66.69749429 | 0.564716006 | 51.75814966 |
| 3736 | Turkmenistan | 2000 | 67.56526312 | 0.567421033 | 51.75814966 |
| 3737 | Turkmenistan | 2001 | 68.23431819 | 0.571043819 | 51.75814966 |
| 3738 | Turkmenistan | 2002 | 68.86907182 | 0.575596166 | 51.75814966 |
| 3739 | Turkmenistan | 2003 | 69.50703134 | 0.580820126 | 51.75814966 |
| 3740 | Turkmenistan | 2004 | 70.18880577 | 0.586712349 | 51.75814966 |
| 3741 | Turkmenistan | 2005 | 70.95491376 | 0.593388895 | 51.75814966 |
| 3742 | Turkmenistan | 2007 | 74.00848868 | 0.606042198 | 51.75814966 |
| 3743 | Turkmenistan | 2009 | 77.91413598 | 0.617652905 | 32.04043631 |
| 3744 | Turkmenistan | 2010 | 79.2400843 | 0.623234589 | 32.04043631 |
| 3745 | Turkmenistan | 2014 | 82.87776175 | 0.647542417 | 22.46369183 |
| 3746 | Turkmenistan | 2015 | 83.34308443 | 0.65335279 | 22.46369183 |
| 3747 | Turkmenistan | 2016 | 83.63416456 | 0.658945613 | 22.46369183 |
| 3748 | Turkmenistan | 2017 | 83.90656673 | 0.664284692 | 22.46369183 |
| 3749 | Turkmenistan | 2018 | 84.11676025 | 0.669433599 | 22.46369183 |
| 3750 | Turkmenistan | 2021 | 82.04849793 | 0.682160776 | 17.2540694 |
| 3751 | Tuvalu | 1990 | 133.3595837 | 0.406247566 | 62.92622144 |
| 3752 | Tuvalu | 1991 | 132.634399 | 0.414819937 | 62.92622144 |
| 3753 | Tuvalu | 1994 | 130.5386975 | 0.438563051 | 62.92622144 |
| 3754 | Tuvalu | 1995 | 129.9106759 | 0.444962242 | 62.92622144 |
| 3755 | Tuvalu | 1996 | 129.2711952 | 0.44980498 | 62.92622144 |
| 3756 | Tuvalu | 1998 | 127.890429 | 0.462454678 | 62.92622144 |
| 3757 | Tuvalu | 1999 | 127.2273262 | 0.468845802 | 53.58976548 |
| 3758 | Tuvalu | 2000 | 126.612546 | 0.474956823 | 53.58976548 |
| 3759 | Tuvalu | 2001 | 125.9468767 | 0.480850886 | 53.58976548 |
| 3760 | Tuvalu | 2006 | 122.3434973 | 0.505975411 | 53.58976548 |
| 3761 | Tuvalu | 2007 | 121.6864136 | 0.510504301 | 53.58976548 |
| 3762 | Tuvalu | 2010 | 120.0117608 | 0.52345551 | 53.58976548 |
| 3763 | Tuvalu | 2013 | 118.6523444 | 0.535166235 | 53.31984512 |
| 3764 | Tuvalu | 2015 | 118.1318669 | 0.54404788 | 51.75814966 |
| 3765 | Tuvalu | 2018 | 118.065286 | 0.559859395 | 51.75814966 |
| 3766 | Tuvalu | 2019 | 118.092926 | 0.566045489 | 51.75814966 |
| 3767 | Tuvalu | 2020 | 118.3907909 | 0.571666102 | 51.75814966 |
| 3768 | Tuvalu | 2021 | 118.7885445 | 0.576620529 | 51.75814966 |
| 3769 | Uganda | 1991 | 296.8036151 | 0.188924156 | 237.6563045 |
| 3770 | Uganda | 1992 | 295.5993553 | 0.191003433 | 237.6563045 |
| 3771 | Uganda | 1993 | 294.5031517 | 0.193557456 | 237.6563045 |
| 3772 | Uganda | 1994 | 293.5727758 | 0.197293193 | 237.6563045 |
| 3773 | Uganda | 1996 | 292.1986558 | 0.207080731 | 101.1599627 |
| 3774 | Uganda | 1997 | 291.5001396 | 0.213091696 | 101.1599627 |
| 3775 | Uganda | 2000 | 290.2146194 | 0.234323225 | 101.1599627 |
| 3776 | Uganda | 2001 | 291.1011223 | 0.242186388 | 100.8275782 |
| 3777 | Uganda | 2003 | 296.1849795 | 0.259164883 | 100.8275782 |
| 3778 | Uganda | 2004 | 298.9317324 | 0.268004611 | 100.8275782 |
| 3779 | Uganda | 2006 | 302.1073774 | 0.287030583 | 100.8275782 |
| 3780 | Uganda | 2007 | 303.3911692 | 0.29674042 | 100.8275782 |
| 3781 | Uganda | 2008 | 304.6697592 | 0.306748843 | 89.96318282 |
| 3782 | Uganda | 2010 | 307.1316574 | 0.326543661 | 66.46502371 |
| 3783 | Uganda | 2011 | 308.667033 | 0.336217716 | 65.82455226 |
| 3784 | Uganda | 2014 | 314.3081452 | 0.363430903 | 64.06460795 |
| 3785 | Uganda | 2016 | 314.9226429 | 0.381312226 | 63.35324861 |
| 3786 | Uganda | 2018 | 313.0764777 | 0.399117777 | 62.92622144 |
| 3787 | Uganda | 2020 | 315.6267396 | 0.416846079 | 62.92622144 |
| 3788 | Uganda | 2021 | 316.2563874 | 0.423261181 | 62.92622144 |
| 3789 | Ukraine | 1992 | 31.16848201 | 0.657600333 | 22.46369183 |
| 3790 | Ukraine | 1993 | 30.83139897 | 0.662867405 | 22.46369183 |
| 3791 | Ukraine | 1994 | 30.58974151 | 0.665506375 | 22.46369183 |
| 3792 | Ukraine | 1995 | 30.4622112 | 0.666950167 | 22.46369183 |
| 3793 | Ukraine | 1996 | 30.45007219 | 0.668104744 | 22.46369183 |
| 3794 | Ukraine | 1997 | 30.50816393 | 0.669207846 | 22.46369183 |
| 3795 | Ukraine | 1998 | 30.58639334 | 0.670322301 | 22.46369183 |
| 3796 | Ukraine | 2000 | 30.6233343 | 0.671095001 | 22.46369183 |
| 3797 | Ukraine | 2002 | 30.17142969 | 0.674543221 | 22.03195463 |
| 3798 | Ukraine | 2003 | 29.84478935 | 0.678614636 | 22.03195463 |
| 3799 | Ukraine | 2007 | 30.14224706 | 0.704202308 | 17.2540694 |
| 3800 | Ukraine | 2009 | 31.38328207 | 0.716185465 | 17.2540694 |
| 3801 | Ukraine | 2011 | 32.14169821 | 0.724891957 | 15.06516239 |
| 3802 | Ukraine | 2012 | 32.44847677 | 0.7288172 | 14.22935117 |
| 3803 | Ukraine | 2013 | 32.75673431 | 0.734033573 | 14.22935117 |
| 3804 | Ukraine | 2014 | 33.06734528 | 0.739883557 | 13.44730416 |
| 3805 | Ukraine | 2020 | 35.00190698 | 0.759929872 | 9.92126395 |
| 3806 | United Arab Emirates | 1990 | 193.5689883 | 0.644412271 | 22.46369183 |
| 3807 | United Arab Emirates | 1991 | 195.1054305 | 0.660739899 | 22.46369183 |
| 3808 | United Arab Emirates | 1992 | 196.6299613 | 0.675926211 | 22.03195463 |
| 3809 | United Arab Emirates | 1993 | 198.10573 | 0.689463801 | 17.2540694 |
| 3810 | United Arab Emirates | 1994 | 199.5067895 | 0.701656792 | 17.2540694 |
| 3811 | United Arab Emirates | 1996 | 202.1657993 | 0.724181976 | 15.06516239 |
| 3812 | United Arab Emirates | 1998 | 205.2865681 | 0.744476623 | 13.44730416 |
| 3813 | United Arab Emirates | 1999 | 206.7966317 | 0.753253916 | 13.44730416 |
| 3814 | United Arab Emirates | 2000 | 208.1415406 | 0.760969094 | 9.92126395 |
| 3815 | United Arab Emirates | 2001 | 209.560095 | 0.767858793 | 9.92126395 |
| 3816 | United Arab Emirates | 2002 | 211.214852 | 0.774346424 | 9.92126395 |
| 3817 | United Arab Emirates | 2003 | 212.9747647 | 0.781143992 | 9.92126395 |
| 3818 | United Arab Emirates | 2004 | 214.7810399 | 0.788525951 | 8.445315313 |
| 3819 | United Arab Emirates | 2005 | 216.5393126 | 0.796428086 | 8.445315313 |
| 3820 | United Arab Emirates | 2008 | 220.7426729 | 0.822793435 | 8.445315313 |
| 3821 | United Arab Emirates | 2009 | 222.2545992 | 0.828304165 | 8.445315313 |
| 3822 | United Arab Emirates | 2010 | 223.9711893 | 0.831284254 | 8.445315313 |
| 3823 | United Arab Emirates | 2011 | 225.9839793 | 0.832912804 | 8.445315313 |
| 3824 | United Arab Emirates | 2012 | 228.3178971 | 0.83358611 | 8.445315313 |
| 3825 | United Arab Emirates | 2013 | 230.7777218 | 0.833803818 | 8.445315313 |
| 3826 | United Arab Emirates | 2015 | 235.2146777 | 0.834175498 | 8.445315313 |
| 3827 | United Arab Emirates | 2016 | 236.9696483 | 0.83522071 | 8.445315313 |
| 3828 | United Arab Emirates | 2017 | 238.6779719 | 0.837213553 | 8.445315313 |
| 3829 | United Arab Emirates | 2018 | 240.1549335 | 0.83990014 | 8.445315313 |
| 3830 | United Arab Emirates | 2019 | 241.2842011 | 0.842985326 | 8.445315313 |
| 3831 | United Arab Emirates | 2020 | 240.6873491 | 0.846151976 | 8.445315313 |
| 3832 | United Arab Emirates | 2021 | 247.083437 | 0.849317734 | 8.445315313 |
| 3833 | United Kingdom | 1993 | 21.18106149 | 0.76015435 | 9.92126395 |
| 3834 | United Kingdom | 1994 | 21.51163063 | 0.7651646 | 9.92126395 |
| 3835 | United Kingdom | 1996 | 22.11056274 | 0.771576484 | 9.92126395 |
| 3836 | United Kingdom | 1997 | 22.42177037 | 0.775050122 | 9.92126395 |
| 3837 | United Kingdom | 1999 | 23.10977234 | 0.784036475 | 8.445315313 |
| 3838 | United Kingdom | 2000 | 23.49530973 | 0.78921896 | 8.445315313 |
| 3839 | United Kingdom | 2001 | 24.01123914 | 0.793513165 | 8.445315313 |
| 3840 | United Kingdom | 2003 | 25.46033344 | 0.799186111 | 8.445315313 |
| 3841 | United Kingdom | 2004 | 26.21024899 | 0.80195087 | 8.445315313 |
| 3842 | United Kingdom | 2005 | 26.86238786 | 0.804489194 | 8.445315313 |
| 3843 | United Kingdom | 2006 | 27.47836677 | 0.806817046 | 8.445315313 |
| 3844 | United Kingdom | 2007 | 28.15314563 | 0.809352772 | 8.445315313 |
| 3845 | United Kingdom | 2009 | 29.54548126 | 0.814793642 | 8.445315313 |
| 3846 | United Kingdom | 2010 | 30.20087665 | 0.818033994 | 8.445315313 |
| 3847 | United Kingdom | 2014 | 33.80947954 | 0.835516642 | 8.445315313 |
| 3848 | United Kingdom | 2015 | 34.37135949 | 0.838951919 | 8.445315313 |
| 3849 | United Kingdom | 2016 | 34.69585184 | 0.842460233 | 8.445315313 |
| 3850 | United Kingdom | 2017 | 34.9675426 | 0.846263618 | 8.445315313 |
| 3851 | United Republic of Tanzania | 1992 | 298.1076943 | 0.264989448 | 100.8275782 |
| 3852 | United Republic of Tanzania | 1993 | 298.0626577 | 0.267254915 | 100.8275782 |
| 3853 | United Republic of Tanzania | 1997 | 296.7082954 | 0.279259184 | 100.8275782 |
| 3854 | United Republic of Tanzania | 1999 | 295.4439367 | 0.287381679 | 100.8275782 |
| 3855 | United Republic of Tanzania | 2002 | 301.2903727 | 0.301983995 | 89.96318282 |
| 3856 | United Republic of Tanzania | 2004 | 310.5930926 | 0.313772983 | 67.91401256 |
| 3857 | United Republic of Tanzania | 2005 | 312.3542059 | 0.320437339 | 66.46502371 |
| 3858 | United Republic of Tanzania | 2006 | 311.7797705 | 0.327234002 | 65.82455226 |
| 3859 | United Republic of Tanzania | 2008 | 308.3968249 | 0.34135263 | 65.82455226 |
| 3860 | United Republic of Tanzania | 2009 | 306.3882916 | 0.348378035 | 65.82455226 |
| 3861 | United Republic of Tanzania | 2010 | 304.6940044 | 0.355567563 | 65.82455226 |
| 3862 | United Republic of Tanzania | 2011 | 302.7727386 | 0.36303632 | 64.06460795 |
| 3863 | United Republic of Tanzania | 2012 | 300.1709282 | 0.370412488 | 64.06460795 |
| 3864 | United Republic of Tanzania | 2014 | 295.3795097 | 0.385974579 | 63.35324861 |
| 3865 | United Republic of Tanzania | 2015 | 294.4202674 | 0.394277757 | 62.92622144 |
| 3866 | United Republic of Tanzania | 2017 | 295.1442109 | 0.411824974 | 62.92622144 |
| 3867 | United Republic of Tanzania | 2018 | 295.9062539 | 0.420985364 | 62.92622144 |
| 3868 | United States of America | 1990 | 97.29419651 | 0.76364769 | 9.92126395 |
| 3869 | United States of America | 1991 | 97.75209599 | 0.766044295 | 9.92126395 |
| 3870 | United States of America | 1992 | 98.25850802 | 0.769595492 | 9.92126395 |
| 3871 | United States of America | 1994 | 99.3481493 | 0.776368588 | 9.92126395 |
| 3872 | United States of America | 1995 | 99.90305503 | 0.779410254 | 9.92126395 |
| 3873 | United States of America | 1998 | 102.3963951 | 0.786076332 | 8.445315313 |
| 3874 | United States of America | 2001 | 105.4225024 | 0.796676793 | 8.445315313 |
| 3875 | United States of America | 2003 | 110.0444784 | 0.803228025 | 8.445315313 |
| 3876 | United States of America | 2004 | 112.7592502 | 0.805304692 | 8.445315313 |
| 3877 | United States of America | 2005 | 115.324373 | 0.80603308 | 8.445315313 |
| 3878 | United States of America | 2007 | 122.550045 | 0.80968805 | 8.445315313 |
| 3879 | United States of America | 2008 | 127.0779523 | 0.814577102 | 8.445315313 |
| 3880 | United States of America | 2009 | 131.3831765 | 0.820294574 | 8.445315313 |
| 3881 | United States of America | 2010 | 134.8455165 | 0.825884658 | 8.445315313 |
| 3882 | United States of America | 2011 | 137.7745488 | 0.830266388 | 8.445315313 |
| 3883 | United States of America | 2012 | 140.7833132 | 0.833958192 | 8.445315313 |
| 3884 | United States of America | 2013 | 143.6886757 | 0.837412988 | 8.445315313 |
| 3885 | United States of America | 2014 | 146.3146914 | 0.840746528 | 8.445315313 |
| 3886 | United States of America | 2015 | 148.4837328 | 0.844370336 | 8.445315313 |
| 3887 | United States of America | 2018 | 153.7810772 | 0.855049193 | 8.445315313 |
| 3888 | United States of America | 2020 | 156.0602366 | 0.860792773 | 8.445315313 |
| 3889 | United States of America | 2021 | 157.5215441 | 0.862448354 | 8.445315313 |
| 3890 | United States Virgin Islands | 1990 | 169.1788933 | 0.655160856 | 22.46369183 |
| 3891 | United States Virgin Islands | 1995 | 172.0930997 | 0.686730211 | 17.2540694 |
| 3892 | United States Virgin Islands | 1998 | 173.5521729 | 0.699768686 | 17.2540694 |
| 3893 | United States Virgin Islands | 1999 | 174.0618503 | 0.703949342 | 17.2540694 |
| 3894 | United States Virgin Islands | 2000 | 174.6363041 | 0.708436565 | 17.2540694 |
| 3895 | United States Virgin Islands | 2002 | 176.5711631 | 0.724398272 | 15.06516239 |
| 3896 | United States Virgin Islands | 2003 | 177.7009238 | 0.73460985 | 14.22935117 |
| 3897 | United States Virgin Islands | 2005 | 179.133637 | 0.754267558 | 10.45018691 |
| 3898 | United States Virgin Islands | 2006 | 179.3071194 | 0.763447496 | 9.92126395 |
| 3899 | United States Virgin Islands | 2007 | 179.4319862 | 0.772180593 | 9.92126395 |
| 3900 | United States Virgin Islands | 2008 | 179.5619134 | 0.780342623 | 9.92126395 |
| 3901 | United States Virgin Islands | 2009 | 179.7260664 | 0.787055146 | 8.445315313 |
| 3902 | United States Virgin Islands | 2010 | 179.9578422 | 0.793219696 | 8.445315313 |
| 3903 | United States Virgin Islands | 2011 | 180.5549293 | 0.798071698 | 8.445315313 |
| 3904 | United States Virgin Islands | 2015 | 184.4743372 | 0.807889245 | 8.445315313 |
| 3905 | United States Virgin Islands | 2019 | 185.6069073 | 0.817429008 | 8.445315313 |
| 3906 | United States Virgin Islands | 2020 | 185.2529276 | 0.819601712 | 8.445315313 |
| 3907 | United States Virgin Islands | 2021 | 186.8034027 | 0.821830853 | 8.445315313 |
| 3908 | Uruguay | 1990 | 60.0973215 | 0.581921855 | 51.75814966 |
| 3909 | Uruguay | 1992 | 60.39717477 | 0.589822355 | 51.75814966 |
| 3910 | Uruguay | 1994 | 61.07588655 | 0.595188424 | 51.75814966 |
| 3911 | Uruguay | 1995 | 61.50526678 | 0.596825409 | 51.75814966 |
| 3912 | Uruguay | 1996 | 62.19564213 | 0.599775104 | 51.75814966 |
| 3913 | Uruguay | 1997 | 63.24550716 | 0.605665051 | 51.75814966 |
| 3914 | Uruguay | 2000 | 66.80509707 | 0.623208291 | 32.04043631 |
| 3915 | Uruguay | 2001 | 67.82049304 | 0.625796068 | 32.04043631 |
| 3916 | Uruguay | 2005 | 72.67318995 | 0.63697577 | 22.46369183 |
| 3917 | Uruguay | 2007 | 75.43444167 | 0.646331714 | 22.46369183 |
| 3918 | Uruguay | 2008 | 76.95211158 | 0.653525522 | 22.46369183 |
| 3919 | Uruguay | 2009 | 78.43867156 | 0.660321135 | 22.46369183 |
| 3920 | Uruguay | 2010 | 79.80316765 | 0.666099135 | 22.46369183 |
| 3921 | Uruguay | 2011 | 81.15409066 | 0.667079733 | 22.46369183 |
| 3922 | Uruguay | 2015 | 86.41386366 | 0.680595506 | 17.2540694 |
| 3923 | Uruguay | 2016 | 87.6973178 | 0.689816733 | 17.2540694 |
| 3924 | Uruguay | 2018 | 90.64268685 | 0.70690493 | 17.2540694 |
| 3925 | Uruguay | 2019 | 91.32504505 | 0.712536588 | 17.2540694 |
| 3926 | Uzbekistan | 1990 | 73.57564294 | 0.500241735 | 53.58976548 |
| 3927 | Uzbekistan | 1991 | 72.57041638 | 0.501544609 | 53.58976548 |
| 3928 | Uzbekistan | 1992 | 71.74456094 | 0.502905194 | 53.58976548 |
| 3929 | Uzbekistan | 1994 | 70.76157516 | 0.511018525 | 53.58976548 |
| 3930 | Uzbekistan | 1997 | 71.40161455 | 0.532686328 | 53.31984512 |
| 3931 | Uzbekistan | 1999 | 72.60856307 | 0.549739391 | 51.75814966 |
| 3932 | Uzbekistan | 2004 | 71.72083854 | 0.583942986 | 51.75814966 |
| 3933 | Uzbekistan | 2005 | 71.77377482 | 0.589530777 | 51.75814966 |
| 3934 | Uzbekistan | 2006 | 72.62149029 | 0.594498057 | 51.75814966 |
| 3935 | Uzbekistan | 2007 | 74.24556385 | 0.599628055 | 51.75814966 |
| 3936 | Uzbekistan | 2009 | 78.0963364 | 0.610351745 | 51.75814966 |
| 3937 | Uzbekistan | 2010 | 79.42440671 | 0.616097907 | 32.04043631 |
| 3938 | Uzbekistan | 2012 | 81.4843791 | 0.626202125 | 32.04043631 |
| 3939 | Uzbekistan | 2013 | 82.52343319 | 0.630796403 | 22.46369183 |
| 3940 | Uzbekistan | 2016 | 84.77287619 | 0.644230597 | 22.46369183 |
| 3941 | Uzbekistan | 2021 | 87.76744855 | 0.662621694 | 22.46369183 |
| 3942 | Vanuatu | 1990 | 94.87141434 | 0.353100252 | 65.82455226 |
| 3943 | Vanuatu | 1991 | 94.13741636 | 0.357005379 | 64.06460795 |
| 3944 | Vanuatu | 1997 | 92.4162134 | 0.380667171 | 63.35324861 |
| 3945 | Vanuatu | 1999 | 92.64747042 | 0.388300526 | 62.92622144 |
| 3946 | Vanuatu | 2000 | 92.7380363 | 0.392158349 | 62.92622144 |
| 3947 | Vanuatu | 2001 | 92.76636965 | 0.395325055 | 62.92622144 |
| 3948 | Vanuatu | 2003 | 92.71938532 | 0.400396456 | 62.92622144 |
| 3949 | Vanuatu | 2004 | 92.69332458 | 0.403226097 | 62.92622144 |
| 3950 | Vanuatu | 2005 | 92.7112595 | 0.406488572 | 62.92622144 |
| 3951 | Vanuatu | 2006 | 92.79113759 | 0.410501729 | 62.92622144 |
| 3952 | Vanuatu | 2008 | 93.03325218 | 0.419853237 | 62.92622144 |
| 3953 | Vanuatu | 2009 | 93.13108564 | 0.42485465 | 62.92622144 |
| 3954 | Vanuatu | 2010 | 93.1823694 | 0.429658049 | 62.92622144 |
| 3955 | Vanuatu | 2012 | 93.04238694 | 0.438661998 | 62.92622144 |
| 3956 | Vanuatu | 2014 | 92.81733203 | 0.446687298 | 62.92622144 |
| 3957 | Vanuatu | 2015 | 92.80513068 | 0.450329861 | 62.92622144 |
| 3958 | Vanuatu | 2018 | 93.44329177 | 0.462080221 | 62.92622144 |
| 3959 | Vanuatu | 2019 | 93.67439716 | 0.466276162 | 62.92622144 |
| 3960 | Vanuatu | 2020 | 94.15297935 | 0.470038391 | 53.58976548 |
| 3961 | Vanuatu | 2021 | 94.37539912 | 0.473100706 | 53.58976548 |
| 3962 | Venezuela (Bolivarian Republic of) | 1990 | 187.7351459 | 0.516891218 | 53.58976548 |
| 3963 | Venezuela (Bolivarian Republic of) | 1993 | 188.3331108 | 0.536279978 | 53.31984512 |
| 3964 | Venezuela (Bolivarian Republic of) | 1994 | 188.3297725 | 0.541866058 | 53.31984512 |
| 3965 | Venezuela (Bolivarian Republic of) | 1996 | 188.047755 | 0.548649226 | 51.75814966 |
| 3966 | Venezuela (Bolivarian Republic of) | 1997 | 187.7012167 | 0.552364437 | 51.75814966 |
| 3967 | Venezuela (Bolivarian Republic of) | 1998 | 187.2235674 | 0.557243117 | 51.75814966 |
| 3968 | Venezuela (Bolivarian Republic of) | 2000 | 185.9466273 | 0.56350626 | 51.75814966 |
| 3969 | Venezuela (Bolivarian Republic of) | 2003 | 182.2439 | 0.555212224 | 51.75814966 |
| 3970 | Venezuela (Bolivarian Republic of) | 2005 | 180.4700985 | 0.553941679 | 51.75814966 |
| 3971 | Venezuela (Bolivarian Republic of) | 2006 | 180.2985613 | 0.56333419 | 51.75814966 |
| 3972 | Venezuela (Bolivarian Republic of) | 2007 | 180.2454685 | 0.575367174 | 51.75814966 |
| 3973 | Venezuela (Bolivarian Republic of) | 2008 | 180.2413646 | 0.586184887 | 51.75814966 |
| 3974 | Venezuela (Bolivarian Republic of) | 2009 | 180.2130442 | 0.593288422 | 51.75814966 |
| 3975 | Venezuela (Bolivarian Republic of) | 2012 | 178.8590175 | 0.610187465 | 51.75814966 |
| 3976 | Venezuela (Bolivarian Republic of) | 2013 | 177.9060737 | 0.614441024 | 32.04043631 |
| 3977 | Venezuela (Bolivarian Republic of) | 2015 | 176.3392115 | 0.610675357 | 51.75814966 |
| 3978 | Venezuela (Bolivarian Republic of) | 2017 | 174.4807929 | 0.605160131 | 51.75814966 |
| 3979 | Venezuela (Bolivarian Republic of) | 2018 | 173.4119287 | 0.604068247 | 51.75814966 |
| 3980 | Venezuela (Bolivarian Republic of) | 2019 | 172.7222592 | 0.602313887 | 51.75814966 |
| 3981 | Venezuela (Bolivarian Republic of) | 2020 | 172.2227154 | 0.600034781 | 51.75814966 |
| 3982 | Venezuela (Bolivarian Republic of) | 2021 | 173.1969353 | 0.596513059 | 51.75814966 |
| 3983 | Viet Nam | 1991 | 215.0783663 | 0.41321725 | 62.92622144 |
| 3984 | Viet Nam | 1996 | 214.471486 | 0.45310241 | 62.92622144 |
| 3985 | Viet Nam | 1998 | 212.4146025 | 0.472478892 | 53.58976548 |
| 3986 | Viet Nam | 1999 | 211.1686654 | 0.481445774 | 53.58976548 |
| 3987 | Viet Nam | 2000 | 209.9845977 | 0.489802045 | 53.58976548 |
| 3988 | Viet Nam | 2001 | 208.6096704 | 0.497630349 | 53.58976548 |
| 3989 | Viet Nam | 2006 | 200.0783416 | 0.536300653 | 53.31984512 |
| 3990 | Viet Nam | 2007 | 198.4550246 | 0.543342652 | 53.31984512 |
| 3991 | Viet Nam | 2008 | 196.9462607 | 0.550127119 | 51.75814966 |
| 3992 | Viet Nam | 2009 | 195.8220841 | 0.556455944 | 51.75814966 |
| 3993 | Viet Nam | 2011 | 195.6554042 | 0.568945458 | 51.75814966 |
| 3994 | Viet Nam | 2014 | 198.5632798 | 0.586913815 | 51.75814966 |
| 3995 | Viet Nam | 2015 | 199.4018427 | 0.592849573 | 51.75814966 |
| 3996 | Viet Nam | 2018 | 202.4492909 | 0.611351406 | 51.75814966 |
| 3997 | Viet Nam | 2019 | 203.0932853 | 0.617733815 | 32.04043631 |
| 3998 | Viet Nam | 2020 | 203.1318486 | 0.62309032 | 32.04043631 |
| 3999 | Viet Nam | 2021 | 205.7102748 | 0.627933721 | 22.46369183 |
| 4000 | Yemen | 1991 | 250.8165113 | 0.222885409 | 101.1599627 |
| 4001 | Yemen | 1995 | 249.7866426 | 0.253962572 | 100.8275782 |
| 4002 | Yemen | 1996 | 249.9375218 | 0.262278613 | 100.8275782 |
| 4003 | Yemen | 1997 | 250.2650557 | 0.270782536 | 100.8275782 |
| 4004 | Yemen | 1999 | 251.0364948 | 0.288261301 | 100.8275782 |
| 4005 | Yemen | 2000 | 251.3129936 | 0.297145157 | 100.8275782 |
| 4006 | Yemen | 2001 | 251.4795712 | 0.306112424 | 89.96318282 |
| 4007 | Yemen | 2003 | 251.8405184 | 0.324081689 | 66.46502371 |
| 4008 | Yemen | 2004 | 252.0165291 | 0.333371285 | 65.82455226 |
| 4009 | Yemen | 2005 | 252.2099835 | 0.343462562 | 65.82455226 |
| 4010 | Yemen | 2006 | 252.4131253 | 0.353472947 | 65.82455226 |
| 4011 | Yemen | 2007 | 252.6705675 | 0.363301218 | 64.06460795 |
| 4012 | Yemen | 2011 | 253.7919104 | 0.401471801 | 62.92622144 |
| 4013 | Yemen | 2012 | 254.1308371 | 0.409561349 | 62.92622144 |
| 4014 | Yemen | 2014 | 254.7147097 | 0.425189667 | 62.92622144 |
| 4015 | Yemen | 2015 | 254.8234449 | 0.430197789 | 62.92622144 |
| 4016 | Yemen | 2017 | 254.3381637 | 0.437549155 | 62.92622144 |
| 4017 | Yemen | 2019 | 253.5995348 | 0.4440014 | 62.92622144 |
| 4018 | Yemen | 2020 | 252.9879342 | 0.446893069 | 62.92622144 |
| 4019 | Yemen | 2021 | 253.7551433 | 0.450376375 | 62.92622144 |
| 4020 | Zambia | 1991 | 293.2420581 | 0.30584236 | 89.96318282 |
| 4021 | Zambia | 1992 | 293.6413399 | 0.307316277 | 89.96318282 |
| 4022 | Zambia | 1993 | 293.9255961 | 0.308780208 | 89.96318282 |
| 4023 | Zambia | 1994 | 294.0871007 | 0.309223449 | 89.96318282 |
| 4024 | Zambia | 1995 | 294.1240009 | 0.309995813 | 89.96318282 |
| 4025 | Zambia | 1996 | 293.9754677 | 0.311326691 | 89.96318282 |
| 4026 | Zambia | 1998 | 293.2184466 | 0.314926435 | 67.17014806 |
| 4027 | Zambia | 1999 | 292.8029821 | 0.317396437 | 67.17014806 |
| 4028 | Zambia | 2001 | 292.2974414 | 0.324401368 | 66.46502371 |
| 4029 | Zambia | 2004 | 291.9712664 | 0.339488917 | 65.82455226 |
| 4030 | Zambia | 2005 | 291.8129576 | 0.346048864 | 65.82455226 |
| 4031 | Zambia | 2007 | 292.028079 | 0.362450967 | 64.06460795 |
| 4032 | Zambia | 2008 | 292.3916446 | 0.371688496 | 64.06460795 |
| 4033 | Zambia | 2009 | 292.7580595 | 0.381667118 | 63.35324861 |
| 4034 | Zambia | 2010 | 293.0106082 | 0.392470989 | 62.92622144 |
| 4035 | Zambia | 2011 | 293.4061554 | 0.403475629 | 62.92622144 |
| 4036 | Zambia | 2013 | 295.075759 | 0.425492302 | 62.92622144 |
| 4037 | Zambia | 2014 | 295.9356253 | 0.436244006 | 62.92622144 |
| 4038 | Zambia | 2015 | 296.5314971 | 0.446744719 | 62.92622144 |
| 4039 | Zambia | 2018 | 298.5479196 | 0.477887712 | 53.58976548 |
| 4040 | Zambia | 2019 | 299.2882466 | 0.487911523 | 53.58976548 |
| 4041 | Zambia | 2021 | 301.5857728 | 0.505948954 | 53.58976548 |
| 4042 | Zimbabwe | 1990 | 201.4663743 | 0.398559341 | 62.92622144 |
| 4043 | Zimbabwe | 1992 | 201.92579 | 0.412083826 | 62.92622144 |
| 4044 | Zimbabwe | 1994 | 201.8648773 | 0.424051564 | 62.92622144 |
| 4045 | Zimbabwe | 1995 | 201.6761894 | 0.42892904 | 62.92622144 |
| 4046 | Zimbabwe | 1996 | 201.264824 | 0.434187192 | 62.92622144 |
| 4047 | Zimbabwe | 1997 | 200.5517936 | 0.438141988 | 62.92622144 |
| 4048 | Zimbabwe | 1999 | 198.8933033 | 0.443246604 | 62.92622144 |
| 4049 | Zimbabwe | 2000 | 198.2126842 | 0.444841402 | 62.92622144 |
| 4050 | Zimbabwe | 2001 | 197.4205963 | 0.446354681 | 62.92622144 |
| 4051 | Zimbabwe | 2003 | 195.0592798 | 0.446002182 | 62.92622144 |
| 4052 | Zimbabwe | 2004 | 193.8814155 | 0.443696404 | 62.92622144 |
| 4053 | Zimbabwe | 2005 | 192.9541781 | 0.439651657 | 62.92622144 |
| 4054 | Zimbabwe | 2006 | 192.0598772 | 0.434405758 | 62.92622144 |
| 4055 | Zimbabwe | 2007 | 190.9936953 | 0.42916514 | 62.92622144 |
| 4056 | Zimbabwe | 2008 | 189.9542486 | 0.422448974 | 62.92622144 |
| 4057 | Zimbabwe | 2009 | 189.1898429 | 0.418330613 | 62.92622144 |
| 4058 | Zimbabwe | 2010 | 188.9168163 | 0.417305357 | 62.92622144 |
| 4059 | Zimbabwe | 2011 | 189.4225443 | 0.419729695 | 62.92622144 |
| 4060 | Zimbabwe | 2012 | 190.5776949 | 0.42574852 | 62.92622144 |
| 4061 | Zimbabwe | 2013 | 191.9615213 | 0.432881888 | 62.92622144 |
| 4062 | Zimbabwe | 2014 | 193.1991683 | 0.439997226 | 62.92622144 |
| 4063 | Zimbabwe | 2015 | 193.8940168 | 0.446552872 | 62.92622144 |
| 4064 | Zimbabwe | 2016 | 194.1470058 | 0.452473037 | 62.92622144 |
| 4065 | Zimbabwe | 2017 | 194.3253719 | 0.458424984 | 62.92622144 |
| 4066 | Zimbabwe | 2019 | 194.6167649 | 0.468384438 | 53.58976548 |
| 4067 | Zimbabwe | 2020 | 195.5754528 | 0.471573171 | 53.58976548 |
| 4068 | NA | NA | NA | NA | NA |

Table S6 Global age-standardized DALY burden and frontier analysis of hypertensive heart disease (HHD), 1990-2021

|  | | | | | |
| --- | --- | --- | --- | --- | --- |
|  | location | year | ASR | SDI | frontier |
| 1 | Afghanistan | 1990 | 1996.881177 | 0.173832165 | 514.5647214 |
| 2 | Afghanistan | 1993 | 1966.391622 | 0.180183713 | 514.5647214 |
| 3 | Afghanistan | 1994 | 1986.024816 | 0.17851866 | 514.5647214 |
| 4 | Afghanistan | 1997 | 1998.230545 | 0.177884124 | 514.5647214 |
| 5 | Afghanistan | 1998 | 1998.954938 | 0.177513403 | 514.5647214 |
| 6 | Afghanistan | 2000 | 2014.051362 | 0.177025772 | 514.5647214 |
| 7 | Afghanistan | 2001 | 2020.171998 | 0.177773144 | 514.5647214 |
| 8 | Afghanistan | 2002 | 1989.609757 | 0.18380843 | 514.5647214 |
| 9 | Afghanistan | 2003 | 1975.708953 | 0.190518991 | 514.5647214 |
| 10 | Afghanistan | 2004 | 1960.443613 | 0.196903541 | 514.5647214 |
| 11 | Afghanistan | 2007 | 1862.58255 | 0.219627611 | 514.5647214 |
| 12 | Afghanistan | 2010 | 1751.414645 | 0.247759949 | 353.8472094 |
| 13 | Afghanistan | 2012 | 1684.138921 | 0.266484407 | 326.8700368 |
| 14 | Afghanistan | 2014 | 1610.05805 | 0.284030351 | 310.7641057 |
| 15 | Afghanistan | 2015 | 1600.511149 | 0.291849505 | 310.7641057 |
| 16 | Afghanistan | 2017 | 1576.122914 | 0.307424618 | 310.7641057 |
| 17 | Afghanistan | 2018 | 1559.374992 | 0.314866093 | 299.4410496 |
| 18 | Albania | 1990 | 243.123318 | 0.5577733 | 80.26046855 |
| 19 | Albania | 1994 | 186.1594655 | 0.553763669 | 80.26046855 |
| 20 | Albania | 1996 | 187.6426216 | 0.562248886 | 80.26046855 |
| 21 | Albania | 1998 | 182.1967055 | 0.572534102 | 80.26046855 |
| 22 | Albania | 1999 | 182.5577009 | 0.578295462 | 80.26046855 |
| 23 | Albania | 2000 | 186.6027888 | 0.584664188 | 80.26046855 |
| 24 | Albania | 2001 | 187.1283507 | 0.592412947 | 80.26046855 |
| 25 | Albania | 2002 | 192.5011921 | 0.599865978 | 80.26046855 |
| 26 | Albania | 2003 | 198.6950959 | 0.606971166 | 80.26046855 |
| 27 | Albania | 2005 | 193.1009537 | 0.620679615 | 76.77579561 |
| 28 | Albania | 2007 | 169.9806509 | 0.634167969 | 67.56319773 |
| 29 | Albania | 2010 | 162.5551305 | 0.652654203 | 66.26296628 |
| 30 | Albania | 2011 | 162.5300289 | 0.658196773 | 66.26296628 |
| 31 | Albania | 2014 | 170.0737337 | 0.674112985 | 66.0451484 |
| 32 | Albania | 2015 | 170.6140603 | 0.679668601 | 66.0451484 |
| 33 | Albania | 2016 | 172.1640839 | 0.685036648 | 66.0451484 |
| 34 | Albania | 2018 | 169.3787568 | 0.695566878 | 66.0451484 |
| 35 | Albania | 2019 | 166.6851784 | 0.700287863 | 66.0451484 |
| 36 | Albania | 2020 | 171.6547567 | 0.703790245 | 66.0451484 |
| 37 | Albania | 2021 | 160.4195009 | 0.706849791 | 66.0451484 |
| 38 | Algeria | 1992 | 1010.990243 | 0.475950937 | 88.47846997 |
| 39 | Algeria | 1993 | 987.8928506 | 0.483255591 | 88.47846997 |
| 40 | Algeria | 1995 | 943.6542046 | 0.497957543 | 88.47846997 |
| 41 | Algeria | 1996 | 923.2169331 | 0.505990967 | 88.47846997 |
| 42 | Algeria | 1997 | 902.6921327 | 0.514056566 | 88.47846997 |
| 43 | Algeria | 2000 | 863.278782 | 0.539481508 | 81.47775441 |
| 44 | Algeria | 2001 | 856.1653394 | 0.547529277 | 80.26046855 |
| 45 | Algeria | 2003 | 871.0213886 | 0.562536956 | 80.26046855 |
| 46 | Algeria | 2008 | 878.0717259 | 0.593190973 | 80.26046855 |
| 47 | Algeria | 2009 | 870.8345705 | 0.597870384 | 80.26046855 |
| 48 | Algeria | 2011 | 868.0983704 | 0.607825529 | 80.26046855 |
| 49 | Algeria | 2012 | 850.0465661 | 0.612703533 | 79.88452611 |
| 50 | Algeria | 2014 | 824.3777234 | 0.622087292 | 76.77579561 |
| 51 | Algeria | 2015 | 842.4583965 | 0.626745568 | 71.37611055 |
| 52 | Algeria | 2016 | 798.6128868 | 0.631710736 | 71.37611055 |
| 53 | Algeria | 2017 | 788.8616627 | 0.636973176 | 67.56319773 |
| 54 | Algeria | 2019 | 794.5416261 | 0.648210785 | 66.26296628 |
| 55 | Algeria | 2020 | 779.1957712 | 0.653651466 | 66.26296628 |
| 56 | Algeria | 2021 | 775.2522059 | 0.659500924 | 66.26296628 |
| 57 | American Samoa | 1990 | 407.7709001 | 0.613633924 | 79.88452611 |
| 58 | American Samoa | 1992 | 407.8715526 | 0.618583654 | 76.77579561 |
| 59 | American Samoa | 1993 | 410.6302958 | 0.622078598 | 76.77579561 |
| 60 | American Samoa | 1994 | 418.2747693 | 0.626053241 | 71.37611055 |
| 61 | American Samoa | 1995 | 423.1045102 | 0.629814054 | 71.37611055 |
| 62 | American Samoa | 1997 | 435.6982029 | 0.634926685 | 67.56319773 |
| 63 | American Samoa | 1999 | 356.1434881 | 0.638322193 | 67.56319773 |
| 64 | American Samoa | 2000 | 340.56618 | 0.64060955 | 66.26296628 |
| 65 | American Samoa | 2001 | 322.2634064 | 0.643214421 | 66.26296628 |
| 66 | American Samoa | 2002 | 320.1403945 | 0.646002062 | 66.26296628 |
| 67 | American Samoa | 2004 | 319.2851931 | 0.651921141 | 66.26296628 |
| 68 | American Samoa | 2005 | 319.758446 | 0.655061005 | 66.26296628 |
| 69 | American Samoa | 2007 | 312.2983147 | 0.660781176 | 66.26296628 |
| 70 | American Samoa | 2010 | 297.670323 | 0.671248916 | 66.0451484 |
| 71 | American Samoa | 2011 | 294.7483235 | 0.675979288 | 66.0451484 |
| 72 | American Samoa | 2012 | 293.1955635 | 0.680861539 | 66.0451484 |
| 73 | American Samoa | 2013 | 295.8437819 | 0.685833061 | 66.0451484 |
| 74 | American Samoa | 2014 | 298.1454149 | 0.691343137 | 66.0451484 |
| 75 | American Samoa | 2015 | 300.5366708 | 0.697162912 | 66.0451484 |
| 76 | American Samoa | 2018 | 300.2806219 | 0.712800591 | 66.0451484 |
| 77 | American Samoa | 2019 | 296.595028 | 0.717308133 | 66.0451484 |
| 78 | American Samoa | 2021 | 293.4936755 | 0.723727533 | 66.0451484 |
| 79 | Andorra | 1990 | 169.9306874 | 0.76146388 | 36.49887056 |
| 80 | Andorra | 1991 | 167.3867009 | 0.764709071 | 36.49887056 |
| 81 | Andorra | 1994 | 165.2355073 | 0.770023765 | 36.49887056 |
| 82 | Andorra | 1996 | 159.1234239 | 0.772403471 | 24.95431148 |
| 83 | Andorra | 1998 | 154.0200164 | 0.776972578 | 24.95431148 |
| 84 | Andorra | 2000 | 150.2460627 | 0.781976506 | 24.95431148 |
| 85 | Andorra | 2003 | 138.3315217 | 0.797278901 | 24.95431148 |
| 86 | Andorra | 2005 | 133.1778586 | 0.81221398 | 24.95431148 |
| 87 | Andorra | 2007 | 128.3376829 | 0.826617254 | 24.95431148 |
| 88 | Andorra | 2009 | 130.2922355 | 0.835929887 | 24.95431148 |
| 89 | Andorra | 2014 | 132.3063039 | 0.851010492 | 24.95431148 |
| 90 | Andorra | 2015 | 130.9987633 | 0.853591218 | 24.95431148 |
| 91 | Andorra | 2017 | 130.5109907 | 0.85947703 | 24.95431148 |
| 92 | Andorra | 2019 | 125.2359459 | 0.865621024 | 23.90520825 |
| 93 | Andorra | 2021 | 103.0557725 | 0.869444113 | 23.90520825 |
| 94 | Angola | 1992 | 1599.272764 | 0.2792315 | 326.8700368 |
| 95 | Angola | 1993 | 1604.746968 | 0.281867703 | 310.7641057 |
| 96 | Angola | 1994 | 1606.698848 | 0.284487594 | 310.7641057 |
| 97 | Angola | 1995 | 1586.483207 | 0.287447813 | 310.7641057 |
| 98 | Angola | 1997 | 1510.147907 | 0.295433993 | 310.7641057 |
| 99 | Angola | 1998 | 1519.037488 | 0.299553353 | 310.7641057 |
| 100 | Angola | 2000 | 1494.542695 | 0.307603312 | 310.7641057 |
| 101 | Angola | 2002 | 1424.933715 | 0.316706571 | 299.4410496 |
| 102 | Angola | 2004 | 1404.091626 | 0.327395266 | 154.9592349 |
| 103 | Angola | 2005 | 1362.003718 | 0.333785188 | 154.9592349 |
| 104 | Angola | 2006 | 1339.968477 | 0.341012622 | 154.9592349 |
| 105 | Angola | 2007 | 1301.651363 | 0.348846841 | 154.9592349 |
| 106 | Angola | 2008 | 1290.817088 | 0.357082005 | 114.1556344 |
| 107 | Angola | 2010 | 1261.180733 | 0.372033537 | 114.1556344 |
| 108 | Angola | 2011 | 1245.661128 | 0.380006613 | 96.4751219 |
| 109 | Angola | 2012 | 1229.611256 | 0.387891857 | 96.4751219 |
| 110 | Angola | 2014 | 1172.20813 | 0.403720086 | 96.4751219 |
| 111 | Angola | 2016 | 1158.121834 | 0.418822062 | 96.4751219 |
| 112 | Angola | 2017 | 1162.82092 | 0.426177082 | 96.4751219 |
| 113 | Angola | 2018 | 1164.279223 | 0.433355005 | 88.47846997 |
| 114 | Angola | 2020 | 1176.919693 | 0.447283992 | 88.47846997 |
| 115 | Antigua and Barbuda | 1990 | 705.9956301 | 0.612104591 | 79.88452611 |
| 116 | Antigua and Barbuda | 1991 | 709.486149 | 0.618817129 | 76.77579561 |
| 117 | Antigua and Barbuda | 1992 | 653.3682852 | 0.624695818 | 71.37611055 |
| 118 | Antigua and Barbuda | 1993 | 676.2122682 | 0.630075854 | 71.37611055 |
| 119 | Antigua and Barbuda | 1994 | 699.4114093 | 0.634768955 | 67.56319773 |
| 120 | Antigua and Barbuda | 1995 | 764.5407375 | 0.638008629 | 67.56319773 |
| 121 | Antigua and Barbuda | 2000 | 627.1573104 | 0.660819064 | 66.26296628 |
| 122 | Antigua and Barbuda | 2002 | 630.1551533 | 0.670360801 | 66.0451484 |
| 123 | Antigua and Barbuda | 2003 | 609.0840232 | 0.67531149 | 66.0451484 |
| 124 | Antigua and Barbuda | 2004 | 579.6962936 | 0.680412429 | 66.0451484 |
| 125 | Antigua and Barbuda | 2006 | 553.5967612 | 0.690826021 | 66.0451484 |
| 126 | Antigua and Barbuda | 2007 | 623.328387 | 0.696360039 | 66.0451484 |
| 127 | Antigua and Barbuda | 2008 | 642.8835137 | 0.702175362 | 66.0451484 |
| 128 | Antigua and Barbuda | 2009 | 633.2710089 | 0.707128564 | 66.0451484 |
| 129 | Antigua and Barbuda | 2011 | 678.562 | 0.715489989 | 66.0451484 |
| 130 | Antigua and Barbuda | 2014 | 741.3297853 | 0.725585978 | 66.0451484 |
| 131 | Antigua and Barbuda | 2015 | 799.7339617 | 0.728372762 | 66.0451484 |
| 132 | Antigua and Barbuda | 2016 | 856.1595034 | 0.731598944 | 66.0451484 |
| 133 | Antigua and Barbuda | 2017 | 921.6279042 | 0.734963713 | 51.23510879 |
| 134 | Antigua and Barbuda | 2018 | 919.2486815 | 0.738879106 | 51.23510879 |
| 135 | Antigua and Barbuda | 2019 | 919.9482091 | 0.74305296 | 40.7830703 |
| 136 | Antigua and Barbuda | 2021 | 819.5379073 | 0.749886887 | 40.7830703 |
| 137 | Argentina | 1991 | 328.7227257 | 0.59170754 | 80.26046855 |
| 138 | Argentina | 1995 | 312.0823874 | 0.614473706 | 79.88452611 |
| 139 | Argentina | 1996 | 300.3629357 | 0.618942624 | 76.77579561 |
| 140 | Argentina | 1997 | 280.3807719 | 0.623934409 | 76.77579561 |
| 141 | Argentina | 1998 | 273.3969751 | 0.628723436 | 71.37611055 |
| 142 | Argentina | 1999 | 274.4980618 | 0.633175129 | 71.37611055 |
| 143 | Argentina | 2000 | 275.4406952 | 0.638194472 | 67.56319773 |
| 144 | Argentina | 2001 | 284.4588871 | 0.642175705 | 66.26296628 |
| 145 | Argentina | 2002 | 287.5003982 | 0.644472507 | 66.26296628 |
| 146 | Argentina | 2003 | 285.1921056 | 0.645906577 | 66.26296628 |
| 147 | Argentina | 2004 | 282.5572608 | 0.648296828 | 66.26296628 |
| 148 | Argentina | 2005 | 275.5907026 | 0.65336883 | 66.26296628 |
| 149 | Argentina | 2006 | 272.307045 | 0.6574459 | 66.26296628 |
| 150 | Argentina | 2007 | 276.1612705 | 0.660187576 | 66.26296628 |
| 151 | Argentina | 2009 | 277.3408831 | 0.665911944 | 66.0451484 |
| 152 | Argentina | 2010 | 287.0076754 | 0.66992588 | 66.0451484 |
| 153 | Argentina | 2013 | 264.7355349 | 0.681633215 | 66.0451484 |
| 154 | Argentina | 2014 | 258.395208 | 0.684974509 | 66.0451484 |
| 155 | Argentina | 2015 | 268.8827457 | 0.691710126 | 66.0451484 |
| 156 | Argentina | 2017 | 271.4148135 | 0.705135529 | 66.0451484 |
| 157 | Argentina | 2019 | 258.5976028 | 0.719002843 | 66.0451484 |
| 158 | Argentina | 2020 | 248.5922874 | 0.721294303 | 66.0451484 |
| 159 | Argentina | 2021 | 229.6979491 | 0.723122973 | 66.0451484 |
| 160 | Armenia | 1991 | 332.3968111 | 0.547561863 | 80.26046855 |
| 161 | Armenia | 1994 | 361.1787413 | 0.553783844 | 80.26046855 |
| 162 | Armenia | 1995 | 354.2087776 | 0.556815513 | 80.26046855 |
| 163 | Armenia | 1997 | 346.3748984 | 0.564627462 | 80.26046855 |
| 164 | Armenia | 1998 | 344.5463593 | 0.569556663 | 80.26046855 |
| 165 | Armenia | 1999 | 344.6733364 | 0.574323064 | 80.26046855 |
| 166 | Armenia | 2001 | 342.820757 | 0.585419295 | 80.26046855 |
| 167 | Armenia | 2003 | 342.6700175 | 0.60080917 | 80.26046855 |
| 168 | Armenia | 2005 | 362.666925 | 0.617759634 | 76.77579561 |
| 169 | Armenia | 2008 | 382.6398945 | 0.644431591 | 66.26296628 |
| 170 | Armenia | 2010 | 388.3320702 | 0.654811534 | 66.26296628 |
| 171 | Armenia | 2011 | 352.3390677 | 0.659818092 | 66.26296628 |
| 172 | Armenia | 2012 | 344.7607511 | 0.664833143 | 66.0451484 |
| 173 | Armenia | 2013 | 318.9545527 | 0.669534496 | 66.0451484 |
| 174 | Armenia | 2014 | 302.521717 | 0.67393219 | 66.0451484 |
| 175 | Armenia | 2015 | 307.739402 | 0.678054635 | 66.0451484 |
| 176 | Armenia | 2019 | 287.5108063 | 0.694765962 | 66.0451484 |
| 177 | Armenia | 2020 | 301.3135969 | 0.698268899 | 66.0451484 |
| 178 | Australia | 1992 | 51.23510879 | 0.733901404 | 51.23510879 |
| 179 | Australia | 1993 | 51.99719143 | 0.738374743 | 51.23510879 |
| 180 | Australia | 1995 | 48.41960406 | 0.747426824 | 40.7830703 |
| 181 | Australia | 1996 | 47.08663171 | 0.751991736 | 40.7830703 |
| 182 | Australia | 1997 | 43.34816247 | 0.756633901 | 36.49887056 |
| 183 | Australia | 1998 | 41.44981043 | 0.761133265 | 36.49887056 |
| 184 | Australia | 1999 | 38.41432522 | 0.765697506 | 36.49887056 |
| 185 | Australia | 2000 | 38.44526097 | 0.770223843 | 36.49887056 |
| 186 | Australia | 2002 | 37.24388503 | 0.77936094 | 24.95431148 |
| 187 | Australia | 2003 | 35.9550859 | 0.783600181 | 24.95431148 |
| 188 | Australia | 2004 | 34.12636631 | 0.787016543 | 24.95431148 |
| 189 | Australia | 2006 | 33.6575189 | 0.791155688 | 24.95431148 |
| 190 | Australia | 2008 | 37.26244379 | 0.7952929 | 24.95431148 |
| 191 | Australia | 2009 | 37.11947717 | 0.799174373 | 24.95431148 |
| 192 | Australia | 2011 | 36.57946801 | 0.80752022 | 24.95431148 |
| 193 | Australia | 2012 | 38.81702737 | 0.811644461 | 24.95431148 |
| 194 | Australia | 2014 | 43.79549733 | 0.82084085 | 24.95431148 |
| 195 | Australia | 2015 | 44.7765333 | 0.824860715 | 24.95431148 |
| 196 | Australia | 2016 | 42.08930477 | 0.828559943 | 24.95431148 |
| 197 | Australia | 2017 | 41.88303978 | 0.831798266 | 24.95431148 |
| 198 | Australia | 2019 | 41.07689746 | 0.839317426 | 24.95431148 |
| 199 | Australia | 2020 | 39.25344042 | 0.842051314 | 24.95431148 |
| 200 | Austria | 1991 | 154.4934649 | 0.751652734 | 40.7830703 |
| 201 | Austria | 1992 | 153.3008383 | 0.754375286 | 36.49887056 |
| 202 | Austria | 1994 | 151.9995541 | 0.764704364 | 36.49887056 |
| 203 | Austria | 1995 | 151.4630204 | 0.769827471 | 36.49887056 |
| 204 | Austria | 1997 | 157.1881674 | 0.779232856 | 24.95431148 |
| 205 | Austria | 1998 | 154.8208295 | 0.783521016 | 24.95431148 |
| 206 | Austria | 1999 | 157.2504151 | 0.787451584 | 24.95431148 |
| 207 | Austria | 2000 | 161.6288072 | 0.79184768 | 24.95431148 |
| 208 | Austria | 2001 | 156.8253295 | 0.795770118 | 24.95431148 |
| 209 | Austria | 2002 | 150.9038133 | 0.79915412 | 24.95431148 |
| 210 | Austria | 2003 | 153.6184261 | 0.802043253 | 24.95431148 |
| 211 | Austria | 2004 | 161.2933876 | 0.804889144 | 24.95431148 |
| 212 | Austria | 2006 | 168.4816113 | 0.812064609 | 24.95431148 |
| 213 | Austria | 2008 | 184.1004423 | 0.818793658 | 24.95431148 |
| 214 | Austria | 2009 | 194.7729174 | 0.821541167 | 24.95431148 |
| 215 | Austria | 2011 | 194.344359 | 0.828001999 | 24.95431148 |
| 216 | Austria | 2012 | 198.7199787 | 0.831113309 | 24.95431148 |
| 217 | Austria | 2013 | 203.220535 | 0.83367978 | 24.95431148 |
| 218 | Austria | 2014 | 204.6218145 | 0.835838577 | 24.95431148 |
| 219 | Austria | 2015 | 203.9639467 | 0.837579895 | 24.95431148 |
| 220 | Austria | 2016 | 195.5790002 | 0.83988379 | 24.95431148 |
| 221 | Austria | 2017 | 179.5395965 | 0.843173331 | 24.95431148 |
| 222 | Austria | 2018 | 173.1017051 | 0.84661447 | 24.95431148 |
| 223 | Azerbaijan | 1993 | 551.8234517 | 0.593542676 | 80.26046855 |
| 224 | Azerbaijan | 1994 | 561.5268628 | 0.592088785 | 80.26046855 |
| 225 | Azerbaijan | 1996 | 539.9432766 | 0.586315946 | 80.26046855 |
| 226 | Azerbaijan | 1997 | 526.2480493 | 0.583221813 | 80.26046855 |
| 227 | Azerbaijan | 1999 | 522.5107422 | 0.579192506 | 80.26046855 |
| 228 | Azerbaijan | 2000 | 525.732453 | 0.580020573 | 80.26046855 |
| 229 | Azerbaijan | 2001 | 527.1899655 | 0.582193708 | 80.26046855 |
| 230 | Azerbaijan | 2002 | 536.0448453 | 0.585716143 | 80.26046855 |
| 231 | Azerbaijan | 2003 | 557.1245125 | 0.590038705 | 80.26046855 |
| 232 | Azerbaijan | 2004 | 557.9009228 | 0.594646085 | 80.26046855 |
| 233 | Azerbaijan | 2005 | 558.2793757 | 0.602485193 | 80.26046855 |
| 234 | Azerbaijan | 2007 | 525.7760282 | 0.626110926 | 71.37611055 |
| 235 | Azerbaijan | 2008 | 526.1091973 | 0.636901109 | 67.56319773 |
| 236 | Azerbaijan | 2009 | 523.690608 | 0.645163341 | 66.26296628 |
| 237 | Azerbaijan | 2010 | 520.3670765 | 0.652056507 | 66.26296628 |
| 238 | Azerbaijan | 2011 | 510.8525461 | 0.657971629 | 66.26296628 |
| 239 | Azerbaijan | 2015 | 480.648231 | 0.676244911 | 66.0451484 |
| 240 | Azerbaijan | 2016 | 473.6514649 | 0.679807643 | 66.0451484 |
| 241 | Azerbaijan | 2018 | 437.865072 | 0.686561093 | 66.0451484 |
| 242 | Azerbaijan | 2020 | 421.5749588 | 0.692605192 | 66.0451484 |
| 243 | Azerbaijan | 2021 | 375.7078609 | 0.694851274 | 66.0451484 |
| 244 | Bahamas | 1992 | 1146.30732 | 0.687343838 | 66.0451484 |
| 245 | Bahamas | 1994 | 1156.56677 | 0.706819892 | 66.0451484 |
| 246 | Bahamas | 1996 | 1182.941323 | 0.728478844 | 66.0451484 |
| 247 | Bahamas | 1997 | 1219.661653 | 0.734781215 | 51.23510879 |
| 248 | Bahamas | 1998 | 1178.171534 | 0.739281293 | 51.23510879 |
| 249 | Bahamas | 2000 | 1131.179905 | 0.742517946 | 40.7830703 |
| 250 | Bahamas | 2002 | 1491.842198 | 0.746250322 | 40.7830703 |
| 251 | Bahamas | 2004 | 1356.495713 | 0.753759643 | 36.49887056 |
| 252 | Bahamas | 2005 | 1247.485895 | 0.756782661 | 36.49887056 |
| 253 | Bahamas | 2006 | 1154.812166 | 0.759185939 | 36.49887056 |
| 254 | Bahamas | 2007 | 1169.817607 | 0.761990997 | 36.49887056 |
| 255 | Bahamas | 2008 | 1221.041678 | 0.765590503 | 36.49887056 |
| 256 | Bahamas | 2015 | 1340.732938 | 0.789956145 | 24.95431148 |
| 257 | Bahamas | 2016 | 1355.738491 | 0.792738696 | 24.95431148 |
| 258 | Bahamas | 2017 | 1334.163825 | 0.795347181 | 24.95431148 |
| 259 | Bahamas | 2018 | 1320.774858 | 0.798179034 | 24.95431148 |
| 260 | Bahamas | 2019 | 1312.453425 | 0.80103307 | 24.95431148 |
| 261 | Bahamas | 2020 | 1293.339628 | 0.802948018 | 24.95431148 |
| 262 | Bahamas | 2021 | 1282.220311 | 0.805020668 | 24.95431148 |
| 263 | Bahrain | 1990 | 640.5935301 | 0.584578852 | 80.26046855 |
| 264 | Bahrain | 1992 | 642.3342272 | 0.596040229 | 80.26046855 |
| 265 | Bahrain | 1993 | 629.5640154 | 0.602165622 | 80.26046855 |
| 266 | Bahrain | 1995 | 636.9568733 | 0.613850722 | 79.88452611 |
| 267 | Bahrain | 1996 | 630.8895865 | 0.619485376 | 76.77579561 |
| 268 | Bahrain | 1997 | 627.4520166 | 0.624596062 | 71.37611055 |
| 269 | Bahrain | 1998 | 604.5265987 | 0.630738301 | 71.37611055 |
| 270 | Bahrain | 2003 | 554.4011748 | 0.669564111 | 66.0451484 |
| 271 | Bahrain | 2004 | 572.1833779 | 0.674147775 | 66.0451484 |
| 272 | Bahrain | 2005 | 572.7058193 | 0.679790598 | 66.0451484 |
| 273 | Bahrain | 2006 | 556.5133649 | 0.686144289 | 66.0451484 |
| 274 | Bahrain | 2007 | 536.4273279 | 0.693045147 | 66.0451484 |
| 275 | Bahrain | 2008 | 517.397663 | 0.699806387 | 66.0451484 |
| 276 | Bahrain | 2009 | 504.6734577 | 0.70488584 | 66.0451484 |
| 277 | Bahrain | 2010 | 514.5157919 | 0.70790262 | 66.0451484 |
| 278 | Bahrain | 2012 | 489.5680166 | 0.710362418 | 66.0451484 |
| 279 | Bahrain | 2013 | 457.3093188 | 0.713343156 | 66.0451484 |
| 280 | Bahrain | 2015 | 385.0703919 | 0.719926605 | 66.0451484 |
| 281 | Bahrain | 2016 | 392.8640988 | 0.723611297 | 66.0451484 |
| 282 | Bahrain | 2017 | 390.6317309 | 0.729323103 | 66.0451484 |
| 283 | Bahrain | 2018 | 385.0991995 | 0.736192583 | 51.23510879 |
| 284 | Bahrain | 2020 | 390.67872 | 0.748103083 | 40.7830703 |
| 285 | Bahrain | 2021 | 391.7203649 | 0.753043204 | 36.49887056 |
| 286 | Bangladesh | 1990 | 474.0773397 | 0.228548934 | 447.2303407 |
| 287 | Bangladesh | 1991 | 448.069827 | 0.237207413 | 366.2096858 |
| 288 | Bangladesh | 1992 | 446.4289157 | 0.245247956 | 353.8472094 |
| 289 | Bangladesh | 1993 | 441.0000376 | 0.25209072 | 353.8472094 |
| 290 | Bangladesh | 1996 | 431.6115305 | 0.271177781 | 326.8700368 |
| 291 | Bangladesh | 1997 | 425.9534698 | 0.27777328 | 326.8700368 |
| 292 | Bangladesh | 1998 | 420.5273508 | 0.28451594 | 310.7641057 |
[truncated: 566,121 more chars]
